# Meningeal inflammation changes the balance of TNF signalling in cortical grey matter in multiple sclerosis

**DOI:** 10.1186/s12974-019-1650-x

**Published:** 2019-12-07

**Authors:** Roberta Magliozzi, Owain William Howell, Pascal Durrenberger, Eleonora Aricò, Rachel James, Carolina Cruciani, Cheryl Reeves, Federico Roncaroli, Richard Nicholas, Richard Reynolds

**Affiliations:** 1 Department of Brain Sciences, Department of Medicine, Imperial College Faculty of Medicine, Hammersmith Hospital Campus, Imperial College London, Burlington Danes Building, Du Cane Road, London, W12 0NN UK; 2 Neurology Unit, Department of Neuroscience, Biomedicine and Movement Science, University of Verona, Policlinico G.B. Rossi, P.le L.A. Scuro, 10, 37134 Verona, Italy; 3 Institute for Life Sciences, Swansea University, Swansea, Wales; 4 FaBioCell, Core Facilities, Istituto Superiore di Sanità, Rome, Italy; 5 Great Ormond Street Hospital, London, UK; 6 Division of Neuroscience and Experimental Psychology, University of Manchester, Manchester, UK

**Keywords:** Multiple sclerosis, TNF, Meningeal inflammation, Cortical grey matter lesion, Necroptosis

## Abstract

**Background:**

Recent studies of cortical pathology in secondary progressive multiple sclerosis have shown that a more severe clinical course and the presence of extended subpial grey matter lesions with significant neuronal/glial loss and microglial activation are associated with meningeal inflammation, including the presence of lymphoid-like structures in the subarachnoid space in a proportion of cases.

**Methods:**

To investigate the molecular consequences of pro-inflammatory and cytotoxic molecules diffusing from the meninges into the underlying grey matter, we carried out gene expression profiling analysis of the motor cortex from 20 post-mortem multiple sclerosis brains with and without substantial meningeal inflammation and 10 non-neurological controls.

**Results:**

Gene expression profiling of grey matter lesions and normal appearing grey matter not only confirmed the substantial pathological cell changes, which were greatest in multiple sclerosis cases with increased meningeal inflammation, but also demonstrated the upregulation of multiple genes/pathways associated with the inflammatory response. In particular, genes involved in tumour necrosis factor (TNF) signalling were significantly deregulated in MS cases compared with controls. Increased meningeal inflammation was found to be associated with a shift in the balance of TNF signalling away from TNFR1/TNFR2 and NFkB-mediated anti-apoptotic pathways towards TNFR1- and RIPK3-mediated pro-apoptotic/pro-necroptotic signalling in the grey matter, which was confirmed by RT-PCR analysis. TNFR1 was found expressed preferentially on neurons and oligodendrocytes in MS cortical grey matter, whereas TNFR2 was predominantly expressed by astrocytes and microglia.

**Conclusions:**

We suggest that the inflammatory milieu generated in the subarachnoid space of the multiple sclerosis meninges by infiltrating immune cells leads to increased demyelinating and neurodegenerative pathology in the underlying grey matter due to changes in the balance of TNF signalling.

## Background

Multiple sclerosis (MS) has long been considered a predominantly white matter (WM) disease due to the presence of demyelinated plaques in major WM tracts, observable at a gross anatomical level and using classical histology and conventional magnetic resonance imaging (MRI). Only during the last 15 years has the true extent and clinical impact of cortical grey matter (GM) pathology been documented [3, 5, 31, 47]. Both neuroimaging and human tissue studies indicate that the extent of GM pathology correlates with disease severity and rate of progression [6, 8, 22, 51] and is initiated at the earliest stages of MS [7, 33]. Demyelinated lesions in cortical GM are accompanied by axonal pathology and neuronal and neuropil loss [36, 37, 43, 47, 60, 62] and the magnitude of this loss is associated with a shorter time to milestones of clinical progression [29, 37, 51].

There is extensive evidence from numerous studies for significant ongoing inflammation in both the WM and GM of the progressive MS brain, both SPMS and PPMS [10, 20, 23]. But in contrast to the WM, lesions in the GM are generally not accompanied by significant perivascular infiltrates or evidence of blood-brain barrier leakage [3, 59]. It is suggested that rather than an abrupt transition from the acute episodes of peripheral immune cell influx that characterise RRMS, it is likely that there is a progressive build-up of sequestered inflammation in the meningeal and WM and GM perivascular spaces that become self-sustaining as MS progresses [5, 32, 51]. However, we know relatively little about the molecular mechanisms involved in cortical tissue damage and the relationship between inflammation and neurodegeneration. Increased cortical demyelination, neuron and neurite loss is associated with greater meningeal inflammation [10, 29, 31, 33, 36, 37, 54], including the presence of aberrant tertiary lymphoid-like structures in a significant proportion of cases [29]. Moreover, a strong association between meningeal inflammation and severity of pathology has also been shown in the spinal cord ([2, 11] and in brain biopsies from patients with a recent diagnosis of MS [33]. Such lymphoid-like aggregates of immune cells are suggested to drive chronic inflammation in target organs in many other inflammatory or autoimmune conditions [40, 46, 55] by accelerating and/or maintaining the disease process. However, the molecular mechanisms by which they drive chronic disease are not well established.

Further investigation of the nature of leptomeningeal infiltrates in MS has shown that, in addition to B-lymphocyte aggregates, they contain both CD4^+^ and CD8^+^ T-lymphocytes [37], many of which express IFN-γ [53], and myeloid cells expressing TNF [24]. Gene expression for TNF and IFN-γ is increased in SPMS cases exhibiting meningeal lymphoid-like structures, together with increased protein levels in the cerebrospinal fluid (CSF) [35]. TNF and IFN-γ have been shown to act synergistically to increase apoptosis in human oligodendrocytes in culture [48] and to upregulate TNFR1 expression, thereby rendering oligodendrocytes responsive to TNF [1]. These data imply a direct role for meningeal immune infiltrates in releasing relevant inflammatory mediators that may diffuse through the adjacent cerebral cortex to, directly and/or indirectly, mediate demyelination and neurodegeneration [37].

Whereas soluble TNF (sTNF) signals predominantly via TNFR1 to promote pro-inflammatory reactions, transmembrane TNF (tmTNF) signals via both TNFR1 and TNFR2 to activate protective and homeostatic functions. Aberrant TNF production plays a role in the pathogenetic mechanisms of many autoimmune and chronic inflammatory conditions, including rheumatoid arthritis, Crohn’s disease, psoriasis, systemic lupus erythematosus, type II diabetes and atherosclerosis [28, 49]. Chronic overexpression of TNF by astrocytes in mice causes CNS inflammation, oligodendrocyte apoptosis, demyelination and neurological dysfunction [30], even in the absence of mature lymphocytes. Selectively blocking the effects of soluble TNF by treating EAE mice with a dominant negative TNF monomer improved clinical outcome by reducing the production of pro-inflammatory cytokines and chemokines, whilst leaving tmTNF free to signal via TNFR2 to promote repair and neuroprotection [4, 9, 57]. Thus, there is increasing evidence that TNF plays a major role in the pathogenesis of MS via TNFR1 signalling.

In order to identify some of the signalling pathways that may be involved in the increase in cortical pathology in response to an increased inflammatory milieu in the subarachnoid space, we have carried out a gene expression profiling study on subpial cortical GM lesions and nearby normal appearing GM from the motor cortex of MS cases with substantial meningeal infiltration, compared with cases with only mild meningeal inflammation and non-neurological controls. In addition to highlighting a number of the key molecular mechanisms of cortical injury in progressive MS, our results suggest that the degree of meningeal inflammation affects the balance between TNFR1 pro-cell death and TNFR1/TNFR2 pro-cell survival signalling, which then determines the severity of the pathology.

## Materials and methods

### Post-mortem MS and control tissues

All post-mortem tissues were obtained from the UK MS Society Tissue Bank at Imperial College and were obtained at autopsy with fully informed consent under ethical approval by the National Research Ethics Committee (08/MRE09/31), with the exception of 6 controls kindly provided by Dr. Isidro Ferrer (Servicio Anatomia Patologica, Hospital Belvitge, Barcelona, Spain). The demographic data and clinical and neuropathological features of the SPMS cases and controls are shown in Table 1. The clinical diagnosis of MS was confirmed based on the patient history (summarised by RN) and a detailed neuropathological analysis (provided by FR) as described previously [51].

**Table 1:** Individual clinical, post-mortem and neuropathology details of the examined MS cases

Cases	Sex/age of death (years)	Post-mortem delay (hours)	Age of onset (years)	Disease duration (years)
Controls				
C14	f/64	18		
C25	m/35	22		
C28	f/60	13		
C41	m/51	22		
A05-58	m/54	3		
A05-149	m/55	5		
A06-65	m/59	7		
A06-189	f/47	9		
A07-9	m/59	6		
A07-67	m/47	4		
Median	3f-7 m/54.5	8		
Follicle-negative SPMS				
MS003	m/55	44	34	21
MS042	m/51	8	29	22
MS056	m/63	11	24	39
MS074	f/64	7	28	36
MS100	m/46	7	38	8
MS104	m/53	12	42	11
MS114	f/53	12	37	16
MS127	m/51	21	28	23
MS163	f/45	28	39	6
MS200	m/43	20	24	19
Median	3f-7 m/52	12	31.5	20
Follicle-positive SPMS				
MS079	f/49	7	25	24
MS092	f/27	26	20	7
MS121	f/49	24	35	14
MS136	m/40	10	28	12
MS153	f/50	12	18	32
MS154	f/35	12	23	12
MS160	f/44	18	28	16
MS176	m/37	12	10	27
MS180	f/44	9	26	18
MS234	f/39	15	24	15
Median	8f-2 m/42	12	24.5	15.5

The current study was performed on precentral gyrus (motor cortex, Additional file 1: Fig. S1A) snap frozen tissue blocks (2 × 2 × 1 cm) from 10 cases of SPMS (median post-mortem delay (PMD) = 12 h; median age at death = 42 years) previously characterised as exhibiting lymphoid-like infiltrates in the leptomeninges (follicle-positive SPMS), 10 cases of SPMS (median PMD = 12 h; median age at death = 52 years) without organised meningeal infiltrates (follicle-negative SPMS) and 10 non-neurological control cases (median PMD = 8 h; median age at death = 54 years). Clinical and neuropathology details of the examined MS and control cases are reported in Table 1. The same tissue blocks from the same MS and control cases were previously analysed in our quantitative study of neuronal and glial alterations in cortical pathology and the presence of lymphoid-like immune cell aggregates was determined as described previously [37].

### Dissection of the cortical lesions and normal appearing grey matter

For each precentral gyrus block (Additional file 1: Figure S1A), one chronic active subpial grey matter lesion (GML—type III; Additional file 1: Figure S1B) and one nearby area of normal appearing grey matter (NAGM; Additional file 1: Figure S1C) in the same tissue block were identified by MOG and MHC class II immunostaining of 10-μm serial cryosections as previously described [37]. Selection of the GML areas for dissection was determined by the presence or absence of substantial inflammatory infiltrates and tertiary lymphoid-like structures in the immediately overlying meninges of the individual block in the cases previously characterised as F+ or F− SPMS [37]. Only subpial cortical lesions extending at least up to layer V were dissected (Additional file 1: Figure S1B). The outlines of the areas of interest were scored on the whole snap frozen tissue block with a scalpel blade and 50–100–μm sections carefully cut on a Leica cryostat. The dissection with the scalpel was limited to layer I to VI of the cortex and did not include WM or meninges. Approximately 50–150 mg of tissue was collected for each area. Fifty milligrams of tissue yielded approximately 25 μg of total RNA, which was sufficient for later analysis.

### RNA extraction and quality assurance

RNA extractions were performed using the RNeasy Lipid Tissue Midi Kit (Qiagen), which was designed for optimal lysis of tissue rich in lipids, following procedures previously optimised for use with human tissues [17]. The RNA concentration and quality were determined using a Nanodrop 2000 spectrophotometer and Agilent 2100 Bioanalyser respectively. Only RNA samples of excellent quality and integrity (RIN > 7) were used for gene expression and real-time RT-PCR analysis. The mean RIN values (± SEM) for each of the examined group of samples were 8.23 (1.88) for controls; 8.33 (0.72) for F+ GML; 7.79 (1.02) for F− GML; 8.36 (0.52) for F+ NAGM; 7.69 (1.09) for F− NAGM.

### Microarray hybridisation and scanning

Hybridisation of the RNA samples onto the Illumina whole genome HumanRef8 v2 BeadChip arrays was conducted at the Genome Centre at Queen Mary College, University of London, following the procedures previously optimised [16]. All the labelling and hybridisation of the samples (*n* = 60, including technical replicates and human reference RNAs) were carried out in a single experiment to reduce the technical variability. RNA samples were prepared using the TotalPrep-96 RNA amplification kit (Ambion/Applied Biosystem, Warrington, UK) following the manufacturer’s instructions. First- and second-strand cDNA (dsDNA) was synthesised from 0.5 μg of total RNA and then purified. Biotin-labelled cRNA was synthesised from dsDNA and then was captured using RNA binding beads, washed twice and stored at − 20 °C. The concentration and quality of the cRNA were checked using ND1000 Nanodrop and Agilent 2100 Bioanalyser respectively. The samples were applied to the arrays and assembled into the BeadChip Hybr Chamber and hybridisation was carried out using the Illumina whole genome gene expression direct assay system at 58 °C overnight. After washes, the signal was developed with Streptavidin-Cy3 and the BeadChips were scanned using the Illumina BeadArray Reader.

### Data analysis, normalisation and pathway analysis

The data from the Illumina BeadArray Reader were extracted using the BeadStudio 3.2 software package (Illumina). The lists of data obtained using BeadStudio 3.2 were converted for data normalisation using the Rosetta Resolver ® system software (Rosetta Biosoftware) [63] and analysed for gene differential expression analysis using the BRB-ArrayTools, developed by the Biometric Research Branch of the Division of Cancer Treatment & Diagnosis of the National Cancer Institute (http://linus.nci.nih.gov/BRB-ArrayTools.html). Univariate principal component analysis of the data obtained from the Illumina arrays was conducted to detect arrays of low quality using Rosetta Resolver software. No technical outliers were detected out of the 50 arrays. We identified genes that were differentially expressed between each pathological condition and/or the control group using a random variance *t* test. Several different stringency conditions were tested in a permutation-based false discovery rate (FDR) assessment performed, as previously described [19], to determine the threshold *p* value and fold change providing the optimal balance of true vs false discovery. Briefly, microarrays were divided in two groups containing the same number of experimental (MS) and control samples, randomly assigned to one of the two groups. Ten different sample permutations underwent class comparison analysis, and the median number of differentially expressed genes obtained from all the iterations was compared with the numbers obtained from the real experimental dataset to determine the overall FDR in our dataset. According to the results of this test, genes were considered statistically significant at *p* < 0.01 and fold change ± 1.5 (Additional file 1: Figure S2). Average linkage hierarchical cluster analysis using Pearson correlation with uncentered metrics was performed using gene cluster and data were visualised by Treeview. Pathway analysis was performed by means of the Gene Set Expression Comparison Tool available in BRB Tools, consisting of a two-sample *t* test (with random variance model) performed among control and MS samples on the basis of 300 BioCarta Pathways gene sets. Tests used to find significant gene sets were the LS/KS permutation test, identifying gene sets which have more genes differentially expressed among the phenotype classes than expected by chance, and Efron-Tibshirani’s GSA maxmean test, to identify differentially expressed gene sets. The threshold of determining significant gene sets was 0.05, and only when at least 2 out the 3 statistical tests confirmed significance the pathway was considered as valid.

### Real-time PCR verification

To confirm individual gene microarray results, the same RNA samples used on the Illumina microarrays were also used for quantitative real-time RT-PCR, using both the QuantiTech® reverse transcription kit, the QuantiTech® SYBR Green kit in combination with the QuantiTech® primer assays (Qiagen) or TaqMan MGB probe assays (Applied Biosystems) using respectively the Mx3000P™ real-time PCR system (Stratagene, La Jolla, USA) or the 7500 Fast Real-Time PCR System (Applied Biosystems) according to the procedures previously optimised [16, 24]. For all RT-PCR assays, the expression levels of target genes were normalised to the levels of the *GAPDH* housekeeping gene, which represented one of the most stable housekeeping genes in the complete dataset [15]. Quantitative analysis was performed evaluating the ‘delta-Ct’ value (the factor 2 delta-Ct is used to express the ratio between the gene of interest and the internal reference gene).

### Protein extract preparation and assay

Total protein extracts were prepared from sections, cut as described above, from other GML areas from the same cases used for the gene expression microarray, and analysed using the Human TNF-RI and TNF-RII Ultra-Sensitive kits from MesoScale Discovery (MSD, Maryland, USA), according to the manufacturer’s instructions.

### Immunohistochemistry/immunofluorescence

Serial sections from the same snap frozen tissue blocks used for RNA extraction were used for the immunohistochemical analysis. Air-dried, 10-μm-thick cryosections were rehydrated with PBS and immunostained with the monoclonal or polyclonal antibodies listed in Additional file 2: Table S1, following the immunohistochemistry and/or immunofluorescence procedures previously described in detail [36]. Antibody binding was visualised using peroxidase or alkaline phosphatase systems (Vector Labs, Peterborough, UK) and with Alexa 488-, Cy3-, rhodamine- or fluorescein-conjugated secondary antibodies when using immunofluorescence.

### Statistical analysis

The following software packages were used: GraphPad Prism 7 (GraphPad Software Inc, La Jolla, CA, USA) and Microsoft® Office Excel® 2007 (Microsoft UK Headquarters, Reading, UK). Statistical analysis of the data was performed using *t* test and the non-parametric Mann-Whitney test, and differences were considered statistically significant if the *p* value was < 0.05.

## Results

### Global microarray datasets

**Fig. 1:**
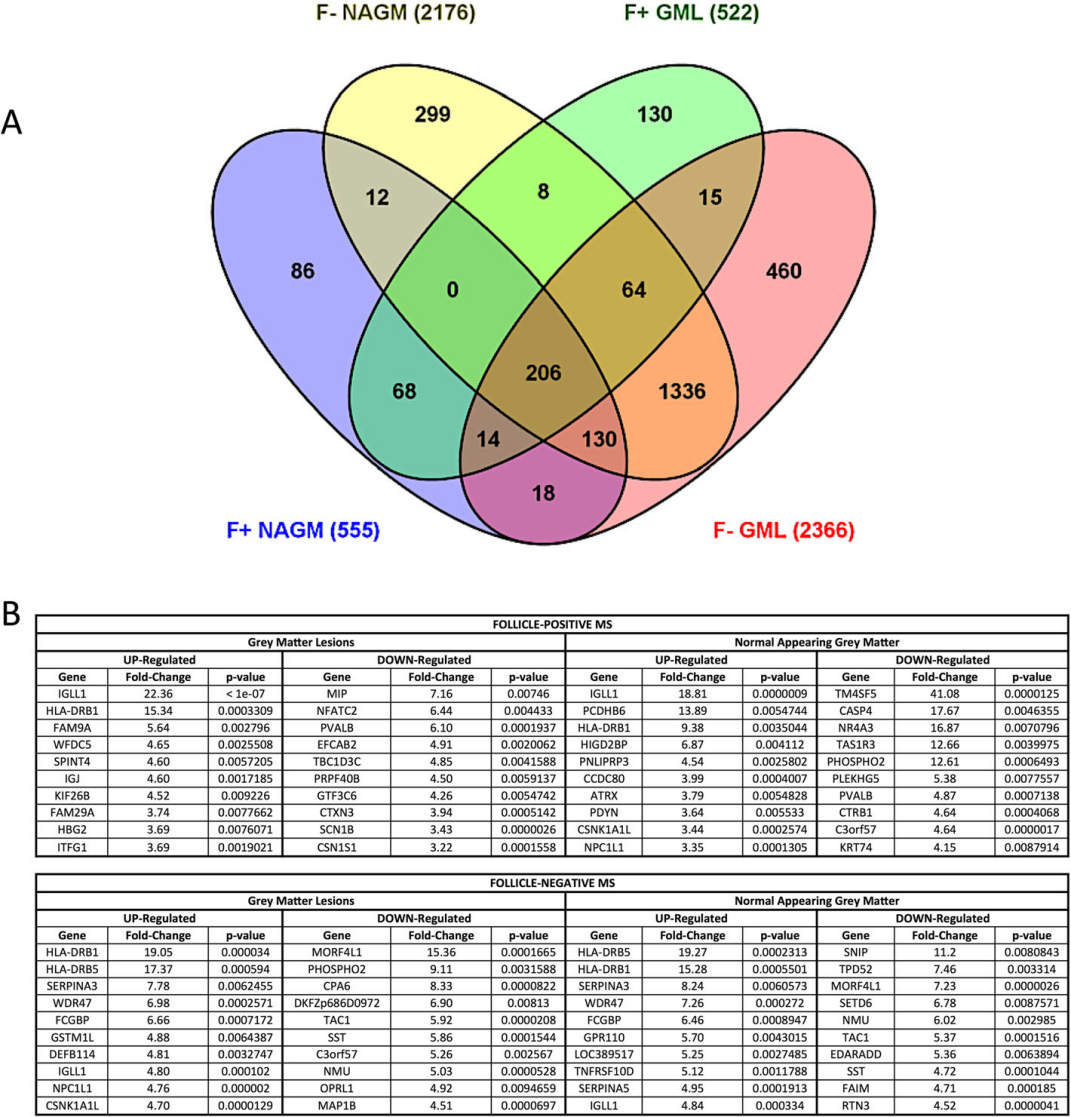
**a** Venn diagram illustrating the relations among the genes differentially expressed in the precentral gyrus in control healthy donor tissue compared with grey matter lesion (GML) and normal appearing grey matter (NAGM) of both F+ SPMS and of F− SPMS cases. Each oval represents the list of genes differentially expressed between one condition and control group (*p* < 0.05 fold change > 1.5). Numbers in each overlapped area indicate the numbers of differently expression genes shared by two or more conditions. Numbers in non-overlapping portion of each oval show the number of transcripts uniquely deregulated in one condition. The brown intersection in the middle represents genes which are significantly differentially expressed in all different conditions vs controls consistently. **b** Table reporting the list of the ten most regulated upregulated and downregulated genes in each examined condition: grey matter lesion and normal appearing grey matter in follicle-positive or follicle-negative MS cases respect to healthy controls. For each listed gene, the fold change of gene expression respect to controls and the *p* value of the univariate test is expressed

Using a class comparison tool (*p* < 0.01 fold change > 1.5 vs control group), all possible comparisons between each group of patients and tissues were made, providing information on the total number of deregulated and unchanged genes in each MS group compared with the controls (Fig. 1a; Additional file 1: Figure S2A; Additional file 2: Table S2). A total of 4658 transcripts showed a significant alteration in any of the groups vs control samples from healthy subjects. A permutation-based false discovery rate (FDR) assessment estimated that these stringency conditions generated, in our dataset, a false discovery rate of 5.7% (Additional file 1: Figure S2B, C), thus indicating that approximately 94% of the observed alterations reflected true biological differences between samples and not casual variations. The complete data files are available at Gene Expression Omnibus (accession number GSE135511).

Analysis of all individual gene changes in the different MS groups compared with controls provided a general picture of the transcriptional modulations specifically observed in one group or shared among different groups. A higher number of deregulated genes was found in F− SPMS cases (GML—2366 genes (1148 up, 1218 down) and NAGM—2176 genes (1058 up, 1118 down)) when compared with those deregulated in the F+ SPMS group (GML—522 genes (231 up, 291 down) and NAGM—555 genes (194 up, 361 down)) (Fig. 1a). This difference between the F+ SPMS and F− SPMS cases is also clearly seen in the heatmap of the total gene expression profiles (Additional file 1: Figure S2A). Of the genes that were significantly altered in GML, 130 genes were found only in F+ GML, 460 only in F− GML and only 15 genes were changed in the GMLs of both F+ and F− SPMS cases (Fig. 1a). In the NAWM, 86 genes were found significantly deregulated only in F+ NAGM, 299 only in F− NAGM, whilst only 12 genes changed in the NAGM of both F+ and F− cases (Fig. 1a).

### Analysis at an individual gene level

Of the 10 most upregulated genes in each group (Fig. 1b), the immunoglobulin genes (IGLL1) and MHC class II genes (HLA-DRB1) were upregulated to the greatest extent in all groups. As expected, the immunoglobulin genes were upregulated to a greater extent in F+ SPMS cases and to a similar extent in GML compared with NAGM tissue. Of the other 10 most upregulated genes, there was a significant representation of various proteases (e.g. SERPIN A3/A5). Of the 10 most downregulated genes in each group (Fig. 1b), the greatest representation was of proteins involved in neuronal function (PVALB, SCN1B, CTXN3, TAC1, NMU, SST, MAP1B), in particular neuropeptide synthesis.

In agreement with our previous data showing significant neuronal loss in all cortical layers [37], among the genes with the greatest decrease in expression in the MS group compared with controls were the neuronal genes for parvalbumin (PVALB), glutamate decarboxylase 1 (GAD-1), NMDA glutamate receptor subunit 2A (GRIN2A), the large, medium and low molecular weight neurofilament proteins (NEFL, NEFM NEFH), microtubule-associated protein 1B (MAP1B), voltage-gated sodium channels sub-units (SCN1B) and synaptobrevin (VAMP1), in addition to downregulation of other neurotransmitter receptors, ion channels and growth factors, such as FGF14 (table in Additional file 1: Figure S3A). Most of the gene expression changes associated with neuronal/axonal alterations, such as PARV, NEFL, NEFM and NEFH, were also verified by real-time RT-PCR and were found to be greater in F+ SPMS compared with F− SPMS cases and controls (Additional file 1: Figure S3B-E). Upregulation of specific markers of microglial activity, such as CD68 and inducible nitric oxide synthase (iNOS), was also further validated by real-time RT-PCR (Additional file 1: Figure S3F, G).

### Gene ontology pathway analysis

**Fig. 2:**
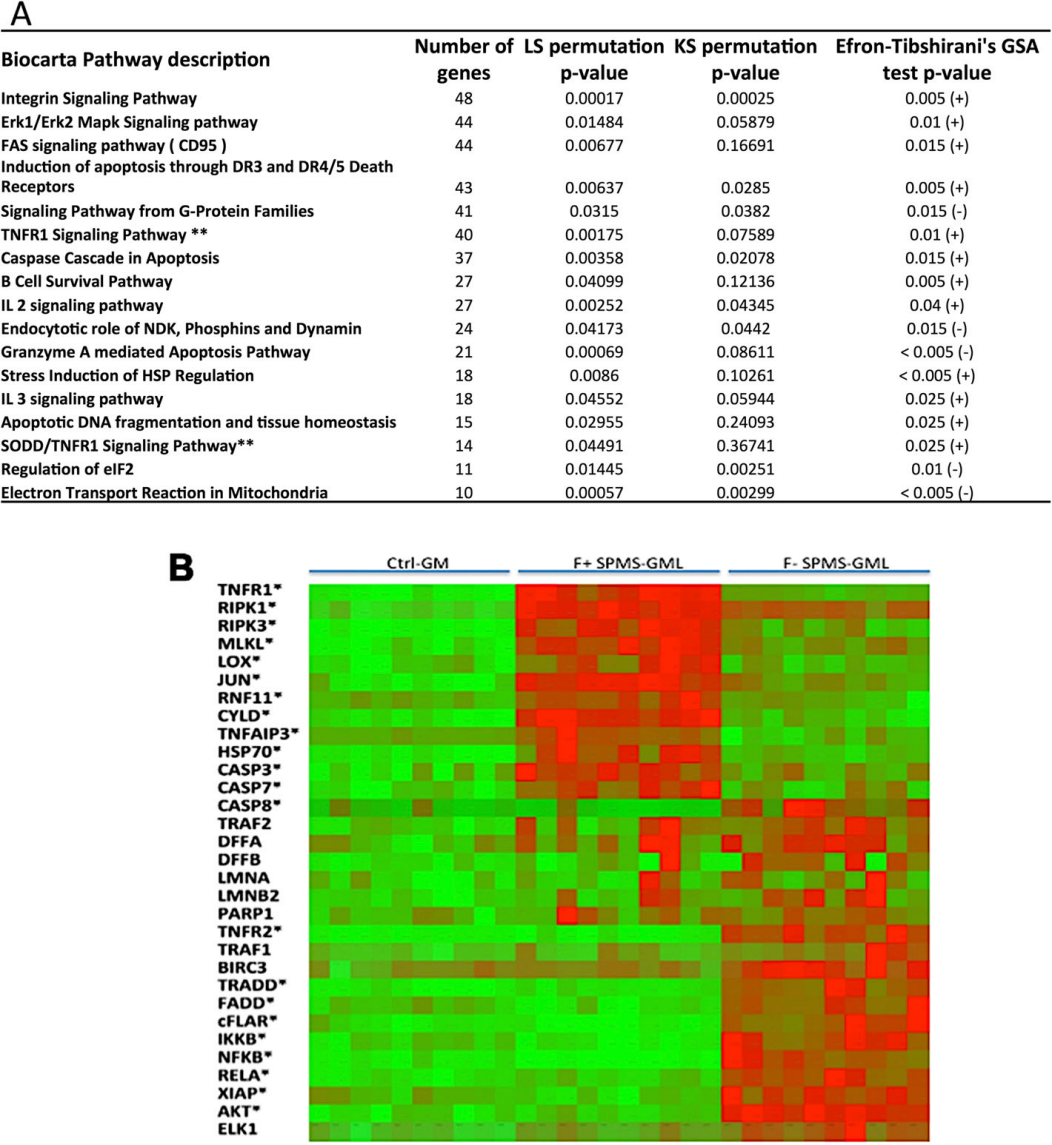
**a** Gene set enrichment analysis comparing controls vs MS samples at the Biocarta pathway level. The table reports the 17 pathways showing the strongest modulation, selected for having a significant *p* value in at least 2 of the 3 different statistical approaches utilised (LS/KS permutation and Efron-Tibshirani’s GSA test). **b** Heatmap showing the level of expression of TNF-related genes clustered into two distinct groups of genes, pro-cell death and pro-survival signalling, preferentially expressed in the cortical lesions (GML) of follicle-positive secondary progressive MS case (F+ SPMS) and of follicle-negative secondary progressive MS case (F− SPMS) respect to the cortex in healthy controls. Asterisks define the gene validated by real-time RT-PCR

In order to identify common biological functions attributed to the deregulated genes, we conducted a gene ontology analysis at the BioCarta pathway level. When controls were compared with all MS samples, we found numerous gene-enriched pathways significantly deregulated in the MS cases (*p* < 0.05 for LS/KS permutation and Efron-Tibshirani’s GSA maxmean test), many of which were associated with inflammatory responses and cell death/cell survival signalling. Out of the 17 most significantly deregulated gene pathways in the MS cortical grey matter (shown in Fig. 2a), 2 are directly involved in TNFR1/death receptor signalling and 8 are involved in cell death/cell survival signalling (Fig. 2a). The complete lists of deregulated pathways are available as Additional file 2: Tables S3–S5.

When F+ SPMS cases were compared with F− SPMS cases, we found 16 gene-enriched sets out of 63 differentially regulated in the F+ cases (Additional file 1: Table S4). Interestingly, dysregulation of gene pathways involved in pro-inflammatory and cell death signalling, such as TNFR1, TRAILR, IL-17, IL-3 and IL-4 signalling, was observed. When the GML was compared with NAGM for all the examined MS cases, we found only 12 (out of 42 detected) gene-enriched pathways significantly deregulated in the GMLs. Two of these functional groups were related to cytokine expression (Additional file 2: Table S4) and included TNF, IFN alpha 1, IFN beta 1, IL-17A, IL-4, IL-10 and IL-2.

### TNF signalling pathways

Because the pathway analysis of all MS cases vs controls indicated an overrepresentation of pathways involving TNF/TNF receptor interaction and its many downstream signalling pathways (Fig. 2a), including cell death signalling, we decided to further analyse the expression of genes involved in TNF/TNFR1 and TNF/TNFR2 interactions, in preference to other significantly deregulated pathways. The data clearly indicated that when the genes involved in TNF signalling were segregated according to TNFR1/RIP1-mediated cell death pathways vs TNFR1/TNFR2/NFkB-mediated cell survival signalling (Fig. 2b), there was a clear difference between the F+ SPMS and F− SPMS cases. Whereas F+ SPMS cases showed enrichment for upregulated genes involved in TNFR1 cell death signalling, F− SPMS cases showed enrichment for TNFR1/TNFR2 cell survival signalling. Therefore, we further investigated differential expression of individual genes in these pathways.

### TNF receptor expression in the motor cortex of secondary progressive MS cases

**Fig. 3:**
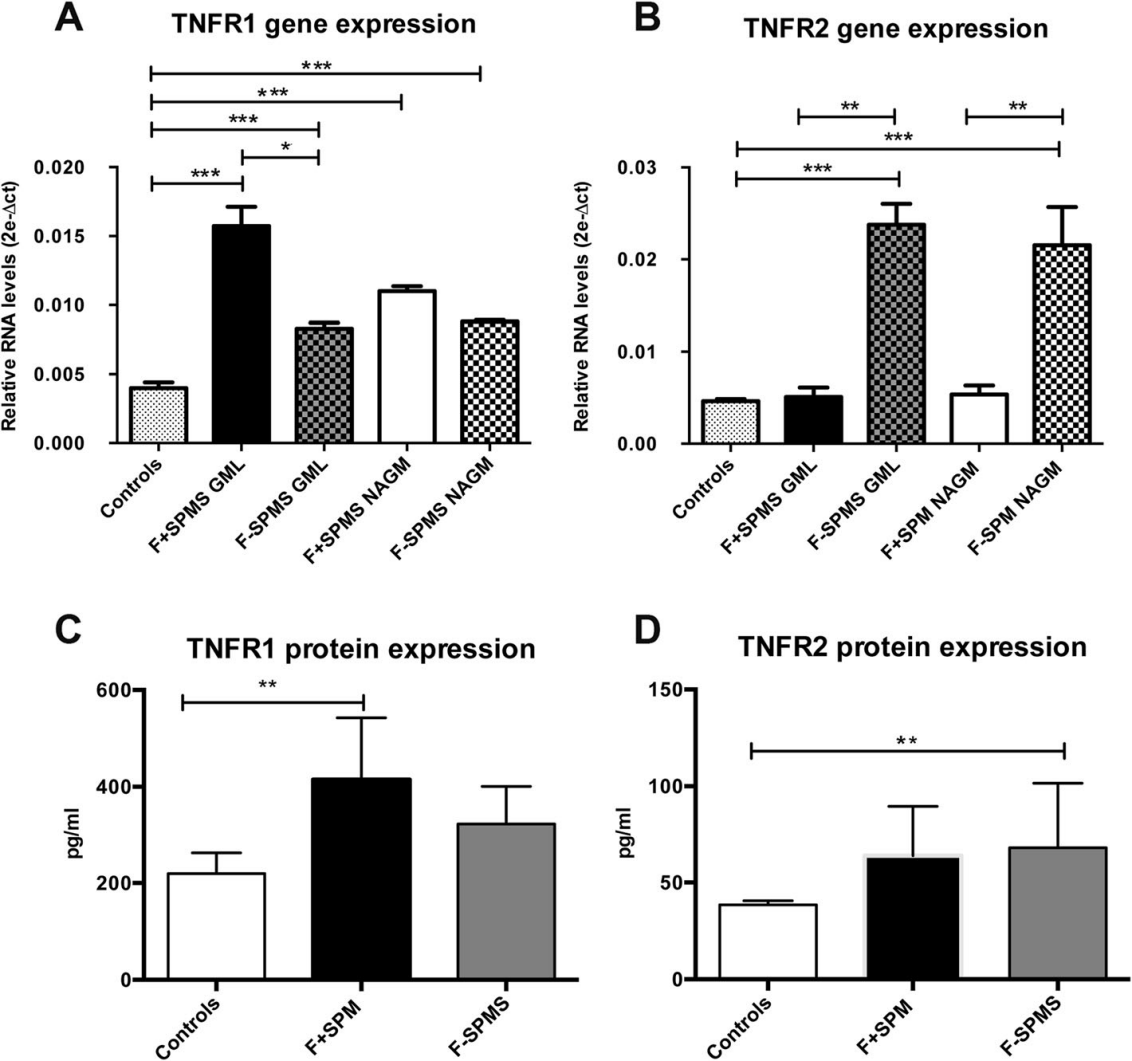
Real-time RT-PCR gene expression of TNFR1 (**a**) and TNFR2 (**b**) in the precentral gyrus, both grey matter lesion (GML) and normal appearing grey matter (NAGM), of 10 F+ and 10 F− SPMS cases compared with 10 controls (***p < 0.001, **p < 0.01, *p < 0.05). Protein expression of TNFR1 (**c**) and TNFR2 (**d**) in precentral gyrus, both grey matter lesion (GML) of 10 F+ and 10 F− SPMS cases compared with 5 controls (**p < 0.01, *p < 0.05). For each statistical comparison, the *p* value, obtained by non-parametric Mann-Whitney test, has been reported

Among the TNF-related genes differentially deregulated in F+ and F− SPMS cases compared with controls, significant and different changes were seen for the two TNF receptors. In order to verify these data, TNFR1 and TNFR2 gene expression was analysed using qRT-PCR. This confirmed significant increases in TNFR1 gene expression in GML (4.50-fold, *p* < 0.001) and NAGM (2.53-fold, *p* < 0.001) of F+ SPMS cases and smaller increases in GML (3.17-fold, *p* < 0.001) and NAGM (2.30-fold, *p* < 0.001) of F− SPMS cases (Fig. 3a). Moreover, the expression of TNFR1 was 1.78-fold higher (*p* < 0.05) in GML of F+ SPMS compared with GML of F− SPMS cases. In contrast, significant increases in TNFR2 gene expression were detected only in the GML (fold change = 4.61, *p* < 0.001) and NAGM (fold change = 4.03, *p* < 0.001) of F− SPMS cases, but not in F+ SPMS cases (Fig. 3b). Electrochemiluminescence-based protein analysis (MesoScale Discovery V-Plex plates) on extracts from dissected chronic active subpial GM lesions from 10 F+ SPMS, 10 F− SPMS and normal GM from 5 controls demonstrated a significant increase in TNFR1 protein expression (1.90-fold, p ≤ 0.01) only in F+ SPMS cases with respect to controls (Fig. 3c), whereas significantly increased TNFR2 protein expression (1.80-fold, *p* ≤ 0.01) was present only in F− SPMS cases with respect to controls (Fig. 3d). However, we did not find a significant difference between the F+ SPMS and F− SPMS cases at the protein level for either receptor.

**Fig. 4:**
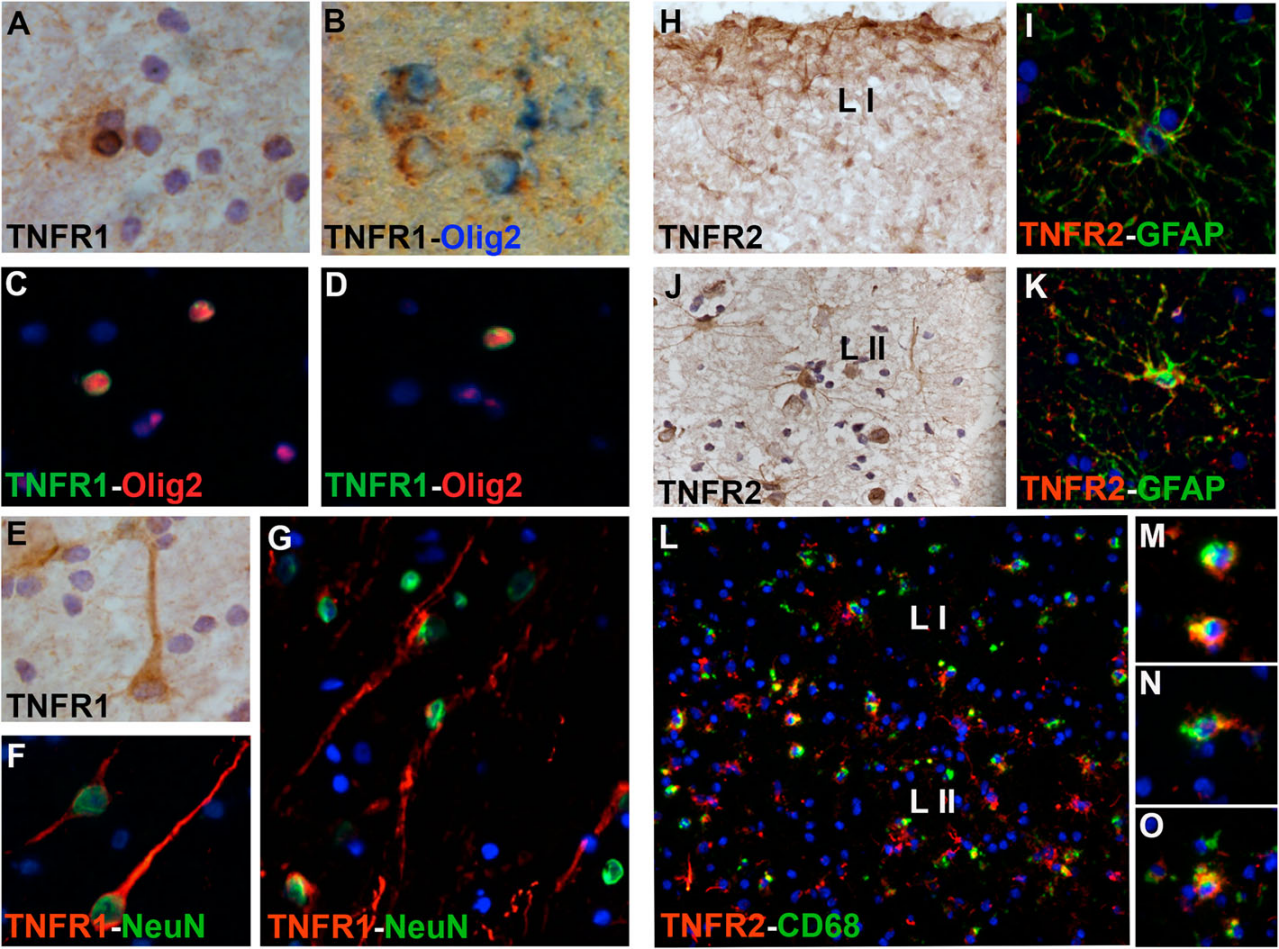
Localisation of TNFR expression in the precentral gyrus of SPMS cases. TNFR1 was found expressed by cells with morphology resembling oligodendrocytes (**a**) as validated by double immunohistochemistry (**b**) and double immunofluorescence (**c**, **d**) with the olig2 oligodendrocyte marker. Furthermore, TNFR1 was found expressed by cells with neuronal morphology (**e**) as validated by double immunofluorescence (**f**, **g**) with NeuN marker of neuronal nuclei. TNFR2 was found expressed by cells with astrocyte morphology (**h**, **j**), in particular in the external cortical layer I (**h**) and II (**j**). Double immunofluorescence (**i**, **k**) with GFAP-specific astrocyte marker confirmed that TNFR2 was mainly expressed by cortical astrocytes. TNFR2 was found expressed also by rare activated microglia/macrophages CD68+ cells (**l**–**o**) in the external cortical layers of grey matter lesions. Original magnifications: × 100 (**l**), × 200 (**g**, **h**, **j**); × 400 (**a**–**f**, **i**, **k**, **m**–**o**)

In order to better understand the cortical cell expression of the two TNF receptors, immunohistochemistry and double immunofluorescence were performed using TNFR1- and TNFR2-specific antibodies. TNFR1 was predominantly detected on cells morphologically resembling oligodendrocytes and neurons (Fig. 4a, e). Double immunofluorescence demonstrated that TNFR1 was predominantly expressed by Olig2+ oligodendrocytes (Fig. 4b–d), in particular in the most inner cortical layers V and VI, close to the WM, and by small numbers of NeuN+ neurons (Fig. 4f, g) in the most external cortical layers I–III. TNFR2 was predominantly detected in the most external cortical layers I and II on cells with ramified astrocyte-like morphology, in particular in layer I in close association with the pial membrane (Fig. 4h), or in adjacent layer II (Fig. 4 j). Double immunofluorescence confirmed that TNFR2 was expressed by GFAP+ astrocytes (Fig. 4i, k), both on the cell body and processes. TNFR2 was also detected on CD68+ microglia close to the pial surface and in the most external cortical layers I–II (Fig. 4l), occasionally with a macrophage-like morphology (Fig. 4m–o).

### Increased meningeal inflammation is associated with a change in the balance of TNF signalling

**Fig. 5:**
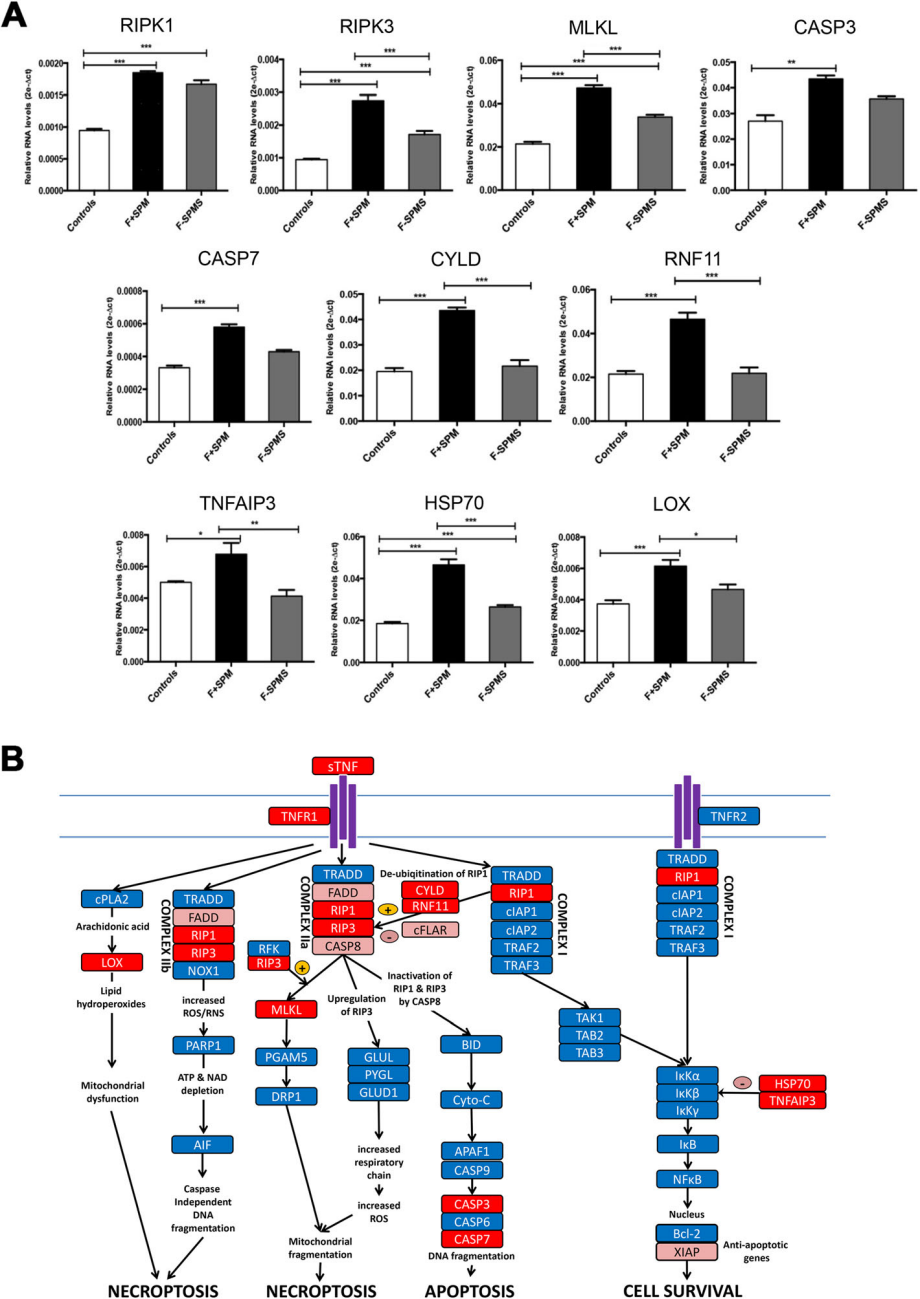
**a** Real-time RT-PCR expression of genes related to TNF/TNFR1 pathway overexpressed in the precentral gyrus grey matter lesions (GML) of 10 F+ SPMS cases compared with both controls and to 10 F− SPMS cases (***p < 0.001, **p < 0.01, *p < 0.05). For each statistical comparison, the *p* value, obtained by non-parametric Mann-Whitney test, has been reported. **b** Schematic diagram of molecular changes involved in TNF/TNFR1 stimulated necroptotic/apoptotic signalling significantly upregulated in particular in the F+ SPMS cortex (red boxes). Pink boxes represent genes that were downregulated in F+ SPMS cases. Changes in both GML and NAGM in each group have been pooled

**Fig. 6:**
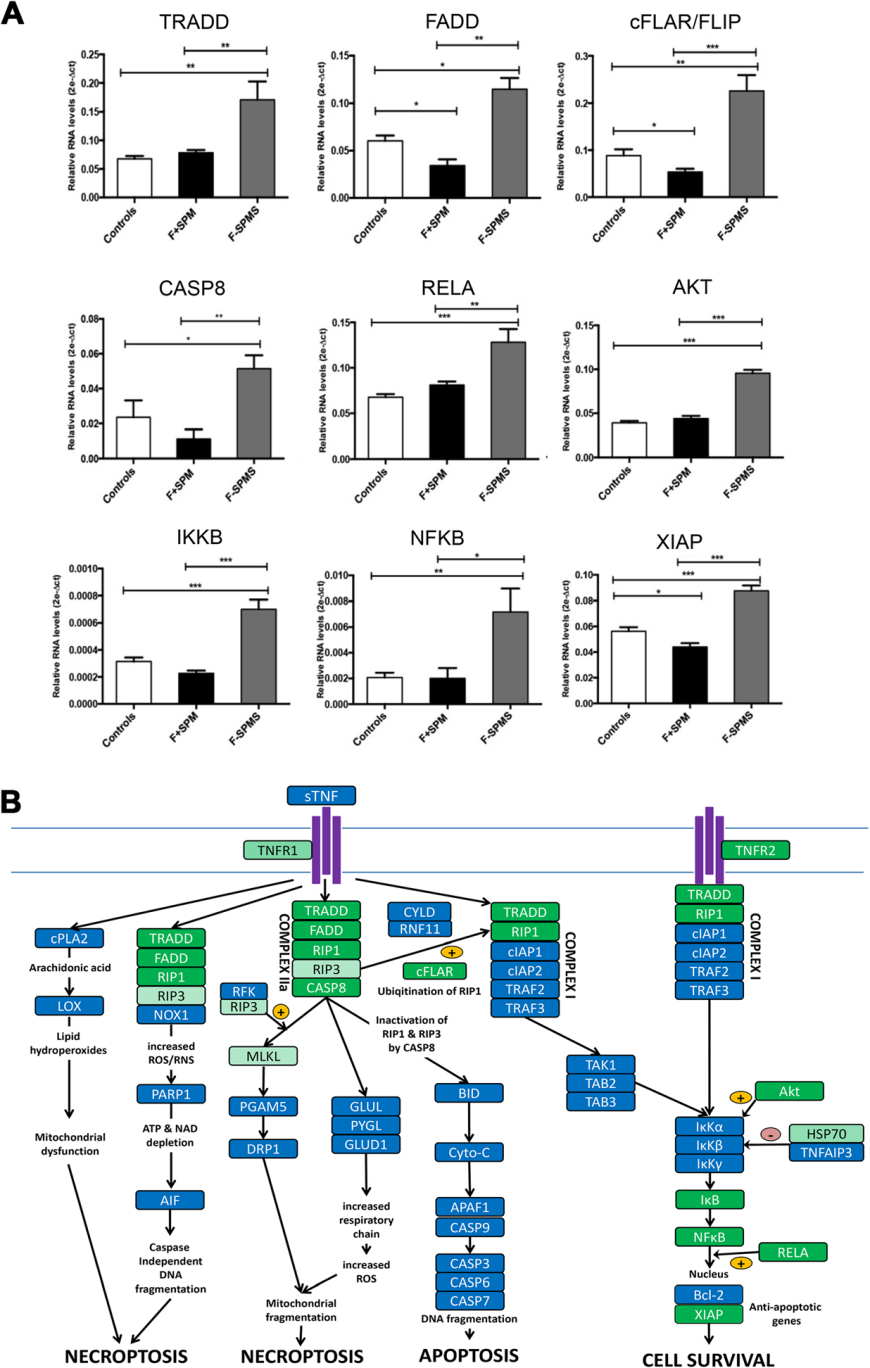
**a** Real-time RT-PCR expression of genes related to TNF/TNFR2 pathway overexpressed in the precentral gyrus grey matter lesion (GML) of 10 F− SPMS cases compared with both controls and 10 F+ SPMS cases. For each statistical comparison, the *p* value, obtained by non-parametric Mann-Whitney test, has been reported (***p < 0.001, **p < 0.01, *p < 0.05). **b** Schematic diagram of molecular changes involved in TNF/TNFR2 stimulated cell survival signalling significantly upregulated in the F− SPMS cortex (dark green boxes). Light green boxes represent genes that were upregulated in F− SPMS cases but to a lesser extent than in F+ SPMS cases. Changes in both GML and NAGM in each group have been pooled

In order to verify changes to the different TNF signalling pathways in GMLs in F+ SPMS compared with F− SPMS cases seen in the microarray analysis, we carried out RT-PCR for key genes in these pathways. Although gene expression for a number of different caspases (CASP3, CASP7) involved in the apoptotic cascade was upregulated in F+ SPMS by 60% and 66% compared with controls (Fig. 5a, b), the key regulator gene, CASP8, was non-significantly decreased by 50% (Fig. 6a), suggesting that there was not an increase in apoptotic signalling in the F+ SPMS GMLs. However, a number of genes involved in TNF/TNFR1-stimulated necroptotic signalling were significantly upregulated in the F+ SPMS cortex, including the key kinases RIPK1 (88%), RIPK3 (160%) and MLKL (135%) (Fig. 5a), which together upon phosphorylation induce necroptosis in conditions of caspase 8 and FADD deficiency via a number of different pathways. RIPK1 ubiquitinylation inhibits the necroptotic pathway, but upregulation of the RNF11 (121%) and CYLD (126%) genes only in the F+ SPMS cortex would act to de-ubiquitinylate RIPK1 and thereby promote necroptosis (Fig. 5a). The LOX (lipid hydroperoxidase) pathway involved in oxidative stress-mediated mitochondrial dysfunction and consequent necroptosis [14] was also found significantly upregulated (57%) only in F+ SPMS cases. Increased expression of the HSP70 (151%) and TNFAIP3 (55%) genes, which would inhibit activation of NFkB [14, 27], was found only in the GML of F+ SPMS cases (Fig. 5a). The combined results suggest that TNF/TNFR1-stimulated necroptotic signalling is upregulated in the MS cortex in conditions of increased meningeal inflammation (Fig. 5b).

In contrast to F+ SPMS cases, in F− SPMS cases, there was a significant upregulation of genes involved in TNFR1/TNFR2-mediated signalling via NFkB (Fig. 6a, b). Increased gene expression of RIPK1 (70%), FADD (96%), TRADD (173%) and cFLAR (130% increase) was seen in the F− SPMS GMLs (Fig. 6a), which would direct signalling towards NFkB activation. Increased expression of IKκB (98%) and NFkB (250%), together with increased expression of genes for anti-apoptotic molecules and survival factors, such as cFLAR (125%), RELA (85%), AKT (125%) and XIAP (47%), was detected only in F− SPMS and not in F+ SPMS (Fig. 6a). The increased gene expression of CASP8 in the F− SPMS cortex would also inhibit necroptotic signalling (Fig. 6a). Collectively, these results would suggest that TNF signalling via both TNFR1 and TNFR2 is directed more towards increasing cell survival in F− SPMS (Fig. 6b).

## Discussion

Chronic inflammation that is sequestered within the CNS is suggested to be one of the main drivers of the accumulation of neurological deficit during the progressive stages of MS [5, 13] and is manifest in the cerebral cortical grey matter as a build-up of meningeal immune cell infiltrates [29, 36], subpial demyelination [3, 31, 36] and neuronal and axonal damage [8, 25, 37, 43, 47, 62]. We show here, using gene expression profiling of demyelinated and normal appearing grey matter from the motor cortex of secondary progressive MS cases with high and low levels of meningeal immune cell infiltrates, that at the RNA transcript level, there is a change in the balance of TNF signalling pathways from TNFR1/2-activated NFκB-dependent cell survival towards TNFR1 activated RIPK3 dependent necroptotic cell death with increasing levels and organisation of meningeal infiltration. Such an altered balance, varying in its extent between different MS cases, may help explain the heterogeneity seen in the degree of cortical pathology and its contribution to disease progression.

Previous studies of gene expression changes in MS cortical grey matter have studied MS cases that have not been stratified in any way and have considered relatively small numbers [19, 58]. When the present data from the study of 20 SPMS cases are considered without any prior stratification, the results are largely in agreement with previous studies and identify changes to individual genes and gene networks involved in activated microglial function, inflammatory processes, oxidative stress [20] immunoglobulin synthesis [58], neuronal damage and mitochondrial dysfunction [19]. The finding of Ig-related genes as one of the group of genes with the highest level of upregulation is consistent with a previous finding [58] and may be explained not only by the presence of contaminating RNA from the adjacent meninges containing B cells and plasma cells but also by the increased frequency of perivascular B cell infiltrates previously identified in cortical grey matter from MS cases with increased meningeal inflammation [38]. Previous studies have also highlighted the presence of neuronal and axonal degeneration in the NAGM [19] and have suggested their role in the accumulation of irreversible disability in progressive MS. Our finding of significant decreases in gene expression for multiple neuronal and synaptic proteins confirms and extends this to show that similar changes occur in both GMLs and NAGM, suggesting that the mechanisms leading to neuronal degeneration may be largely independent of demyelination.

Although previous studies have illuminated a number of downstream pathogenetic mechanisms involved in cortical damage [18–20, 26], they have not been able to identify possible initial inflammatory and/or neurodegenerative triggers for the subsequent cascade of pathological events [5, 12]. In light of our previous findings suggesting a link between meningeal inflammation and increased demyelinating and neurodegenerative pathology, we have specifically sought to understand the molecular mechanisms by which increasing cortical pathology occurs by stratifying well-characterised MS cases into those with high levels of meningeal infiltrates with lymphoid-like tissue formation and those with lower levels of diffuse meningeal infiltrates. The presence of lymphoid-like structures in the meninges of a substantial proportion of cases with secondary progressive MS (SPMS), that associates with more extensive subpial cortical damage, early disease onset and rapid clinical progression [29, 37, 51], suggests that diffusion of cytotoxic and myelinotoxic factors from the inflamed meninges across the compromised glia limitans might have a major role in causing injury in the adjacent cortical GM. Furthermore, increased levels of gene and protein expression for TNF and IFN-γ are found when increased meningeal infiltration is seen [35]. Therefore, it is not unexpected that there would be an upregulation of TNF signalling pathways in the MS cortical grey matter.

Substantial evidence exists for a role for TNF in the pathogenesis of MS [28, 52], both in the relapsing-remitting stage that is driven by the peripheral immune response, and also in the progressive stage when a compartmentalised inflammatory response may predominate. Our finding that an unbiased pathway analysis of cortical grey matter tissue revealed changes to multiple pathways and processes involving TNF/TNFR interaction extends this evidence to include a role in the pathogenesis of cortical injury. The finding of significant changes to TNF/TNFR signalling pathways at the bulk transcript level when comparing all MS cases to controls, which then were shown to vary according to the level of meningeal inflammation, provides further confirmation of a major role for TNF in the genesis of cortical pathology in MS. Local delivery of TNF into the CNS by intraventricular injection exacerbates EAE clinical disease [56] and local transgenic production in the CNS by astrocytes results in an inflammatory demyelinating pathology [30, 50]. The different responses to binding of sTNF and tmTNF to TNFR1 and TNFR2 in the inflamed CNS have recently been elucidated in EAE mice [4, 34, 57, 64] and demonstrate that, whereas binding of sTNF to TNFR1 results in the production of pro-inflammatory cytokines and increased pathology, binding of tmTNF to TNFR2 promotes remyelination and neuroprotection. TNFR1-mediated signalling in the absence of TNFR2 expression results in exacerbated chronic EAE disease [34]. The change in the balance of TNFR1- vs TNFR2-mediated signalling pathways at the gene expression level in the MS cortical GM in response to the degree of meningeal inflammation supports these animal studies. However, it needs to be emphasised that our data suggests that there is a change in the balance of TNFR2 to TNFR1 signalling, not a complete shift. Although we have separated the MS cases into those with organised lymphoid-like structures in the meninges (i.e. high levels of inflammation) and those without (lower levels of inflammation), in reality it is a biological continuum. Therefore, follicle-negative cases also have a variable level of cortical demyelination, neurodegeneration and diffuse meningeal infiltrates, but at a lower level than the follicle-positive cases.

Although the levels of TNFR1 and TNFR2 protein were not significantly different between F+ SPMS and F− SPMS cases, our further analysis of the downstream pathways suggests that the increased pro-inflammatory reactions in the MS meninges, indicated by increased levels in patient CSF [35], direct soluble TNF/TNFR1 interaction towards RIPK1/RIPK3/MLKL-mediated necroptosis, rather than caspase 8-dependent apoptosis or IKK/NFkB-dependent cell survival. This is in agreement with a recent study in which RIPK3-mediated necroptosis was demonstrated in oligodendrocytes in the MS brain under caspase 8-deficient conditions [42]. Similar to this study, we found that reduced CASP8 and c-FLIP gene expression was accompanied by increased expression of genes, such as CYLD and RNF11, involved in de-ubiquitination of RIPK1, that in combination with increased RIPK3 expression would direct TNF signalling towards necroptosis [14, 41, 44]. Our data adds to this finding by suggesting that this signalling/cell fate may be directed by soluble mediators originating from the inflamed meninges and may also be involved in neuronal damage in MS, although this will require confirmation via single-cell RNA analysis and further protein localisation studies. However, in keeping with this idea, the predominant localisation of TNFR1 in the MS cortical grey matter was in neurons and oligodendrocytes and a recent study of the topography of demyelination and neurodegeneration in MS highlighted the association of oxidative injury to neurons with an increased inflammatory process in the meninges [25]. The shift away from apoptosis agrees with a number of studies showing that only rarely could apoptotic neurons be observed in the MS cortical layers [37, 47]. However, definitive evidence for this change in the balance of cell death pathways in the MS cortex must await specific and reliable histological and molecular markers of necroptosis [44], which are currently not available. It is also not possible to say whether such changes in TNF signalling leading to neuronal cell death are specific to MS or may occur in other chronic CNS inflammatory conditions characterised by meningeal inflammation [20]. Tuberculous meningitis (TBM) is probably the most studied of the non-MS conditions that can give rise to a more long-term meningeal inflammation. Whilst elevated TNF levels are seen in the CSF of some TBM patients, there is no consistent pattern and the levels are not related to severity or clinical course of disease [39, 45]. No detailed molecular study of TNF signalling in brain tissue from TBM has been carried out and elevated meningeal inflammation is present for a much shorter time than is the case in MS. However, it is possible that there are effects of chronic TNF cytotoxicity in the TBM brain. Although it has been shown that subpial cortical demyelination is not a feature of TBM [20, 37], it is not known whether there is a significant loss of cortical neurons similar to that seen in MS. To date, it has not been possible to procure suitable frozen tissue samples from non-MS chronic inflammatory CNS conditions that have a similar disease duration.

When the level of inflammation was lower in the MS meninges, TNF/TNFR1 and TNF/TNFR2 signalling appeared to be directed more towards IKK/NFkB-mediated cell survival. The upregulation of CASP8, TRADD, FADD and RIPK1 in the presence of increased c-FLIP expression, as seen in the F− SPMS cortex, would be expected to inhibit both necroptotic and apoptotic signalling and lead to IKK complex formation and NFκB activation and translocation [14]. Our finding of TNFR2 expression predominantly in cortical astrocytes and microglia, in particular in the most external cortical layers, and the concomitant evidence of significant upregulation of TNFR2 gene expression only in F− SPMS but not in F+ SPMS cases, suggests that this mechanism may protect both oligodendrocytes and neurons via indirect pathways. Selective stimulation of human-TNFR2 on astrocytes in culture has been shown to lead to leukaemia inhibitory factor secretion, which promotes oligodendrocyte survival and differentiation [21] and stimulation of TNFR2 on mouse microglia leads to the upregulation of anti-inflammatory cytokines [61]. Thus, the lack of activation of TNFR2-mediated pathways in the MS cortex in the presence of increased meningeal inflammation would have a deleterious effect not only by exacerbating cell loss/damage but also by inhibiting tissue repair. Again, it needs to be noted that the degree in shift in the balance of these competing TNF signalling pathways is variable from case to case. Single-cell RNA sequencing approaches will be needed to verify the cellular compartments in which these changes in TNF signalling are taking place.

## Conclusions

This study suggests that inflammatory and cytotoxic molecules released in the subarachnoid space by meningeal infiltrates could induce a substantial shift in the TNF receptor balance in the cortex and of the associated molecular pathways, resulting in the exacerbation of cortical pathology via increased pro-necroptotic signalling. Further studies are necessary in order to demonstrate this at a protein and functional level. A changing balance of TNF signalling depending on the degree of inflammation also helps to explain the large heterogeneity in the extent of the cortical pathology seen across the spectrum of MS cases. All of the MS patients in the high meningeal inflammation group were characterised as still having active inflammatory activity and progressive disease when they died, exemplified by the earlier age at death in this group. Therefore, we suggest that the current study informs us about the ongoing inflammatory disease processes during the progressive stage of MS and will help design new therapeutic approaches.

## Supplementary information

**Additional file 1: Figure S1.** Tissue blocks were taken from the precentral gyrus of 1 cm thick coronal slices of post-mortem MS brains that had been dissected into 2cmx2cm blocks and snap frozen. Sections from the bocks were then subjected to anti-MOG immunohistochemistry to identify areas of subpial demyelination and NAGM. The areas of tissue were then dissected out and processed as shown in the flow diagram. **Figure S2. A.** Supervised hierarchical clustering of 4658 transcripts showing a significant alteration in any of the groups vs control samples from healthy subjects (*p*<0.01 Fold Change>1.5 vs Control group). A false discovery rate (FDR) assessment was performed by permutating MS and control samples according to the table shown in **B**. 5 MS and 5 control samples were included in each of two groups and FDR analysis was conducted by comparing the statistically different genes between the two groups. Different stringency conditions were tested. The table in panel **C.** shows for each tested condition (p<0.05-0.01; fold change 1.5-2), the resulting numbers of real differentially expressed transcripts (Experimental), the median number of probe sets derived from the permutation analysis (FRD#) and the percentage of FDR with respect to the real values (FDR%). A plot of the results showing p-value/fold cutoff on the x axis, FDR% on the left axis and Experimental values on the right axis is also shown (D). **Figure S3.** A: List of several genes of neuronal/axonal components downregulated in the GML of SPMS respect to healthy controls as revealed by microarray gene expression analysis. B-G: Real-time RT-PCR using the same RNA samples employed in the microarray analysis was performed in order to validate the greatest deregulated gene expression in the motor cortex of MS group compared to controls including: decreased expression of neuronal genes, as the neuronal genes for parvalbumin (PVALB, B) and the large (C), medium (D) and low (E) molecular weight neurofilament proteins (NEFL, NEFM NEFH); increased expression of activated microglia markers, CD68 (F) and inducible NO synthase (iNOS, G) was measured by Real-time RT-PCR. For each statistical comparison the p-value, obtained by non-parametric Mann-Whitney test, has been reported (***p<0.001, **p<0.01, *p<0.05).

**Additional file 2: **T**able S1.** Primary antibodies used for immunohistochemistry/immunofluorescence. **Table S2.** Complete list of genes differentially expressed between each MS group and CTR samples 2. T**able S3.** Complete list of 89 Gene Sets significantly modulated in MS samples vs CTR, according to Biocarta Pathway analysis (p<0.05) (significant p-values are in red). **Table S4.** Complete list of 63 Gene Sets significantly modulated in F+SPMS samples vs F-SPMS, according to Biocarta Pathway analysis (p<0.05) (significant p-values are in red). **Table S5.** Complete list of 42 Gene Sets significantly modulated in GML vs NAGM samples, according to Biocarta Pathway analysis (p<0.05) (significant p-values are in red).

## Data Availability

All the data and the datasets supporting the conclusions of this article are included within the article and its additional supplementary tables and figures. The complete data files are available at Gene Expression Omnibus (Accession number GSE135511).
