# Supplementary material for: Meningeal inflammation changes the balance of TNF signalling in cortical grey matter in multiple sclerosis
Source: J Neuroinflammation. 2019 Dec 7;16:259. doi: 10.1186/s12974-019-1650-x (PMC6898969; doi:10.1186/s12974-019-1650-x)
Supplement: Supplementary file 2 — Additional file 2: Table S1. Primary antibodies used for immunohistochemistry/immunofluorescence. Table S2. Complete list of genes differentially expressed between each MS group and CTR samples 2. Table S3. Complete list of 89 Gene Sets significantly modulated in MS samples vs CTR, according to Biocarta Pathway analysis (p<0.05) (significant p-values are in red). Table S4. Complete list of 63 Gene Sets significantly modulated in F+SPMS samples vs F-SPMS, according to Biocarta Pathway analysis (p<0.05) (significant p-values are in red). Table S5. Complete list of 42 Gene Sets significantly modulated in GML vs NAGM samples, according to Biocarta Pathway analysis (p<0.05) (significant p-values are in red). [file 12974_2019_1650_MOESM2_ESM.zip › Suppl table 1.docx]

**Supplementary Table 1**

Primary antibodies used for immunohistochemistry/immunofluorescence

| **Antigen** | **Cell Specificity** | **Clone** | **Dilution** | **Source** |
| --- | --- | --- | --- | --- |
| MOG | Myelin oligodendrocyte glycoprotein | Z12 | 1:50 | Reynolds, London |
| MHC-class II | Antigen presenting cells | CR3/43 | 1:50 | Dako, Carpenteria, CA |
| Olig2 | Oligodendrocytes | Rabbit polyclonal | 1:200 | SinoBiological, Beijing (China) |
| NeuN | Neurons |  | 1:1000 | Chemicon International, Temecula, CA |
| CD68 | Macrophages | KP1 | 1:50 | Dako |
| TNFR1 | TNFRSF1A | H398 | 1:50 | AbD Serotec, Oxford, UK |
| TNFR2 | TNFRSF1B | Rabbit polyclonal | 1:50 | Sigma, St Luise, MO |

For MOG and Olig2 immunostaining, treatment with cold methanol was performed. For TNFRs the staining was performed on fresh cut sections.

Antigen retrieval procedures for CD3, CD8, TNF and TNF-Rs immunostainings utilized microwave of sections in citrate buffer; for CNPase immunostaining, treatment with cold methanol was performed.
