# Supplementary material for: Meningeal inflammation changes the balance of TNF signalling in cortical grey matter in multiple sclerosis
Source: J Neuroinflammation. 2019 Dec 7;16:259. doi: 10.1186/s12974-019-1650-x (PMC6898969; doi:10.1186/s12974-019-1650-x)
Supplement: Supplementary file 2 — Additional file 2: Table S1. Primary antibodies used for immunohistochemistry/immunofluorescence. Table S2. Complete list of genes differentially expressed between each MS group and CTR samples 2. Table S3. Complete list of 89 Gene Sets significantly modulated in MS samples vs CTR, according to Biocarta Pathway analysis (p<0.05) (significant p-values are in red). Table S4. Complete list of 63 Gene Sets significantly modulated in F+SPMS samples vs F-SPMS, according to Biocarta Pathway analysis (p<0.05) (significant p-values are in red). Table S5. Complete list of 42 Gene Sets significantly modulated in GML vs NAGM samples, according to Biocarta Pathway analysis (p<0.05) (significant p-values are in red). [file 12974_2019_1650_MOESM2_ESM.zip › Suppl Table 2.pdf]

Supplementary Table 2

Complete list of genes differentially expressed between each MS group and CTR samples

1) List of genes differentially expressed between F+GML and CTR samples

p<0.01 Fold Change≥1.5 vs Control group

(Fold Change F+ GML/CTR ≥1.5= Up-regulated in F+GML)

(Fold Change F+ GML/CTR ≤0.66= Down-regulated in F+GML)

F+ GML vs CTR					
			Geometric Mean of Intensity		Fold Change
**UniqueID**	**Symbol**	**Parametric p-value**	**CTR**	**F+ GML**	**F+ GML/CTR**
**1381005**	IGLL1	< 1e-07	12.53	280.12	22.36
**1389039**	HLA-DRB1	3.31E-04	4.93	75.63	15.34
**1377798**	FAM9A	2.80E-03	1.55	8.74	5.64
**1645241**	C21orf41	2.32E-04	1.13	5.79	5.12
**1381616**	WFDC5	2.55E-03	1.12	5.21	4.65
**1645763**	SPINT4	5.72E-03	0.58	2.67	4.60
**1386467**	IGJ	1.72E-03	2.57	11.82	4.60
**1644131**	KIF26B	9.23E-03	2.53	11.43	4.52
**1390315**	C6orf150	5.55E-03	1.14	4.68	4.11
**1644651**	FAM29A	7.77E-03	0.92	3.44	3.74
**1383433**	HBG2	7.61E-03	15.69	57.88	3.69
**1644193**	ITFG1	1.90E-03	1.56	5.75	3.69
**1645962**	SEBOX	6.89E-03	1.65	6.08	3.68
**1394705**	PNLIPRP3	5.45E-03	1.03	3.59	3.49
**1381779**	IGSF22	2.26E-04	2.6	8.94	3.44
**1380935**	C4orf17	9.20E-03	1.76	5.92	3.36
**1384695**	KCTD4	1.68E-04	11.08	36.73	3.31
**1377726**	ASB15	2.76E-03	1.63	5.23	3.21
**1391547**	NUDT4	8.91E-03	2.49	7.85	3.15
**1392659**	SLAMF1	9.19E-03	2.95	9.17	3.11
**1644798**	DRD2	2.94E-04	2.85	8.81	3.09
**1378798**	SLC17A4	6.72E-03	2.2	6.8	3.09
**1397122**	IL7R	5.28E-04	3.25	10	3.08
**1380123**	LBP	8.40E-03	1.27	3.86	3.04
**1645071**	CENPE	7.02E-03	2.37	7.13	3.01
**1378218**	CCT8L2	1.49E-03	1.96	5.87	2.99
**1379621**	SERPINE1	8.97E-03	12.92	38.68	2.99
**1646572**	PRAMEF10	4.02E-03	1.45	4.07	2.81
**1393216**	CARD14	8.74E-04	2.02	5.6	2.77
**1645166**	CASP12	9.91E-03	3.29	8.92	2.71
**1383005**	CTAGE5	5.04E-03	2.67	7.05	2.64
**1378600**	FAM46C	4.50E-06	20.49	52.96	2.58
**1393202**	LELP1	3.51E-03	3.69	9.48	2.57

**1389663**	CAMP	1.74E-03	4.24	10.82	2.55
**1385341**	NRTN	5.82E-03	2.91	7.31	2.51
**1377824**	NPC1L1	8.63E-03	4.29	10.62	2.48
**1396240**	SLC5A3	6.67E-04	28.47	70.27	2.47
**1391690**	TPRG1	4.85E-03	3.5	8.62	2.46
**1377837**	MGC29506	9.77E-04	19.93	48.42	2.43
**1394564**	CD79A	9.90E-05	13.68	32.2	2.35
**1392232**	PMCH	1.07E-03	3.48	8.17	2.35
**1380400**	SFMBT2	1.07E-05	34.74	80.36	2.31
**1395534**	SLFNL1	1.52E-03	4.5	10.38	2.31
**1386335**	MST1R	1.42E-03	3.89	8.92	2.29
**1644789**	DUSP5P	4.68E-04	13.04	29.79	2.28
**1643486**	ZNF345	9.85E-03	3.36	7.55	2.25
**1392814**	ANKRD1	1.37E-03	3.86	8.64	2.24
**1393443**	APOC1	4.51E-03	111.89	249.6	2.23
**1385912**	MICB	8.93E-03	20.64	45.77	2.22
**1390387**	CLEC5A	7.68E-03	9.14	20.09	2.20
**1395882**	C1orf157	4.61E-03	4.04	8.88	2.20
**1395261**	GHR	1.59E-03	29.44	64.62	2.19
**1390964**	TBC1D26	2.79E-05	17.29	37.88	2.19
**1386932**	CT45-4	1.71E-03	10.81	23.63	2.19
**1643558**	WNT7B	1.74E-05	26.18	57.11	2.18
**1395371**	IL9	9.37E-03	3.28	7.14	2.18
**1390518**	EFNA2	9.13E-03	2.04	4.37	2.14
**1387613**	LRRIQ1	7.00E-04	4.59	9.83	2.14
**1643454**	ZNF831	1.02E-05	58.2	124.13	2.13
**1396398**	RAB7B	2.45E-04	35.03	74.46	2.13
**1646049**	B3GALT5	9.62E-04	2.71	5.75	2.12
**1392878**	ESR1	2.66E-03	6.1	12.92	2.12
**1378270**	POU4F1	8.76E-03	4.66	9.85	2.11
**1386588**	MLXIP	1.79E-03	5.89	12.4	2.11
**1646067**	ATP6V0A4	7.13E-03	5.39	11.29	2.09
**1390993**	CXCL11	4.06E-03	2.76	5.78	2.09
**1382576**	SP7	7.26E-03	4.02	8.37	2.08
**1394770**	NMNAT2	9.35E-03	3.91	8.1	2.07
**1388535**	IFT122	8.25E-03	2.67	5.52	2.07
**1380959**	LYPD1	1.02E-03	43.52	89.93	2.07
**1379097**	SERTAD1	6.79E-03	185.59	380.47	2.05
**1390353**	SLIT3	6.11E-05	35.7	72.76	2.04
**1395016**	AGTR1	7.13E-03	3.65	7.36	2.02
**1379339**	TCAP	9.64E-03	9.43	18.96	2.01
**1393105**	PCOLCE2	3.30E-03	25.53	51.33	2.01
**1386522**	ODF1	5.20E-06	7.53	15.13	2.01
**1395945**	MS4A8B	2.05E-03	9.07	18.22	2.01
**1378792**	OXTR	1.07E-03	43.44	86.04	1.98
**1386218**	GDAP2	5.10E-03	8.57	16.73	1.95
**1388524**	BCCIP	4.60E-03	13.23	25.7	1.94
**1383056**	KCNG1	2.67E-03	132.08	256.43	1.94

**1387606**	PTER	6.34E-03	3.61	6.98	1.93
**1378314**	SEZ6	3.56E-04	163.6	315.16	1.93
**1392303**	SCYE1	9.38E-03	3.65	7.03	1.93
**1380102**	TMEM26	3.65E-04	18.68	35.78	1.92
**1382630**	HLA-DRB3	2.43E-03	15.54	29.73	1.91
**1395225**	ELF5	3.21E-03	3.52	6.69	1.90
**1388928**	TSPAN16	1.66E-03	9.28	17.51	1.89
**1392226**	RUNX3	1.51E-03	21.36	40.1	1.88
**1386926**	WSCD1	1.72E-03	50.18	94.11	1.88
**1378264**	ABCD1	1.73E-03	8.56	16.02	1.87
**1388090**	PARVG	8.93E-03	45.91	85.84	1.87
**1385519**	UNC13D	8.57E-03	8.83	16.49	1.87
**1646393**	RHOD	7.15E-03	11.08	20.62	1.86
**1393650**	ANXA1	6.05E-03	199.62	371.17	1.86
**1395954**	HPX	2.50E-03	6.51	12.07	1.85
**1395835**	KCNN3	3.10E-05	115.81	213.4	1.84
**1382594**	PLXDC2	8.52E-03	43.29	79.72	1.84
**1396741**	GZMK	1.31E-03	8.55	15.74	1.84
**1380447**	PLEKHA4	9.86E-03	20.55	37.65	1.83
**1395553**	FZD2	5.21E-03	25	45.62	1.82
**1377897**	CPM	2.07E-03	5.47	9.98	1.82
**1645818**	SNAI2	1.41E-03	18.54	33.79	1.82
**1378478**	SRGAP1	1.04E-03	55.51	100.39	1.81
**1644195**	ITGAX	6.37E-03	30.56	55.21	1.81
**1385882**	KRAS	7.13E-03	3.56	6.43	1.81
**1396692**	ODF2L	4.43E-03	12.74	22.94	1.80
**1389268**	PML	9.49E-03	10.08	18	1.79
**1384359**	FLJ41649	4.99E-04	19.15	34.09	1.78
**1387656**	CNGB1	3.35E-03	76.76	136.57	1.78
**1393809**	IQGAP2	2.87E-03	8.15	14.49	1.78
**1382698**	SLC1A5	5.28E-03	13.63	24.22	1.78
**1384640**	PTCHD1	2.00E-06	230.67	409.3	1.77
**1389903**	ISLR2	5.45E-03	264.28	468.92	1.77
**1646097**	ATF3	7.55E-03	55.24	97.76	1.77
**1395339**	SYT14	2.64E-03	16	28.31	1.77
**1376932**	CAMK2D	8.23E-04	51.18	90.54	1.77
**1382602**	SLC25A41	3.05E-03	10.08	17.81	1.77
**1381124**	WDR66	2.31E-04	24.35	42.91	1.76
**1388036**	PXT1	7.19E-03	4.2	7.4	1.76
**1387894**	TTYH3	5.87E-04	212.12	370.21	1.75
**1390081**	FPR3	5.13E-03	8.77	15.29	1.74
**1396637**	TMSL8	2.62E-03	13.89	24.18	1.74
**1382787**	SERINC5	4.21E-03	9.95	17.32	1.74
**1646449**	RBM39	3.16E-04	427.51	743.54	1.74
**1377253**	TAOK2	1.16E-04	106.9	185.7	1.74
**1381335**	BIRC3	5.43E-04	24.52	42.58	1.74
**1391175**	ALDH1A2	6.97E-03	53.02	92.04	1.74
**1389365**	UBD	8.34E-03	5.53	9.58	1.73

**1390529**	VPS37B	3.05E-05	131.63	227.65	1.73
**1380642**	DDX3X	3.82E-03	7.35	12.71	1.73
**1390012**	CAMK2D	2.98E-03	22.85	39.5	1.73
**1385522**	ZDHHC18	4.41E-03	35.48	61.3	1.73
**1377210**	JSRP1	3.26E-04	15.19	26.13	1.72
**1377929**	KIAA1529	7.05E-03	3.98	6.82	1.71
**1378240**	TNRC4	2.70E-05	484.61	829.39	1.71
**1394151**	C10orf85	6.78E-03	53.21	90.67	1.70
**1379797**	FLJ14107	8.25E-03	9.58	16.32	1.70
**1387531**	ZDHHC23	1.60E-04	78.87	133.89	1.70
**1643814**	37681	7.89E-03	58.17	98.66	1.70
**1385530**	GLIS3	9.80E-03	36.12	61.22	1.69
**1379626**	FLJ33590	3.19E-03	637.97	1077.57	1.69
**1644267**	HYDIN	1.09E-03	10.81	18.24	1.69
**1382522**	SDF2L1	5.01E-03	436.35	732.31	1.68
**1380875**	ANKRD6	4.18E-03	157.14	262.41	1.67
**1390503**	GRAMD2	5.62E-03	29.41	49.03	1.67
**1380637**	HIST1H1A	5.78E-04	14.35	23.91	1.67
**1388964**	KCNQ2	5.10E-04	44.91	74.8	1.67
**1646462**	RAET1L	6.60E-03	5.93	9.83	1.66
**1384438**	RNF150	5.00E-07	509.1	842.18	1.65
**1377399**	PNMA5	8.93E-03	59.29	98.08	1.65
**1394202**	GLI2	3.76E-03	13.79	22.78	1.65
**1393378**	GNG4	9.19E-03	63.18	104.19	1.65
**1381351**	HCG9	8.03E-03	16.54	27.22	1.65
**1390479**	FAM124A	7.72E-03	13.12	21.59	1.65
**1392464**	ZNF701	4.19E-03	6.88	11.31	1.64
**1388459**	DUSP8	5.73E-03	437.78	718.89	1.64
**1380073**	OLFML2B	3.20E-03	29.16	47.73	1.64
**1393062**	ELK1	8.84E-04	584.29	955.05	1.63
**1379043**	HTR3A	9.02E-03	19.12	31.11	1.63
**1386496**	SYTL2	3.50E-03	28.72	46.72	1.63
**1378636**	SEPN1	1.89E-03	280.06	455.03	1.62
**1382933**	ZNF609	1.07E-03	88.78	144.02	1.62
**1394993**	ZC3HAV1	8.22E-03	153.01	247.95	1.62
**1379726**	CACNA1C	5.54E-03	96.54	156.24	1.62
**1378468**	JUN	6.75E-04	827.17	1338.13	1.62
**1396184**	SLC2A4RG	1.79E-03	36.54	59.1	1.62
**1388607**	LTBP4	7.99E-04	62.22	100.56	1.62
**1377491**	DUSP18	1.64E-03	91.05	147.13	1.62
**1387595**	KIF12	8.56E-03	10.64	17.19	1.62
**1646748**	PCDH18	4.64E-04	98.3	158.69	1.61
**1396546**	TTC38	7.93E-03	38.94	62.75	1.61
**1388604**	ABCA1	5.13E-03	336.09	540.52	1.61
**1390885**	CPNE3	1.81E-03	482.17	774.47	1.61
**1384341**	GDF15	3.79E-04	12.95	20.68	1.60
**1395057**	PTGER4	1.02E-03	30.13	48.07	1.60
**1392446**	MYB	1.32E-03	21.8	34.73	1.59

**1391031**	SMC4	9.03E-03	7.22	11.5	1.59
**1389471**	LMAN2L	1.82E-03	148.17	235.71	1.59
**1391016**	STH	2.93E-03	53.05	84.21	1.59
**1396745**	BLM	2.90E-04	25.96	41.13	1.58
**1381751**	CBFB	7.23E-03	97.08	153.53	1.58
**1390850**	ASCL2	4.55E-03	35.33	55.8	1.58
**1384465**	SP100	9.07E-03	9.97	15.73	1.58
**1378251**	MAPK7	1.31E-03	52.18	82.32	1.58
**1380834**	RAI14	9.98E-03	123.8	194.23	1.57
**1383148**	SHF	4.30E-04	21.79	34.14	1.57
**1387066**	LPPR2	6.15E-04	237.16	371.57	1.57
**1381564**	DMWD	7.44E-03	93.15	145.74	1.56
**1646390**	RN7SK	1.80E-03	548.81	857.88	1.56
**1645932**	SEPN1	8.67E-03	33.04	51.61	1.56
**1380001**	IL34	2.34E-03	80.18	125.06	1.56
**1381452**	MOV10	3.54E-03	47.26	73.67	1.56
**1643757**	MGC4677	9.17E-03	360	560.64	1.56
**1394107**	KLKB1	4.80E-03	32.42	50.34	1.55
**1379925**	TMEM137	5.50E-03	59.55	92.31	1.55
**1382685**	ANKRD57	6.92E-04	141.73	219.62	1.55
**1384899**	RGS2	6.93E-03	605.09	936.78	1.55
**1391719**	RBM14	5.71E-04	747.24	1155.89	1.55
**1380401**	PRR5	1.52E-03	94.27	145.54	1.54
**1381021**	ZDHHC14	1.28E-04	342.06	527.26	1.54
**1646902**	ODZ3	9.77E-05	494.29	760.97	1.54
**1379298**	ZBTB40	4.27E-03	107.17	164.98	1.54
**1646418**	RFX2	6.94E-03	24.25	37.23	1.54
**1646238**	AGBL5	1.17E-03	24.35	37.37	1.53
**1387101**	EIF2S2	1.85E-03	45.61	69.92	1.53
**1393598**	CACHD1	6.76E-03	100.97	154.72	1.53
**1385918**	CALU	1.91E-03	168.55	258.06	1.53
**1389198**	FGD1	1.64E-04	117.64	179.96	1.53
**1387855**	PAK6	4.41E-03	334.85	511.91	1.53
**1388298**	FERMT2	1.59E-03	952.57	1454.02	1.53
**1391963**	HSPA5	3.35E-03	400.94	610.98	1.52
**1380882**	MYH7	6.56E-03	57.27	87.25	1.52
**1380724**	PEA15	3.34E-05	5219.68	7940.66	1.52
**1396935**	GALM	4.19E-03	38.16	57.95	1.52
**1395744**	C4orf44	9.42E-03	35.25	53.43	1.52
**1386615**	FLJ41733	6.85E-03	7.04	10.66	1.51
**1378821**	SYT17	1.39E-04	428.96	649.38	1.51
**1381059**	DMD	8.50E-03	59.34	89.76	1.51
**1380976**	PRSS21	3.96E-03	7.58	11.46	1.51
**1381301**	PYGO2	2.38E-03	39.92	60.33	1.51
**1378229**	DTNBP1	6.93E-04	18.06	27.28	1.51
**1380824**	SHC3	2.15E-03	166.2	250.98	1.51
**1377236**	POLN	3.83E-03	36.51	55.12	1.51
**1395936**	FOXF2	6.57E-03	36.14	54.55	1.51

**1382547**	DIAPH2	1.34E-03	78.75	118.86	1.51
**1381391**	FNBP1L	4.70E-03	90.4	136.34	1.51
**1392542**	TRAF2	3.20E-03	38.14	57.46	1.51
**1377377**	MASP1	1.17E-03	17.9	26.9	1.50
**1396132**	PELI1	2.89E-04	151.79	228.09	1.50
**1384720**	VAT1	4.53E-03	96.77	145.36	1.50
**1387823**	GABRD	9.09E-03	267.63	178.38	0.67
**1646060**	ATP9A	8.73E-03	35.61	23.72	0.67
**1395201**	ZNF585A	2.08E-03	49.63	33.05	0.67
**1386935**	APP	8.03E-03	3567.97	2375.3	0.67
**1380861**	ZNF324B	6.77E-03	27.48	18.29	0.67
**1394016**	PPA2	5.23E-03	387.15	257.6	0.67
**1644260**	IDH3B	4.41E-03	1123.66	746.81	0.66
**1378536**	MRPS30	9.00E-04	507.79	337.23	0.66
**1645951**	SEH1L	5.47E-03	157.26	104.41	0.66
**1388932**	ADPRHL1	5.50E-04	144.46	95.91	0.66
**1378737**	NDUFA10	9.83E-03	218.81	145.23	0.66
**1378021**	NDRG3	4.97E-03	1187.94	787.73	0.66
**1394653**	MGC15763	5.68E-03	50.15	33.24	0.66
**1643479**	ZNF536	9.81E-03	99.05	65.61	0.66
**1384633**	ROPN1L	1.59E-03	62.8	41.59	0.66
**1384855**	CBWD5	2.02E-03	134.94	89.33	0.66
**1384718**	UQCRB	3.06E-03	102.35	67.62	0.66
**1644331**	HIGD1A	4.23E-03	2039.96	1347.61	0.66
**1392332**	NCKIPSD	2.62E-03	468.57	309.48	0.66
**1645444**	UBA3	3.97E-03	249.73	164.92	0.66
**1387115**	GSTM2	2.16E-05	2788.06	1838.92	0.66
**1391407**	TTBK2	8.72E-03	35.86	23.64	0.66
**1395845**	ILF3	7.36E-04	105.55	69.41	0.66
**1381771**	CHMP2A	3.74E-03	135.96	89.25	0.66
**1389381**	GNB5	3.71E-03	251.63	164.89	0.66
**1393089**	RSRC1	1.22E-04	156.74	102.48	0.65
**1392751**	KRIT1	9.71E-03	13.85	9.04	0.65
**1380626**	TBRG1	9.56E-03	32.74	21.36	0.65
**1391830**	MAPK9	1.55E-03	642	418.19	0.65
**1392460**	GSTM1	8.50E-06	1614.7	1051.63	0.65
**1392667**	CASC4	4.14E-03	66.77	43.46	0.65
**1392126**	ASB13	1.84E-03	279.85	181.85	0.65
**1386405**	C6orf106	2.35E-03	57.16	37.13	0.65
**1646072**	ATP5A1	9.31E-04	7639.38	4960.42	0.65
**1378137**	PIB5PA	7.40E-03	174.27	113.1	0.65
**1382752**	RPA3	1.27E-03	519.83	337.36	0.65
**1379289**	NINJ2	9.11E-03	380.29	246.44	0.65
**1387911**	SLC2A11	1.25E-03	122.99	79.7	0.65
**1381048**	FEZ1	1.11E-03	771.82	500.1	0.65
**1645950**	SEDLP	2.98E-03	146.33	94.7	0.65
**1381295**	ANKRD16	4.21E-03	66.7	43.16	0.65
**1386173**	UGP2	5.62E-04	1101.59	712.53	0.65

**1395568**	LDB1	9.42E-03	17.07	11.04	0.65
**1381097**	SMYD2	3.34E-03	529.25	342	0.65
**1646673**	PIAS2	1.41E-03	44.66	28.84	0.65
**1380493**	PLCH1	5.06E-03	103.56	66.77	0.64
**1383170**	LIN52	1.49E-03	60.66	39.01	0.64
**1646131**	ARHGAP9	2.28E-04	42.1	27.06	0.64
**1393856**	PPM1A	7.46E-03	58.96	37.78	0.64
**1383782**	C18orf10	9.97E-03	336.38	215.37	0.64
**1389904**	RAD23B	7.44E-03	426.89	273.3	0.64
**1383637**	CYP2E1	3.01E-03	197	126.05	0.64
**1391585**	C16orf86	2.27E-03	37.81	24.13	0.64
**1380195**	SUCLA2	3.68E-03	850.94	541.9	0.64
**1645849**	SLC4A8	5.42E-05	44.33	28.23	0.64
**1377382**	MAD1L1	1.75E-03	97.95	62.33	0.64
**1377548**	NDUFS2	6.83E-03	55.77	35.47	0.64
**1392100**	CAPN3	9.07E-03	312.26	198.36	0.64
**1383369**	TM2D3	3.11E-03	102.72	65.21	0.63
**1389027**	ARMC8	1.92E-03	175.36	111.28	0.63
**1645759**	SPIN2B	7.06E-03	61.95	39.2	0.63
**1384806**	GPR155	4.27E-03	10.52	6.65	0.63
**1394977**	MPPED1	9.24E-03	32.69	20.66	0.63
**1377901**	SCN2B	7.59E-03	1253.51	791.66	0.63
**1381722**	RASGRF1	1.41E-03	57.43	36.21	0.63
**1378701**	ACADSB	4.11E-03	55.75	35.14	0.63
**1381622**	UCHL5	1.50E-03	346.08	218.08	0.63
**1387715**	PIK3CB	3.74E-03	143.13	90.14	0.63
**1379210**	ATP1A1	1.17E-03	2624.35	1652.54	0.63
**1383583**	EXTL2	8.13E-05	713.74	449.09	0.63
**1378726**	GNG13	9.81E-03	31.42	19.76	0.63
**1379268**	HECW1	3.56E-04	48.18	30.3	0.63
**1391061**	MKKS	7.52E-03	47.09	29.6	0.63
**1378883**	SNTG1	4.32E-03	93.12	58.47	0.63
**1384437**	TRO	6.83E-04	404.83	253.13	0.63
**1396014**	KCTD9	9.90E-03	95.71	59.73	0.62
**1380065**	SEPSECS	1.73E-05	95.31	59.45	0.62
**1644086**	LAMA2	6.97E-03	70.07	43.64	0.62
**1387334**	GLRX2	9.24E-03	146.3	91.1	0.62
**1388745**	SLC2A11	3.36E-04	72.39	45.06	0.62
**1379237**	SLC25A46	3.63E-03	232.49	144.68	0.62
**1380429**	RET	7.36E-03	21.12	13.13	0.62
**1389165**	CREBZF	4.01E-03	81.42	50.6	0.62
**1381251**	CAMK2G	3.87E-03	1704.54	1059.1	0.62
**1645405**	USP14	8.77E-03	225.49	139.96	0.62
**1392824**	MATR3	6.76E-03	205.11	127.19	0.62
**1387936**	BMP2K	7.90E-06	38.59	23.9	0.62
**1397189**	RASGRP2	3.87E-04	89.87	55.65	0.62
**1381184**	C14orf124	1.36E-03	108.62	67.15	0.62
**1385566**	PDZD7	8.27E-03	72.49	44.81	0.62

**1381888**	CDCA5	3.65E-03	142.17	87.87	0.62
**1645251**	C20orf100	5.60E-03	1594.76	985.29	0.62
**1646379**	RNF14	1.77E-03	397.21	245.09	0.62
**1385842**	C12orf24	1.34E-03	707.34	435.99	0.62
**1382349**	MGC16291	4.87E-03	23.35	14.37	0.62
**1381809**	F8	3.49E-03	23.5	14.46	0.62
**1382558**	AMACR	7.36E-03	100.41	61.71	0.61
**1386973**	DRP2	9.79E-03	22.96	14.11	0.61
**1378751**	EID2	2.72E-03	360.35	221.01	0.61
**1396933**	GPR89A	3.51E-03	87	53.32	0.61
**1646325**	ABCA11	4.80E-03	53.12	32.55	0.61
**1377319**	C4orf27	2.38E-04	288.25	176.41	0.61
**1391554**	MRS2	1.42E-03	61.45	37.58	0.61
**1395635**	LGI3	4.46E-03	347.47	212.49	0.61
**1383032**	PFAAP5	1.02E-03	319.08	195.12	0.61
**1646521**	PSMC4	2.48E-03	220.06	134.25	0.61
**1386044**	OXCT2	7.41E-04	105.97	64.59	0.61
**1396788**	PARK2	1.50E-03	34.41	20.96	0.61
**1387919**	NEFL	6.70E-03	2247.19	1368.04	0.61
**1389580**	MAT2B	6.87E-03	30.7	18.67	0.61
**1382226**	RFC2	6.79E-04	34.71	21.08	0.61
**1395633**	FAM80A	2.84E-03	103.27	62.69	0.61
**1646088**	ATG4B	9.38E-03	296.43	179.61	0.61
**1393282**	PAIP2	6.57E-04	37.59	22.75	0.61
**1383914**	C6orf65	4.99E-04	1183.37	713.17	0.60
**1382399**	GRIN2A	2.46E-03	79.65	48	0.60
**1390021**	APITD1	3.77E-03	72.69	43.75	0.60
**1645170**	CAPN3	2.92E-03	204.82	122.81	0.60
**1392679**	CLU	1.84E-03	63.11	37.82	0.60
**1381297**	C7orf46	1.47E-03	33.78	20.24	0.60
**1387798**	CABP1	4.90E-03	214.35	128.38	0.60
**1644180**	KCMF1	3.98E-04	264.21	158.04	0.60
**1395364**	PRKCB1	5.48E-03	725.07	432.98	0.60
**1380393**	THAP7	1.32E-03	25.18	15.01	0.60
**1384834**	ASAH1	2.55E-03	190.55	113.49	0.60
**1392255**	AUH	3.22E-03	474.03	282.13	0.60
**1380760**	GAD1	4.88E-03	36.73	21.86	0.60
**1397270**	TMC4	5.65E-03	9.22	5.48	0.59
**1643778**	MEIS3P1	1.10E-05	185.75	110.34	0.59
**1645590**	TMEM120B	1.14E-03	26.38	15.67	0.59
**1386064**	HACL1	4.95E-04	520.37	308.75	0.59
**1384540**	TSC22D1	4.18E-03	1775.21	1053.16	0.59
**1396977**	SCN8A	8.95E-03	26.52	15.73	0.59
**1391068**	FGF9	5.12E-05	699.45	414.7	0.59
**1646458**	RAP1GDS1	9.76E-03	114.15	67.52	0.59
**1644860**	DGKH	4.58E-03	10.35	6.12	0.59
**1383382**	GLS2	1.33E-04	180.35	106.56	0.59
**1388266**	CCT6A	4.04E-03	152.42	89.8	0.59

**1390772**	DLD	2.00E-03	282.69	166.37	0.59
**1380608**	PPAPDC2	2.51E-03	112.12	65.97	0.59
**1394862**	PSMG1	3.38E-03	270.29	158.93	0.59
**1393503**	DYNC2LI1	8.16E-03	270.2	157.83	0.58
**1389211**	PSMA1	3.84E-03	193.24	112.85	0.58
**1385135**	SLFN5	1.26E-03	19.02	11.09	0.58
**1382390**	SULT1A1	8.03E-03	203.62	118.51	0.58
**1377588**	PRDX2	1.43E-03	725.72	421.53	0.58
**1383506**	CENPC1	4.30E-03	10.56	6.13	0.58
**1393805**	ATP5C1	5.90E-03	764.17	441.19	0.58
**1393995**	CDC42SE2	3.68E-03	49.93	28.79	0.58
**1383198**	C3orf57	9.23E-04	21.82	12.58	0.58
**1381882**	GSTM4	7.84E-04	35.97	20.71	0.58
**1382748**	C14orf138	2.71E-04	164.8	94.74	0.57
**1396774**	UQCRC2	5.50E-03	288.15	165.5	0.57
**1382408**	CLCN4	2.58E-03	72.8	41.77	0.57
**1645438**	UBE2E3	3.08E-03	285.89	163.79	0.57
**1387399**	CA14	3.54E-03	30.65	17.54	0.57
**1382038**	SRPK2	3.94E-03	73.3	41.93	0.57
**1386265**	FCRLB	1.21E-03	134.64	76.85	0.57
**1389956**	CBLN2	7.86E-04	812.75	463.77	0.57
**1392687**	SLC37A3	8.53E-03	36.51	20.83	0.57
**1378095**	PLEKHB2	8.05E-03	358.92	204.68	0.57
**1389253**	ABHD11	2.29E-04	47.85	26.93	0.56
**1646305**	ABHD11	1.15E-04	40.06	22.54	0.56
**1394339**	SEPT5	9.15E-03	64.22	36.09	0.56
**1385204**	TM6SF1	1.85E-04	377.54	211.18	0.56
**1384092**	MAGED1	4.61E-04	22.6	12.64	0.56
**1396282**	GCC2	4.54E-04	32.52	18.15	0.56
**1387157**	RASSF6	5.74E-03	4.54	2.53	0.56
**1644113**	KLHDC9	1.99E-03	301.12	167.35	0.56
**1645392**	USP33	7.32E-05	79.46	44.04	0.55
**1388599**	SLC25A12	3.77E-04	668.23	370.27	0.55
**1645686**	SYNJ1	8.61E-03	78.33	43.4	0.55
**1646680**	PHYH	7.39E-05	157.59	87.27	0.55
**1390986**	PCGF6	3.47E-04	23.21	12.85	0.55
**1385080**	TUBGCP5	1.39E-03	203.11	112.36	0.55
**1396998**	PPM1A	3.99E-04	102.11	56.46	0.55
**1377965**	RAB37	7.64E-03	145.64	80.45	0.55
**1644690**	EXTL2	2.91E-05	286.31	157.48	0.55
**1389131**	CORO2A	1.51E-04	72.51	39.87	0.55
**1379429**	TMEM27	7.61E-03	20.71	11.38	0.55
**1644013**	LOC285359	3.61E-03	11.29	6.17	0.55
**1386981**	C19orf18	4.15E-03	21.32	11.65	0.55
**1646179**	ANKRD29	2.96E-05	312.11	170.41	0.55
**1381967**	USH1C	9.23E-03	9.38	5.12	0.55
**1394514**	CDC42	9.80E-03	108.98	59.42	0.55
**1389460**	CADPS2	1.10E-03	526.57	287.04	0.55

**1391132**	NME2	7.99E-03	39.72	21.56	0.54
**1396319**	SCP2	2.88E-04	132.41	71.84	0.54
**1386859**	CHGA	3.82E-04	2167.22	1172.29	0.54
**1392537**	PSMG1	3.22E-03	172.02	92.91	0.54
**1390046**	C13orf16	9.84E-03	165.6	89.44	0.54
**1384585**	PDCD10	1.95E-03	49.68	26.71	0.54
**1388064**	ATP6V1H	9.85E-03	38.31	20.53	0.54
**1394136**	EEF1B2	1.92E-03	139.82	74.44	0.53
**1384568**	UQCC	1.45E-03	47.23	25.14	0.53
**1377405**	FASTKD3	1.82E-04	59.79	31.75	0.53
**1393661**	RPH3A	1.73E-03	143.09	75.98	0.53
**1393284**	GDAP1	7.16E-03	99.63	52.69	0.53
**1389939**	HMGCLL1	4.19E-03	55.29	29.24	0.53
**1396080**	TRIM37	3.66E-03	1096.66	579.39	0.53
**1383440**	TBR1	6.11E-03	162.03	85.02	0.52
**1387569**	RIT2	3.71E-03	238.6	125.07	0.52
**1393575**	RELL2	3.36E-04	226.75	118.46	0.52
**1389358**	TSC22D3	1.21E-03	25.95	13.53	0.52
**1377415**	EMILIN3	1.85E-03	29.12	15.15	0.52
**1383377**	RBM11	3.02E-03	50.09	26	0.52
**1396881**	KCNA1	2.41E-04	32.56	16.76	0.51
**1390880**	FBXW7	2.01E-03	212.86	109.42	0.51
**1645936**	SEPT4	8.52E-03	94.99	48.82	0.51
**1646555**	PRKACB	1.76E-03	193.9	99.58	0.51
**1388812**	RTN3	1.62E-03	95.31	48.78	0.51
**1380702**	MAEA	1.12E-03	100.59	51.29	0.51
**1387456**	TNNT2	2.34E-04	229.54	116.94	0.51
**1645878**	SLC22A18	1.36E-04	107.18	54.4	0.51
**1380538**	NOMO3	3.49E-03	46.09	23.39	0.51
**1383057**	SCAMP1	1.94E-03	138.04	69.84	0.51
**1381675**	AMACR	5.46E-03	88.35	44.04	0.50
**1645176**	CAMK2G	6.04E-04	663	330.34	0.50
**1378972**	ESRRG	4.29E-03	250.57	124.82	0.50
**1396699**	FAM53A	1.98E-03	7.58	3.77	0.50
**1387454**	SPAG8	9.85E-03	28.23	14.04	0.50
**1394203**	ELMO1	4.76E-03	193.36	96.11	0.50
**1391134**	ATP5G1	1.04E-03	116.78	57.96	0.50
**1380344**	PPP2R5D	3.80E-03	38.92	19.27	0.50
**1388773**	FBXO17	7.24E-04	9.48	4.69	0.49
**1392403**	LOC729399	2.55E-04	73.54	36.18	0.49
**1382774**	RWDD1	3.52E-04	194.41	95.27	0.49
**1392288**	MAGED2	5.87E-05	49.62	24.16	0.49
**1396459**	VPS16	7.12E-05	34.89	16.96	0.49
**1387885**	LYNX1	3.26E-04	37.69	18.3	0.49
**1385252**	ELAVL2	5.08E-03	189.21	91.41	0.48
**1645798**	SNAP25	7.26E-03	5513.37	2660.72	0.48
**1383310**	FBLN7	1.77E-03	146.5	70.66	0.48
**1390148**	CADPS	3.43E-05	170.01	81.55	0.48

**1384233**	GRB14	8.38E-03	6.18	2.96	0.48
**1377663**	ROS1	3.81E-03	20.99	10.03	0.48
**1386431**	PEBP4	2.06E-03	30.34	14.48	0.48
**1392178**	FGF14	3.69E-03	153.6	73.25	0.48
**1377231**	C9orf72	1.29E-04	33.86	16.13	0.48
**1396033**	PHLDA2	6.31E-04	27.5	13.06	0.47
**1391313**	DLGAP1	4.32E-03	43.46	20.57	0.47
**1386691**	EYA3	3.01E-03	15.95	7.54	0.47
**1393415**	NUDT18	5.95E-03	177.13	83.54	0.47
**1384817**	TRUB1	7.87E-03	71.86	33.88	0.47
**1384482**	FBXO9	3.65E-03	97.7	45.83	0.47
**1389428**	MDFI	6.93E-03	11.57	5.36	0.46
**1379348**	SLC22A18AS	1.87E-03	83.92	38.44	0.46
**1377858**	C15orf26	9.69E-03	10.79	4.93	0.46
**1392947**	FAIM	4.39E-03	17.56	8	0.46
**1387336**	CABP1	1.22E-04	128.45	58.11	0.45
**1645456**	TTLL8	7.43E-03	9.19	4.13	0.45
**1383809**	TBRG4	6.82E-03	16.37	7.34	0.45
**1384744**	OPN3	2.91E-03	367.22	164.54	0.45
**1384366**	MORF4L1	4.89E-04	22.81	10.2	0.45
**1645135**	CCNL2	1.04E-03	85.56	38.13	0.45
**1390841**	NEFM	5.85E-05	3599.84	1565.66	0.43
**1382607**	RIBC2	7.54E-03	5.89	2.53	0.43
**1646886**	OPN3	5.75E-03	93.58	39.01	0.42
**1397081**	PCP4	3.32E-05	2761.72	1146.41	0.42
**1388130**	USP12	5.45E-03	12.72	5.25	0.41
**1396358**	TRIM37	1.29E-03	230.18	94.88	0.41
**1382854**	NRG1	3.05E-03	35.61	14.55	0.41
**1644158**	KCNQ5	1.06E-03	24.94	10.16	0.41
**1386613**	FBXO44	1.00E-04	9.47	3.85	0.41
**1392346**	SLC25A40	9.72E-05	105.55	42.7	0.40
**1383903**	KCNC1	3.98E-05	53.96	21.77	0.40
**1378185**	TPD52	4.35E-03	37.74	14.97	0.40
**1377573**	STAT4	1.84E-03	628.75	246.86	0.39
**1378328**	HSD11B1	5.50E-06	114.47	44.62	0.39
**1384214**	HTR5A	9.92E-03	37.42	14.54	0.39
**1393966**	WBSCR23	9.89E-03	4.38	1.68	0.38
**1383049**	CNDP1	2.26E-04	296.49	113.01	0.38
**1388744**	RTN3	2.28E-03	168.34	63.51	0.38
**1385278**	HAPLN4	4.34E-03	979.1	366.83	0.37
**1394677**	SHD	5.15E-04	219.92	81.74	0.37
**1383406**	MAP1B	8.02E-04	1373.75	479.29	0.35
**1397381**	HSD11B1	1.60E-06	86.28	30.1	0.35
**1645390**	VAMP1	2.40E-05	151.86	50.98	0.34
**1377361**	NEFH	3.85E-04	1878.39	627.95	0.33
**1397061**	VAMP1	2.80E-06	2937.83	954.41	0.32
**1380551**	CSN1S1	1.56E-04	21.67	6.73	0.31
**1379809**	SCN1B	2.60E-06	395.32	115.11	0.29

**1644941**	CTXN3	5.14E-04	156.01	39.56	0.25
**1391652**	GTF3C6	5.47E-03	5.22	1.23	0.24
**1390766**	PRPF40B	5.91E-03	6.49	1.44	0.22
**1645227**	C3orf57	8.10E-06	28.79	6.33	0.22
**1645661**	TBC1D3C	4.16E-03	3.46	0.71	0.21
**1386098**	EFCAB2	2.01E-03	2.93	0.6	0.20
**1391600**	PVALB	1.94E-04	1132.23	185.49	0.16
**1381346**	NFATC2	4.43E-03	4.68	0.73	0.16
**1396101**	MIP	7.46E-03	5.44	0.76	0.14

2) List of genes differentially expressed between F-GML and CTR samples

p<0.01 Fold Change≥1.5 vs Control group

(Fold Change F- GML/CTR ≥1.5= Up-regulated in F-GML)

(Fold Change F- GML/CTR ≤0.66= Down-regulated in F-GML)

F- GML vs CTR					
			Geometric Mean of Intensity		Fold Change
UniqueID	Symbol	Parametric p-value	CTR	F- GML	F- GML/CTR
1389039	HLA-DRB1	3.40E-05	4.93	93.94	19.05
1381814	HLA-DRB5	5.94E-04	6.96	120.9	17.37
1392290	SERPINA3	6.25E-03	277.4	2159.07	7.78
1643579	WDR47	2.57E-04	3.26	22.76	6.98
1644613	FCGBP	7.17E-04	205.09	1365.27	6.66
1377824	NPC1L1	2.00E-06	4.29	20.42	4.76
1379832	CSNK1A1L	1.29E-05	1.41	6.62	4.70
1381005	IGLL1	1.02E-04	12.53	60.13	4.80
1644879	DEFB114	3.27E-03	1.05	5.05	4.81
1644385	GSTM1L	6.44E-03	0.8	3.9	4.88
1392709	LOC389517	6.21E-03	1.03	4.62	4.49
1390306	ANGPT2	1.31E-04	60.66	260.25	4.29
1383734	SLC12A3	3.51E-03	1.41	6.24	4.43
1391959	BCMO1	4.14E-04	12.4	51.53	4.16
1391999	LDB3	3.16E-03	0.93	3.98	4.28
1380783	SERPINA5	7.93E-04	5.22	21.05	4.03
1397370	SCIN	9.23E-04	35.35	139.98	3.96
1644651	FAM29A	4.29E-03	0.92	3.62	3.93
1644612	FCGBP	1.07E-03	15.83	60.6	3.83
1396240	SLC5A3	4.00E-07	28.47	105.72	3.71
1381800	GFAP	7.00E-07	1626.88	6002.48	3.69
1377223	LOC402573	1.20E-06	9.56	35.38	3.70
1381616	WFDC5	3.67E-03	1.12	4.08	3.64
1390131	S100A3	4.50E-03	4.52	16.6	3.67
1396234	LOC283755	6.07E-05	36.05	130.67	3.62
1377828	C10orf10	3.13E-04	196.57	692.85	3.52
1380109	MARCH8	1.24E-03	8.43	30.27	3.59
1393547	ADAMTS9	1.12E-04	61.73	213.44	3.46
1397153	CHI3L2	2.84E-03	17.98	62.55	3.48
1397001	BTBD14A	< 1e-07	63.23	213.62	3.38
1378460	CD44	1.36E-03	121.91	407.3	3.34
1385164	C22orf34	2.97E-03	2.24	7.52	3.36
1393771	GBP2	2.53E-05	121.61	388.35	3.19
1386932	CT45-4	4.84E-04	10.81	34.95	3.23
1392217	STC1	2.78E-03	19.57	63.79	3.26
1391496	RALGPS2	5.08E-03	3.79	12.21	3.22
1384389	CEL	< 1e-07	26.6	82.39	3.10
1383402	SERPINH1	4.00E-07	52.93	164.22	3.10
1384968	HGFAC	4.00E-07	14.7	46.3	3.15
1381799	TIMP1	4.99E-05	581.02	1829.98	3.15
1386288	AQP1	1.35E-04	19.6	61.56	3.14
1645473	TTTY7	8.86E-03	1.75	5.5	3.14
1386137	COL4A1	9.00E-07	137.59	419.4	3.05
1387719	KCNE4	8.30E-04	4.28	12.85	3.00

**1384093**	CCDC80	3.97E-03	2.04	6.14	3.01
**1388753**	CHST6	3.00E-07	45.14	130.93	2.90
**1382454**	LMOD1	5.53E-05	17.46	51.71	2.96
**1377288**	FGF17	< 1e-07	54.94	159.02	2.89
**1381556**	RBMS2	< 1e-07	19.34	54.51	2.82
**1397138**	POU2F2	1.98E-04	4.24	12	2.83
**1383769**	TSKS	3.62E-03	3.05	8.66	2.84
**1395048**	NLRP4	7.37E-03	1.88	5.37	2.86
**1391606**	GJA4	9.00E-07	37.33	102.92	2.76
**1385707**	HMOX1	7.94E-04	24.96	68.89	2.76
**1390389**	MYBPH	1.09E-03	16.36	45.37	2.77
**1395897**	NAPSA	2.40E-03	7.47	20.66	2.77
**1389697**	E2F2	2.78E-03	5.07	14.26	2.81
**1393216**	CARD14	3.10E-04	2.02	5.46	2.70
**1378719**	STON1	1.39E-03	6.51	17.51	2.69
**1383005**	CTAGE5	1.72E-03	2.67	7.19	2.69
**1383911**	TEX14	6.40E-03	3.22	8.6	2.67
**1388470**	PRKCZ	7.67E-03	1.92	5.19	2.70
**1377852**	C21orf29	8.41E-03	2.32	6.21	2.68
**1391000**	TFPI	4.30E-06	41.19	108.23	2.63
**1377170**	GDPD3	7.52E-05	19.93	52.24	2.62
**1377549**	TTPA	2.42E-04	2.61	6.88	2.64
**1379339**	TCAP	2.90E-04	9.43	24.54	2.60
**1396183**	RARRES3	1.38E-03	301.33	803.36	2.67
**1384062**	TEX101	2.61E-03	4.71	12.26	2.60
**1386219**	WWTR1	3.11E-03	3.28	8.61	2.63
**1383246**	ZYG11A	5.49E-03	2.91	7.71	2.65
**1379577**	UBE2C	7.76E-03	4.19	10.91	2.60
**1392530**	CYP3A7	8.41E-03	3.71	9.85	2.65
**1384903**	EVI1	6.30E-06	103.39	263.7	2.55
**1385494**	A4GALT	1.29E-05	24.02	62.19	2.59
**1377281**	BAG3	2.97E-04	411.2	1062.11	2.58
**1392414**	SYTL4	3.81E-04	101	259.26	2.57
**1384973**	P8	3.84E-04	65.06	167.51	2.57
**1379424**	HSPA1A	1.09E-03	449.92	1156.4	2.57
**1386566**	SRPX2	5.07E-03	8.99	22.89	2.55
**1643700**	MSLN	6.17E-03	27.24	69.81	2.56
**1392798**	CCNB2	6.62E-03	4.32	11.08	2.56
**1397108**	HEY2	< 1e-07	70.3	173.91	2.47
**1382940**	HSPB1	< 1e-07	1194.5	2977.27	2.49
**1394788**	ABCA7	2.30E-06	24.92	62.52	2.51
**1379615**	LILRA3	9.00E-06	5.96	15.02	2.52
**1393040**	IER3	1.37E-05	76.62	191.26	2.50
**1388459**	DUSP8	6.50E-05	437.78	1094.14	2.50
**1388090**	PARVG	6.82E-05	45.91	113.87	2.48
**1377486**	CSDA	2.89E-04	453.91	1144.14	2.52
**1396746**	GPR4	5.76E-04	45.63	114.55	2.51
**1645184**	CALCA	1.38E-03	2.82	6.98	2.48
**1385341**	NRTN	2.87E-03	2.91	7.35	2.53
**1389179**	LOC440558	3.16E-03	2.16	5.42	2.51
**1643486**	ZNF345	8.58E-03	3.36	8.35	2.49
**1395360**	VRK2	8.88E-03	3.61	8.99	2.49
**1389766**	ATOH8	6.48E-05	35.99	88.43	2.46

**1376923**	PLA1A	9.11E-05	40.39	98.88	2.45
**1395156**	STAB1	2.41E-04	32.55	80.29	2.47
**1380284**	TMEM149	3.30E-04	55.03	134.19	2.44
**1379359**	FAM129A	4.84E-04	58.63	141.38	2.41
**1377925**	MS4A14	5.22E-04	8.82	21.77	2.47
**1395207**	SUSD2	6.87E-04	41.63	100.75	2.42
**1376977**	THEX1	9.20E-04	1.06	2.6	2.45
**1378792**	OXTR	2.79E-03	43.44	106.17	2.44
**1384915**	KRTAP4-4	4.21E-03	2.12	5.17	2.44
**1391690**	TPRG1	5.55E-03	3.5	8.57	2.45
**1646390**	RN7SK	< 1e-07	548.81	1304.18	2.38
**1389467**	MUC1	3.10E-05	33.97	80.93	2.38
**1378600**	FAM46C	4.30E-05	20.49	48.53	2.37
**1645328**	C10orf54	5.53E-05	123	292.08	2.37
**1393946**	ATHL1	6.29E-05	23.26	55.06	2.37
**1390640**	TLR5	9.54E-05	36	84.97	2.36
**1396050**	AKR1C3	1.65E-04	261.1	614.46	2.35
**1389852**	C12orf28	1.86E-04	11.07	26.27	2.37
**1396710**	MPZL1	6.26E-04	5.87	14.13	2.41
**1393639**	UCP2	1.21E-03	24.77	59.11	2.39
**1385519**	UNC13D	1.42E-03	8.83	20.83	2.36
**1382777**	TRIM5	4.17E-03	5.29	12.48	2.36
**1395371**	IL9	4.50E-03	3.28	7.78	2.37
**1377701**	S100A4	4.68E-03	87.27	209.68	2.40
**1645111**	CD44	6.15E-03	18.42	43.61	2.37
**1381747**	RBM35B	6.84E-03	2.33	5.57	2.39
**1393594**	KIF20A	8.62E-03	5.43	12.83	2.36
**1392254**	BAIAP2L1	5.00E-07	7.93	18.45	2.33
**1392857**	CSAG1	8.00E-07	32.85	77.12	2.35
**1383211**	STEAP3	3.10E-06	13.75	32.14	2.34
**1391016**	STH	4.20E-06	53.05	122.7	2.31
**1378152**	QRICH2	5.70E-06	10.3	23.86	2.32
**1385198**	WFIKKN1	9.70E-06	9.74	22.53	2.31
**1379925**	TMEM137	3.93E-05	59.55	137.28	2.31
**1388614**	TRIM47	4.11E-05	76.77	178.76	2.33
**1378190**	ADORA2A	6.98E-05	55.12	129.49	2.35
**1646393**	RHOD	8.65E-05	11.08	25.75	2.32
**1397167**	CCL19	2.75E-04	29.43	68.75	2.34
**1644348**	HERC2P2	3.87E-04	37.09	85.86	2.31
**1390575**	PTGER1	4.16E-04	20.06	46.6	2.32
**1377929**	KIAA1529	5.24E-04	3.98	9.29	2.33
**1389074**	IFITM2	1.60E-03	2291.03	5288.41	2.31
**1393417**	TMBIM1	1.77E-03	131.19	305.21	2.33
**1377255**	SPP1	2.33E-03	1220.74	2857.88	2.34
**1391314**	RASEF	2.92E-03	5.21	12.06	2.31
**1383273**	DTNA	4.64E-03	613.1	1429.7	2.33
**1381991**	GLIS2	5.72E-03	3.45	7.94	2.30
**1644202**	IRF7	8.18E-03	10.04	23.36	2.33
**1396987**	MARVELD2	8.94E-03	5.6	13.05	2.33
**1387534**	GPR109B	9.77E-03	5.71	13.22	2.32
**1380920**	TEAD2	< 1e-07	122.02	278.64	2.28
**1379395**	COL18A1	1.00E-07	76.52	174.85	2.29
**1385531**	MLKL	1.86E-05	30.26	69.48	2.30

**1390508**	POLE	2.10E-05	34.69	79.68	2.30
**1395686**	ITPKB	5.36E-05	317.43	724.97	2.28
**1383421**	FGFRL1	1.24E-04	109.45	248.21	2.27
**1380400**	SFMBT2	2.01E-04	34.74	79.04	2.28
**1379335**	FCN3	3.22E-03	22.89	51.99	2.27
**1381357**	UBQLNL	4.45E-03	8.15	18.5	2.27
**1381779**	IGSF22	4.65E-03	2.6	5.94	2.28
**1380227**	TLR9	6.52E-03	6.92	15.55	2.25
**1388258**	TCL1A	7.02E-03	2.64	5.99	2.27
**1380021**	DAZ1	8.98E-03	2.61	5.89	2.26
**1395241**	CCDC102A	3.00E-07	39.12	86.95	2.22
**1379896**	SOLH	8.00E-07	20.1	44.32	2.20
**1380768**	ZMIZ2	4.00E-06	82.14	180.85	2.20
**1395167**	TCIRG1	1.35E-05	21.89	48.73	2.23
**1392315**	HSD3B7	2.81E-05	20.74	45.65	2.20
**1382594**	PLXDC2	5.60E-05	43.29	97.28	2.25
**1379079**	AEBP1	6.07E-05	98.17	216.48	2.21
**1383530**	CCL27	5.96E-04	4.8	10.59	2.21
**1389341**	KIR2DS5	1.16E-03	5.12	11.33	2.21
**1644234**	IL18BP	1.39E-03	53.67	119.07	2.22
**1379797**	FLJ14107	1.53E-03	9.58	21.31	2.22
**1646041**	BACE2	1.77E-03	11.33	25.18	2.22
**1382885**	LOC552891	3.30E-03	3.76	8.3	2.21
**1389616**	C1R	3.48E-03	35.55	79.23	2.23
**1390904**	MBD2	4.54E-03	3.06	6.76	2.21
**1391403**	C1orf87	4.88E-03	7.14	15.98	2.24
**1382921**	SYT8	5.32E-03	4.89	10.85	2.22
**1385941**	DNASE1L2	1.10E-06	24.87	53.76	2.16
**1380447**	PLEKHA4	3.20E-06	20.55	44.2	2.15
**1386370**	SBNO2	7.50E-06	12.46	26.98	2.17
**1378166**	FGF11	5.02E-05	37.89	83.15	2.19
**1395050**	C1orf64	6.96E-05	256.88	561.72	2.19
**1644115**	KLHL17	1.96E-04	38.2	82.21	2.15
**1644966**	CSAG3B	2.03E-04	4.56	9.9	2.17
**1390794**	ENG	2.06E-04	110.85	239	2.16
**1381157**	IRF7	3.16E-04	61.69	134.72	2.18
**1382787**	SERINC5	3.22E-04	9.95	21.78	2.19
**1378270**	POU4F1	4.18E-04	4.66	10.14	2.18
**1645196**	C9orf68	7.92E-04	9.32	20.06	2.15
**1378550**	TGM2	1.84E-03	143.91	315.46	2.19
**1379136**	IFITM3	2.36E-03	1843.1	4023.54	2.18
**1393448**	ITGA1	2.69E-03	13.42	28.96	2.16
**1384102**	SCN10A	3.25E-03	3.29	7.21	2.19
**1383897**	NQO1	3.64E-03	238.2	519.79	2.18
**1645486**	TSPYL3	4.40E-03	2.74	5.94	2.17
**1379868**	CDKN1A	4.56E-03	179.28	385.82	2.15
**1392963**	TGM1	4.95E-03	5.79	12.69	2.19
**1644463**	GGT1	5.11E-03	6.65	14.58	2.19
**1391781**	DEPDC1B	6.93E-03	3.87	8.47	2.19
**1393511**	TNFRSF6B	7.37E-03	118.31	257.81	2.18
**1644488**	GALNT1	7.79E-03	2.02	4.37	2.16
**1646053**	ATXN2L	2.00E-07	34.17	73.39	2.15
**1379715**	BGN	1.00E-06	1761.62	3770.64	2.14

**1393568**	TFPI	1.26E-05	18.51	39.66	2.14
**1378478**	SRGAP1	2.14E-05	55.51	118.87	2.14
**1395389**	ANKHD1-EIF4EBP3	2.38E-05	10.5	22.46	2.14
**1385157**	LGI4	3.28E-05	55.34	117.22	2.12
**1390353**	SLIT3	4.81E-05	35.7	76.41	2.14
**1393261**	TAGLN2	1.55E-04	226.72	478.9	2.11
**1383729**	TNS1	7.34E-04	24.31	51.49	2.12
**1381028**	MYO1F	8.86E-04	13.53	28.92	2.14
**1388404**	C1orf106	1.36E-03	16.82	35.97	2.14
**1383793**	HABP2	1.49E-03	3.95	8.37	2.12
**1382879**	NRM	1.71E-03	9.76	20.56	2.11
**1385651**	LY96	1.86E-03	148.56	318.85	2.15
**1389116**	ANGPTL4	3.74E-03	86.07	183.03	2.13
**1380921**	ARSE	8.33E-03	4	8.56	2.14
**1386335**	MST1R	9.76E-03	3.89	8.34	2.14
**1384843**	DPP9	6.90E-06	146.68	306.13	2.09
**1393062**	ELK1	7.00E-06	584.29	1213.57	2.08
**1379298**	ZBTB40	7.00E-06	107.17	223.1	2.08
**1379188**	TNFRSF1A	8.20E-06	180.64	376.05	2.08
**1381452**	MOV10	2.15E-05	47.26	98.42	2.08
**1393433**	EFNA1	7.43E-05	130.09	270.76	2.08
**1388481**	BST2	9.31E-05	128.29	268.92	2.10
**1382602**	SLC25A41	2.19E-04	10.08	21.06	2.09
**1381564**	DMWD	3.22E-04	93.15	193.29	2.08
**1386081**	RAPGEF3	3.37E-04	72.83	150.34	2.06
**1644588**	FGFR4	3.55E-04	13.81	28.67	2.08
**1395842**	CFB	4.51E-04	86.08	179.44	2.08
**1644843**	DLEC1	4.89E-04	14.75	30.76	2.09
**1381818**	SLCO4A1	5.28E-04	106.07	219.18	2.07
**1391905**	SMAD6	7.46E-04	158.15	331.2	2.09
**1390324**	GBGT1	9.46E-04	22.56	46.59	2.07
**1645355**	BRCA1	1.39E-03	6.58	13.68	2.08
**1394627**	ANG	1.82E-03	64.17	132.71	2.07
**1380559**	CXCL10	2.87E-03	17.28	36.38	2.11
**1378428**	ARHGEF5	3.52E-03	3.29	6.83	2.08
**1396874**	MMP25	5.38E-03	5.25	10.98	2.09
**1378781**	SHC1	< 1e-07	266.29	541.38	2.03
**1387433**	ZNF692	6.00E-07	174.25	356.03	2.04
**1395760**	AKAP8L	2.50E-06	157.86	323.35	2.05
**1385071**	KIF7	9.70E-06	24.51	49.98	2.04
**1646238**	AGBL5	1.81E-05	24.35	50.1	2.06
**1380910**	IKBKE	2.34E-05	15.89	32.43	2.04
**1378040**	DFNB31	2.41E-05	35.19	71.27	2.03
**1377236**	POLN	3.15E-05	36.51	73.99	2.03
**1387088**	ATPAF2	3.57E-05	35.69	73.07	2.05
**1643745**	MKNK2	4.02E-05	386.75	782.31	2.02
**1389813**	MIER2	4.94E-05	18.58	37.64	2.03
**1384628**	REP15	5.62E-05	22.62	46.49	2.06
**1644262**	HYAL1	9.15E-05	17.64	35.91	2.04
**1377253**	TAOK2	1.06E-04	106.9	218.75	2.05
**1379246**	CRYAB	1.76E-04	2437.64	4968.02	2.04
**1380093**	TM4SF18	3.87E-04	100.95	204.51	2.03
**1384566**	GALNTL2	4.98E-04	57.39	116.89	2.04

**1381220**	TDRD10	5.16E-04	11.43	23.55	2.06
**1385367**	GNG8	5.29E-04	15.85	32.16	2.03
**1394726**	LAT2	6.62E-04	36.73	74.59	2.03
**1385727**	SLC19A3	9.36E-04	39.79	81.78	2.06
**1393219**	RHBDF2	1.14E-03	117.82	241.83	2.05
**1383686**	C21orf58	2.45E-03	4.01	8.1	2.02
**1646067**	ATP6V0A4	9.81E-03	5.39	10.96	2.03
**1377496**	PLOD3	< 1e-07	222.42	445.96	2.01
**1394418**	MTHFR	6.00E-07	73.61	145.87	1.98
**1378636**	SEPN1	9.00E-07	280.06	559.52	2.00
**1382933**	ZNF609	2.10E-06	88.78	176.58	1.99
**1390479**	FAM124A	3.00E-06	13.12	26.32	2.01
**1646448**	RBM38	3.70E-06	48.15	95.37	1.98
**1381301**	PYGO2	6.80E-06	39.92	80.24	2.01
**1397318**	COL4A2	9.50E-06	34.44	69.21	2.01
**1380277**	TNFRSF10B	2.15E-05	37.71	75.36	2.00
**1382449**	DGCR14	6.00E-05	44.35	88.53	2.00
**1383727**	SEPT6	7.00E-05	12.68	25.22	1.99
**1377364**	IGFBP4	7.90E-05	117.46	234.42	2.00
**1382721**	FAM39DP	1.21E-04	182.56	365.22	2.00
**1392226**	RUNX3	1.28E-04	21.36	42.42	1.99
**1379247**	ID3	3.33E-04	399.85	802.04	2.01
**1396994**	HEYL	4.08E-04	81.11	161.92	2.00
**1382136**	C6orf201	1.02E-03	3.54	7.08	2.00
**1378380**	FAM124B	1.04E-03	25.42	51.2	2.01
**1378715**	CASP4	1.46E-03	20.45	40.85	2.00
**1644888**	DDX11	1.82E-03	11.45	22.77	1.99
**1389505**	TNFRSF1B	3.74E-03	26.12	52.37	2.00
**1386061**	IFITM1	4.11E-03	638.84	1274.48	1.99
**1383748**	PGAM2	6.30E-03	23.51	47.24	2.01
**1389563**	CNTN4	6.44E-03	3.75	7.48	1.99
**1381666**	SLC9A3R2	6.64E-03	12.04	23.94	1.99
**1644017**	LOC339240	6.79E-03	2.5	4.99	2.00
**1392422**	ADH1B	7.05E-03	6.76	13.49	2.00
**1388834**	CD300A	8.15E-03	28	56.04	2.00
**1643691**	MUC1	9.10E-03	12.57	25.21	2.01
**1386777**	STXBP2	9.97E-03	20.54	41.4	2.02
**1381850**	SNX33	1.00E-06	26.95	53.24	1.98
**1387430**	MYO1D	3.60E-06	36.44	70.8	1.94
**1394993**	ZC3HAV1	3.80E-06	153.01	297.99	1.95
**1380127**	TCF3	4.40E-06	44.13	87.13	1.97
**1385131**	PLEKHF1	6.70E-06	91.52	180.51	1.97
**1395553**	FZD2	1.24E-05	25	49.22	1.97
**1384998**	RPS6KA1	1.42E-05	55.14	107.6	1.95
**1381384**	C20orf117	3.07E-05	93.06	181.11	1.95
**1386926**	WSCD1	4.82E-05	50.18	98.51	1.96
**1396546**	TTC38	4.88E-05	38.94	76.76	1.97
**1395936**	FOXF2	9.38E-05	36.14	70.38	1.95
**1394218**	RFXDC2	1.09E-04	58.99	115.15	1.95
**1379252**	TM4SF1	1.18E-04	777.18	1524.6	1.96
**1394637**	CD34	1.34E-04	166.29	327.09	1.97
**1383884**	DISC1	1.68E-04	12.42	24.12	1.94
**1386636**	ISG20	1.95E-04	71.88	140.39	1.95

**1378559**	DNAJB6	2.13E-04	248.27	486.17	1.96
**1396385**	HYAL1	4.83E-04	62.83	123.66	1.97
**1388819**	ITPR3	4.91E-04	184.96	361.39	1.95
**1386240**	CABP4	5.76E-04	6.21	12.24	1.97
**1645255**	C1QTNF1	8.29E-04	99.68	193.99	1.95
**1385310**	ATP8B3	1.14E-03	9.62	18.91	1.97
**1392324**	ITGAL	1.66E-03	12.76	24.78	1.94
**1391916**	ATP4A	1.72E-03	18.89	36.93	1.96
**1383646**	SPI1	2.63E-03	31.07	60.66	1.95
**1379974**	PKHD1	3.06E-03	2.34	4.55	1.94
**1385925**	BPI	5.21E-03	5.96	11.59	1.94
**1381237**	PLEKHG2	5.89E-03	8.01	15.75	1.97
**1397389**	PNLIPRP1	6.41E-03	6.07	11.83	1.95
**1383209**	GBP1	7.60E-03	23.85	46.39	1.95
**1386865**	EBI3	9.32E-03	32.91	64.46	1.96
**1377491**	DUSP18	3.60E-06	91.05	175.36	1.93
**1390061**	RUNX1	8.70E-06	12.7	24.38	1.92
**1381436**	FAM176B	3.09E-05	18.55	35.33	1.90
**1384720**	VAT1	3.42E-05	96.77	187.36	1.94
**1379119**	GIMAP8	4.14E-05	58.42	111.28	1.90
**1388571**	GAB3	6.88E-05	9.42	18	1.91
**1385912**	MICB	9.41E-05	20.64	39.54	1.92
**1380422**	WASF2	1.30E-04	76.83	146.84	1.91
**1388827**	ANLN	1.98E-04	92.24	177.35	1.92
**1378862**	BEST3	5.80E-04	8.4	16.16	1.92
**1646197**	ALPK1	9.39E-04	21.37	41.21	1.93
**1646398**	RHBDF2	1.41E-03	60.4	115.46	1.91
**1391279**	TFPI2	1.49E-03	4.22	8.11	1.92
**1391802**	DNAH17	1.54E-03	19.26	36.84	1.91
**1395867**	C9orf61	1.64E-03	119.39	227.79	1.91
**1377816**	EDNRA	1.69E-03	35.77	68.14	1.90
**1388807**	UPP2	1.72E-03	38.52	73.87	1.92
**1386218**	GDAP2	1.81E-03	8.57	16.42	1.92
**1384695**	KCTD4	1.91E-03	11.08	21.16	1.91
**1383552**	ERBB2	1.96E-03	50.94	97.31	1.91
**1381744**	BATF	2.50E-03	11.85	22.75	1.92
**1390770**	EMILIN1	2.64E-03	12.37	23.77	1.92
**1384427**	VAMP8	3.15E-03	211.51	410.2	1.94
**1380048**	ACVRL1	3.59E-03	33.06	64.07	1.94
**1395504**	BOC	3.95E-03	8.62	16.56	1.92
**1395225**	ELF5	5.27E-03	3.52	6.79	1.93
**1396576**	ADAMTS1	5.89E-03	77.86	150.79	1.94
**1391123**	IL4R	5.89E-03	59.06	112.63	1.91
**1394960**	LIPG	6.19E-03	19.58	37.46	1.91
**1644951**	CT45-5	7.19E-03	5.57	10.79	1.94
**1389252**	CCDC105	7.25E-03	4.76	9.07	1.91
**1379654**	LIMK2	8.20E-03	13.02	25	1.92
**1380399**	IHH	8.69E-03	4.27	8.28	1.94
**1646400**	RGL3	9.63E-03	7.33	14.19	1.94
**1395928**	CCDC50	3.00E-07	177.54	332.16	1.87
**1390255**	HS1BP3	5.00E-07	47.04	89.33	1.90
**1388549**	JAG1	1.50E-06	109.33	204.37	1.87
**1394329**	UPF1	2.50E-06	189.18	357.91	1.89

**1377906**	ANO6	5.90E-06	88.04	164.82	1.87
**1392438**	ZFHX3	7.00E-06	83.52	158.98	1.90
**1385485**	MYO9B	1.45E-05	79.53	150.29	1.89
**1388231**	DTX2	1.60E-05	96.91	181.49	1.87
**1379886**	PALLD	1.74E-05	336.36	637.65	1.90
**1392253**	MLLT6	2.08E-05	365.55	688.93	1.88
**1645932**	SEPN1	2.24E-05	33.04	62.8	1.90
**1387224**	DOCK6	2.33E-05	42.66	81.12	1.90
**1379097**	SERTAD1	2.69E-05	185.59	352.36	1.90
**1382398**	REXO4	4.13E-05	183.1	343.8	1.88
**1392565**	LOC400464	5.74E-05	23.82	45.05	1.89
**1389878**	ZCCHC24	1.12E-04	430.67	819.72	1.90
**1396651**	M6PRBP1	1.15E-04	284.22	531.64	1.87
**1381128**	VWA1	1.20E-04	48.42	91.84	1.90
**1392956**	FAM39DP	1.89E-04	341.7	646.1	1.89
**1395457**	RECQL4	2.19E-04	14.76	27.61	1.87
**1394202**	GLI2	2.36E-04	13.79	26.03	1.89
**1377387**	PRRG2	3.05E-04	13.5	25.59	1.90
**1386453**	TRIP10	3.22E-04	16.85	31.99	1.90
**1381580**	LAMB2	3.85E-04	77.46	145.73	1.88
**1395761**	MAPKAPK2	4.54E-04	23.5	44.14	1.88
**1390786**	CALCRL	4.62E-04	25.9	49.3	1.90
**1384723**	MARCH6	5.08E-04	1262.24	2380.58	1.89
**1384160**	FLCN	5.09E-04	23.62	44.45	1.88
**1645096**	CDC2L2	5.34E-04	49.76	94.24	1.89
**1384359**	FLJ41649	5.35E-04	19.15	36.37	1.90
**1380155**	SCARA3	5.76E-04	54.6	103.98	1.90
**1390964**	TBC1D26	6.82E-04	17.29	32.39	1.87
**1390611**	TMEM140	7.26E-04	83.21	158.38	1.90
**1394085**	PLCD1	7.32E-04	89.46	169.28	1.89
**1378819**	RASL12	8.62E-04	134.43	252.52	1.88
**1384310**	ITGA10	9.36E-04	59.18	112.54	1.90
**1385756**	STARD8	1.01E-03	49.78	93.33	1.87
**1390294**	TBX2	1.13E-03	36.18	68.38	1.89
**1380982**	COLEC12	1.27E-03	191.5	363.12	1.90
**1389735**	SPATA13	1.39E-03	30.38	57.28	1.89
**1382331**	CLIC1	3.08E-03	182	342.56	1.88
**1395773**	TJP3	4.36E-03	9.69	18.22	1.88
**1383893**	C5AR1	5.60E-03	14.62	27.62	1.89
**1386067**	TTK	7.10E-03	4.9	9.32	1.90
**1384845**	GLUL	8.06E-03	4.91	9.34	1.90
**1392086**	KIAA0913	4.00E-07	459.09	852.74	1.86
**1396184**	SLC2A4RG	6.00E-07	36.54	67.61	1.85
**1391647**	NEK6	8.00E-07	84.51	156.36	1.85
**1388811**	INPPL1	9.00E-07	342.95	636.46	1.86
**1394151**	C10orf85	2.60E-06	53.21	98.22	1.85
**1380406**	SLC26A6	4.70E-06	37.64	69.13	1.84
**1393290**	CIC	5.90E-06	142.98	264.2	1.85
**1381677**	GABRE	7.60E-06	19.32	35.62	1.84
**1385522**	ZDHHC18	1.44E-05	35.48	66.1	1.86
**1382389**	CLEC1A	1.78E-05	27.59	50.93	1.85
**1380712**	SART1	1.99E-05	41.7	77.73	1.86
**1390690**	S1PR3	2.01E-05	47.49	87.46	1.84

**1383945**	PROM2	3.27E-05	23.88	43.92	1.84
**1395210**	KIAA1754	3.55E-05	57.98	108	1.86
**1394733**	LYG1	4.91E-05	18.85	34.68	1.84
**1392713**	CNN3	7.02E-05	1809.66	3328.74	1.84
**1389288**	TNFRSF10A	1.12E-04	12.28	22.69	1.85
**1388998**	GPNMB	1.37E-04	127.76	235.7	1.84
**1383014**	MGC33556	1.56E-04	27.2	49.99	1.84
**1390276**	RASGRP3	2.05E-04	131.42	243.69	1.85
**1388842**	SLC9A9	2.54E-04	78.21	144.87	1.85
**1380244**	SAMD4B	4.46E-04	154.06	283.29	1.84
**1382345**	HIPK2	4.50E-04	482.67	900.53	1.87
**1380159**	AHDC1	5.28E-04	78.67	146.71	1.86
**1644470**	GEM	6.30E-04	27.19	50.65	1.86
**1388708**	GRRP1	8.91E-04	90.77	169.47	1.87
**1394645**	CACNA1A	9.79E-04	64.36	118.39	1.84
**1393382**	GPR146	1.44E-03	28.58	52.46	1.84
**1646921**	NTRK1	1.62E-03	3.03	5.65	1.86
**1387870**	HAS2	1.93E-03	5.88	10.98	1.87
**1388638**	SIX5	1.98E-03	62.36	115.68	1.86
**1384678**	RAB13	2.06E-03	32.52	59.69	1.84
**1391669**	SHROOM1	2.15E-03	22.38	41.59	1.86
**1379306**	IL17RB	2.73E-03	242.55	451.01	1.86
**1643954**	LOC613037	3.18E-03	1686.65	3133.12	1.86
**1395440**	RAD54L	5.17E-03	9.55	17.58	1.84
**1384175**	RESP18	5.36E-03	28.77	52.85	1.84
**1391364**	MAP3K8	6.31E-03	61.76	114.86	1.86
**1383492**	SLC6A12	7.11E-03	222.33	409.43	1.84
**1644420**	GPER	7.61E-03	234.18	434.15	1.85
**1381079**	LTB4R	7.68E-03	8.78	16.17	1.84
**1378357**	HIGD1B	9.30E-03	187.87	348.38	1.85
**1386588**	MLXIP	9.36E-03	5.89	11	1.87
**1645195**	CACNA1I	1.30E-06	737.31	1334.23	1.81
**1389047**	PLOD1	2.80E-06	561.5	1021.11	1.82
**1389241**	VAMP5	1.14E-05	363.06	665.26	1.83
**1646748**	PCDH18	1.74E-05	98.3	179.2	1.82
**1380315**	BCL6	2.10E-05	803.91	1450.93	1.80
**1392040**	CCNA2	2.17E-05	26.51	48.02	1.81
**1379023**	SLC4A2	2.59E-05	22.59	41.33	1.83
**1389815**	FAM80B	2.65E-05	53.14	96.68	1.82
**1646768**	PAPSS2	2.65E-05	109.22	199.94	1.83
**1395556**	MORC2	2.88E-05	83.43	151.64	1.82
**1382246**	WWP2	6.16E-05	6.91	12.62	1.83
**1393041**	TNFRSF25	6.77E-05	349.75	633.94	1.81
**1393657**	PCOLCE	9.41E-05	28.29	51.49	1.82
**1377609**	PTK7	9.90E-05	15.05	27.57	1.83
**1395468**	CENTD2	1.07E-04	19.39	35.28	1.82
**1385887**	SFTPC	1.11E-04	18.55	33.81	1.82
**1384689**	NFKB2	1.15E-04	10.92	19.79	1.81
**1392938**	HCP5	1.19E-04	108.93	198.47	1.82
**1381957**	LEF1	1.48E-04	80.73	147.23	1.82
**1396858**	CGNL1	1.51E-04	682.97	1235.83	1.81
**1388624**	C14orf139	1.94E-04	23.89	43.75	1.83
**1386271**	TBL1X	2.06E-04	286.13	519.07	1.81

**1385840**	KIAA0323	2.28E-04	59.21	108.45	1.83
**1390431**	BNIPL	2.79E-04	22.76	41.32	1.82
**1385817**	BAZ1A	4.40E-04	34.2	62.23	1.82
**1378265**	SPR	4.59E-04	69.34	125.74	1.81
**1388986**	DDR2	5.52E-04	60.23	110.14	1.83
**1395464**	FERMT3	6.04E-04	26.46	48.11	1.82
**1395741**	CDK2	6.70E-04	41.24	74.64	1.81
**1379931**	PLEK	7.19E-04	84.9	155.64	1.83
**1394207**	GSDMD	8.77E-04	87.66	159.1	1.81
**1377374**	TMEM63A	9.07E-04	62.94	113.85	1.81
**1644372**	HAP1	9.85E-04	9.05	16.33	1.80
**1387414**	PYGL	1.05E-03	42.78	77.61	1.81
**1379209**	C14orf151	1.09E-03	20	36.2	1.81
**1378025**	CLDN9	1.30E-03	27.75	50.37	1.82
**1391431**	C10orf116	1.49E-03	1310.04	2379.45	1.82
**1389444**	GATA2	2.23E-03	14.67	26.8	1.83
**1379488**	ITGA5	2.51E-03	44.9	82.16	1.83
**1388524**	BCCIP	2.64E-03	13.23	23.88	1.80
**1378389**	PECAM1	3.37E-03	126.21	229.78	1.82
**1395165**	NR2E3	3.38E-03	6.15	11.09	1.80
**1644247**	IFNW1	3.86E-03	3.69	6.73	1.82
**1381604**	LRRC32	4.27E-03	105.86	194.13	1.83
**1643683**	MVP	4.76E-03	31.4	56.62	1.80
**1388451**	APOLD1	6.79E-03	340.33	616	1.81
**1382306**	ORAI1	7.82E-03	16.52	30.04	1.82
**1393918**	PSCA	9.35E-03	6.65	12.01	1.81
**1384587**	P2RX7	4.00E-07	293.08	524.3	1.79
**1392542**	TRAF2	8.00E-07	38.14	68.29	1.79
**1644820**	DNAJB2	1.30E-06	1296.45	2295.82	1.77
**1393154**	CYLN2	1.80E-06	360.95	644.37	1.79
**1392954**	TAZ	4.50E-06	77.83	138.24	1.78
**1379666**	C10orf33	9.20E-06	45.24	80.78	1.79
**1381236**	CDH23	2.02E-05	11.77	21.12	1.79
**1378608**	ZNF264	2.27E-05	94.44	168.02	1.78
**1388868**	FAM125B	2.40E-05	304.01	545.1	1.79
**1392661**	STK36	3.30E-05	540.88	959.12	1.77
**1381884**	UNC5B	4.09E-05	37.59	66.56	1.77
**1396736**	INPP5D	4.66E-05	35	62.41	1.78
**1378461**	RFX1	4.87E-05	69.06	122.25	1.77
**1387508**	NXN	8.05E-05	81.59	145.41	1.78
**1384036**	GIT1	1.22E-04	186.38	331.96	1.78
**1380834**	RAI14	1.75E-04	123.8	222.21	1.79
**1384355**	GIMAP4	1.81E-04	300.73	541.53	1.80
**1396812**	NOD1	2.02E-04	25.12	44.95	1.79
**1395996**	B4GALNT4	2.17E-04	203.25	362.91	1.79
**1393061**	SERPING1	2.93E-04	62.01	110.75	1.79
**1387842**	C12orf34	3.57E-04	49.87	89.13	1.79
**1380846**	PDIA4	3.92E-04	16.26	29.1	1.79
**1389548**	DNAJB1	4.54E-04	434.88	778.77	1.79
**1385847**	CASP7	4.79E-04	39.74	71.38	1.80
**1393126**	LDLRAP1	6.91E-04	22.8	40.92	1.79
**1382967**	AHNAK	1.10E-03	421.07	745.45	1.77
**1395266**	SLC45A3	1.12E-03	42.85	76.37	1.78

**1396945**	MYO1C	1.43E-03	26.58	47.7	1.79
**1377931**	SNX31	2.16E-03	12	21.44	1.79
**1386388**	KIF1C	3.79E-03	26.9	48.17	1.79
**1646924**	NRG2	3.96E-03	20.37	36.08	1.77
**1382059**	ECHDC3	4.29E-03	36.27	64.4	1.78
**1645485**	TSPO	4.42E-03	37.92	68.1	1.80
**1393022**	NAALADL1	4.92E-03	13.2	23.44	1.78
**1383834**	CNTD2	6.28E-03	13.01	23.39	1.80
**1376940**	PTPN6	8.43E-03	39.62	70.28	1.77
**1387238**	GH1	8.82E-03	4.92	8.78	1.78
**1393105**	PCOLCE2	9.07E-03	25.53	45.44	1.78
**1387167**	CGN	5.00E-07	93.29	162.72	1.74
**1389621**	GSDMB	9.00E-07	120.17	212.59	1.77
**1395122**	NECAP2	7.20E-06	141.65	249.96	1.76
**1379312**	SLC25A34	1.62E-05	83.38	147.47	1.77
**1389051**	SLC9A5	2.31E-05	77.26	136.27	1.76
**1385409**	MC1R	3.23E-05	96.64	170.07	1.76
**1394860**	TTBK1	5.22E-05	53.52	94.63	1.77
**1381378**	CUZD1	5.66E-05	10.8	19.06	1.76
**1395503**	ATXN3	6.43E-05	127.34	221.88	1.74
**1396737**	MSN	7.71E-05	379.35	663.03	1.75
**1379227**	ATXN7L2	8.19E-05	82.38	143.61	1.74
**1644267**	HYDIN	8.44E-05	10.81	18.94	1.75
**1380073**	OLFML2B	9.50E-05	29.16	51.38	1.76
**1378734**	CDK2AP2	1.30E-04	73.55	129.65	1.76
**1389157**	SCAMP2	1.55E-04	50.42	88.78	1.76
**1394077**	FLJ37078	2.12E-04	169.87	296.06	1.74
**1396894**	UCP3	2.56E-04	13.37	23.52	1.76
**1643636**	NBPF20	2.70E-04	1834.4	3193.32	1.74
**1387894**	TTYH3	3.65E-04	212.12	370.59	1.75
**1395770**	NOTCH3	5.02E-04	106.78	186.29	1.74
**1394737**	KIAA0329	5.15E-04	30.95	54.4	1.76
**1394617**	CCDC88B	6.39E-04	14.2	25.1	1.77
**1379100**	CCDC81	7.86E-04	8.37	14.8	1.77
**1379376**	CEBPD	8.58E-04	658.1	1162.64	1.77
**1394129**	ACSBG2	8.85E-04	5.97	10.42	1.75
**1389506**	PTHR1	8.94E-04	287.8	509.18	1.77
**1387867**	TGFBR2	9.55E-04	97.29	169.68	1.74
**1396643**	EPHB4	1.08E-03	21.69	38.28	1.76
**1394974**	PMP2	1.08E-03	2531.77	4450.83	1.76
**1388666**	HIP1	1.38E-03	67.31	117.66	1.75
**1388902**	CLDN5	1.89E-03	744.2	1304.37	1.75
**1391167**	HIST1H2BD	2.08E-03	95.89	167.11	1.74
**1378264**	ABCD1	2.27E-03	8.56	15.05	1.76
**1396512**	PRKCH	2.78E-03	100.87	177.4	1.76
**1379142**	C9orf167	3.16E-03	9.68	16.97	1.75
**1390024**	PPIC	3.63E-03	38.45	67.61	1.76
**1394066**	LOC92017	3.81E-03	44.28	77.12	1.74
**1394107**	KLKB1	4.24E-03	32.42	56.81	1.75
**1394900**	MRPL4	4.91E-03	5.47	9.64	1.76
**1393121**	PRAM1	5.78E-03	18.1	31.87	1.76
**1395835**	KCNN3	7.19E-03	115.81	203.74	1.76
**1383355**	BBOX1	7.25E-03	151.64	264.73	1.75

**1645758**	SPI1	8.53E-03	13.76	24.24	1.76
**1379770**	OAS2	9.02E-03	50.19	87.79	1.75
**1395927**	PCDHGA10	9.20E-03	12.53	21.88	1.75
**1383577**	GNRH1	1.00E-06	44.3	77	1.74
**1377289**	RALGDS	1.70E-06	1256.18	2180.47	1.74
**1377982**	STK38	1.70E-06	118.79	205.36	1.73
**1389064**	PCTK3	6.60E-06	84.42	144.87	1.72
**1396595**	TMEM2	1.24E-05	184.48	317.58	1.72
**1381970**	GLTP	2.26E-05	1031.82	1791.99	1.74
**1389189**	NT5DC2	5.00E-05	99.48	172.56	1.73
**1394071**	FZD4	5.35E-05	177.01	303.29	1.71
**1379274**	KCNJ2	5.47E-05	156.74	268.36	1.71
**1387363**	PACS2	9.40E-05	94.38	163.26	1.73
**1387038**	MAP3K11	1.03E-04	70.24	121.29	1.73
**1385253**	GNA11	1.13E-04	599.69	1025.64	1.71
**1646904**	NUPR1	1.17E-04	204.47	351.52	1.72
**1391224**	SIPA1	1.29E-04	75.6	130.53	1.73
**1390449**	FYCO1	1.32E-04	73.3	125.36	1.71
**1393546**	FOXO4	1.53E-04	273.18	467.11	1.71
**1387491**	ANTXR2	1.63E-04	41.91	71.79	1.71
**1646510**	PTGS2	2.70E-04	241.1	417.63	1.73
**1386607**	SLC15A3	3.08E-04	165.65	285.7	1.72
**1378273**	PLAC9	5.44E-04	70.59	121.26	1.72
**1383282**	PARP14	6.76E-04	54.18	92.65	1.71
**1390587**	LYL1	7.01E-04	139.51	238.79	1.71
**1645866**	SLC30A4	7.21E-04	22.63	39.02	1.72
**1379053**	DFFA	7.54E-04	357.91	621.83	1.74
**1646036**	BANP	8.07E-04	29.23	50.37	1.72
**1388515**	LEPREL2	8.54E-04	33.38	58.01	1.74
**1385110**	PDGFRB	1.12E-03	373.22	645.69	1.73
**1379845**	PRB1	1.20E-03	8.15	14.17	1.74
**1391306**	C10orf11	1.27E-03	11.87	20.58	1.73
**1378516**	CHST3	1.66E-03	128.24	221.84	1.73
**1644936**	CUTL1	2.23E-03	185.27	319.62	1.73
**1385103**	MFNG	2.29E-03	50.73	87.47	1.72
**1393848**	DOCK5	2.44E-03	15.64	27.06	1.73
**1390157**	TMC6	2.73E-03	63.63	110.33	1.73
**1394619**	SLC38A10	2.77E-03	25.21	43.51	1.73
**1644767**	DYRK1B	3.45E-03	34.84	59.85	1.72
**1379297**	LGALS9	5.12E-03	19.43	33.72	1.74
**1388455**	HSD17B1	5.48E-03	15.44	26.65	1.73
**1393619**	SASH3	7.07E-03	29.44	50.38	1.71
**1393263**	ADCY6	2.70E-06	205.58	349.27	1.70
**1382335**	STAT3	4.40E-06	419.45	712.8	1.70
**1646626**	POL3S	5.10E-06	52.58	88.46	1.68
**1395287**	BARD1	8.40E-06	55.67	94.21	1.69
**1385032**	MKL1	9.50E-06	118.22	201.9	1.71
**1643662**	MYH9	1.44E-05	998.61	1680.61	1.68
**1391719**	RBM14	1.73E-05	747.24	1274.5	1.71
**1396002**	CELSR3	2.57E-05	579.77	985.2	1.70
**1383904**	CD247	2.59E-05	69.63	118.27	1.70
**1382232**	KCNJ8	2.71E-05	99.42	169.16	1.70
**1386911**	UNKL	3.12E-05	89.36	151.89	1.70

**1382778**	SERPINB6	3.22E-05	1238.1	2096.65	1.69
**1379475**	BMP1	3.59E-05	44.43	75.05	1.69
**1381529**	SH3BP5L	4.91E-05	226.19	385.65	1.70
**1390999**	DMPK	4.99E-05	40.08	67.76	1.69
**1390529**	VPS37B	5.69E-05	131.63	224.85	1.71
**1379993**	C1orf144	7.48E-05	245.75	418.9	1.70
**1378251**	MAPK7	1.14E-04	52.18	88.09	1.69
**1388746**	PHF19	1.16E-04	63.28	107.78	1.70
**1380051**	TLE2	1.25E-04	160.07	271.62	1.70
**1395624**	ZBTB46	1.61E-04	104.06	174.93	1.68
**1395462**	LAMA5	1.75E-04	673.07	1148.05	1.71
**1397032**	LOC63920	1.84E-04	73.78	125.53	1.70
**1382452**	ZDHHC8	2.28E-04	1159.24	1977.45	1.71
**1386923**	IQSEC2	2.71E-04	318.08	537.33	1.69
**1381240**	GSTM4	3.20E-04	56.84	96.34	1.69
**1397240**	STARD3	3.71E-04	44.92	75.84	1.69
**1378678**	TRIM5	3.97E-04	23.6	39.75	1.68
**1384962**	SEMA6C	4.90E-04	32.64	54.99	1.68
**1381204**	ABHD4	6.74E-04	30.29	51.73	1.71
**1391040**	LOC401233	6.74E-04	12.47	21.21	1.70
**1397366**	DDR1	7.73E-04	181.68	307.35	1.69
**1645202**	C9orf58	8.79E-04	161.87	274.97	1.70
**1390444**	FER1L3	8.80E-04	61.66	105.1	1.70
**1377576**	IGFBP7	9.48E-04	861.42	1450.34	1.68
**1379260**	ZNF397	9.50E-04	38.86	65.46	1.68
**1378267**	ABCC3	1.19E-03	14.66	25.03	1.71
**1392225**	GPR124	1.25E-03	56.68	96.23	1.70
**1385224**	PLEKHO2	1.49E-03	45.01	76.71	1.70
**1646547**	PRKX	1.82E-03	43.57	74.39	1.71
**1394448**	AQP1	1.92E-03	13.45	22.78	1.69
**1646418**	RFX2	1.96E-03	24.25	41.29	1.70
**1387415**	PLXNB1	1.99E-03	275.77	464.78	1.69
**1391747**	HAP1	2.49E-03	28.45	48.11	1.69
**1381062**	WHSC2	2.52E-03	35.55	60.43	1.70
**1645271**	C1orf152	3.12E-03	78.26	131.93	1.69
**1380532**	REXO1L1	3.25E-03	3.38	5.72	1.69
**1397387**	P2RY5	3.99E-03	96.22	162.94	1.69
**1382177**	MBTD1	5.12E-03	35.04	59.81	1.71
**1646624**	PODXL	5.38E-03	234.39	399.45	1.70
**1646838**	OR5D16	6.02E-03	8.99	15.35	1.71
**1377050**	FAM46B	6.54E-03	12.66	21.33	1.68
**1645836**	SLC7A5P1	8.37E-03	7.02	11.86	1.69
**1382810**	LSM11	8.72E-03	53.32	90.71	1.70
**1392665**	FSTL1	9.16E-03	88.35	149.33	1.69
**1379772**	HPS4	5.00E-07	45.56	75.55	1.66
**1388373**	FLNB	1.31E-05	225.61	376.8	1.67
**1395275**	HDAC1	1.67E-05	447.14	742.81	1.66
**1392957**	GLT25D1	1.67E-05	266.64	442.13	1.66
**1388861**	ZER1	2.22E-05	184.24	309.32	1.68
**1384945**	POLD1	2.75E-05	29.29	48.87	1.67
**1390268**	ARID3A	3.37E-05	100.4	168.36	1.68
**1382167**	EDC3	5.66E-05	81.24	134.35	1.65
**1387975**	SIX4	6.40E-05	86.35	143.34	1.66

**1385681**	SEMA4B	6.63E-05	119.41	197.57	1.65
**1378676**	MOBKL2C	9.07E-05	86.97	144.66	1.66
**1393743**	PPARD	1.10E-04	70.21	116.39	1.66
**1386710**	EEF1D	1.19E-04	298.68	494.73	1.66
**1382187**	ZNF324	1.22E-04	196.06	328.57	1.68
**1391210**	TAP1	1.50E-04	342.1	573.53	1.68
**1385530**	GLIS3	2.23E-04	36.12	60.41	1.67
**1646449**	RBM39	2.24E-04	427.51	712.08	1.67
**1379582**	SIN3B	2.60E-04	206.91	342.74	1.66
**1379559**	ProSAPiP1	3.13E-04	726.5	1208.92	1.66
**1644673**	FAM129B	3.89E-04	22.35	37.01	1.66
**1393595**	C6orf59	3.91E-04	55.68	92.59	1.66
**1395970**	ABCB7	5.83E-04	77.71	129.85	1.67
**1644044**	LINCR	6.31E-04	18.63	30.9	1.66
**1396745**	BLM	7.35E-04	25.96	43.05	1.66
**1378314**	SEZ6	1.02E-03	163.6	272.05	1.66
**1392002**	NFKBIZ	1.09E-03	146.44	245.99	1.68
**1389242**	DDAH2	1.11E-03	30.67	51.14	1.67
**1376858**	FOXQ1	1.13E-03	202.51	336.32	1.66
**1387683**	FAM107B	1.24E-03	276.68	464.73	1.68
**1394181**	TRIOBP	1.27E-03	121.16	200.79	1.66
**1395401**	TSPO	1.32E-03	443.4	734.93	1.66
**1389498**	BOK	1.68E-03	97.21	161.35	1.66
**1382906**	SCARA3	1.69E-03	148.24	246.75	1.66
**1644445**	GNA12	1.82E-03	218.01	365.99	1.68
**1377611**	GNG11	2.19E-03	314.68	521.93	1.66
**1383450**	C20orf160	2.41E-03	84.46	141.9	1.68
**1377537**	RRBP1	2.50E-03	39.28	65.26	1.66
**1396935**	GALM	2.65E-03	38.16	63.12	1.65
**1377977**	SHC1	2.79E-03	10.06	16.82	1.67
**1387083**	NHLRC2	2.90E-03	17.42	29.24	1.68
**1387942**	MYOM1	3.00E-03	186.41	310.19	1.66
**1387438**	CLDN15	3.01E-03	9.31	15.45	1.66
**1384168**	PLCG2	3.19E-03	84.38	139.95	1.66
**1395806**	FBLN1	3.28E-03	304.22	506.57	1.67
**1644050**	LLGL2	3.34E-03	8.43	14.12	1.67
**1389019**	SYDE1	3.60E-03	25.91	43.32	1.67
**1388848**	MX2	4.95E-03	25.91	43.29	1.67
**1392392**	C16orf5	5.26E-03	76.09	127.4	1.67
**1645773**	SP100	5.39E-03	13.35	22.15	1.66
**1380208**	FAM167B	6.37E-03	10.54	17.54	1.66
**1381408**	MVP	6.60E-03	125.93	209.58	1.66
**1379113**	SLC7A2	6.99E-03	74.14	124.48	1.68
**1393840**	COL20A1	8.76E-03	69.89	117	1.67
**1381141**	ALPL	9.92E-03	511.35	854.75	1.67
**1379728**	NIPBL	3.70E-06	210.22	346.46	1.65
**1386077**	TICAM2	5.20E-06	101.3	165.69	1.64
**1388011**	CDC2L6	8.60E-06	827.37	1351.53	1.63
**1394602**	MTMR11	1.03E-05	141.34	231.75	1.64
**1644857**	DIAPH1	1.53E-05	306.88	501.61	1.63
**1378914**	LEPRE1	1.61E-05	57.19	93.86	1.64
**1389093**	PTBP1	1.95E-05	798.32	1314.58	1.65
**1379915**	STK11IP	2.06E-05	102.37	166.73	1.63

**1389075**	PHF2	2.92E-05	173.32	283.56	1.64
**1391461**	SH3PXD2A	3.51E-05	912	1491.33	1.64
**1385928**	RAVER1	4.47E-05	69.88	114.28	1.64
**1394184**	LAMC1	4.78E-05	338.95	552.17	1.63
**1395051**	ASB6	5.23E-05	134.55	221.36	1.65
**1378583**	DGKG	5.47E-05	65.7	108.43	1.65
**1393929**	TSHZ1	6.48E-05	377.54	615.79	1.63
**1377945**	TSEN54	7.66E-05	72.48	119.02	1.64
**1378148**	SYNJ2BP	1.06E-04	613.89	1014.62	1.65
**1387267**	L3MBTL	1.23E-04	16.5	27.19	1.65
**1377524**	P4HA2	1.36E-04	50.56	83.4	1.65
**1384457**	PPP1R16B	1.43E-04	956.4	1570.57	1.64
**1393348**	ANXA11	1.47E-04	21.84	35.91	1.64
**1381334**	CDK5RAP2	1.70E-04	178.21	291.15	1.63
**1388307**	MBD3	1.99E-04	96.63	159.29	1.65
**1393073**	MAPKBP1	2.31E-04	81.19	133.25	1.64
**1389210**	SLC7A9	2.38E-04	55.56	91.76	1.65
**1377807**	NEO1	2.43E-04	126.7	207.42	1.64
**1379090**	NPAS3	2.92E-04	187.36	305.22	1.63
**1644351**	HDAC10	3.20E-04	14.96	24.35	1.63
**1379514**	TTC14	3.40E-04	340.7	561.74	1.65
**1381486**	SEMA3F	4.02E-04	33.24	54.52	1.64
**1385018**	JUP	4.79E-04	55.15	89.9	1.63
**1376873**	FXYD5	5.95E-04	120.49	197.92	1.64
**1379866**	VARS2	6.08E-04	386.79	632.21	1.63
**1388916**	C9orf164	7.03E-04	215.97	355.42	1.65
**1395513**	ITGA2	7.15E-04	24.21	39.38	1.63
**1392496**	CPS1	8.61E-04	47.07	76.85	1.63
**1381526**	FAM38A	9.11E-04	172.75	284.15	1.64
**1389391**	RHPN2	9.91E-04	306.9	506.72	1.65
**1378577**	LRDD	9.95E-04	36.04	58.83	1.63
**1382265**	CXCL16	1.27E-03	308.62	505.26	1.64
**1395101**	RAMP3	1.31E-03	64.81	106.45	1.64
**1384850**	TRAM2	2.22E-03	29.34	48.22	1.64
**1378223**	ADA	2.40E-03	69.01	113.59	1.65
**1388632**	LRFN4	2.98E-03	79.44	129.52	1.63
**1380825**	IL15RA	3.19E-03	7.4	12.17	1.64
**1391199**	SAMD9	3.41E-03	26.3	43.3	1.65
**1389462**	PILRB	3.48E-03	163.34	266.58	1.63
**1386572**	SARM1	3.51E-03	86.39	141.55	1.64
**1379115**	TEAD4	3.67E-03	43.2	70.39	1.63
**1390830**	FAM107A	4.91E-03	9206.49	15152.85	1.65
**1392207**	MMP11	5.50E-03	8.43	13.86	1.64
**1386675**	MTHFD2	5.61E-03	39.32	64.4	1.64
**1379186**	ITGA11	5.67E-03	8.35	13.61	1.63
**1387243**	RHBDF2	5.88E-03	15.08	24.55	1.63
**1387797**	PTRF	7.50E-03	292.03	481.9	1.65
**1392657**	S100A10	7.51E-03	736.18	1199.45	1.63
**1386387**	GGTLC1	9.70E-03	11.49	18.92	1.65
**1393582**	TBC1D2B	3.00E-07	149.96	241.77	1.61
**1381453**	PHCA	1.60E-06	379.01	613.14	1.62
**1387202**	CTDSP2	1.90E-06	563.12	914.57	1.62
**1645116**	CD151	2.40E-06	1094.97	1766.85	1.61

**1387264**	RPAP1	2.60E-06	68.65	110.82	1.61
**1389471**	LMAN2L	5.20E-06	148.17	240.78	1.63
**1396067**	CRYGS	8.40E-06	76.72	123.04	1.60
**1392172**	FAM48A	8.70E-06	68.32	110.8	1.62
**1380597**	PCNX	1.05E-05	173.95	281.04	1.62
**1385115**	SYNE2	1.17E-05	21.19	34.42	1.62
**1387236**	UBXN2A	1.48E-05	153.85	249	1.62
**1392897**	ASPRV1	1.78E-05	36.63	59.32	1.62
**1378720**	TEF	2.14E-05	604.01	977.9	1.62
**1378574**	DENND2A	6.04E-05	221.07	354.68	1.60
**1392912**	SAP30BP	6.09E-05	92.04	147.82	1.61
**1380401**	PRR5	6.15E-05	94.27	153.25	1.63
**1643834**	MAP2K3	7.26E-05	70.73	113.2	1.60
**1378790**	ATN1	7.67E-05	110.9	177.48	1.60
**1379200**	RXRA	7.70E-05	726.36	1174.21	1.62
**1391318**	BRPF1	9.93E-05	62.62	100.24	1.60
**1385976**	SETD1A	1.23E-04	164.19	264.85	1.61
**1392568**	FARP1	1.34E-04	603.77	975.34	1.62
**1388751**	OGDH	1.45E-04	240.83	390.58	1.62
**1388038**	IKZF4	1.67E-04	68.09	109.61	1.61
**1391879**	ATG16L2	1.85E-04	85.17	136.71	1.61
**1378692**	MGAT1	2.12E-04	318.03	514.24	1.62
**1390627**	C15orf39	2.22E-04	19.96	32.2	1.61
**1380604**	VASH1	2.54E-04	192.09	308.68	1.61
**1382861**	PTTG1IP	2.60E-04	1291.82	2073.22	1.60
**1395331**	SFRS16	3.15E-04	31.51	51.01	1.62
**1381872**	KHSRP	3.16E-04	343.29	556.77	1.62
**1394409**	SLC25A29	3.24E-04	128.64	206.03	1.60
**1385814**	ROBO3	3.79E-04	99.39	159.45	1.60
**1376927**	PARP10	4.48E-04	26.7	43.08	1.61
**1646777**	PALM	4.49E-04	247.84	402.78	1.63
**1644189**	ITGB5	4.62E-04	492.07	788.5	1.60
**1392446**	MYB	4.81E-04	21.8	34.89	1.60
**1378936**	SESN2	5.14E-04	23.71	38.45	1.62
**1385484**	EIF2C2	5.35E-04	517.96	837.41	1.62
**1383996**	TAOK1	5.79E-04	134.29	217.6	1.62
**1396586**	NDUFA4L2	7.59E-04	148.2	238.48	1.61
**1392620**	CEP164	8.96E-04	67.41	108.59	1.61
**1376933**	DNM2	1.06E-03	70.25	112.67	1.60
**1386212**	VPS13C	1.09E-03	81.68	132.14	1.62
**1379748**	KIF15	1.15E-03	19.83	32.11	1.62
**1377874**	FLJ20920	1.16E-03	15.86	25.68	1.62
**1380798**	ITGB1	1.31E-03	799.13	1298.67	1.63
**1396291**	FBXL20	1.36E-03	140.07	224.62	1.60
**1380882**	MYH7	1.67E-03	57.27	91.93	1.61
**1389577**	COL27A1	1.78E-03	14.77	23.77	1.61
**1379010**	INHBB	2.00E-03	35.85	57.9	1.62
**1645511**	TRIM5	2.77E-03	13.64	21.87	1.60
**1643814**	MARCH3	2.78E-03	58.17	94.22	1.62
**1380045**	GRAP	2.82E-03	36.96	60.05	1.62
**1377947**	APOL3	3.25E-03	58.22	94.55	1.62
**1382147**	EXPH5	3.59E-03	30.36	48.69	1.60
**1388510**	C10orf90	4.32E-03	26.73	43.35	1.62

**1379375**	ANKRD13B	4.56E-03	26.7	42.87	1.61
**1396125**	CD22	4.56E-03	15.5	25.08	1.62
**1644073**	LAT2	4.79E-03	21.36	34.53	1.62
**1646026**	BCL2L12	4.86E-03	26	41.98	1.61
**1388150**	PROCR	5.48E-03	22.57	36.14	1.60
**1387956**	HERC5	5.58E-03	151.97	246.58	1.62
**1396985**	PLSCR4	5.62E-03	216.13	350.24	1.62
**1383434**	HIST2H2BE	5.71E-03	97.41	157.91	1.62
**1382166**	RPN2	5.78E-03	35.93	57.99	1.61
**1381876**	PEX6	6.80E-03	78.09	126.47	1.62
**1397246**	FLJ43806	6.86E-03	19	30.73	1.62
**1391335**	C5orf39	7.99E-03	38.31	61.97	1.62
**1385837**	C20orf94	8.09E-03	57.79	93.31	1.61
**1377717**	FTCD	8.65E-03	74.63	120.34	1.61
**1386718**	FOXC1	9.65E-03	560.92	910.07	1.62
**1387693**	HIC2	2.00E-06	186.21	295.97	1.59
**1389826**	RBCK1	8.30E-06	135.48	215.49	1.59
**1385406**	TOP3B	1.79E-05	146.39	232.99	1.59
**1378076**	RGS11	2.34E-05	536.7	852.82	1.59
**1380539**	C16orf79	2.68E-05	108.71	171.53	1.58
**1644679**	FAM110A	2.77E-05	15.61	24.59	1.58
**1383584**	MLL4	3.72E-05	100.9	160.29	1.59
**1383016**	TMCC2	4.53E-05	124.38	197.92	1.59
**1385706**	NUMA1	4.80E-05	296.49	467.61	1.58
**1394599**	HMG20B	4.93E-05	256.22	404.79	1.58
**1394015**	ZNF137	5.12E-05	32.05	50.96	1.59
**1394959**	U2AF1L2	5.58E-05	348.07	549.8	1.58
**1392681**	BIN3	6.89E-05	32.31	50.95	1.58
**1390108**	LZTR1	7.15E-05	699.6	1109.73	1.59
**1383522**	USP37	8.84E-05	74.08	117.77	1.59
**1397226**	ZNF175	9.10E-05	100.87	159.59	1.58
**1383921**	MAPK8IP1	9.73E-05	304.51	483.92	1.59
**1380295**	AKNA	9.79E-05	63.06	100.55	1.59
**1644152**	KIAA0562	9.97E-05	72.03	114.57	1.59
**1394196**	CMIP	1.05E-04	1191.96	1892.2	1.59
**1388845**	KLHL36	1.61E-04	65.88	104.15	1.58
**1394831**	VANGL2	2.14E-04	70.38	110.86	1.58
**1384527**	MXD4	2.23E-04	1339.9	2113.31	1.58
**1378845**	C19orf25	2.48E-04	62.42	99.46	1.59
**1392582**	SHB	3.24E-04	44.19	69.94	1.58
**1395135**	FSTL3	3.43E-04	64.16	101.34	1.58
**1387600**	NADSYN1	3.60E-04	50.34	80.39	1.60
**1377845**	STK32B	5.07E-04	47.33	75.38	1.59
**1393003**	CROCC	5.24E-04	46.88	74.07	1.58
**1389808**	CDC42BPB	5.67E-04	320.44	512.07	1.60
**1377154**	DHX34	5.87E-04	78.74	125.11	1.59
**1386999**	PLXNA3	6.20E-04	75.95	121.04	1.59
**1393941**	SLC25A37	6.65E-04	261.11	411.95	1.58
**1395636**	ISG20L1	6.97E-04	90.05	142.98	1.59
**1384640**	PTCHD1	7.45E-04	230.67	363.29	1.57
**1390721**	BCORL1	9.19E-04	25.33	40.28	1.59
**1385073**	C1QTNF6	9.77E-04	15.2	24.03	1.58
**1392130**	LTA	1.03E-03	14.43	23.08	1.60

**1377995**	SLC6A9	1.30E-03	86.25	137.16	1.59
**1392606**	COX19	1.47E-03	291.28	461.65	1.58
**1385392**	FAM111A	1.59E-03	55.74	88.4	1.59
**1388015**	PIM1	1.65E-03	120.18	191.81	1.60
**1394633**	ESAM	1.66E-03	248.71	395.88	1.59
**1394328**	CD151	1.70E-03	30.47	48.3	1.59
**1383148**	SHF	1.99E-03	21.79	34.73	1.59
**1384006**	ZDHHC12	2.37E-03	18.15	28.61	1.58
**1378421**	LITAF	2.51E-03	360.34	574.02	1.59
**1384509**	RPRM	2.69E-03	334.61	528.1	1.58
**1382904**	CARHSP1	2.82E-03	350.66	556.05	1.59
**1384836**	DIP2A	3.56E-03	17.98	28.61	1.59
**1385315**	MAMDC4	3.97E-03	20.75	32.81	1.58
**1384335**	CCDC134	4.10E-03	8.1	12.9	1.59
**1385133**	RFTN2	4.83E-03	585.66	926.57	1.58
**1393246**	MTMR3	5.09E-03	36.87	58.47	1.59
**1383020**	TP53INP2	5.87E-03	385.28	613.26	1.59
**1645639**	TCL1B	7.46E-03	18.22	28.77	1.58
**1380113**	P2RY6	9.49E-03	12.26	19.33	1.58
**1380603**	SLC5A11	9.59E-03	57.63	91.24	1.58
**1378998**	TNPO3	6.30E-06	334.38	520.3	1.56
**1390754**	UBQLN4	1.96E-05	1009.77	1573.12	1.56
**1380292**	GPT2	2.03E-05	1304.84	2046.86	1.57
**1388888**	DGKD	3.12E-05	14.57	22.88	1.57
**1396523**	ABCA2	4.66E-05	167.84	263.26	1.57
**1388398**	STARD10	5.15E-05	249.94	391.33	1.57
**1396396**	ARHGEF10	6.00E-05	383.46	595.99	1.55
**1389550**	MYST3	6.91E-05	359.84	566.21	1.57
**1380207**	FAM20C	8.22E-05	148.04	229.56	1.55
**1384882**	IL10RB	1.04E-04	242.09	378.79	1.56
**1385491**	GRIK5	1.45E-04	69.52	107.83	1.55
**1381947**	COG7	1.46E-04	53.05	83.23	1.57
**1379328**	FURIN	1.47E-04	42.51	66.66	1.57
**1384285**	CEBPA	1.47E-04	152.26	237.92	1.56
**1387271**	ZNF444	2.11E-04	125.29	195.13	1.56
**1391739**	RIMS4	2.15E-04	226.51	356.54	1.57
**1383536**	DNHD1	2.44E-04	26.7	42.04	1.57
**1383446**	FLJ11783	2.48E-04	20.27	31.49	1.55
**1643887**	LRCH4	2.49E-04	81.89	128.4	1.57
**1380579**	CYP21A2	2.84E-04	20.73	32.61	1.57
**1393253**	TRIM14	2.88E-04	11.89	18.63	1.57
**1381820**	ACTL6A	3.05E-04	107.54	168.9	1.57
**1380938**	TAS2R10	3.22E-04	25.11	39.31	1.57
**1388897**	PHKA2	3.72E-04	151.12	237.6	1.57
**1393818**	SNX6	4.18E-04	143.79	225.19	1.57
**1388298**	FERMT2	4.37E-04	952.57	1480.54	1.55
**1387458**	ACTN4	4.51E-04	331.71	520.78	1.57
**1389449**	BAZ2B	5.55E-04	370.49	577.6	1.56
**1386550**	ST5	5.58E-04	54.15	84.83	1.57
**1393087**	FBXO27	5.65E-04	22.47	35.14	1.56
**1388712**	TTC23	6.32E-04	50.79	79.75	1.57
**1387959**	SOX12	6.38E-04	45.4	70.9	1.56
**1390513**	PHYHIP	7.25E-04	362.84	565.49	1.56

**1390125**	MICAL1	7.62E-04	173.62	270.17	1.56
**1390662**	CNOT6L	7.71E-04	36.46	56.71	1.56
**1381727**	LPP	8.03E-04	429.31	675.32	1.57
**1396972**	STAT5A	8.36E-04	56.91	88.72	1.56
**1389246**	C9orf114	9.17E-04	230.91	359.28	1.56
**1378699**	PIP5K1C	9.41E-04	812.41	1275.01	1.57
**1645193**	CACNA1G	9.58E-04	8.83	13.78	1.56
**1381920**	PHF16	9.83E-04	77.31	120.4	1.56
**1395663**	LMNA	1.02E-03	93.39	146.65	1.57
**1646519**	PSMB8	1.03E-03	76.72	120.71	1.57
**1393599**	PARP4	1.04E-03	189.69	297.73	1.57
**1386760**	KDELC2	1.05E-03	58.87	92.19	1.57
**1392458**	TRIM21	1.19E-03	24.34	38.2	1.57
**1379815**	ATP5D	1.21E-03	192.12	298.53	1.55
**1390831**	TLN1	1.28E-03	110.9	173.44	1.56
**1378121**	CDC20	1.53E-03	53.4	83.25	1.56
**1391827**	BCOR	1.55E-03	11.45	17.95	1.57
**1378229**	DTNBP1	1.80E-03	18.06	28.03	1.55
**1378880**	CUBN	1.98E-03	13.96	21.8	1.56
**1387149**	C16orf55	2.05E-03	15.78	24.57	1.56
**1392811**	SERTAD3	2.10E-03	31.17	48.77	1.56
**1385051**	SLC6A8	2.13E-03	253.57	398.61	1.57
**1386581**	SH2B2	2.32E-03	15.54	24.27	1.56
**1382505**	CLDN15	2.39E-03	104.51	163	1.56
**1387409**	TBX3	2.54E-03	19.22	29.89	1.56
**1378828**	METTL7B	2.95E-03	36.27	56.56	1.56
**1384447**	LDLRAD3	3.15E-03	21.97	34.57	1.57
**1391538**	DIDO1	3.18E-03	98	152.12	1.55
**1381207**	LRP10	3.21E-03	77.29	120.35	1.56
**1378877**	NR2F1	3.46E-03	634.94	996.2	1.57
**1383699**	PARP12	3.55E-03	55.35	85.83	1.55
**1379240**	COX18	3.69E-03	15.25	24	1.57
**1396153**	PSMB8	3.79E-03	18.23	28.43	1.56
**1380440**	ADAT3	3.82E-03	13.55	21.21	1.57
**1646659**	PKD1	3.91E-03	290.64	454.19	1.56
**1377331**	C10orf81	3.97E-03	9.15	14.36	1.57
**1391738**	CLEC14A	4.32E-03	152.46	236.43	1.55
**1377817**	RIT1	5.05E-03	34.78	54.56	1.57
**1378910**	OR5T3	5.55E-03	6.2	9.75	1.57
**1377897**	CPM	5.62E-03	5.47	8.48	1.55
**1391374**	DOCK8	6.30E-03	18.52	29.14	1.57
**1377103**	RAPSN	7.45E-03	7.26	11.31	1.56
**1377972**	NFKBIB	7.60E-03	17.77	27.65	1.56
**1384236**	CACNA1G	8.32E-03	8.58	13.44	1.57
**1385588**	DYSF	8.72E-03	188.11	292.16	1.55
**1644376**	H19	9.02E-03	15.09	23.68	1.57
**1390646**	LGALS4	9.88E-03	8.65	13.55	1.57
**1382260**	TYK2	4.00E-06	612.71	946.5	1.54
**1380565**	SLC24A6	4.70E-06	141.51	216.66	1.53
**1388912**	NR2C2AP	1.04E-05	75.53	115.38	1.53
**1391312**	LASS1	1.18E-05	281.6	430.92	1.53
**1391311**	ZNF341	1.24E-05	94.09	144.53	1.54
**1379183**	ANXA11	1.55E-05	587.93	909.5	1.55

**1390228**	LLGL1	3.04E-05	522.91	808.09	1.55
**1394122**	ZCCHC14	3.51E-05	352.7	546.34	1.55
**1379134**	BAT2	5.23E-05	240.84	371.17	1.54
**1382968**	STIP1	6.58E-05	741.01	1147.18	1.55
**1388500**	CDC42BPA	6.82E-05	98.73	150.96	1.53
**1385481**	STK40	7.24E-05	328.29	503.23	1.53
**1390717**	CBLB	9.78E-05	115.37	176.17	1.53
**1382565**	NOTCH4	1.22E-04	122.42	189.71	1.55
**1393492**	SLC39A1	1.26E-04	239.15	367.32	1.54
**1380867**	OSBPL7	1.48E-04	95.15	145.35	1.53
**1383818**	PTDSS2	1.62E-04	138.01	211.59	1.53
**1395461**	GLIPR2	1.87E-04	315.99	485.16	1.54
**1384286**	BCAR1	2.01E-04	122.15	189.28	1.55
**1389779**	ST3GAL4	2.17E-04	72.96	112.05	1.54
**1389198**	FGD1	2.30E-04	117.64	181.3	1.54
**1381114**	RAB11FIP4	2.73E-04	234.06	362.79	1.55
**1385633**	TRABD	2.89E-04	464.97	711.5	1.53
**1397396**	ITSN1	4.06E-04	239.5	368.67	1.54
**1393020**	MAN2B1	4.15E-04	30.26	46.42	1.53
**1378852**	EMP3	6.03E-04	272.64	420.75	1.54
**1384374**	FAM109A	6.62E-04	28.88	44.24	1.53
**1379034**	MAML1	6.77E-04	42.21	65.11	1.54
**1388764**	PAPOLA	6.90E-04	381.52	584.4	1.53
**1378727**	RSAD1	7.02E-04	69.96	108.33	1.55
**1382522**	SDF2L1	7.18E-04	436.35	676.07	1.55
**1385929**	ELF1	7.32E-04	126.81	194.56	1.53
**1390969**	UGCGL1	9.23E-04	78.92	122.03	1.55
**1384619**	ANXA5	9.60E-04	644.36	987.54	1.53
**1383274**	CNTN2	1.14E-03	737.21	1125.57	1.53
**1388849**	RNF166	1.18E-03	20.79	32.15	1.55
**1643533**	YWHAE	1.41E-03	248.77	382.13	1.54
**1377481**	LENG8	1.47E-03	22.39	34.24	1.53
**1385003**	MBD6	1.49E-03	192.54	294.61	1.53
**1645721**	ST5	1.88E-03	72.16	111.27	1.54
**1644555**	FLJ45445	1.97E-03	12.37	18.9	1.53
**1385687**	BVES	1.98E-03	23.87	36.65	1.54
**1388576**	FAM43A	2.27E-03	273.29	420.12	1.54
**1377290**	TIMP4	2.32E-03	37.48	57.52	1.53
**1377404**	KIAA0649	2.72E-03	86.64	132.49	1.53
**1645192**	CACNA1A	3.09E-03	13.53	20.89	1.54
**1388595**	PREX1	3.32E-03	67.01	103.66	1.55
**1395407**	C17orf62	3.67E-03	161.35	248.58	1.54
**1378483**	ENTPD1	3.70E-03	36.52	56.57	1.55
**1396617**	ITIH4	3.95E-03	51.14	79.29	1.55
**1393650**	ANXA1	4.05E-03	199.62	306.04	1.53
**1379902**	PPFIBP2	4.61E-03	166.5	255.18	1.53
**1644439**	GNB4	4.63E-03	24.03	37.06	1.54
**1384728**	C1orf198	4.82E-03	274.67	422.65	1.54
**1390266**	GATS	4.95E-03	888.13	1363.29	1.54
**1644864**	DENR	4.98E-03	469.19	724.21	1.54
**1390204**	SMTN	5.09E-03	98.15	150.45	1.53
**1394372**	TMEM156	5.16E-03	34.3	52.89	1.54
**1396167**	TRIM56	5.32E-03	34.16	52.4	1.53

**1379707**	TCN2	5.69E-03	65.17	100.96	1.55
**1388964**	KCNQ2	6.12E-03	44.91	68.92	1.53
**1393698**	EHD4	6.20E-03	64.65	99.71	1.54
**1644423**	GPNMB	6.65E-03	68.38	105.79	1.55
**1646579**	PRB1	6.75E-03	13.84	21.25	1.54
**1381761**	TPCN1	7.27E-03	16.62	25.61	1.54
**1645012**	CMTM3	8.89E-03	28.58	43.75	1.53
**1395110**	SOX10	9.13E-03	194.63	298.71	1.53
**1396159**	FOXF1	9.17E-03	18.42	28.43	1.54
**1391366**	SLC1A3	9.35E-03	3935.85	6058.89	1.54
**1381320**	SPEN	2.00E-06	757.11	1151.68	1.52
**1389525**	SHMT2	7.60E-06	352.65	530.94	1.51
**1383973**	PHF1	1.41E-05	156.18	236.38	1.51
**1382062**	PLEKHM1	2.04E-05	93.58	142.25	1.52
**1381601**	PPFIA4	2.65E-05	755.26	1140.87	1.51
**1645118**	CD276	3.10E-05	76.55	115.98	1.52
**1380813**	APBA3	3.35E-05	129.81	197.65	1.52
**1646085**	ATG12	3.74E-05	869.5	1324.76	1.52
**1388179**	ACADVL	3.84E-05	1469.81	2217.24	1.51
**1391708**	WDR6	4.91E-05	974.15	1477.69	1.52
**1385932**	LMNB2	6.78E-05	261.33	397.28	1.52
**1645371**	VPS13B	7.28E-05	99.23	150.72	1.52
**1394978**	ANKZF1	8.03E-05	51.64	78.82	1.53
**1380185**	NDST1	1.01E-04	109.25	164.58	1.51
**1392642**	CDK10	1.02E-04	899.28	1372.14	1.53
**1395722**	KIF13B	1.02E-04	166.53	253.98	1.53
**1377251**	FAM89B	1.08E-04	513.28	776.16	1.51
**1391546**	ZNF621	1.16E-04	134.62	203.09	1.51
**1390030**	BSDC1	1.24E-04	598.14	911.54	1.52
**1377732**	CDC2L5	1.29E-04	163.04	247.61	1.52
**1646231**	AHCTF1	1.73E-04	95.57	145.09	1.52
**1380873**	APC2	1.90E-04	213.04	324.63	1.52
**1392150**	SMAD3	2.45E-04	322.28	487.12	1.51
**1391579**	SFRS14	2.82E-04	154.5	235.65	1.53
**1393030**	NFIB	3.09E-04	925.11	1407.92	1.52
**1386237**	PLEC1	3.40E-04	95.61	145.47	1.52
**1395935**	KIAA1522	3.71E-04	107.64	162.02	1.51
**1396040**	ZNF827	4.49E-04	308.77	465.91	1.51
**1387039**	IFNAR2	4.49E-04	126.96	192.11	1.51
**1389664**	FCHO1	4.72E-04	73.37	110.42	1.50
**1383474**	IFIT2	5.37E-04	200.07	302.29	1.51
**1646006**	RRBP1	5.38E-04	193.69	294.77	1.52
**1643660**	N6AMT1	5.51E-04	100.84	153.8	1.53
**1393191**	PLOD2	6.54E-04	205.51	311.82	1.52
**1643561**	WHDC1	6.75E-04	32.62	49.38	1.51
**1645871**	SLC25A29	6.95E-04	89.1	134.21	1.51
**1381958**	RPS19	6.96E-04	4136.31	6266.27	1.51
**1395254**	ITFG3	8.45E-04	154.21	233.8	1.52
**1389433**	HDAC8	1.03E-03	71.66	109.25	1.52
**1384682**	SLC29A2	1.17E-03	60.72	92.28	1.52
**1387795**	NY-SAR-48	1.19E-03	17.42	26.39	1.51
**1386524**	SLC22A14	1.24E-03	18.23	27.56	1.51
**1396752**	JPH4	1.66E-03	571.96	861.13	1.51

**1643536**	ZBTB16	1.72E-03	350.76	530.52	1.51
**1389190**	CYP27A1	1.74E-03	180.24	272.84	1.51
**1390009**	CSF1	1.76E-03	9.09	13.86	1.52
**1383880**	RHOC	1.95E-03	431.95	656.39	1.52
**1394161**	ALMS1	2.04E-03	67.41	101.96	1.51
**1394989**	NKIRAS2	2.16E-03	21.35	32.53	1.52
**1397064**	CXXC5	2.16E-03	1227.4	1864.19	1.52
**1393842**	CCND1	2.53E-03	459.36	697.65	1.52
**1381110**	ZNF771	2.86E-03	34.22	51.88	1.52
**1389687**	DDN	2.87E-03	2860.49	4351.33	1.52
**1644576**	FLJ10324	3.03E-03	26.54	40.01	1.51
**1382827**	LASS4	3.17E-03	41.13	62.45	1.52
**1396806**	FTL	3.28E-03	9642.4	14518.79	1.51
**1396143**	PKP4	3.29E-03	676.18	1018.27	1.51
**1394881**	MAP1A	3.39E-03	2839.9	4284.05	1.51
**1388607**	LTBP4	3.50E-03	62.22	94.4	1.52
**1391156**	TJP1	3.56E-03	749.15	1134.43	1.51
**1397313**	NPIP	4.00E-03	257.79	392.27	1.52
**1384592**	C16orf74	4.04E-03	7.59	11.54	1.52
**1394907**	TMEM79	4.49E-03	96.99	146.69	1.51
**1395378**	PDCD4	4.67E-03	779.33	1172.91	1.51
**1379509**	FGD3	4.97E-03	51.41	78.24	1.52
**1384090**	CRTC1	5.93E-03	48.27	73.55	1.52
**1646580**	PRB3	6.77E-03	24.09	36.55	1.52
**1644438**	GOLPH4	7.95E-03	228.38	346.78	1.52
**1387408**	TGFBR3	8.65E-03	229.51	346.02	1.51
**1394109**	SH3BP4	8.99E-03	137.1	208.33	1.52
**1387931**	UBA7	9.29E-03	74.81	113.47	1.52
**1390269**	TNPO1	3.50E-06	174.51	262.35	1.50
**1387117**	PLAGL2	5.00E-06	93.77	140.69	1.50
**1381521**	KIAA0922	1.55E-04	32.24	48.47	1.50
**1380012**	TJAP1	1.84E-04	572.08	860.11	1.50
**1389034**	HELZ	2.09E-04	281.32	422.44	1.50
**1379250**	SRC	2.21E-04	154.37	231.61	1.50
**1388183**	TRIM25	2.21E-04	91.25	136.88	1.50
**1385438**	GAA	3.05E-04	58.42	87.63	1.50
**1388198**	SAPS1	6.05E-04	122.57	184.14	1.50
**1385512**	ZNF524	6.41E-04	119.05	179.02	1.50
**1390941**	KIFC2	9.99E-04	550.41	825.98	1.50
**1646756**	PC-3	1.03E-03	15.1	22.68	1.50
**1646713**	PDE9A	2.62E-03	166.37	249.96	1.50
**1644342**	HGF	5.75E-03	14.07	21.16	1.50
**1384379**	STK3	6.96E-03	124.96	187.5	1.50
**1644672**	FAM127C	8.48E-03	14.05	21.13	1.50
**1384366**	MORF4L1	1.67E-04	22.81	1.49	0.07
**1385806**	PHOSPHO2	3.16E-03	7.35	0.81	0.11
**1384368**	CPA6	8.22E-05	3.18	0.38	0.12
**1388248**	DKFZp686D0972	8.13E-03	5.67	0.82	0.14
**1645670**	TAC1	2.08E-05	176.79	29.85	0.17
**1391148**	SST	1.54E-04	630.88	107.57	0.17
**1645227**	C3orf57	2.57E-03	28.79	5.48	0.19
**1382946**	NMU	5.28E-05	18.96	3.77	0.20
**1378411**	OPRL1	9.47E-03	9.4	1.91	0.20

**1383406**	MAP1B	6.97E-05	1373.75	304.45	0.22
**1397198**	USMG5	3.65E-03	97.22	22.68	0.23
**1381672**	RTN1	4.12E-04	2435.69	570.79	0.23
**1388744**	RTN3	9.60E-06	168.34	40.23	0.24
**1646503**	PTPN20A	2.28E-04	111.87	27.21	0.24
**1388130**	USP12	3.68E-04	12.72	3.17	0.25
**1378185**	TPD52	9.64E-04	37.74	9.41	0.25
**1396101**	MIP	6.51E-03	5.44	1.38	0.25
**1379872**	SNX14	4.07E-03	5.11	1.35	0.26
**1393284**	GDAP1	8.00E-07	99.63	26.7	0.27
**1384214**	HTR5A	5.10E-06	37.42	10.46	0.28
**1387834**	OR6C1	2.58E-03	4.26	1.19	0.28
**1388790**	C4orf28	9.32E-03	6.12	1.72	0.28
**1392346**	SLC25A40	< 1e-07	105.55	29.7	0.28
**1646555**	PRKACB	7.00E-07	193.9	55.41	0.29
**1394850**	PPP2R2B	1.50E-03	11.43	3.31	0.29
**1386676**	GOLT1A	5.22E-05	46.49	13.6	0.29
**1386122**	ZDHHC13	6.21E-03	10.74	3.16	0.29
**1383314**	RRAGB	8.00E-07	43.17	12.84	0.30
**1376915**	PENK	1.52E-04	121.14	36.13	0.30
**1384533**	HK1	4.34E-04	40.18	12.17	0.30
**1391600**	PVALB	1.14E-03	1132.23	344.93	0.30
**1384482**	FBXO9	4.00E-06	97.7	29.83	0.31
**1390261**	SLC25A3	1.02E-03	64.31	19.66	0.31
**1396358**	TRIM37	5.07E-05	230.18	70.89	0.31
**1384817**	TRUB1	9.70E-06	71.86	22.4	0.31
**1397393**	NETO1	5.65E-05	28.35	8.95	0.32
**1394203**	ELMO1	1.01E-05	193.36	61.19	0.32
**1395139**	SLC10A4	8.48E-04	21.97	6.99	0.32
**1386633**	MAL2	1.06E-03	817.7	260.48	0.32
**1389485**	BTBD1	2.20E-05	196.98	63.17	0.32
**1377453**	CRYM	2.16E-04	349.26	113.46	0.32
**1394514**	CDC42	4.30E-06	108.98	35.51	0.33
**1393606**	SNX12	5.89E-05	38.02	12.38	0.33
**1387902**	RAB12	7.42E-04	35.93	11.74	0.33
**1395300**	PRKAR1B	1.84E-04	380.37	125.44	0.33
**1388064**	ATP6V1H	3.58E-05	38.31	12.66	0.33
**1392696**	FGF22	5.82E-03	11.94	3.96	0.33
**1381675**	AMACR	4.37E-05	88.35	29.38	0.33
**1378151**	KCNC2	5.01E-05	109.02	36.25	0.33
**1378710**	BAAT	1.25E-03	21.92	7.29	0.33
**1387095**	SLC32A1	3.19E-04	474.27	158.78	0.33
**1380538**	NOMO3	1.23E-04	46.09	15.47	0.34
**1645798**	SNAP25	3.42E-04	5513.37	1855.03	0.34
**1393503**	DYNC2LI1	5.10E-06	270.2	91.75	0.34
**1393471**	AK5	1.11E-03	67.2	23	0.34
**1385252**	ELAVL2	3.86E-05	189.21	65.03	0.34
**1393118**	PNOC	3.06E-04	67.12	23.17	0.35
**1644421**	GPM6A	1.15E-04	2099.93	728.25	0.35
**1385169**	EXDL1	4.33E-03	5.2	1.8	0.35
**1645135**	CCNL2	7.70E-05	85.56	29.87	0.35
**1389580**	MAT2B	8.78E-05	30.7	10.74	0.35
**1391313**	DLGAP1	1.05E-04	43.46	15.21	0.35

**1396916**	MME	6.54E-03	6.58	2.3	0.35
**1644521**	FRMPD2L2	1.41E-03	27.64	9.71	0.35
**1383238**	ZNF596	7.54E-03	7.92	2.78	0.35
**1390880**	FBXW7	2.09E-05	212.86	74.83	0.35
**1396744**	SYCE1	2.16E-04	24.69	8.75	0.35
**1394580**	KCNIP4	2.01E-04	350.34	124.51	0.36
**1386448**	NEUROD6	4.59E-04	190.2	68.51	0.36
**1397377**	KRT74	9.31E-03	4.16	1.5	0.36
**1643625**	NCKAP1	1.87E-05	203.26	73.65	0.36
**1383057**	SCAMP1	2.50E-06	138.04	50.18	0.36
**1388812**	RTN3	3.58E-05	95.31	34.67	0.36
**1390709**	FGF13	2.45E-04	27.77	10.1	0.36
**1384151**	CHURC1	9.53E-05	589.16	215.04	0.36
**1645380**	VIP	3.92E-04	279.09	101.78	0.36
**1383377**	RBM11	5.16E-04	50.09	18.3	0.37
**1380389**	RPL41	4.10E-06	63.86	23.48	0.37
**1644493**	GABRG2	3.50E-03	940.4	347.91	0.37
**1382774**	RWDD1	< 1e-07	194.41	72.38	0.37
**1644519**	FRMPD2L1	4.03E-03	22.54	8.38	0.37
**1394136**	EEF1B2	3.50E-06	139.82	52.14	0.37
**1391061**	MKKS	1.46E-04	47.09	17.6	0.37
**1644753**	EFHB	8.97E-04	26.39	9.83	0.37
**1380928**	ZNF557	1.64E-03	4.46	1.67	0.37
**1396211**	ENSA	6.00E-07	325.25	121.65	0.37
**1380193**	GABRA1	6.21E-04	490.59	183.86	0.37
**1396029**	RFC3	1.90E-06	16.73	6.28	0.38
**1380644**	CNTN1	2.75E-05	55.67	20.9	0.38
**1644104**	KPNA2	4.61E-04	63.14	23.74	0.38
**1397134**	BEX5	1.05E-04	1916	723.35	0.38
**1389123**	CHGB	1.71E-03	1178.28	444.38	0.38
**1644941**	CTXN3	3.84E-04	156.01	59.03	0.38
**1646728**	PCSK1	2.75E-03	306.17	115.85	0.38
**1391134**	ATP5G1	2.64E-03	116.78	44.81	0.38
**1391383**	CORT	3.79E-03	138.67	53.03	0.38
**1394742**	HNRNPA1	1.73E-05	57.66	22.21	0.39
**1394862**	PSMG1	< 1e-07	270.29	104.21	0.39
**1378095**	PLEKHB2	4.84E-05	358.92	138.77	0.39
**1377766**	CRSP9	4.64E-03	7.04	2.72	0.39
**1384092**	MAGED1	3.61E-05	22.6	8.76	0.39
**1377551**	VGF	1.64E-03	2746.91	1066.02	0.39
**1392537**	PSMG1	8.00E-07	172.02	67.01	0.39
**1381513**	LOC348840	1.83E-03	13.73	5.35	0.39
**1380551**	CSN1S1	6.72E-03	21.67	8.51	0.39
**1379547**	CRH	1.85E-03	126.22	49.63	0.39
**1383369**	TM2D3	3.00E-07	102.72	40.53	0.39
**1389211**	PSMA1	7.89E-05	193.24	76.25	0.39
**1390063**	NCOA2	7.10E-06	23.21	9.26	0.40
**1382229**	HLA-DQB1	4.44E-03	32.39	12.88	0.40
**1388266**	CCT6A	9.10E-06	152.42	60.98	0.40
**1386183**	MACROD2	2.48E-05	64.48	25.77	0.40
**1645009**	CNTN1	2.81E-05	445.99	178.24	0.40
**1382854**	NRG1	4.00E-07	35.61	14.3	0.40
**1645769**	SPAG8	3.56E-03	21.77	8.73	0.40

**1389404**	MIA	8.24E-03	6.36	2.55	0.40
**1393588**	SYT1	8.56E-03	3885.62	1563.21	0.40
**1646458**	RAP1GDS1	5.21E-05	114.15	46.09	0.40
**1379809**	SCN1B	8.70E-04	395.32	159.12	0.40
**1645671**	TAC3	7.90E-03	41.52	16.76	0.40
**1390147**	CNTN4	1.01E-04	108.29	43.92	0.41
**1389358**	TSC22D3	3.22E-04	25.95	10.49	0.40
**1386066**	GAD2	1.17E-04	578.14	234.96	0.41
**1393805**	ATP5C1	1.63E-05	764.17	312.46	0.41
**1645438**	UBE2E3	7.20E-06	285.89	117.7	0.41
**1645405**	USP14	1.02E-05	225.49	92.62	0.41
**1395408**	PIP4K2B	1.16E-05	306.97	126.57	0.41
**1384764**	PGAM1	5.51E-05	1615.74	665.35	0.41
**1389200**	GOLGA8G	1.66E-03	5.17	2.13	0.41
**1646189**	ANKHD1	4.52E-03	20.06	8.25	0.41
**1645132**	CCNC	6.58E-05	194.5	80.24	0.41
**1381919**	FAM3C	1.55E-04	579.86	239.96	0.41
**1383953**	GPLD1	2.55E-03	15.15	6.27	0.41
**1380649**	TASP1	< 1e-07	104.44	43.31	0.41
**1396482**	ASPH	2.99E-04	78.19	32.49	0.42
**1384585**	PDCD10	3.00E-07	49.68	20.7	0.42
**1384417**	PCDH8	3.90E-06	57.99	24.14	0.42
**1378751**	EID2	3.14E-05	360.35	149.87	0.42
**1646297**	ACOT1	1.58E-04	151.76	63.34	0.42
**1389253**	ABHD11	3.34E-04	47.85	19.91	0.42
**1387879**	SNX10	4.06E-03	446.92	185.98	0.42
**1644518**	FRMPD2	8.35E-03	69.05	28.72	0.42
**1389939**	HMGCLL1	4.00E-07	55.29	23.13	0.42
**1392030**	PHF14	1.75E-05	152.05	63.49	0.42
**1388504**	PREI3	6.79E-05	71.48	29.95	0.42
**1644028**	LOC201229	1.67E-04	148.02	61.91	0.42
**1390772**	DLD	2.00E-06	282.69	118.69	0.42
**1384978**	RTN1	1.62E-05	72.43	30.47	0.42
**1388268**	AP1S1	9.05E-04	885.35	371.47	0.42
**1644293**	HS6ST2	1.79E-05	68.59	28.89	0.42
**1392358**	ATP2B1	3.02E-03	415.52	175.2	0.42
**1392178**	FGF14	5.18E-04	153.6	64.96	0.42
**1387994**	NRXN1	8.75E-05	209.07	89.1	0.43
**1397086**	TSPAN3	1.04E-04	1007.04	428.16	0.43
**1644394**	GRIA4	1.76E-03	119.97	50.97	0.42
**1645331**	C10orf93	7.35E-03	13.01	5.54	0.43
**1377996**	FBXL17	7.35E-03	67.83	28.92	0.43
**1384834**	ASAH1	5.20E-06	190.55	81.41	0.43
**1644062**	LETMD1	2.23E-05	108.65	46.66	0.43
**1383621**	ATP5S	3.16E-04	50.66	21.76	0.43
**1390767**	WTAP	2.57E-03	11.48	4.94	0.43
**1389904**	RAD23B	6.00E-07	426.89	184.24	0.43
**1393995**	CDC42SE2	8.30E-06	49.93	21.48	0.43
**1382038**	SRPK2	1.37E-05	73.3	31.65	0.43
**1397081**	PCP4	8.94E-05	2761.72	1190.67	0.43
**1377573**	STAT4	6.18E-04	628.75	270.75	0.43
**1396933**	GPR89A	2.10E-05	87	37.71	0.43
**1397381**	HSD11B1	4.32E-04	86.28	37.27	0.43

**1395751**	STMN1	5.66E-04	21.7	9.4	0.43
**1392815**	KIAA1967	6.60E-04	21.81	9.46	0.43
**1644397**	GRIP1	1.69E-03	12.33	5.33	0.43
**1386269**	CIRBP	2.64E-05	3202.77	1395.16	0.44
**1645074**	CEP170	7.37E-03	21.79	9.48	0.44
**1646285**	ACP1	6.48E-05	1110.78	484.92	0.44
**1391453**	MAGED2	1.39E-04	45.84	20.07	0.44
**1380702**	MAEA	1.67E-04	100.59	44.03	0.44
**1396998**	PPM1A	8.60E-06	102.11	44.94	0.44
**1381708**	ZNF429	5.37E-03	20.5	9.03	0.44
**1394238**	RXFP1	7.17E-03	25.83	11.35	0.44
**1380344**	PPP2R5D	2.80E-03	38.92	17.23	0.44
**1377231**	C9orf72	1.18E-05	33.86	15.03	0.44
**1645391**	USP32	8.70E-05	33.72	14.96	0.44
**1383957**	ANO5	8.85E-05	16.12	7.17	0.44
**1382858**	PPM1B	1.54E-03	70.55	31.3	0.44
**1384776**	ZMYM3	1.56E-03	18.77	8.35	0.44
**1384744**	OPN3	4.63E-03	367.22	163.07	0.44
**1645289**	C17orf91	1.00E-07	112.43	50.13	0.45
**1646680**	PHYH	5.80E-06	157.59	70.48	0.45
**1645481**	TTC8	1.28E-05	159.69	71.36	0.45
**1389110**	TOMM20	1.55E-05	2864.4	1277.97	0.45
**1394413**	CHM	2.03E-04	31.72	14.15	0.45
**1377829**	OLFM3	5.38E-04	138.17	61.83	0.45
**1379619**	ZNHIT3	4.90E-06	89.7	40.35	0.45
**1643504**	ZNF226	1.12E-04	39.76	17.93	0.45
**1384568**	UQCC	1.19E-04	47.23	21.28	0.45
**1396080**	TRIM37	6.30E-04	1096.66	493.55	0.45
**1376926**	GGH	1.06E-03	29.03	13.08	0.45
**1385204**	TM6SF1	9.00E-07	377.54	171.05	0.45
**1387766**	RPS24	1.50E-06	423.94	191.9	0.45
**1388251**	CITED2	1.27E-05	268.88	121.48	0.45
**1392288**	MAGED2	2.09E-05	49.62	22.47	0.45
**1388656**	CHMP5	5.72E-05	644.98	292.33	0.45
**1644653**	FAM3C	5.79E-05	582.24	264	0.45
**1386149**	DMXL2	7.84E-05	216.39	98.07	0.45
**1396369**	WDR69	1.03E-04	53	23.93	0.45
**1645686**	SYNJ1	1.34E-04	78.33	35.42	0.45
**1391215**	SNRPN	3.88E-04	4403.59	1993.88	0.45
**1383752**	KCNJ12	8.49E-03	127.52	57.63	0.45
**1385249**	CCNDBP1	1.07E-05	39.68	18.01	0.45
**1377588**	PRDX2	8.05E-05	725.72	330.17	0.45
**1384312**	MOCS2	1.19E-04	158.89	72.21	0.45
**1393279**	WASF1	3.98E-03	28.01	12.72	0.45
**1385766**	HNRPM	7.08E-03	12.22	5.56	0.45
**1645392**	USP33	< 1e-07	79.46	36.23	0.46
**1396319**	SCP2	4.80E-06	132.41	60.34	0.46
**1646372**	RNF38	6.61E-05	118.14	53.91	0.46
**1377197**	SGPP1	7.05E-04	49.71	22.66	0.46
**1394156**	BNIP3L	3.91E-03	54.4	24.87	0.46
**1384806**	GPR155	4.46E-04	10.52	4.83	0.46
**1381566**	SNCA	9.44E-04	941.18	432.56	0.46
**1646886**	OPN3	1.99E-03	93.58	42.97	0.46

**1384538**	HSPB3	3.14E-03	380.31	174.39	0.46
**1644197**	IQWD1	3.32E-03	37.78	17.36	0.46
**1383903**	KCNC1	6.27E-03	53.96	24.71	0.46
**1396774**	UQCRC2	4.15E-05	288.15	132.95	0.46
**1383868**	RPL6	1.68E-04	215.82	99.26	0.46
**1381201**	TCP1	2.53E-04	90.13	41.59	0.46
**1645254**	C1QL3	2.66E-04	51.23	23.61	0.46
**1394037**	C14orf126	8.28E-04	29.1	13.4	0.46
**1377405**	FASTKD3	< 1e-07	59.79	27.69	0.46
**1646305**	ABHD11	3.66E-05	40.06	18.57	0.46
**1379788**	KLK12	9.74E-03	4.93	2.28	0.46
**1387159**	RCHY1	< 1e-07	127.88	59.41	0.46
**1389807**	NXPH1	< 1e-07	309.74	143.82	0.46
**1644331**	HIGD1A	2.42E-05	2039.96	950.79	0.47
**1390069**	RAN	2.56E-05	972.53	451.84	0.46
**1643654**	MYO5B	7.88E-05	21.43	9.97	0.47
**1393994**	DLX1	1.21E-04	432.2	200.99	0.47
**1383598**	WAC	7.42E-04	28.11	13.09	0.47
**1388901**	CCDC132	2.17E-03	85.99	39.94	0.46
**1381142**	CDKN3	3.56E-03	51.78	24.13	0.47
**1386375**	C14orf45	4.18E-03	13.07	6.07	0.46
**1391034**	PPP1R2	1.34E-05	574.25	268.57	0.47
**1645113**	CD47	8.27E-05	103.84	48.45	0.47
**1389598**	DOK5	6.69E-04	44.33	20.71	0.47
**1392824**	MATR3	1.53E-05	205.11	96.12	0.47
**1643618**	NETO1	2.44E-05	46.11	21.6	0.47
**1645578**	TMEM70	2.06E-04	30.84	14.5	0.47
**1394315**	LRRC24	3.34E-04	18.5	8.67	0.47
**1380665**	GAD1	5.68E-04	2995.28	1406.79	0.47
**1393931**	SERF1A	2.89E-05	24.5	11.55	0.47
**1390986**	PCGF6	7.63E-05	23.21	10.96	0.47
**1380351**	TCEAL6	1.41E-04	963.91	454.61	0.47
**1380165**	RGS12	5.70E-04	29.08	13.71	0.47
**1376970**	MED12L	1.32E-03	14.07	6.64	0.47
**1646602**	PPM1B	1.57E-03	30.46	14.35	0.47
**1397061**	VAMP1	2.70E-03	2937.83	1388.04	0.47
**1383210**	CACNB4	4.39E-05	123.51	58.59	0.47
**1390148**	CADPS	4.82E-04	170.01	80.75	0.47
**1397293**	CASD1	1.92E-05	208.48	99.26	0.48
**1644834**	DLX1	1.24E-04	64.17	30.55	0.48
**1384600**	DBN1	6.89E-04	13.8	6.58	0.48
**1380188**	DUSP6	1.04E-03	43.31	20.6	0.48
**1376918**	HDC	1.29E-03	17.32	8.23	0.48
**1389700**	ELMOD1	1.96E-03	1911.22	907.95	0.48
**1643923**	LOC645039	2.99E-05	62.98	30.07	0.48
**1394339**	SEPT5	4.16E-05	64.22	30.66	0.48
**1384952**	ASB3	1.02E-04	134.4	64.26	0.48
**1386915**	C7orf20	2.13E-04	46.27	22.14	0.48
**1395730**	RIMS1	2.59E-04	114.8	54.83	0.48
**1380032**	CCNH	8.96E-04	94.58	45.35	0.48
**1384675**	KCNK1	1.68E-03	279	133.72	0.48
**1378791**	DYNC1I1	2.56E-03	1574.57	752.36	0.48
**1377516**	SPG21	3.01E-03	28.62	13.67	0.48

**1645687**	SUSD4	5.47E-03	47.17	22.56	0.48
**1378701**	ACADSB	< 1e-07	55.75	26.76	0.48
**1395628**	TMEM70	1.43E-05	150.9	72.59	0.48
**1646606**	PPCS	8.17E-05	123.34	59.43	0.48
**1382408**	CLCN4	2.44E-04	72.8	34.96	0.48
**1384950**	BCL11A	7.02E-04	142.76	68.72	0.48
**1643646**	NAT5	1.30E-05	1093.99	529.58	0.48
**1383335**	STAG3L1	1.83E-05	57.74	27.84	0.48
**1382390**	SULT1A1	2.13E-05	203.62	98.39	0.48
**1392687**	SLC37A3	5.74E-05	36.51	17.65	0.48
**1390376**	TMEM35	5.04E-04	361.45	174.96	0.48
**1387219**	THYN1	1.77E-03	146.56	70.69	0.48
**1646060**	ATP9A	8.09E-05	35.61	17.32	0.49
**1644204**	ISCA1	9.32E-05	238.86	116.17	0.49
**1393310**	SLC41A2	4.16E-04	13.83	6.73	0.49
**1645141**	CCDC85A	5.66E-04	37.82	18.38	0.49
**1643483**	ZNF43	1.22E-03	5.97	2.9	0.49
**1387949**	APOL1	1.94E-03	136.71	66.41	0.49
**1377047**	GABARAPL2	5.24E-03	62.24	30.19	0.49
**1396914**	CDC42EP3	1.00E-05	33.09	16.18	0.49
**1393661**	RPH3A	2.88E-05	143.09	69.66	0.49
**1393516**	C1orf102	6.20E-05	53.44	26.12	0.49
**1645950**	SEDLP	1.08E-04	146.33	71.43	0.49
**1378328**	HSD11B1	5.77E-04	114.47	55.71	0.49
**1396459**	VPS16	1.10E-03	34.89	17.04	0.49
**1378698**	XK	4.77E-03	166.02	80.88	0.49
**1645390**	VAMP1	5.66E-03	151.86	74.23	0.49
**1378110**	DNAJC19	4.00E-07	286.28	140.06	0.49
**1385946**	TRIM23	9.03E-05	542.91	265.75	0.49
**1380899**	CREG2	7.63E-03	1421.82	696.13	0.49
**1392403**	LOC729399	1.25E-05	73.54	36.29	0.49
**1396728**	PIAS2	2.46E-05	159.62	78.49	0.49
**1395216**	MDH1B	2.99E-05	34.22	16.83	0.49
**1646521**	PSMC4	7.65E-05	220.06	108.3	0.49
**1644901**	DCLK1	5.72E-03	4568.07	2252.53	0.49
**1396282**	GCC2	2.13E-05	32.52	16.08	0.49
**1391465**	EIF5	1.21E-04	61.71	30.55	0.50
**1384535**	DNAJB6	1.46E-04	1461.78	723.67	0.50
**1394789**	ELF3	5.38E-04	15.47	7.65	0.49
**1393415**	NUDT18	2.78E-05	177.13	88.14	0.50
**1644783**	DYRK1A	4.15E-05	151.47	75.38	0.50
**1381809**	F8	5.56E-05	23.5	11.71	0.50
**1645444**	UBA3	1.34E-04	249.73	124.28	0.50
**1396666**	PPEF1	1.18E-03	115.75	57.53	0.50
**1384263**	TSPYL2	1.53E-03	215.14	107.14	0.50
**1380607**	RLBP1L1	8.40E-06	53.11	26.6	0.50
**1395861**	ST6GALNAC5	4.06E-03	101.48	50.66	0.50
**1384581**	OPN3	7.65E-03	118.9	59.38	0.50
**1392838**	DPYS	8.46E-03	49.44	24.72	0.50
**1384183**	TMEM14B	1.11E-05	740.94	371.64	0.50
**1382558**	AMACR	3.86E-05	100.41	50.41	0.50
**1386497**	KHDRBS2	5.92E-05	52.05	26.09	0.50
**1387885**	LYNX1	7.17E-05	37.69	18.92	0.50

**1380293**	NAP1L5	1.04E-04	2048.82	1031.8	0.50
**1396209**	CCNC	1.18E-04	131.68	66.02	0.50
**1382789**	ADK	1.32E-04	56.24	28.29	0.50
**1390589**	GNAQ	2.07E-04	379.44	190.88	0.50
**1385080**	TUBGCP5	1.05E-03	203.11	102.25	0.50
**1391132**	NME2	1.49E-03	39.72	19.97	0.50
**1646629**	PMS2	4.00E-07	37.79	19.12	0.51
**1391946**	HNRPK	6.00E-07	1274.62	644.26	0.51
**1381537**	UBLCP1	2.85E-05	280.17	141.25	0.50
**1390021**	APITD1	3.89E-05	72.69	36.69	0.50
**1390049**	GRM7	4.85E-05	25.73	12.99	0.50
**1644180**	KCMF1	5.09E-05	264.21	133.44	0.51
**1646609**	POT1	7.53E-05	36.18	18.24	0.50
**1645348**	BRUNOL4	2.99E-04	123.41	62.47	0.51
**1380703**	TTC32	3.14E-04	164.33	83.01	0.51
**1644041**	LOC134997	4.23E-04	1770.86	896.26	0.51
**1388217**	ACTR3B	1.25E-03	88.55	44.67	0.50
**1645016**	CLSTN1	1.93E-03	190.76	96.4	0.51
**1391782**	MAEL	2.06E-03	49.03	24.76	0.50
**1388681**	ETV1	2.90E-03	17.61	8.87	0.50
**1377921**	SNX24	7.87E-03	31.38	15.82	0.50
**1380341**	GNG2	8.89E-03	399.99	202.5	0.51
**1387911**	SLC2A11	< 1e-07	122.99	62.43	0.51
**1396977**	SCN8A	2.51E-05	26.52	13.47	0.51
**1644931**	CXorf40B	5.70E-05	137.56	69.74	0.51
**1379348**	SLC22A18AS	1.80E-03	83.92	42.6	0.51
**1379237**	SLC25A46	2.40E-06	232.49	118.64	0.51
**1396992**	P2RX5	3.07E-05	21.6	11.04	0.51
**1379201**	ZFPM2	6.44E-05	135.83	69.32	0.51
**1395793**	PFKFB2	1.14E-04	74.03	37.86	0.51
**1393491**	TARBP1	1.14E-04	276.08	140.52	0.51
**1385842**	C12orf24	8.56E-04	707.34	360.94	0.51
**1383548**	GLRB	6.02E-03	1284.76	654.04	0.51
**1393050**	ECHDC1	6.07E-03	40.46	20.67	0.51
**1390766**	PRPF40B	7.12E-03	6.49	3.31	0.51
**1645365**	WDR17	5.00E-07	107.46	55.15	0.51
**1384437**	TRO	3.61E-05	404.83	207.36	0.51
**1384300**	SUB1	6.12E-05	669.68	344.14	0.51
**1380195**	SUCLA2	1.58E-04	850.94	437.22	0.51
**1382226**	RFC2	4.31E-04	34.71	17.76	0.51
**1382971**	GABRB2	7.33E-04	11.41	5.85	0.51
**1394939**	METTL6	1.60E-03	28.16	14.44	0.51
**1389956**	CBLN2	9.01E-03	812.75	416.34	0.51
**1377389**	METTL5	8.00E-07	522.3	269.92	0.52
**1388885**	STS-1	1.80E-06	580.28	298.93	0.52
**1388953**	TSPYL5	4.90E-06	331.2	170.95	0.52
**1385905**	OLA1	1.21E-05	36.55	18.86	0.52
**1383032**	PFAAP5	3.12E-05	319.08	164.8	0.52
**1389541**	TCTEX1D2	3.79E-05	706.91	364.24	0.52
**1381771**	CHMP2A	5.40E-05	135.96	70.21	0.52
**1386582**	GRIA3	5.83E-05	72.43	37.3	0.51
**1392291**	ATRX	1.16E-04	25.72	13.27	0.52
**1388958**	SPIN2A	7.37E-04	19.99	10.3	0.52

**1382399**	GRIN2A	8.48E-04	79.65	41.16	0.52
**1385975**	UNC5D	9.14E-04	28.48	14.66	0.51
**1390985**	PDE4D	1.77E-03	218.29	112.32	0.51
**1388408**	MYL5	2.23E-03	133.02	68.5	0.51
**1388938**	HSPC159	2.71E-03	36.36	18.75	0.52
**1381388**	GNAO1	2.78E-03	310.17	160.09	0.52
**1378419**	TFG	5.12E-03	35.13	18.1	0.52
**1392767**	TRHDE	8.26E-03	19.33	9.94	0.51
**1383250**	SYNCRIP	< 1e-07	397.83	206.27	0.52
**1393258**	MRPS35	1.62E-05	229.95	119.16	0.52
**1646673**	PIAS2	3.71E-05	44.66	23.13	0.52
**1394263**	TIPRL	9.47E-05	323.2	167.17	0.52
**1386401**	TXNDC9	1.75E-04	80.78	41.87	0.52
**1392424**	PLK4	6.80E-04	39.81	20.62	0.52
**1387569**	RIT2	1.27E-03	238.6	123.31	0.52
**1389133**	PCSK2	1.60E-03	47.34	24.55	0.52
**1393565**	KIAA0251	3.17E-03	57.27	29.74	0.52
**1396558**	CCT6B	3.71E-03	39.38	20.36	0.52
**1386977**	SCAND1	2.00E-07	117.92	61.41	0.52
**1646179**	ANKRD29	3.87E-05	312.11	162.66	0.52
**1377680**	IARS	7.21E-05	577.11	301.33	0.52
**1643856**	MAEA	1.00E-04	272.03	141.83	0.52
**1381046**	CMC1	1.07E-04	133.48	69.57	0.52
**1394534**	MAP2	3.19E-04	295.7	154.14	0.52
**1380760**	GAD1	6.82E-04	36.73	19.17	0.52
**1395814**	HSD17B11	7.29E-04	32.5	16.91	0.52
**1378733**	C6orf114	7.64E-04	22.32	11.65	0.52
**1386843**	GDA	1.20E-03	19.87	10.37	0.52
**1644029**	LOC201229	1.29E-03	125.46	65.19	0.52
**1393398**	SLC16A6	1.42E-03	46.3	24.17	0.52
**1386973**	DRP2	1.75E-03	22.96	11.97	0.52
**1393282**	PAIP2	3.51E-03	37.59	19.58	0.52
**1381202**	C9orf4	3.54E-03	348.41	181.78	0.52
**1377283**	UCHL1	6.26E-03	4491.41	2344.36	0.52
**1389333**	ORMDL1	7.37E-03	16.59	8.66	0.52
**1643606**	NFU1	4.00E-07	555.47	290.71	0.52
**1377086**	USP16	6.00E-07	139.78	73.24	0.52
**1392667**	CASC4	9.00E-07	66.77	34.96	0.52
**1643832**	MAP2	2.52E-05	177.27	92.87	0.52
**1387791**	COPS4	3.49E-05	752.48	393.29	0.52
**1381184**	C14orf124	7.37E-05	108.62	56.87	0.52
**1393856**	PPM1A	3.23E-04	58.96	30.92	0.52
**1381722**	RASGRF1	1.04E-03	57.43	30.06	0.52
**1645049**	CHCHD7	3.97E-03	49.32	25.77	0.52
**1386064**	HACL1	1.90E-06	520.37	273.67	0.53
**1645759**	SPIN2B	4.10E-06	61.95	32.66	0.53
**1392255**	AUH	2.42E-04	474.03	248.87	0.53
**1385357**	POP4	4.41E-04	55.15	29.07	0.53
**1643648**	NAE1	8.75E-04	147.37	77.45	0.53
**1396238**	RTCD1	9.34E-04	177.56	93.61	0.53
**1378737**	NDUFA10	1.72E-03	218.81	114.92	0.53
**1387840**	B4GALT6	2.54E-03	101.35	53.46	0.53
**1381927**	EFCAB1	9.26E-03	71.17	37.5	0.53

**1644388**	GSTZ1	8.00E-07	91.22	48.26	0.53
**1386727**	CHMP2B	8.00E-07	282.81	149.46	0.53
**1387322**	PPP2CB	9.93E-05	116.71	61.7	0.53
**1396279**	MTUS1	1.78E-04	18.59	9.82	0.53
**1395476**	CLTC	2.65E-04	48.03	25.39	0.53
**1644307**	HNRNPH2	3.65E-04	240.67	127.31	0.53
**1390177**	FAM19A1	7.38E-04	343.7	181.86	0.53
**1383310**	FBLN7	1.68E-03	146.5	77.7	0.53
**1381350**	GPRASP1	6.10E-03	12.79	6.77	0.53
**1396247**	ZNF695	8.93E-03	6.33	3.35	0.53
**1646035**	BCAP29	1.53E-05	311.32	165.37	0.53
**1388468**	SRP72	1.58E-05	176.01	93.59	0.53
**1386173**	UGP2	4.55E-05	1101.59	587.33	0.53
**1377987**	GPATCH4	4.57E-05	189.83	101.23	0.53
**1391071**	NME2	5.59E-05	176.51	93.87	0.53
**1395872**	MGC4172	8.29E-05	553.16	293.76	0.53
**1646751**	PCDHA3	1.03E-04	30.15	16.08	0.53
**1645176**	CAMK2G	1.39E-04	663	352.64	0.53
**1378090**	GPRASP2	2.12E-04	800.25	426.04	0.53
**1380393**	THAP7	3.57E-04	25.18	13.38	0.53
**1388399**	SNRPN	8.55E-04	11.39	6.07	0.53
**1381307**	FGF12	1.11E-03	645.76	342.88	0.53
**1643968**	LOC440145	1.15E-03	119.89	63.65	0.53
**1381108**	HKDC1	2.63E-03	14.03	7.47	0.53
**1393875**	KIRREL3	4.18E-03	9.49	5.06	0.53
**1383942**	UCHL5IP	5.26E-03	32.66	17.38	0.53
**1392638**	C20orf107	1.29E-04	18.18	9.72	0.53
**1643544**	XPOT	7.67E-04	59.1	31.62	0.54
**1394016**	PPA2	7.99E-04	387.15	206.68	0.53
**1393396**	C3orf14	8.12E-04	1916.02	1024.18	0.53
**1382524**	MECR	8.23E-04	28.98	15.52	0.54
**1389530**	TMEM14A	1.39E-03	1216.19	650.27	0.53
**1377357**	D4S234E	1.85E-03	3294.2	1764.83	0.54
**1384022**	CACYBP	6.16E-03	740.04	396.8	0.54
**1382752**	RPA3	7.64E-05	519.83	279.73	0.54
**1645590**	TMEM120B	8.58E-05	26.38	14.16	0.54
**1646020**	BEX4	1.71E-04	2019.92	1083.49	0.54
**1396507**	CCBL2	1.00E-03	101.64	54.65	0.54
**1377548**	NDUFS2	1.28E-03	55.77	30	0.54
**1379524**	LOC400120	1.47E-03	178.41	95.89	0.54
**1391512**	ZNF226	2.68E-03	23.46	12.62	0.54
**1644297**	HPRT1	3.31E-03	2451.78	1321.2	0.54
**1397255**	RPS7	3.52E-03	83.59	44.89	0.54
**1384665**	C12orf54	4.94E-03	28.37	15.26	0.54
**1387454**	SPAG8	6.56E-03	28.23	15.17	0.54
**1396788**	PARK2	4.00E-07	34.41	18.61	0.54
**1646379**	RNF14	2.10E-06	397.21	215.07	0.54
**1644192**	ITPA	3.37E-05	146.8	79.4	0.54
**1381549**	NFS1	5.75E-05	64.97	35.11	0.54
**1391792**	NCAM2	6.15E-05	737.76	399.69	0.54
**1646522**	PSMD10	8.85E-05	285.27	154.35	0.54
**1385714**	PEX7	2.29E-04	114.3	61.81	0.54
**1646216**	AKIRIN1	3.54E-04	298.91	161.81	0.54

**1396779**	MOAP1	5.83E-04	1990.9	1073.67	0.54
**1644279**	HSPBP1	6.44E-04	81.89	44.35	0.54
**1391231**	TATDN1	1.40E-03	47.95	25.98	0.54
**1381622**	UCHL5	2.13E-03	346.08	186.74	0.54
**1389023**	NEGR1	2.55E-03	45.39	24.5	0.54
**1644396**	GRINA	2.10E-06	717.06	389.8	0.54
**1394977**	MPPED1	5.30E-06	32.69	17.81	0.54
**1383439**	SNAPC5	9.25E-05	71.9	39.03	0.54
**1382273**	SCOC	1.12E-04	976.99	531.72	0.54
**1392845**	PFN2	4.02E-04	1265.34	689.13	0.54
**1389204**	IDI1	1.50E-03	102.5	55.78	0.54
**1382205**	USP6	2.26E-03	16.11	8.76	0.54
**1387979**	CACNB2	3.78E-03	312.62	169.62	0.54
**1644117**	KITLG	4.67E-03	89.33	48.67	0.54
**1396367**	SYN1	5.80E-03	23.97	12.99	0.54
**1391096**	UTP18	2.10E-06	101.21	55.43	0.55
**1377963**	MRPL20	1.13E-05	306.15	167.56	0.55
**1644440**	GNL3	6.29E-05	393.37	215.29	0.55
**1392604**	KCMF1	7.52E-05	57.07	31.27	0.55
**1378012**	ABCA5	7.89E-05	96.1	52.42	0.55
**1377343**	MAD2L1BP	1.78E-04	171.99	94.15	0.55
**1386857**	NRG1	8.46E-04	21.3	11.65	0.55
**1377315**	C1orf94	8.93E-04	22.62	12.35	0.55
**1394773**	NME5	8.94E-04	487.34	266.34	0.55
**1386799**	TUSC3	2.39E-03	174.69	95.2	0.54
**1390427**	ACP1	2.28E-05	927.72	509.16	0.55
**1643942**	LOC653566	2.91E-05	306.87	168.65	0.55
**1385194**	CLASP2	4.37E-05	611.99	336.02	0.55
**1382748**	C14orf138	4.50E-05	164.8	90.32	0.55
**1643720**	MRPL21	5.00E-05	725.93	399.84	0.55
**1643949**	LOC653803	7.25E-05	24.51	13.48	0.55
**1646104**	ASPH	4.96E-04	99.28	54.69	0.55
**1379972**	ACOT4	3.64E-03	141.78	77.95	0.55
**1379391**	KIAA2022	5.71E-03	60.71	33.37	0.55
**1382112**	ANKRD13C	3.70E-06	144.45	79.99	0.55
**1645341**	BTBD1	6.39E-05	255.46	140.89	0.55
**1389869**	COX7B	8.16E-05	718.55	397	0.55
**1389625**	HSPBP1	9.47E-05	69.65	38.53	0.55
**1644158**	KCNQ5	1.14E-04	24.94	13.81	0.55
**1382706**	RQCD1	1.15E-04	299.95	165.35	0.55
**1389267**	ALG1	1.34E-04	35.89	19.84	0.55
**1382824**	RMND1	3.19E-04	68.54	37.86	0.55
**1644498**	G3BP2	3.26E-04	2311.47	1274.56	0.55
**1396547**	APOO	3.88E-04	531.14	293.73	0.55
**1389165**	CREBZF	1.38E-03	81.42	45.09	0.55
**1387715**	PIK3CB	1.50E-03	143.13	79.24	0.55
**1396881**	KCNA1	2.15E-03	32.56	18.01	0.55
**1395046**	FAM161B	5.01E-03	34.82	19.25	0.55
**1394170**	NPTN	7.43E-03	511.29	281.78	0.55
**1394020**	MOXD1	7.87E-03	95.14	52.65	0.55
**1393372**	CNIH3	8.86E-03	105.25	58.21	0.55
**1646123**	ARID4A	7.00E-07	72.66	40.27	0.55
**1380626**	TBRG1	1.80E-06	32.74	18.22	0.56

**1383170**	LIN52	3.30E-06	60.66	33.63	0.55
**1379861**	SNX4	3.94E-05	299.31	166.59	0.56
**1385896**	RAD51C	1.91E-04	202.96	112.57	0.55
**1390539**	TMTC3	2.47E-04	80.26	44.66	0.56
**1378396**	EIF1B	2.48E-04	1012.09	563.72	0.56
**1380689**	BCL10	2.95E-04	21.74	12.11	0.56
**1386433**	KCNN2	3.71E-04	48.23	26.86	0.56
**1645879**	SLC22A4	1.06E-03	33.54	18.62	0.56
**1390828**	PRPF6	1.08E-03	42.18	23.44	0.56
**1646023**	BCL11A	1.21E-03	284.97	158.71	0.56
**1381396**	SNCG	3.07E-03	388.68	216.15	0.56
**1393544**	FAM49B	7.02E-03	62.43	34.69	0.56
**1382978**	CSTF1	7.48E-03	15.75	8.73	0.55
**1645849**	SLC4A8	3.30E-06	44.33	24.78	0.56
**1388074**	SLC25A17	5.00E-06	45.88	25.59	0.56
**1387413**	PPIA	1.35E-05	65.11	36.38	0.56
**1391831**	LDHB	2.37E-05	1508.83	845	0.56
**1384966**	PPP1R1B	2.59E-05	20.1	11.22	0.56
**1376959**	FAIM	3.15E-05	57.25	31.99	0.56
**1377319**	C4orf27	3.34E-05	288.25	161.24	0.56
**1395716**	TBC1D23	4.56E-05	57.67	32.29	0.56
**1392840**	ASMTL	7.26E-05	96.33	53.9	0.56
**1378504**	PIGH	1.81E-04	114.9	64.04	0.56
**1378536**	MRPS30	1.86E-04	507.79	283.91	0.56
**1378523**	NCKAP1	2.79E-04	987.86	553.38	0.56
**1646807**	OSBPL3	3.55E-04	155.71	86.86	0.56
**1646563**	PRDX3	4.03E-04	1333.52	743.84	0.56
**1644237**	IHPK2	9.48E-04	85.6	47.73	0.56
**1389593**	C10orf67	1.85E-03	18.09	10.13	0.56
**1382113**	TMEM186	2.22E-03	38.29	21.42	0.56
**1385218**	LARP6	2.27E-03	176.72	98.47	0.56
**1646088**	ATG4B	2.27E-03	296.43	165.87	0.56
**1646337**	RPS26L	2.79E-03	925.1	517.5	0.56
**1383702**	PDPK1	5.56E-03	285.56	159.43	0.56
**1391301**	DGKI	6.74E-03	67.33	37.57	0.56
**1389731**	LBXCOR1	9.47E-03	9.95	5.56	0.56
**1380435**	CRYZL1	< 1e-07	849.87	477.55	0.56
**1391498**	CLIP4	2.28E-05	409.93	230.28	0.56
**1380222**	FLJ20125	6.11E-05	43.95	24.71	0.56
**1392041**	YEATS4	1.47E-04	110.27	61.94	0.56
**1377298**	MRPL15	1.86E-04	433.81	243.31	0.56
**1645951**	SEH1L	2.04E-04	157.26	88.54	0.56
**1382182**	SRPK2	2.57E-04	279.6	157.19	0.56
**1381276**	CTH	2.91E-04	100.96	56.79	0.56
**1382498**	SMYD3	3.30E-04	342.62	192.29	0.56
**1395064**	NRG3	3.85E-04	224.88	126.67	0.56
**1383044**	CYP4A11	9.88E-04	36.81	20.71	0.56
**1387228**	MRPL1	1.69E-03	135.4	76	0.56
**1383039**	TCEAL1	4.58E-03	17.3	9.7	0.56
**1383382**	GLS2	9.35E-03	180.35	101.42	0.56
**1394885**	TXNL2	3.08E-04	323.98	183.12	0.57
**1386838**	NALCN	4.39E-04	66.34	37.54	0.57
**1388335**	AMD1	1.08E-03	402.13	227.11	0.56

**1383166**	KRT222P	8.50E-03	679.31	384.37	0.57
**1384864**	CYB5D2	3.00E-07	402.6	228.48	0.57
**1378445**	C1orf165	1.90E-06	200.08	113.42	0.57
**1393049**	CASC4	2.42E-05	394.3	223.78	0.57
**1380467**	RAB3C	3.51E-05	56.2	31.92	0.57
**1396212**	NDUFS4	5.17E-05	1796.81	1020.73	0.57
**1389647**	ALDOA	2.52E-04	4505.5	2564.12	0.57
**1390141**	UBE2B	5.17E-04	33.67	19.08	0.57
**1645644**	TCEAL1	6.17E-04	305.78	173.92	0.57
**1379210**	ATP1A1	1.54E-03	2624.35	1491.77	0.57
**1382010**	BCAT1	1.60E-03	196.16	111.34	0.57
**1383095**	AADAT	1.81E-03	29.15	16.56	0.57
**1390019**	TMEM200A	2.15E-03	62.31	35.36	0.57
**1376883**	MAP1B	2.16E-03	2273.43	1293.16	0.57
**1387009**	RABL3	2.33E-03	76.03	43.19	0.57
**1380975**	MDH1	3.05E-03	11425.32	6481.63	0.57
**1396225**	TSC22D1	4.28E-03	76.81	43.65	0.57
**1378241**	TPTE2	8.64E-03	55.3	31.37	0.57
**1381955**	SCP2	1.78E-05	69.08	39.4	0.57
**1646688**	PGK1	1.98E-05	435.23	249.39	0.57
**1378617**	C17orf97	4.05E-05	368.23	210.26	0.57
**1390906**	GRIK2	4.80E-05	74.57	42.55	0.57
**1396813**	SMPD1	8.45E-05	98.06	56.18	0.57
**1644713**	ERP29	1.18E-03	261.75	149.42	0.57
**1644435**	GOLGA6B	1.39E-03	42.08	24.05	0.57
**1391603**	EIF5	1.88E-03	1077.43	615.37	0.57
**1645175**	CAMK2D	5.37E-03	57.07	32.62	0.57
**1385346**	KCTD8	6.49E-03	436.26	249.77	0.57
**1394023**	CASQ1	7.42E-03	191.59	109.64	0.57
**1394827**	PHTF2	9.85E-03	15.41	8.79	0.57
**1387448**	TMEM177	5.40E-06	219.67	126.08	0.57
**1646146**	APEX1	8.70E-06	618.3	356.06	0.58
**1377017**	HRASLS	1.53E-05	101.51	58.2	0.57
**1391692**	UBE2E3	1.78E-05	838.01	482.79	0.58
**1644383**	GTPBP8	1.88E-05	99.47	57.16	0.57
**1396669**	PLD3	2.29E-05	32.23	18.5	0.57
**1393239**	C3orf10	3.95E-05	721.37	414.18	0.57
**1386640**	PSMD7	4.20E-05	1121.34	643.13	0.57
**1391713**	TMEM126A	6.06E-05	853.97	490.76	0.57
**1388358**	HINT1	7.36E-05	6296.41	3618.86	0.57
**1388219**	UQCRFS1	9.02E-05	3551.17	2046.62	0.58
**1646325**	ABCA11	9.25E-05	53.12	30.53	0.57
**1390657**	ACTR10	1.32E-04	872.99	503.13	0.58
**1645973**	SBDSP	1.45E-04	192.8	111.01	0.58
**1379747**	PSPC1	1.57E-04	80.69	46.43	0.58
**1645403**	UQCRH	2.00E-04	5473.82	3146.71	0.57
**1391147**	ITGB1BP1	2.20E-04	776.89	446.03	0.57
**1389381**	GNB5	2.94E-04	251.63	144.77	0.58
**1378580**	ZNF25	2.95E-04	2069.9	1187.78	0.57
**1393934**	B3GALNT1	3.50E-04	27.85	16.03	0.58
**1391830**	MAPK9	8.04E-04	642	369.24	0.58
**1646512**	PTHLH	8.79E-04	57.54	33.02	0.57
**1396073**	OXCT1	9.27E-04	403.05	231.74	0.57

**1388622**	KCTD16	1.02E-03	70.81	40.59	0.57
**1380704**	TAF9	1.23E-03	30.7	17.68	0.58
**1393738**	GNG10	1.63E-03	339.48	194.91	0.57
**1645840**	SLC6A1	1.97E-03	607.61	348.95	0.57
**1391763**	VWC2	2.38E-03	74.17	42.73	0.58
**1396674**	TSGA14	2.84E-03	352.69	203.25	0.58
**1381882**	GSTM4	3.92E-03	35.97	20.67	0.57
**1383478**	MPO	6.09E-03	33.61	19.34	0.58
**1384839**	CDH13	7.93E-03	435.82	249.84	0.57
**1397007**	PREI3	9.20E-06	456.87	263.75	0.58
**1388401**	NOV	2.44E-05	303.92	175.91	0.58
**1391901**	KIAA0564	3.19E-05	22.77	13.13	0.58
**1396904**	SLC22A17	3.27E-05	44.12	25.45	0.58
**1381048**	FEZ1	1.28E-04	771.82	446.46	0.58
**1645878**	SLC22A18	1.70E-04	107.18	61.86	0.58
**1393312**	C10orf46	2.62E-04	26.17	15.11	0.58
**1389027**	ARMC8	4.11E-04	175.36	101.4	0.58
**1386405**	C6orf106	4.34E-04	57.16	32.99	0.58
**1378911**	ZBTB41	9.05E-04	40.49	23.38	0.58
**1378303**	DHDH	1.19E-03	102.16	59.16	0.58
**1381382**	ZNF655	1.50E-03	29.22	16.84	0.58
**1377072**	C1orf183	1.56E-03	35.49	20.48	0.58
**1646868**	OR2L13	5.46E-03	130.05	75.38	0.58
**1384276**	HOMER1	6.07E-03	89.95	52.09	0.58
**1390038**	CLTB	7.39E-03	149.61	86.3	0.58
**1385988**	DPP10	7.95E-03	145.45	84.07	0.58
**1395768**	GRIK1	9.94E-03	11.33	6.56	0.58
**1377151**	ICA1L	9.00E-07	43.28	25.15	0.58
**1388745**	SLC2A11	7.50E-06	72.39	42.03	0.58
**1379877**	PEX1	1.00E-05	110.07	63.98	0.58
**1386072**	PQBP1	1.23E-05	31.19	18.14	0.58
**1644819**	DNAJB12	2.43E-05	59.15	34.3	0.58
**1393360**	PAIP2	3.25E-05	1293.81	750.2	0.58
**1381368**	C11orf51	3.83E-05	107.07	62.43	0.58
**1646016**	BCLAF1	5.03E-05	224.98	130.91	0.58
**1384633**	ROPN1L	5.96E-05	62.8	36.51	0.58
**1645247**	C20orf7	7.15E-05	83.58	48.63	0.58
**1387016**	ING3	7.58E-05	33.37	19.35	0.58
**1646117**	ARMC10	1.35E-04	625	364	0.58
**1387919**	NEFL	3.98E-04	2247.19	1305.55	0.58
**1378525**	CXorf56	4.52E-04	68.86	39.97	0.58
**1384563**	AMDHD2	7.04E-04	46.08	26.83	0.58
**1383353**	CHAF1B	2.50E-03	70.7	41.19	0.58
**1646283**	ACOT7	3.55E-03	657.48	381.26	0.58
**1379413**	FGF13	6.42E-03	122.05	71.03	0.58
**1377485**	ARMCX5	7.27E-03	64.79	37.68	0.58
**1383556**	SUDS3	9.04E-03	18.04	10.51	0.58
**1393293**	UBE1DC1	8.20E-06	94.78	55.36	0.58
**1385838**	ME3	1.93E-05	201.71	118.23	0.59
**1646065**	ATRN	2.52E-05	71.29	41.62	0.58
**1393029**	ESD	4.08E-05	101.67	59.56	0.59
**1391129**	TSGA10	4.88E-05	43.35	25.28	0.58
**1386839**	YME1L1	4.19E-04	219.86	128.35	0.58

**1384270**	ATCAY	4.78E-04	424.93	248.8	0.59
**1645655**	TATDN3	5.41E-04	159.43	93.04	0.58
**1385405**	FAF1	1.19E-03	102.31	59.79	0.58
**1386871**	UBE2H	4.86E-03	29.06	17.03	0.59
**1378587**	FKBP3	4.86E-03	285.86	167.64	0.59
**1387334**	GLRX2	6.44E-03	146.3	85.81	0.59
**1387677**	SNCA	9.23E-03	3863.87	2264.72	0.59
**1381769**	SLC25A36	6.30E-06	260.33	153.45	0.59
**1395257**	WDR61	1.26E-05	829.81	488.42	0.59
**1384855**	CBWD5	3.82E-05	134.94	79.48	0.59
**1646072**	ATP5A1	8.62E-05	7639.38	4485.69	0.59
**1393653**	VPS29	8.96E-05	1325.08	779.47	0.59
**1390953**	SARS	1.31E-04	340.56	200.22	0.59
**1379133**	PRPS1	2.22E-04	605.87	355.57	0.59
**1645598**	TIMM23	2.55E-04	750.85	440.74	0.59
**1395191**	C8orf38	2.60E-04	91.94	54.07	0.59
**1645767**	SPG21	6.72E-04	254.75	149.93	0.59
**1645326**	C11orf49	9.52E-04	247.96	145.68	0.59
**1378726**	GNG13	1.19E-03	31.42	18.48	0.59
**1386157**	FAM162A	1.23E-03	126.83	74.43	0.59
**1389875**	LRCH1	1.26E-03	37.99	22.4	0.59
**1378028**	CCDC25	2.20E-03	122.51	71.97	0.59
**1380493**	PLCH1	3.13E-03	103.56	60.95	0.59
**1396106**	GULP1	3.42E-03	43.86	25.73	0.59
**1395677**	NRXN1	3.59E-03	213.68	125.85	0.59
**1390470**	PNMA1	3.66E-03	613.33	361.16	0.59
**1391079**	NHP2L1	5.15E-03	122.51	72.02	0.59
**1379166**	C6orf120	6.52E-03	37.97	22.31	0.59
**1390876**	LACTB2	1.17E-05	258.86	153	0.59
**1387113**	MFSD8	2.44E-05	96.21	56.87	0.59
**1397228**	APITD1	6.65E-05	237.89	140.76	0.59
**1385603**	MRPL35	1.22E-04	164.99	97.84	0.59
**1646658**	PJCG6	1.25E-04	56.44	33.47	0.59
**1382250**	IMMT	1.61E-04	443.63	262.37	0.59
**1378854**	MAPK10	1.80E-04	1138.43	674.03	0.59
**1381552**	PRKAG1	2.16E-04	281.84	167.03	0.59
**1392901**	ARMCX3	3.26E-04	566.58	335.68	0.59
**1395767**	DNM1L	3.94E-04	906.89	537.19	0.59
**1380296**	NRN1	4.44E-04	144.46	85.41	0.59
**1382769**	ATP5J	4.69E-04	623.24	367.98	0.59
**1388116**	PTCD2	6.63E-04	152.67	90.37	0.59
**1390838**	RAB2A	8.41E-04	144.38	85.4	0.59
**1646758**	PCBP4	1.63E-03	144.14	85.41	0.59
**1386925**	MAGEF1	2.29E-03	118.48	70.26	0.59
**1645318**	C13orf1	2.87E-03	425.32	252.27	0.59
**1644405**	GRB14	3.48E-03	127.27	75.29	0.59
**1645876**	SLC25A15	3.96E-03	18.82	11.12	0.59
**1390056**	RPS26	4.11E-03	1654.51	976.79	0.59
**1392046**	B3GNT6	6.31E-03	1695.92	1004.42	0.59
**1378318**	EAF1	6.98E-03	60.48	35.75	0.59
**1390948**	THOC3	9.69E-03	295.06	174.77	0.59
**1378066**	C2orf25	1.00E-06	591.15	352.59	0.60
**1384498**	FBXO3	8.00E-06	140.74	83.81	0.60

**1381194**	ACSL3	2.12E-05	282.53	167.84	0.59
**1382321**	PRMT6	2.95E-05	146.7	87.08	0.59
**1645599**	TIRAP	3.03E-05	16.28	9.7	0.60
**1643607**	NGDN	5.02E-05	232.94	138.58	0.59
**1392260**	ATP5L	6.12E-05	474.2	281.51	0.59
**1385670**	LYPLAL1	1.03E-04	260.37	155.32	0.60
**1382160**	C11orf49	1.13E-04	57.59	34.3	0.60
**1391286**	ELAC1	1.72E-04	23.22	13.79	0.59
**1394709**	TTPAL	4.07E-04	132.62	78.77	0.59
**1389972**	C1orf53	4.11E-04	149.01	88.72	0.60
**1393537**	C1orf128	4.91E-04	2700.06	1604.92	0.59
**1394589**	UBR1	6.44E-04	29.71	17.72	0.60
**1381931**	BTF3L4	9.03E-04	422.57	251.32	0.59
**1396353**	NPPC	9.26E-04	22.97	13.66	0.59
**1390103**	GLE1	1.05E-03	36.28	21.63	0.60
**1645966**	SCRN3	1.11E-03	37.56	22.35	0.60
**1396675**	C12orf5	1.90E-03	96.47	57.51	0.60
**1378432**	CUL3	3.04E-03	108.93	64.74	0.59
**1382702**	AMN1	3.12E-03	132.45	78.72	0.59
**1382991**	C15orf23	5.83E-03	54.83	32.72	0.60
**1644065**	LGALS8	6.15E-03	122.22	72.54	0.59
**1387968**	PAPD4	2.10E-06	278.94	166.83	0.60
**1386234**	MDP-1	6.80E-06	195.95	117.47	0.60
**1384630**	TTC19	4.46E-05	809.01	483.59	0.60
**1384718**	UQCRB	7.67E-05	102.35	61.47	0.60
**1377936**	SNX25	1.02E-04	176.93	105.92	0.60
**1395900**	POLR2B	1.27E-04	299.42	179.12	0.60
**1382563**	CD5	1.55E-04	41.12	24.66	0.60
**1385983**	MPHOSPH6	2.20E-04	71.07	42.49	0.60
**1644934**	CUL5	2.55E-04	570.28	340.53	0.60
**1390290**	PLEKHA3	5.62E-04	140.27	84.05	0.60
**1395293**	CNIH	9.96E-04	446.6	267.32	0.60
**1377065**	MRFAP1L1	1.13E-03	66.6	39.85	0.60
**1382645**	CHRFAM7A	1.84E-03	20.5	12.26	0.60
**1387336**	CABP1	6.50E-03	128.45	76.95	0.60
**1385079**	SPCS1	5.80E-06	2435.68	1470.87	0.60
**1386700**	C6orf57	1.04E-05	180.93	109.17	0.60
**1646779**	PANK2	1.24E-05	342.04	205.78	0.60
**1392321**	C2orf47	1.70E-05	295.58	178.42	0.60
**1392381**	TIMM17A	3.14E-05	47.01	28.4	0.60
**1378096**	HMGB3	7.02E-05	115.7	69.73	0.60
**1381642**	TMEM85	7.03E-05	2033.08	1223	0.60
**1394052**	DNAJC25	2.47E-04	156.15	93.99	0.60
**1393275**	FUNDC1	2.74E-04	279.7	168.94	0.60
**1383256**	ID2	5.79E-04	642.52	386.78	0.60
**1387541**	NOLC1	1.98E-03	102.7	61.75	0.60
**1380076**	MRPS34	3.06E-03	65.37	39.5	0.60
**1394072**	CMTM4	5.05E-03	26.92	16.24	0.60
**1384249**	NGEF	5.33E-03	711.97	429.23	0.60
**1386292**	HOPX	5.49E-03	22.97	13.87	0.60
**1644962**	CSNK1G3	8.41E-03	58.92	35.52	0.60
**1389519**	GOT1	9.17E-03	4557.71	2739.17	0.60
**1392854**	C9orf24	2.10E-06	664.05	403.33	0.61

**1395881**	HNRNPA0	2.70E-06	1394.31	842.8	0.60
**1380224**	C10orf4	6.40E-06	29.47	17.9	0.61
**1382050**	MCEE	1.08E-05	348.05	210.97	0.61
**1377360**	CXorf40A	1.29E-05	359.17	217.12	0.60
**1396687**	NIT2	4.53E-05	207.11	125.3	0.60
**1396079**	DHX36	7.90E-05	124.63	75.46	0.61
**1387195**	CCT7	1.59E-04	393.66	239.1	0.61
**1386801**	ATPIF1	1.60E-04	960.91	583.99	0.61
**1380140**	MAP4K3	1.62E-04	127.85	77.31	0.60
**1377871**	BLOC1S2	3.22E-04	68.29	41.4	0.61
**1387661**	TMEM69	4.63E-04	262.41	158.75	0.60
**1388340**	IL1RL2	6.42E-04	20.98	12.73	0.61
**1643869**	LRRTM3	9.97E-04	51.1	31	0.61
**1389208**	PSMA3	1.00E-03	100.27	60.8	0.61
**1384726**	CUL4B	1.15E-03	27.7	16.83	0.61
**1380906**	IHPK2	2.12E-03	137.12	83.25	0.61
**1377150**	FSIP2	3.84E-03	6.41	3.88	0.61
**1380671**	ARID4B	4.18E-03	36.51	22.15	0.61
**1390785**	PPP2R2C	5.83E-03	47.46	28.73	0.61
**1396084**	FXN	8.79E-03	15.34	9.32	0.61
**1382382**	SVOP	9.55E-03	875.5	529.35	0.60
**1393831**	KLHL23	3.36E-05	70.14	42.72	0.61
**1392646**	DTD1	3.44E-05	278.14	169.09	0.61
**1382617**	C4orf30	7.32E-05	171.02	104.23	0.61
**1397357**	ATP5O	1.51E-04	3557.91	2168.78	0.61
**1386721**	ALS2CR8	1.64E-04	31.07	18.91	0.61
**1381406**	STXBP5	2.91E-04	216.46	132.12	0.61
**1388788**	C7orf28A	2.94E-04	62.19	37.98	0.61
**1381097**	SMYD2	3.52E-04	529.25	322.4	0.61
**1378071**	RBM18	3.99E-04	107.96	65.88	0.61
**1392561**	THOC4	1.04E-03	50.96	31.04	0.61
**1386211**	PRMT8	1.06E-03	118.58	72.16	0.61
**1383141**	LRRC15	1.75E-03	53.16	32.43	0.61
**1643827**	MAP3K7	1.98E-03	70.58	43.17	0.61
**1380360**	PARP6	2.02E-03	49.12	29.94	0.61
**1389289**	TAC3	5.97E-03	152.2	92.95	0.61
**1389673**	UBE1DC1	8.75E-03	18.8	11.44	0.61
**1394125**	ZBBX	8.94E-03	125.93	76.76	0.61
**1380699**	AZIN1	1.97E-05	963.77	591.42	0.61
**1382354**	ANAPC10	2.39E-05	173.57	106.71	0.61
**1391654**	PSMA1	5.66E-05	844.58	519.13	0.61
**1377057**	POLR3C	1.11E-04	382.12	234.31	0.61
**1389469**	PFKM	1.56E-04	1126.03	691.21	0.61
**1388347**	FAM65B	2.23E-04	194.62	119.09	0.61
**1380887**	UBE2N	2.30E-04	2104.55	1294.32	0.62
**1396397**	CGREF1	2.53E-04	37.61	23.02	0.61
**1385333**	SLC25A5	2.86E-04	4427.72	2717.81	0.61
**1393787**	MAN1A1	3.10E-04	144.55	88.81	0.61
**1381942**	PPIL1	5.08E-04	221.23	136.06	0.62
**1389671**	CHCHD6	5.32E-04	448.92	274.96	0.61
**1646773**	PARG	6.80E-04	35.28	21.61	0.61
**1380547**	PIP5K1B	1.14E-03	43.24	26.46	0.61
**1376937**	CYB561	1.29E-03	222.19	136.08	0.61

**1380907**	CFL2	1.82E-03	49.38	30.37	0.62
**1646380**	RNF149	2.61E-03	28.13	17.28	0.61
**1383879**	PPM1E	2.75E-03	698.55	429.32	0.61
**1386929**	RBBP7	2.84E-03	162.2	99.45	0.61
**1382923**	RTN4IP1	3.31E-03	138.38	84.77	0.61
**1383022**	BRWD1	3.32E-03	1128.76	693.09	0.61
**1382083**	ATP6V1A	3.43E-03	5770.9	3529.73	0.61
**1392023**	TUBB3	3.61E-03	2978.08	1825.6	0.61
**1397169**	COG5	4.21E-03	50.83	31.21	0.61
**1394654**	DLX5	5.77E-03	110.31	67.7	0.61
**1386998**	SH3BGR	6.52E-03	95.45	58.65	0.61
**1382490**	C17orf75	9.16E-03	237.74	145.64	0.61
**1389528**	APEX1	2.30E-06	1141.84	705.45	0.62
**1646556**	PRKAG1	1.34E-05	187.17	115.37	0.62
**1390439**	GSTA4	1.44E-04	569.87	351.49	0.62
**1389605**	GPM6A	2.06E-04	325.5	201.13	0.62
**1390881**	MRPS23	4.85E-04	205.66	126.58	0.62
**1379006**	NGFRAP1	7.46E-04	976.74	604.43	0.62
**1646052**	ATXN10	8.65E-04	34.13	21.02	0.62
**1382239**	ADARB1	1.02E-03	44.67	27.56	0.62
**1644169**	KCNIP4	1.23E-03	1923.11	1187.47	0.62
**1394443**	DPH2	1.41E-03	22.13	13.7	0.62
**1390206**	SSX2IP	1.43E-03	691.06	427.85	0.62
**1381898**	NRXN3	1.73E-03	24.93	15.35	0.62
**1644659**	FAM19A4	2.32E-03	27.1	16.76	0.62
**1378742**	WDR55	3.18E-03	57.18	35.37	0.62
**1383322**	CAMK1G	4.22E-03	637.91	394.11	0.62
**1377009**	SUMF2	6.64E-03	71.81	44.32	0.62
**1377835**	BEST4	8.81E-03	26.88	16.59	0.62
**1378576**	COQ7	8.00E-07	81	50.39	0.62
**1644408**	GRIA3	1.20E-06	130.93	81.38	0.62
**1643591**	NME1	6.70E-06	170.5	106	0.62
**1380505**	JTV1	6.70E-06	472.56	294.31	0.62
**1377068**	ADSS	5.41E-05	1246.19	773.62	0.62
**1397325**	ZNF101	1.47E-04	24.56	15.23	0.62
**1390031**	ISCU	2.19E-04	762.88	474.36	0.62
**1389438**	DLAT	2.43E-04	322.82	200.76	0.62
**1384107**	OCIAD1	3.06E-04	2800.74	1735.7	0.62
**1386516**	RSPH1	3.09E-04	199.48	123.57	0.62
**1393621**	NDUFAB1	5.01E-04	2033.1	1263.38	0.62
**1383779**	HOXD13	6.62E-04	157.17	97.43	0.62
**1393367**	EPB41L3	7.87E-04	2040.91	1264.5	0.62
**1394260**	RAB18	8.05E-04	143.7	89.03	0.62
**1646170**	ANKRA2	1.31E-03	171.17	106.28	0.62
**1392850**	TM2D3	1.74E-03	1497.54	928.59	0.62
**1380100**	JAK1	1.74E-03	263.33	164.05	0.62
**1644057**	LGMN	1.81E-03	119.46	74.18	0.62
**1377394**	NDUFB6	1.95E-03	937.99	581.38	0.62
**1388029**	BAIAP2L2	3.03E-03	536.53	332.28	0.62
**1386188**	LAPTM4B	4.22E-03	905.76	563.25	0.62
**1385880**	SLC25A4	4.48E-03	4706.16	2919.08	0.62
**1381612**	NETO2	6.56E-03	381.72	237.01	0.62
**1385942**	PEMT	6.87E-03	36.64	22.73	0.62

**1381979**	HRNBP3	7.28E-03	14.83	9.2	0.62
**1382840**	TMCC1	7.78E-03	42.94	26.6	0.62
**1378372**	RPGRIP1L	8.21E-03	26.14	16.25	0.62
**1380445**	GOLT1B	9.30E-03	86.35	53.66	0.62
**1396662**	KCNIP2	9.39E-03	26.48	16.43	0.62
**1376985**	GTF2H3	3.60E-06	109.9	68.84	0.63
**1394559**	MDM4	2.01E-04	32.39	20.25	0.63
**1393685**	RPAIN	2.10E-04	368.66	230.57	0.63
**1382710**	FLJ20628	3.77E-04	89.69	56.01	0.62
**1645210**	C7orf44	5.45E-04	1052.84	656.55	0.62
**1385065**	OSGEPL1	7.93E-04	48.25	30.14	0.62
**1390862**	PAFAH1B1	8.73E-04	1801.82	1125.98	0.62
**1379591**	C9orf116	8.87E-04	141.96	88.8	0.63
**1389572**	PNPLA4	1.33E-03	45.33	28.4	0.63
**1380741**	MRPL39	1.46E-03	344.6	214.93	0.62
**1378301**	GPR176	1.61E-03	44.09	27.62	0.63
**1643531**	ZC3H15	1.73E-03	322.87	202.37	0.63
**1394035**	MAGI1	2.27E-03	147.46	92.11	0.62
**1380997**	UBE2G1	2.53E-03	352.33	220.06	0.62
**1377048**	RAB17	3.03E-03	59.97	37.6	0.63
**1394397**	CHRNA1	3.12E-03	18.54	11.6	0.63
**1383204**	MYRIP	3.65E-03	323.26	202.02	0.62
**1383637**	CYP2E1	4.12E-03	197	123.23	0.63
**1378856**	C2orf40	4.19E-03	97.87	61.27	0.63
**1645364**	WDR16	6.11E-03	26.72	16.67	0.62
**1395332**	ATP6V1C1	7.52E-03	323.71	201.99	0.62
**1390852**	LPHN3	8.61E-03	101.31	63.28	0.62
**1397265**	C6orf105	9.59E-03	24.81	15.51	0.63
**1393342**	TRIM32	1.80E-06	155.39	97.52	0.63
**1389131**	CORO2A	2.30E-06	72.51	45.71	0.63
**1394082**	CNOT7	7.90E-06	341.85	214.77	0.63
**1381295**	ANKRD16	9.70E-06	66.7	41.97	0.63
**1646240**	AGGF1	2.38E-05	451.36	283.11	0.63
**1389423**	BPGM	6.59E-05	112.9	70.96	0.63
**1387417**	KHDRBS1	8.11E-05	1353.66	851.05	0.63
**1382060**	LYRM2	8.97E-05	709.81	447.22	0.63
**1384804**	LYCAT	1.12E-04	140.15	88.22	0.63
**1394208**	ATP5F1	1.20E-04	1409.85	886.57	0.63
**1392109**	TXN	1.40E-04	1714.18	1077.93	0.63
**1384909**	GDF10	2.65E-04	89.68	56.33	0.63
**1377101**	SPA17	2.81E-04	220.28	138.8	0.63
**1646652**	PKP2	3.22E-04	32.82	20.63	0.63
**1386512**	PMS2	3.79E-04	49.28	31.06	0.63
**1382646**	RAD51C	6.49E-04	257.32	161.94	0.63
**1391997**	HMBS	6.57E-04	53.37	33.48	0.63
**1392754**	ARL4C	9.19E-04	44.1	27.76	0.63
**1644678**	FAM10A7	9.29E-04	141.9	89.52	0.63
**1386579**	AGK	1.08E-03	559.66	352.64	0.63
**1382156**	C12orf31	1.32E-03	96.71	60.75	0.63
**1644379**	GTF2I	2.98E-03	19.58	12.29	0.63
**1645178**	CAMKK1	3.06E-03	72.7	45.67	0.63
**1397337**	ABHD7	4.13E-03	147.43	92.54	0.63
**1382126**	BTBD10	4.37E-03	559.16	352.3	0.63

**1378510**	ABCC12	4.41E-03	160.96	101.44	0.63
**1643970**	LOC440354	5.84E-03	95.98	60.19	0.63
**1392236**	C1orf19	1.20E-06	454.74	288.68	0.63
**1378024**	MXRA7	6.60E-06	179.31	113.72	0.63
**1390115**	FAM18B	1.89E-05	176.47	111.41	0.63
**1397190**	GBAS	2.61E-05	306.77	193.79	0.63
**1392630**	LOC441150	4.28E-05	106.93	67.74	0.63
**1383755**	BFSP1	1.18E-04	34.7	22.01	0.63
**1644113**	KLHDC9	2.72E-04	301.12	190.01	0.63
**1377482**	C12orf4	2.74E-04	196.28	124.35	0.63
**1391109**	C9orf103	2.86E-04	550.98	348.79	0.63
**1646074**	ATP5J	3.17E-04	5585.71	3535.45	0.63
**1384193**	FANCF	3.35E-04	20.25	12.84	0.63
**1378885**	TMEM55A	3.52E-04	450.91	285.25	0.63
**1386238**	SC5DL	4.77E-04	1510.86	956.98	0.63
**1377892**	DLG3	5.50E-04	52.1	33.01	0.63
**1644357**	HAX1	5.75E-04	90.53	57.25	0.63
**1378953**	WWP2	6.95E-04	179.71	113.43	0.63
**1394402**	CD200	1.30E-03	122.67	77.8	0.63
**1387912**	PHYHIPL	1.33E-03	941.79	594.75	0.63
**1394623**	EEF1B2	1.57E-03	1099.38	696.86	0.63
**1378651**	GNG3	1.70E-03	1605.26	1017.9	0.63
**1644219**	ING2	1.77E-03	113.47	72.04	0.63
**1391407**	TTBK2	2.93E-03	35.86	22.72	0.63
**1646071**	ATP2C1	3.83E-03	363.35	230.16	0.63
**1387861**	NME1	4.08E-03	1636.06	1032.51	0.63
**1393192**	CACNA2D1	4.96E-03	15.32	9.73	0.64
**1395915**	DNM1L	5.13E-03	107	67.65	0.63
**1390733**	MAK16	5.13E-03	99.74	63.15	0.63
**1393421**	ARPC3	5.14E-03	1273.12	808.14	0.63
**1383105**	TUBA1C	7.81E-03	8643.45	5487.22	0.63
**1394843**	TIPRL	8.85E-03	78.24	49.44	0.63
**1383784**	CADM2	5.05E-05	433.77	277.01	0.64
**1645642**	TCEA1	5.64E-05	303.63	192.99	0.64
**1393434**	SCO1	7.48E-05	399.09	253.4	0.63
**1377603**	SFTPD	1.13E-04	129.74	82.5	0.64
**1397233**	ALDH5A1	1.74E-04	943.82	599.29	0.63
**1386044**	OXCT2	2.26E-04	105.97	67.31	0.64
**1393101**	SLC25A26	2.76E-04	146.79	93.34	0.64
**1396391**	SCAND1	3.28E-04	393.08	250.04	0.64
**1646108**	ARPC4	3.43E-04	751.38	477.7	0.64
**1391859**	MRPS28	4.91E-04	285.68	181.39	0.63
**1644711**	ERCC8	6.83E-04	21.1	13.4	0.64
**1386712**	CTSL1	9.25E-04	77.44	49.35	0.64
**1389455**	FBXO32	9.34E-04	160.65	102.05	0.64
**1391533**	NFYB	1.07E-03	62.58	39.78	0.64
**1384256**	NDUFA12	1.61E-03	2338.95	1490.33	0.64
**1378585**	C1orf59	2.59E-03	573.75	365.44	0.64
**1395706**	COMMD10	3.30E-03	234.71	149.47	0.64
**1643809**	MAX	4.11E-03	28.99	18.44	0.64
**1389335**	SYTL2	4.84E-03	35.76	22.75	0.64
**1378782**	IFT52	6.48E-03	41.45	26.45	0.64
**1383023**	IPO7	9.06E-03	80.56	51.25	0.64

**1392791**	EXOSC6	6.67E-05	332.15	213.06	0.64
**1381274**	THYN1	1.68E-04	698.55	447.78	0.64
**1378728**	ANKMY2	2.60E-04	561.59	359.48	0.64
**1384492**	CHMP6	2.71E-04	71.04	45.47	0.64
**1377790**	TRMT11	2.79E-04	130.65	84.01	0.64
**1386152**	C1orf57	2.81E-04	337.23	215.66	0.64
**1393774**	ATP6V0D1	3.32E-04	1324.33	849.39	0.64
**1383517**	PMPCB	3.52E-04	800.65	513.81	0.64
**1378873**	DHX9	3.59E-04	99.68	63.92	0.64
**1384106**	TRAPPC4	4.01E-04	784.14	504.23	0.64
**1390050**	DNER	4.33E-04	985.19	632.29	0.64
**1646402**	RGN	4.54E-04	41	26.25	0.64
**1391848**	MAX	4.74E-04	50.12	32.18	0.64
**1644206**	ISCU	6.63E-04	3147.27	2014.66	0.64
**1646527**	PSMA3	8.37E-04	265.15	169.7	0.64
**1382560**	LIN7B	1.21E-03	604.96	387.05	0.64
**1643919**	LOC644096	1.23E-03	79.38	50.82	0.64
**1644260**	IDH3B	1.26E-03	1123.66	718.83	0.64
**1388777**	ICA1L	1.86E-03	187.31	119.72	0.64
**1388945**	INPP5F	1.87E-03	135.37	86.52	0.64
**1383389**	NKIRAS1	2.67E-03	1362.26	870.62	0.64
**1383732**	HM13	3.14E-03	80.58	51.77	0.64
**1378021**	NDRG3	3.25E-03	1187.94	762.5	0.64
**1380441**	ATP6V0C	4.25E-03	879.07	564.7	0.64
**1645971**	SCG3	5.00E-03	1272.18	814.33	0.64
**1391936**	RNF122	6.42E-03	56.99	36.5	0.64
**1643815**	MARCH5	8.93E-03	89.64	57.58	0.64
**1395845**	ILF3	2.43E-05	105.55	68.01	0.64
**1384299**	CUTC	8.16E-05	197.24	127.35	0.65
**1646608**	PORCN	1.85E-04	124.54	80.28	0.64
**1377733**	CEP68	2.72E-04	180.47	116.53	0.65
**1386703**	CCNDBP1	3.46E-04	1113.85	718.54	0.65
**1383231**	RNF128	4.91E-04	21.13	13.59	0.64
**1395318**	NHP2L1	6.06E-04	2322.88	1500.54	0.65
**1384632**	TXNL4A	6.24E-04	89.08	57.65	0.65
**1393636**	C1orf149	6.28E-04	410.96	264.43	0.64
**1396325**	RFC3	6.38E-04	41.29	26.62	0.64
**1380030**	ZFYVE9	6.41E-04	13.55	8.76	0.65
**1394296**	C20orf7	6.97E-04	485.56	313.27	0.65
**1394288**	MRPL30	7.54E-04	71.51	46.19	0.65
**1391771**	SUMO2	8.51E-04	846.15	546.25	0.65
**1378147**	KDELR3	8.81E-04	27.62	17.85	0.65
**1383018**	ATP5J	8.98E-04	5043.85	3264.3	0.65
**1391068**	FGF9	9.00E-04	699.45	450.52	0.64
**1646476**	RAB5C	1.18E-03	204.2	131.78	0.65
**1377808**	LRRC8B	1.37E-03	40.67	26.26	0.65
**1384197**	SEC61G	1.50E-03	1656.99	1066.58	0.64
**1376910**	DNAJB14	1.52E-03	208.53	134.42	0.64
**1391010**	C18orf19	1.89E-03	41.72	26.87	0.64
**1646394**	RHOT1	2.38E-03	527.28	340.66	0.65
**1644243**	IGSF3	2.46E-03	28.97	18.63	0.64
**1381150**	ADK	4.05E-03	18.63	12.04	0.65
**1382414**	ADH5	4.17E-03	64.28	41.57	0.65

**1390691**	RNF111	6.57E-03	53.02	34.25	0.65
**1378883**	SNTG1	8.70E-03	93.12	60.12	0.65
**1378950**	SLC20A1	2.80E-06	1202.34	783.14	0.65
**1394905**	ACTR8	9.80E-06	66.28	43.03	0.65
**1383590**	TTC37	4.86E-05	378.87	245.92	0.65
**1386490**	C15orf44	5.45E-05	42.64	27.75	0.65
**1382165**	SSBP1	5.70E-05	787.19	511.01	0.65
**1387716**	EBNA1BP2	6.95E-05	1555.36	1006.76	0.65
**1391263**	LMBR1	6.96E-05	620.54	403.14	0.65
**1382158**	ECOP	8.65E-05	131.06	85.3	0.65
**1384019**	BECN1	9.86E-05	332.38	216.53	0.65
**1644144**	KIAA1128	1.44E-04	769.94	499.88	0.65
**1378929**	LCMT2	1.46E-04	127.28	82.44	0.65
**1391377**	TPM3	1.63E-04	592.93	384.37	0.65
**1378291**	CLTA	2.97E-04	590.41	384.46	0.65
**1393137**	XRCC5	3.31E-04	303.37	196.47	0.65
**1643852**	MAGED1	4.10E-04	1550.88	1005.52	0.65
**1393190**	NOLA2	4.27E-04	500.36	325.37	0.65
**1644808**	DPP8	4.56E-04	240.81	156.82	0.65
**1387557**	HTRA2	5.04E-04	131.23	85.3	0.65
**1384497**	ATP5S	5.06E-04	89.4	58.06	0.65
**1396465**	HLTF	5.08E-04	261.8	170.04	0.65
**1393043**	NCAPH2	5.41E-04	14.84	9.66	0.65
**1377535**	RND3	8.38E-04	129.67	84.32	0.65
**1376994**	AGPAT5	1.03E-03	156.21	101.49	0.65
**1380714**	CISD1	1.04E-03	3497.36	2274.93	0.65
**1379588**	C7orf36	1.16E-03	103.19	67.21	0.65
**1645190**	CABYR	1.20E-03	159.02	103.53	0.65
**1380523**	PSMD14	1.20E-03	496.78	322.21	0.65
**1383749**	CLYBL	1.79E-03	150.63	97.84	0.65
**1394711**	B3GALNT1	2.80E-03	182.88	118.61	0.65
**1381926**	BHLHB9	4.18E-03	283.39	184.34	0.65
**1379329**	STT3B	4.21E-03	74.01	48.18	0.65
**1385789**	LOC400506	4.29E-03	49.33	31.93	0.65
**1386362**	TSC1	4.67E-03	18.27	11.9	0.65
**1377114**	AKTIP	5.00E-03	174.02	112.8	0.65
**1644593**	FIGNL1	6.08E-03	40.01	26	0.65
**1389584**	ARSF	7.09E-03	27.54	17.84	0.65
**1395219**	VRK3	7.23E-03	53.99	34.96	0.65
**1377472**	C6orf66	9.55E-03	19.33	12.58	0.65
**1388473**	HAS1	9.84E-03	23.12	15.01	0.65
**1388017**	MXI1	9.91E-03	28.63	18.64	0.65
**1379941**	SUV420H1	1.32E-05	55.04	35.94	0.65
**1387936**	BMP2K	3.18E-05	38.59	25.24	0.65
**1377148**	C3orf31	8.73E-05	262.18	171.22	0.65
**1645720**	ST3GAL5	1.72E-04	295.6	193.56	0.65
**1377247**	VKORC1L1	1.95E-04	342.35	223.17	0.65
**1379976**	RALB	2.10E-04	2025.38	1320.31	0.65
**1395037**	POP7	2.21E-04	482.75	314.83	0.65
**1396194**	CDC123	2.32E-04	519.46	339.11	0.65
**1387319**	C14orf100	2.98E-04	271.35	177.63	0.65
**1381075**	CSNK2A1	3.13E-04	249.3	162.83	0.65
**1383214**	FBXO11	3.15E-04	53.39	34.99	0.66

**1381296**	CHCHD7	5.05E-04	488.06	319.42	0.65
**1387664**	MCART1	8.94E-04	23.81	15.57	0.65
**1387524**	DUS4L	1.17E-03	173.69	113.22	0.65
**1393083**	C10orf32	1.29E-03	425.54	278.33	0.65
**1384435**	MORF4L1	1.40E-03	1630.37	1067.3	0.65
**1391193**	KIAA0859	1.67E-03	236.82	154.73	0.65
**1385447**	RAB33A	1.72E-03	297.13	194.26	0.65
**1378070**	C3orf26	2.18E-03	639.44	419.07	0.66
**1392364**	TCEAL4	2.32E-03	60.35	39.5	0.65
**1394051**	B3GNT2	2.43E-03	79.09	51.77	0.65
**1378611**	C14orf129	3.12E-03	90.23	59.05	0.65
**1385523**	C1orf135	3.46E-03	45.82	29.9	0.65
**1382085**	HN1	3.71E-03	131.2	85.53	0.65
**1645120**	CCPG1	4.37E-03	46.55	30.4	0.65
**1644883**	DEFB131	4.40E-03	35.65	23.25	0.65
**1646286**	ACPL2	4.86E-03	299.67	196.42	0.66
**1382298**	ASTN1	5.20E-03	32.44	21.25	0.66
**1396689**	C9orf150	5.31E-03	47.91	31.28	0.65
**1379539**	SDHD	6.59E-03	327.43	214.58	0.66
**1384171**	NECAP1	7.46E-03	2700.91	1764.64	0.65
**1384420**	PCDH10	7.62E-03	74.76	48.82	0.65
**1381862**	TMED4	4.00E-07	556.95	365.74	0.66
**1384693**	DHRS7B	1.42E-05	164.09	108.28	0.66
**1385520**	ZMYM5	1.60E-05	53.31	35.04	0.66
**1390956**	MMACHC	3.02E-05	112.65	74.21	0.66
**1389965**	AZI2	1.82E-04	204.83	134.69	0.66
**1382211**	C11orf31	1.99E-04	75.71	49.7	0.66
**1645351**	BOLA3	3.30E-04	996.55	657.52	0.66
**1389222**	H2AFY	4.34E-04	465.51	306.06	0.66
**1392460**	GSTM1	4.49E-04	1614.7	1063.76	0.66
**1377267**	CD2BP2	4.84E-04	768.83	506.32	0.66
**1379148**	MOSC2	5.42E-04	95.68	62.77	0.66
**1645603**	THYN1	6.42E-04	553.91	364.8	0.66
**1395386**	AGA	6.51E-04	152.08	100.31	0.66
**1397291**	TBL1XR1	7.23E-04	108.37	71.4	0.66
**1382424**	STAM	7.50E-04	553.18	362.8	0.66
**1390962**	KIAA0232	8.67E-04	255.45	168.03	0.66
**1385269**	BTRC	9.70E-04	19.71	12.99	0.66
**1380880**	SGPP2	1.03E-03	22.12	14.58	0.66
**1389010**	ZMAT2	1.31E-03	1187.32	783.22	0.66
**1644077**	LCMT1	1.49E-03	938.09	616.57	0.66
**1646545**	PRKDC	1.76E-03	16.58	10.9	0.66
**1394948**	SLC6A15	2.38E-03	26.31	17.3	0.66
**1646910**	NUP54	2.77E-03	174.98	114.77	0.66
**1644042**	LOC143543	3.25E-03	22.65	14.87	0.66
**1395674**	WDFY3	5.35E-03	21.52	14.17	0.66
**1396278**	NCAPG2	5.68E-03	35.18	23.08	0.66
**1381039**	HTR3B	5.80E-03	24.76	16.29	0.66
**1380323**	MCAT	5.91E-03	48.38	31.91	0.66
**1646190**	AMY1A	6.10E-03	45.01	29.62	0.66
**1380862**	FBXO44	6.68E-03	74.02	48.78	0.66
**1394681**	MAST4	7.07E-03	45.46	29.93	0.66
**1386265**	FCRLB	8.41E-03	134.64	88.57	0.66

**1378686**	PCDHB12	9.59E-03	46.88	30.79	0.66
**1646019**	BEX2	9.81E-03	3922.75	2579.25	0.66
**1392721**	UCRC	4.58E-05	675.43	447.21	0.66
**1397004**	CISD2	5.65E-05	169.8	112.18	0.66
**1376928**	PITPNA	9.95E-05	279.93	185.85	0.66
**1645240**	C21orf33	1.12E-04	314.2	208.3	0.66
**1392635**	ERICH1	1.13E-04	515.77	341.5	0.66
**1393359**	RAB4A	1.25E-04	296.53	196.92	0.66
**1381468**	RPP40	1.31E-04	418	277.27	0.66
**1380608**	PPAPDC2	1.33E-04	112.12	74.34	0.66
**1387059**	ALS2CR4	1.43E-04	273.18	181.29	0.66
**1377168**	DOLK	1.58E-04	142.5	94.37	0.66
**1644605**	FEN1	2.72E-04	444.47	294.19	0.66
**1387141**	RAB28	2.92E-04	113.53	75.36	0.66
**1389380**	ZNF707	3.91E-04	23.22	15.42	0.66
**1379257**	LRRC7	8.73E-04	64.39	42.54	0.66
**1382586**	MRPL22	8.89E-04	325.04	215.09	0.66
**1395071**	NUDT9	9.59E-04	19.23	12.74	0.66
**1381500**	ERO1LB	1.61E-03	30.45	20.11	0.66
**1389118**	C12orf49	1.83E-03	60.92	40.26	0.66
**1391468**	INCA1	2.26E-03	26.77	17.73	0.66
**1393804**	MRPL46	2.53E-03	489.38	325.07	0.66
**1644743**	EIF5	3.12E-03	92.61	61.23	0.66
**1381162**	ADAM28	3.31E-03	21.56	14.25	0.66
**1377200**	PCNA	4.14E-03	20.76	13.79	0.66
**1645298**	C17orf58	4.15E-03	360.82	239.02	0.66
**1644709**	ETNK1	4.86E-03	15.33	10.15	0.66
**1384008**	CXCL14	6.92E-03	551.4	365.97	0.66
**1379248**	OPA1	7.65E-03	698.21	461.07	0.66
**1377544**	C9orf23	8.71E-03	45.93	30.32	0.66
**1386489**	VAMP7	7.86E-05	605.55	402.61	0.66
**1378775**	LOC400657	1.36E-04	123.26	82.02	0.67
**1378871**	ETFB	2.42E-04	905.22	601.51	0.66
**1389235**	PTRH2	3.69E-04	500.86	333.26	0.67
**1393297**	C7orf30	5.17E-04	980.58	651.66	0.66
**1644816**	DNASE1L1	1.43E-03	139.13	92.52	0.66
**1378245**	DDX47	1.95E-03	245.37	163.44	0.67
**1388718**	MTHFD1L	2.03E-03	217.47	144.61	0.66
**1389073**	VPS25	2.22E-03	184.18	122.7	0.67
**1395326**	EBP	2.64E-03	149.38	99.26	0.66
**1379716**	FKBPL	2.92E-03	39.32	26.2	0.67
**1385343**	ATP6AP2	3.21E-03	2986.99	1990.37	0.67
**1395491**	VDAC1	3.72E-03	2773.54	1843.32	0.66

3) List of genes differentially expressed between F+NAGM and CTR samples

p<0.01 Fold Change≥1.5 vs Control group

(Fold Change F+ NAGM/CTR ≥1.5= Up-regulated in F+NAGM)

(Fold Change F+ NAGM/CTR ≤0.66= Down-regulated in F+NAGM)

**F+ NAGM vs CTR**					
			**Geometric Mean of Intensity**		**Fold Change**
**UniqueID**	**Symbol**	**Parametric p-value**	**CTR**	**F+ NAGM**	**F+NAGM/CTR**
**1381005**	IGLL1	9.00E-07	12.53	235.75	18.81
**1646743**	PCDHB6	5.47E-03	0.35	4.86	13.89
**1389039**	HLA-DRB1	3.50E-03	4.93	46.22	9.38
**1644333**	HIGD2BP	4.11E-03	0.68	4.67	6.87
**1394705**	PNLIPRP3	2.58E-03	1.03	4.68	4.54
**1384093**	CCDC80	4.01E-04	2.04	8.13	3.99
**1392819**	ATRX	5.48E-03	1	3.79	3.79
**1396865**	PDYN	5.53E-03	57.31	208.43	3.64
**1379832**	CSNK1A1L	2.57E-04	1.41	4.85	3.44
**1377824**	NPC1L1	1.31E-04	4.29	14.38	3.35
**1377223**	LOC402573	2.62E-04	9.56	31.62	3.31
**1395475**	CXCL9	9.34E-03	4.19	12.69	3.03
**1645241**	C21orf41	8.05E-03	1.13	3.4	3.01
**1386747**	CETN1	7.36E-03	1.65	4.95	3.00
**1384695**	KCTD4	3.06E-05	11.08	33.18	2.99
**1381779**	IGSF22	1.30E-03	2.6	7.33	2.82
**1644798**	DRD2	5.91E-04	2.85	7.87	2.76
**1377837**	MGC29506	6.46E-04	19.93	55	2.76
**1644275**	HSP90AB4P	7.91E-03	1.78	4.9	2.75
**1377480**	ZNF645	4.05E-03	1.65	4.51	2.73
**1378600**	FAM46C	2.87E-05	20.49	55.55	2.71
**1393216**	CARD14	5.42E-04	2.02	5.45	2.70
**1391212**	HSFY1	2.67E-03	3.36	9.06	2.70
**1394564**	CD79A	9.55E-04	13.68	35.66	2.61
**1397122**	IL7R	7.34E-04	3.25	8.42	2.59
**1384958**	FCRLA	9.21E-03	4.46	11.48	2.57
**1644109**	KLKBL4	6.61E-03	1.36	3.45	2.54
**1646789**	P2RX2	6.14E-03	11	27.4	2.49
**1645498**	TRPV5	5.07E-03	2.49	6.2	2.49
**1391281**	TNFRSF17	7.31E-03	2.33	5.79	2.48
**1645668**	TAGAP	5.82E-03	3.97	9.86	2.48
**1377925**	MS4A14	5.03E-03	8.82	21.78	2.47
**1643460**	ZNF75D	8.08E-03	18.23	44.67	2.45
**1383742**	HNMT	8.61E-03	2.79	6.65	2.38
**1396240**	SLC5A3	7.23E-04	28.47	66.9	2.35
**1377929**	KIAA1529	6.72E-04	3.98	9.13	2.29
**1377238**	ZP2	8.41E-03	2.66	6.07	2.28
**1644789**	DUSP5P	4.57E-04	13.04	29.33	2.25

**1385341**	NRTN	3.22E-03	2.91	6.46	2.22
**1390964**	TBC1D26	1.67E-04	17.29	38.06	2.20
**1396584**	C2orf51	1.17E-03	3.18	6.99	2.20
**1380400**	SFMBT2	3.63E-05	34.74	75.52	2.17
**1378091**	OR10G8	6.91E-03	3.14	6.79	2.16
**1391576**	NMS	2.57E-03	3.05	6.48	2.12
**1394244**	FOXJ1	9.96E-03	6.45	13.69	2.12
**1385519**	UNC13D	2.94E-03	8.83	18.71	2.12
**1386067**	TTK	5.62E-03	4.9	10.38	2.12
**1396609**	KRTAP12-3	5.75E-03	4.29	9.04	2.11
**1389439**	NPNT	6.47E-03	4.9	10.27	2.10
**1390353**	SLIT3	1.15E-05	35.7	74.53	2.09
**1390373**	ZC3H12A	6.17E-03	5.55	11.52	2.08
**1387613**	LRRIQ1	9.75E-03	4.59	9.51	2.07
**1643558**	WNT7B	1.36E-04	26.18	53	2.02
**1378264**	ABCD1	3.09E-04	8.56	17.24	2.01
**1387375**	RNF17	2.89E-04	4.09	8.22	2.01
**1643454**	ZNF831	1.00E-07	58.2	116.87	2.01
**1645897**	SLAMF6	2.94E-03	7.71	15.41	2.00
**1386335**	MST1R	5.79E-03	3.89	7.77	2.00
**1396398**	RAB7B	1.93E-03	35.03	69.57	1.99
**1392878**	ESR1	3.29E-04	6.1	12.06	1.98
**1380961**	HIST1H3H	1.84E-03	5.97	11.77	1.97
**1377123**	NR2E3	1.93E-03	2.33	4.56	1.96
**1393105**	PCOLCE2	3.31E-03	25.53	49.55	1.94
**1392814**	ANKRD1	3.85E-03	3.86	7.45	1.93
**1379339**	TCAP	6.19E-03	9.43	18.13	1.92
**1385239**	LY6G5C	5.22E-03	4.42	8.48	1.92
**1386218**	GDAP2	8.95E-03	8.57	16.43	1.92
**1378314**	SEZ6	7.95E-04	163.6	311.68	1.91
**1390012**	CAMK2D	4.44E-04	22.85	43.4	1.90
**1376932**	CAMK2D	1.60E-05	51.18	96.89	1.89
**1395261**	GHR	6.15E-03	29.44	55.53	1.89
**1389736**	KEAP1	6.62E-03	4.37	8.24	1.89
**1389341**	KIR2DS5	8.20E-03	5.12	9.61	1.88
**1389848**	LOC440944	1.94E-03	5.43	10.09	1.86
**1388753**	CHST6	7.69E-03	45.14	83.87	1.86
**1385198**	WFIKKN1	3.30E-03	9.74	18.09	1.86
**1377282**	HRK	8.98E-04	134.72	248.46	1.84
**1379097**	SERTAD1	8.11E-03	185.59	341.96	1.84
**1380799**	C6orf206	3.73E-03	13.06	23.99	1.84
**1380073**	OLFML2B	1.56E-04	29.16	53.41	1.83
**1646393**	RHOD	9.73E-03	11.08	20.24	1.83
**1377905**	FOXD4L2	4.41E-03	6.01	10.89	1.81
**1387894**	TTYH3	2.69E-04	212.12	382.37	1.80
**1379314**	RBMY1E	2.60E-03	4.77	8.57	1.80
**1646097**	ATF3	7.43E-03	55.24	98.65	1.79
**1390529**	VPS37B	6.00E-07	131.63	233.76	1.78

**1386496**	SYTL2	1.08E-03	28.72	50.92	1.77
**1378240**	TNRC4	3.08E-05	484.61	858.09	1.77
**1394151**	C10orf85	2.76E-03	53.21	93.77	1.76
**1384389**	CEL	3.74E-03	26.6	46.82	1.76
**1381301**	PYGO2	8.19E-05	39.92	70.25	1.76
**1386926**	WSCD1	3.46E-03	50.18	88.15	1.76
**1380102**	TMEM26	1.47E-03	18.68	32.65	1.75
**1395835**	KCNN3	3.50E-04	115.81	200.64	1.73
**1643814**	MARCH3	7.30E-03	58.17	100.64	1.73
**1386370**	SBNO2	9.52E-03	12.46	21.55	1.73
**1391059**	TACC3	8.64E-03	8.88	15.34	1.73
**1644175**	KCNH2	4.03E-03	4.75	8.16	1.72
**1380951**	PKD2L1	1.14E-03	35.38	60.68	1.72
**1377210**	JSRP1	1.21E-03	15.19	26.05	1.71
**1383148**	SHF	2.01E-04	21.79	37.31	1.71
**1377621**	C5orf20	3.33E-03	8.77	14.98	1.71
**1396157**	KIF23	7.22E-03	7.78	13.27	1.71
**1378478**	SRGAP1	6.67E-03	55.51	94.05	1.69
**1388928**	TSPAN16	5.44E-03	9.28	15.69	1.69
**1393008**	NDST4	5.31E-03	9.08	15.23	1.68
**1388404**	C1orf106	6.11E-04	16.82	28.2	1.68
**1396692**	ODF2L	2.48E-03	12.74	21.35	1.68
**1385941**	DNASE1L2	9.15E-03	24.87	41.54	1.67
**1387531**	ZDHHC23	1.01E-03	78.87	131.64	1.67
**1377496**	PLOD3	6.50E-03	222.42	370.5	1.67
**1381452**	MOV10	5.25E-04	47.26	78.68	1.66
**1385522**	ZDHHC18	8.01E-03	35.48	58.9	1.66
**1390850**	ASCL2	3.78E-03	35.33	58.47	1.65
**1382522**	SDF2L1	9.20E-03	436.35	721.88	1.65
**1646449**	RBM39	4.95E-04	427.51	706.75	1.65
**1380910**	IKBKE	2.42E-03	15.89	26.26	1.65
**1384438**	RNF150	2.40E-06	509.1	840.88	1.65
**1393191**	PLOD2	9.34E-03	205.51	339.01	1.65
**1384640**	PTCHD1	4.80E-06	230.67	380.16	1.65
**1381564**	DMWD	7.27E-04	93.15	153.43	1.65
**1378155**	OTOF	8.50E-03	26.28	43.24	1.65
**1395625**	TSPAN32	2.23E-03	8.5	13.93	1.64
**1377253**	TAOK2	7.20E-04	106.9	174.77	1.63
**1391016**	STH	1.02E-03	53.05	86.61	1.63
**1391251**	FILIP1	9.01E-03	71.46	116.62	1.63
**1395339**	SYT14	6.31E-03	16	26.06	1.63
**1387149**	C16orf55	1.25E-03	15.78	25.67	1.63
**1390983**	TP53I11	8.43E-03	22.88	36.96	1.62
**1389242**	DDAH2	5.62E-03	30.67	49.41	1.61
**1388459**	DUSP8	3.90E-03	437.78	704.64	1.61
**1396184**	SLC2A4RG	2.59E-03	36.54	58.79	1.61
**1395760**	AKAP8L	1.45E-03	157.86	253.75	1.61
**1381335**	BIRC3	2.54E-04	24.52	39.37	1.61

**1388604**	ABCA1	8.83E-03	336.09	538.63	1.60
**1382933**	ZNF609	1.91E-04	88.78	142.02	1.60
**1381124**	WDR66	1.39E-03	24.35	38.95	1.60
**1379925**	TMEM137	5.18E-04	59.55	94.88	1.59
**1396745**	BLM	4.32E-04	25.96	41.31	1.59
**1646238**	AGBL5	2.26E-04	24.35	38.73	1.59
**1646053**	ATXN2L	1.45E-03	34.17	54.23	1.59
**1395295**	HBEGF	1.07E-03	99.4	157.33	1.58
**1644267**	HYDIN	4.70E-03	10.81	17.06	1.58
**1386581**	SH2B2	3.02E-03	15.54	24.52	1.58
**1389471**	LMAN2L	2.47E-03	148.17	233.47	1.58
**1396258**	APOE	3.04E-03	3392.57	5334.34	1.57
**1384359**	FLJ41649	1.05E-03	19.15	29.99	1.57
**1392897**	ASPRV1	4.67E-04	36.63	57.35	1.57
**1379298**	ZBTB40	1.30E-03	107.17	167.7	1.56
**1378251**	MAPK7	1.29E-04	52.18	81.48	1.56
**1379043**	HTR3A	4.07E-03	19.12	29.78	1.56
**1377491**	DUSP18	8.71E-04	91.05	141.8	1.56
**1394107**	KLKB1	9.27E-03	32.42	50.42	1.56
**1644439**	GNB4	1.47E-03	24.03	37.23	1.55
**1377236**	POLN	6.51E-04	36.51	56.52	1.55
**1393484**	MEIS3	4.37E-03	59.32	91.8	1.55
**1395287**	BARD1	4.09E-04	55.67	86.08	1.55
**1646902**	ODZ3	2.98E-05	494.29	764.18	1.55
**1387066**	LPPR2	9.64E-04	237.16	366.14	1.54
**1644263**	HYDIN	1.38E-03	8.16	12.59	1.54
**1387595**	KIF12	7.12E-03	10.64	16.41	1.54
**1377479**	PCDHGA12	7.20E-03	11.89	18.32	1.54
**1390503**	GRAMD2	5.81E-03	29.41	45.29	1.54
**1378468**	JUN	2.86E-03	827.17	1273.13	1.54
**1388632**	LRFN4	1.80E-04	79.44	122.19	1.54
**1380401**	PRR5	9.49E-04	94.27	144.92	1.54
**1391647**	NEK6	8.83E-03	84.51	129.83	1.54
**1645965**	SCN9A	9.56E-03	10.02	15.38	1.53
**1383727**	SEPT6	3.54E-03	12.68	19.45	1.53
**1378636**	SEPN1	1.70E-03	280.06	429.11	1.53
**1386027**	FLJ34503	6.78E-03	7.03	10.77	1.53
**1646418**	RFX2	5.93E-03	24.25	37.13	1.53
**1393062**	ELK1	1.82E-03	584.29	894.62	1.53
**1394788**	ABCA7	3.18E-03	24.92	38.06	1.53
**1381384**	C20orf117	1.20E-04	93.06	142.07	1.53
**1391719**	RBM14	5.66E-04	747.24	1138.88	1.52
**1381021**	ZDHHC14	9.60E-06	342.06	520.63	1.52
**1380834**	RAI14	4.12E-03	123.8	188.15	1.52
**1385485**	MYO9B	4.53E-03	79.53	120.8	1.52
**1392438**	ZFHX3	3.85E-03	83.52	126.86	1.52
**1379726**	CACNA1C	1.58E-04	96.54	146.36	1.52
**1385032**	MKL1	4.60E-04	118.22	179.05	1.51

**1379582**	SIN3B	7.87E-04	206.91	313.27	1.51
**1386058**	PTGIS	3.35E-03	13.5	20.43	1.51
**1391348**	SOX4	4.73E-04	33.55	50.7	1.51
**1388607**	LTBP4	1.95E-03	62.22	94	1.51
**1646626**	POL3S	1.54E-03	52.58	79.42	1.51
**1380159**	AHDC1	2.62E-03	78.67	118.36	1.50
**1392542**	TRAF2	2.22E-03	38.14	57.36	1.50
**1382685**	ANKRD57	5.75E-04	141.73	212.84	1.50
**1387101**	EIF2S2	5.45E-04	45.61	68.49	1.50
**1379896**	SOLH	4.52E-03	20.1	30.17	1.50
**1383168**	ZNF202	6.93E-03	33.17	49.75	1.50
**1383490**	C4orf23	2.40E-03	10.61	15.91	1.50
**1383150**	TM4SF5	1.25E-05	3.89	0.095	0.02
**1378429**	CASP4	4.64E-03	4.94	0.28	0.06
**1384541**	NR4A3	7.08E-03	3.35	0.2	0.06
**1645665**	TAS1R3	4.00E-03	3.25	0.26	0.08
**1385806**	PHOSPHO2	6.49E-04	7.35	0.58	0.08
**1646640**	PLEKHG5	7.76E-03	4.81	0.89	0.19
**1391600**	PVALB	7.14E-04	1132.23	232.49	0.21
**1381684**	CTRB1	4.07E-04	3.98	0.86	0.22
**1645227**	C3orf57	1.70E-06	28.79	6.2	0.22
**1397377**	KRT74	8.79E-03	4.16	1	0.24
**1384366**	MORF4L1	2.10E-06	22.81	5.86	0.26
**1380551**	CSN1S1	1.50E-03	21.67	5.77	0.27
**1388809**	STEAP4	8.27E-03	4.41	1.17	0.27
**1644941**	CTXN3	1.21E-04	156.01	43.19	0.28
**1388744**	RTN3	4.75E-04	168.34	55.43	0.33
**1383406**	MAP1B	2.99E-04	1373.75	465.13	0.34
**1379809**	SCN1B	3.43E-05	395.32	135.32	0.34
**1645458**	TTN	5.81E-03	5.88	2.06	0.35
**1377806**	INHBC	4.97E-03	4.31	1.54	0.36
**1388201**	RNASE4	1.04E-03	5.78	2.11	0.37
**1382293**	AMY1B	3.10E-03	12.37	4.66	0.38
**1644573**	FLJ32679	7.92E-03	8.31	3.13	0.38
**1394761**	FLJ41821	6.81E-03	5.14	1.97	0.38
**1378185**	TPD52	1.80E-03	37.74	14.45	0.38
**1397061**	VAMP1	2.68E-05	2937.83	1133.16	0.39
**1388130**	USP12	3.71E-03	12.72	4.9	0.39
**1388064**	ATP6V1H	5.29E-05	38.31	14.88	0.39
**1397381**	HSD11B1	7.38E-05	86.28	33.98	0.39
**1377231**	C9orf72	4.11E-05	33.86	13.4	0.40
**1645390**	VAMP1	9.60E-05	151.86	60.53	0.40
**1377361**	NEFH	8.20E-04	1878.39	751.49	0.40
**1382338**	JAKMIP1	8.83E-03	12.48	5.04	0.40
**1392346**	SLC25A40	2.36E-05	105.55	42.53	0.40
**1386866**	MLN	2.56E-03	9.6	3.91	0.41
**1385278**	HAPLN4	3.44E-03	979.1	398.17	0.41
**1396029**	RFC3	2.63E-03	16.73	6.84	0.41

**1645135**	CCNL2	6.39E-03	85.56	35	0.41
**1396358**	TRIM37	3.19E-04	230.18	94.54	0.41
**1381672**	RTN1	5.73E-03	2435.69	1009.28	0.41
**1379348**	SLC22A18AS	8.55E-04	83.92	34.99	0.42
**1646886**	OPN3	2.77E-03	93.58	39.14	0.42
**1394677**	SHD	3.97E-03	219.92	92.87	0.42
**1389657**	HTATSF1	8.83E-03	5.05	2.13	0.42
**1384214**	HTR5A	1.41E-03	37.42	15.94	0.43
**1377745**	CYP26C1	6.03E-03	9.32	3.96	0.42
**1389358**	TSC22D3	1.06E-03	25.95	11.19	0.43
**1383364**	ASB6	9.18E-03	9.34	4.05	0.43
**1383314**	RRAGB	3.35E-03	43.17	18.81	0.44
**1383903**	KCNC1	3.61E-05	53.96	23.5	0.44
**1383091**	ARNTL2	3.68E-04	8.27	3.59	0.43
**1645734**	SSX4	8.04E-03	5.26	2.3	0.44
**1384482**	FBXO9	1.05E-03	97.7	43.02	0.44
**1378328**	HSD11B1	4.20E-05	114.47	50.86	0.44
**1646168**	ANKRD56	8.79E-03	9.87	4.38	0.44
**1397081**	PCP4	1.74E-03	2761.72	1231.98	0.45
**1394203**	ELMO1	2.82E-03	193.36	86.86	0.45
**1392178**	FGF14	6.84E-04	153.6	69.11	0.45
**1390880**	FBXW7	9.08E-04	212.86	95.76	0.45
**1377415**	EMILIN3	1.18E-03	29.12	13.09	0.45
**1393284**	GDAP1	8.17E-04	99.63	45.13	0.45
**1387336**	CABP1	7.35E-04	128.45	58.12	0.45
**1644354**	HAPLN1	3.36E-03	5.43	2.45	0.45
**1377573**	STAT4	5.55E-03	628.75	286.44	0.46
**1384817**	TRUB1	1.16E-03	71.86	32.64	0.45
**1381170**	SEPT6	4.47E-03	8.11	3.73	0.46
**1386613**	FBXO44	8.88E-03	9.47	4.34	0.46
**1384744**	OPN3	2.84E-03	367.22	169.53	0.46
**1646555**	PRKACB	8.32E-04	193.9	89.37	0.46
**1384092**	MAGED1	2.06E-05	22.6	10.41	0.46
**1390840**	SPANXN5	9.77E-03	4.9	2.27	0.46
**1384585**	PDCD10	5.71E-04	49.68	22.99	0.46
**1388399**	SNRPN	5.42E-04	11.39	5.32	0.47
**1383057**	SCAMP1	4.37E-04	138.04	64.67	0.47
**1389485**	BTBD1	1.23E-03	196.98	93.04	0.47
**1381015**	IRX3	4.56E-03	29.44	13.86	0.47
**1391313**	DLGAP1	1.90E-03	43.46	20.58	0.47
**1396459**	VPS16	8.31E-04	34.89	16.64	0.48
**1386122**	ZDHHC13	9.12E-04	10.74	5.14	0.48
**1388958**	SPIN2A	7.93E-04	19.99	9.72	0.49
**1396482**	ASPH	2.61E-03	78.19	37.94	0.49
**1392288**	MAGED2	1.03E-05	49.62	24.05	0.48
**1394580**	KCNIP4	4.28E-03	350.34	171.45	0.49
**1381675**	AMACR	4.68E-03	88.35	43.43	0.49
**1378151**	KCNC2	1.85E-03	109.02	53.9	0.49

**1396881**	KCNA1	7.82E-03	32.56	16.17	0.50
**1386183**	MACROD2	7.11E-03	64.48	32.01	0.50
**1385252**	ELAVL2	8.73E-04	189.21	94.89	0.50
**1390841**	NEFM	7.46E-04	3599.84	1804.71	0.50
**1393415**	NUDT18	8.42E-03	177.13	88.79	0.50
**1390147**	CNTN4	5.21E-03	108.29	54.7	0.51
**1396744**	SYCE1	9.65E-03	24.69	12.56	0.51
**1644062**	LETMD1	9.47E-04	108.65	55.4	0.51
**1389580**	MAT2B	4.92E-04	30.7	15.65	0.51
**1396319**	SCP2	1.79E-04	132.41	68.05	0.51
**1385249**	CCNDBP1	1.04E-04	39.68	20.38	0.51
**1383621**	ATP5S	5.93E-03	50.66	25.99	0.51
**1382854**	NRG1	1.58E-03	35.61	18.26	0.51
**1380538**	NOMO3	9.44E-04	46.09	23.62	0.51
**1645798**	SNAP25	3.04E-03	5513.37	2822.67	0.51
**1396998**	PPM1A	6.33E-05	102.11	52.24	0.51
**1645176**	CAMK2G	1.55E-03	663	342.17	0.52
**1394339**	SEPT5	2.76E-03	64.22	33.13	0.52
**1394136**	EEF1B2	3.19E-04	139.82	72.1	0.52
**1383049**	CNDP1	2.08E-03	296.49	152.51	0.51
**1390148**	CADPS	3.21E-03	170.01	88.17	0.52
**1395454**	GMPR2	4.85E-03	31.4	16.26	0.52
**1643504**	ZNF226	8.91E-03	39.76	20.72	0.52
**1387885**	LYNX1	7.55E-04	37.69	19.64	0.52
**1390772**	DLD	1.38E-04	282.69	148.02	0.52
**1383310**	FBLN7	5.08E-03	146.5	76.66	0.52
**1381201**	TCP1	4.71E-03	90.13	47.47	0.53
**1383377**	RBM11	1.97E-03	50.09	26.38	0.53
**1387456**	TNNT2	1.74E-03	229.54	120.78	0.53
**1384533**	HK1	4.64E-03	40.18	21.3	0.53
**1394789**	ELF3	8.39E-03	15.47	8.17	0.53
**1382408**	CLCN4	7.64E-03	72.8	38.77	0.53
**1643625**	NCKAP1	7.32E-03	203.26	108.04	0.53
**1646680**	PHYH	1.79E-05	157.59	83.7	0.53
**1391061**	MKKS	1.08E-03	47.09	25.23	0.54
**1386691**	EYA3	6.97E-03	15.95	8.54	0.54
**1644158**	KCNQ5	3.86E-04	24.94	13.35	0.54
**1388266**	CCT6A	1.17E-03	152.42	81.57	0.54
**1392537**	PSMG1	7.98E-04	172.02	91.9	0.53
**1394514**	CDC42	2.85E-03	108.98	58.22	0.53
**1382774**	RWDD1	1.42E-03	194.41	104.68	0.54
**1383953**	GPLD1	2.87E-03	15.15	8.15	0.54
**1382226**	RFC2	2.96E-04	34.71	18.62	0.54
**1380702**	MAEA	5.21E-03	100.59	54.42	0.54
**1378095**	PLEKHB2	2.43E-03	358.92	194.08	0.54
**1392403**	LOC729399	5.28E-04	73.54	39.68	0.54
**1388812**	RTN3	1.66E-03	95.31	51.38	0.54
**1377588**	PRDX2	1.09E-04	725.72	394.91	0.54

**1645074**	CEP170	6.16E-04	21.79	11.85	0.54
**1396080**	TRIM37	1.69E-03	1096.66	595.38	0.54
**1389956**	CBLN2	1.90E-03	812.75	440.54	0.54
**1389253**	ABHD11	1.70E-04	47.85	26.21	0.55
**1384540**	TSC22D1	5.55E-04	1775.21	972.18	0.55
**1390063**	NCOA2	3.48E-03	23.21	12.7	0.55
**1646458**	RAP1GDS1	2.91E-03	114.15	62.41	0.55
**1391132**	NME2	2.81E-03	39.72	21.68	0.55
**1390986**	PCGF6	2.72E-03	23.21	12.77	0.55
**1382390**	SULT1A1	7.82E-04	203.62	111.63	0.55
**1645392**	USP33	1.63E-04	79.46	44	0.55
**1394003**	GPR20	6.14E-03	11.1	6.14	0.55
**1386149**	DMXL2	5.68E-03	216.39	119.55	0.55
**1381297**	C7orf46	2.58E-03	33.78	18.63	0.55
**1384966**	PPP1R1B	7.52E-05	20.1	11.19	0.56
**1382971**	GABRB2	1.07E-03	11.41	6.35	0.56
**1393995**	CDC42SE2	9.55E-04	49.93	27.78	0.56
**1380193**	GABRA1	7.35E-03	490.59	272.89	0.56
**1378972**	ESRRG	8.85E-03	250.57	138.91	0.55
**1396211**	ENSA	7.90E-03	325.25	181.89	0.56
**1385763**	C4orf38	5.74E-03	14.3	7.99	0.56
**1645686**	SYNJ1	1.38E-03	78.33	43.73	0.56
**1391215**	SNRPN	3.85E-03	4403.59	2458.11	0.56
**1395814**	HSD17B11	9.67E-03	32.5	18.14	0.56
**1396225**	TSC22D1	5.86E-03	76.81	42.83	0.56
**1394742**	HNRNPA1	1.10E-03	57.66	32.34	0.56
**1396774**	UQCRC2	5.03E-03	288.15	163.07	0.57
**1389204**	IDI1	8.30E-03	102.5	57.91	0.56
**1395793**	PFKFB2	1.33E-03	74.03	41.74	0.56
**1379619**	ZNHIT3	9.16E-04	89.7	50.57	0.56
**1397134**	BEX5	4.91E-03	1916	1091.54	0.57
**1644086**	LAMA2	3.54E-04	70.07	39.83	0.57
**1396282**	GCC2	3.46E-04	32.52	18.46	0.57
**1382038**	SRPK2	1.75E-03	73.3	41.55	0.57
**1646773**	PARG	3.11E-03	35.28	20.2	0.57
**1392255**	AUH	3.67E-04	474.03	271.08	0.57
**1388045**	PSMF1	2.46E-03	7.91	4.52	0.57
**1393503**	DYNC2LI1	3.17E-03	270.2	154.17	0.57
**1646305**	ABHD11	7.58E-05	40.06	22.85	0.57
**1393805**	ATP5C1	1.96E-03	764.17	435.87	0.57
**1646060**	ATP9A	1.08E-05	35.61	20.51	0.58
**1378618**	IQSEC3	3.11E-03	33.41	19.24	0.58
**1393661**	RPH3A	8.01E-03	143.09	82.37	0.58
**1645438**	UBE2E3	1.25E-03	285.89	164.51	0.58
**1389897**	GPR150	9.97E-03	13.62	7.82	0.57
**1646189**	ANKHD1	8.24E-03	20.06	11.5	0.57
**1387840**	B4GALT6	7.53E-03	101.35	58.09	0.57
**1394862**	PSMG1	3.34E-03	270.29	154.91	0.57

**1381809**	F8	5.65E-05	23.5	13.6	0.58
**1389211**	PSMA1	1.61E-03	193.24	111.83	0.58
**1382748**	C14orf138	3.32E-04	164.8	95.28	0.58
**1393565**	KIAA0251	7.02E-03	57.27	33.11	0.58
**1384853**	SCN1A	8.93E-03	235.14	135.57	0.58
**1646179**	ANKRD29	4.53E-05	312.11	179.93	0.58
**1386265**	FCRLB	8.82E-04	134.64	78.5	0.58
**1381882**	GSTM4	1.33E-04	35.97	20.96	0.58
**1385080**	TUBGCP5	2.51E-03	203.11	118.11	0.58
**1378542**	PDE6H	4.40E-03	19.05	11.07	0.58
**1379429**	TMEM27	2.26E-03	20.71	12.09	0.58
**1644783**	DYRK1A	2.56E-03	151.47	88.33	0.58
**1391068**	FGF9	2.89E-04	699.45	412.39	0.59
**1386859**	CHGA	1.28E-03	2167.22	1276.91	0.59
**1390261**	SLC25A3	1.29E-03	64.31	37.84	0.59
**1391134**	ATP5G1	3.70E-03	116.78	68.64	0.59
**1376926**	GGH	5.69E-03	29.03	17.04	0.59
**1386401**	TXNDC9	7.80E-03	80.78	47.41	0.59
**1644113**	KLHDC9	1.41E-03	301.12	176.66	0.59
**1645132**	CCNC	5.63E-03	194.5	115.29	0.59
**1377965**	RAB37	3.75E-03	145.64	86.27	0.59
**1380760**	GAD1	2.06E-03	36.73	21.71	0.59
**1385135**	SLFN5	3.38E-03	19.02	11.24	0.59
**1383198**	C3orf57	1.07E-03	21.82	12.88	0.59
**1383957**	ANO5	1.65E-04	16.12	9.51	0.59
**1395364**	PRKCB1	7.69E-03	725.07	432.85	0.60
**1385946**	TRIM23	4.10E-03	542.91	324.06	0.60
**1393575**	RELL2	5.00E-03	226.75	135.21	0.60
**1643778**	MEIS3P1	4.00E-06	185.75	110.71	0.60
**1645405**	USP14	5.60E-04	225.49	134.36	0.60
**1383320**	OR2W3	6.70E-03	20.75	12.35	0.60
**1645878**	SLC22A18	1.92E-03	107.18	63.74	0.59
**1397293**	CASD1	2.62E-03	208.48	123.94	0.59
**1392824**	MATR3	3.61E-03	205.11	123	0.60
**1384952**	ASB3	5.53E-03	134.4	80.55	0.60
**1383809**	TBRG4	7.28E-03	16.37	9.81	0.60
**1646379**	RNF14	5.79E-04	397.21	237.8	0.60
**1643949**	LOC653803	7.13E-03	24.51	14.67	0.60
**1384568**	UQCC	9.39E-03	47.23	28.25	0.60
**1377086**	USP16	1.70E-03	139.78	83.57	0.60
**1388599**	SLC25A12	1.07E-03	668.23	399.5	0.60
**1644405**	GRB14	4.73E-03	127.27	76.06	0.60
**1383369**	TM2D3	1.65E-03	102.72	61.38	0.60
**1384726**	CUL4B	9.00E-04	27.7	16.74	0.60
**1378701**	ACADSB	8.30E-04	55.75	33.68	0.60
**1381537**	UBLCP1	8.96E-03	280.17	168.99	0.60
**1386064**	HACL1	4.14E-04	520.37	313.47	0.60
**1380649**	TASP1	8.81E-03	104.44	62.89	0.60

**1383095**	AADAT	2.02E-03	29.15	17.53	0.60
**1378751**	EID2	2.40E-03	360.35	216.55	0.60
**1383032**	PFAAP5	6.48E-04	319.08	191.73	0.60
**1644293**	HS6ST2	2.81E-03	68.59	41.2	0.60
**1645849**	SLC4A8	1.28E-04	44.33	26.94	0.61
**1390021**	APITD1	1.92E-03	72.69	44.15	0.61
**1384417**	PCDH8	4.99E-04	57.99	35.19	0.61
**1391766**	GOSR2	7.06E-03	23.49	14.25	0.61
**1384834**	ASAH1	1.44E-03	190.55	115.59	0.61
**1390007**	PRKACB	5.88E-03	11.23	6.81	0.61
**1384718**	UQCRB	5.78E-04	102.35	62.06	0.61
**1644104**	KPNA2	6.23E-03	63.14	38.24	0.61
**1385204**	TM6SF1	3.62E-04	377.54	228.64	0.61
**1394016**	PPA2	5.65E-04	387.15	236.7	0.61
**1383493**	KAZALD1	5.02E-03	15.92	9.73	0.61
**1394315**	LRRC24	6.98E-03	18.5	11.3	0.61
**1644180**	KCMF1	6.86E-04	264.21	161.38	0.61
**1396933**	GPR89A	2.33E-04	87	53.09	0.61
**1391407**	TTBK2	3.42E-03	35.86	21.85	0.61
**1392679**	CLU	1.37E-03	63.11	38.43	0.61
**1387919**	NEFL	5.70E-03	2247.19	1366.15	0.61
**1396992**	P2RX5	3.29E-03	21.6	13.26	0.61
**1394413**	CHM	6.32E-03	31.72	19.47	0.61
**1646673**	PIAS2	2.30E-03	44.66	27.41	0.61
**1389335**	SYTL2	1.93E-03	35.76	21.89	0.61
**1381182**	RTDR1	4.08E-03	10.23	6.33	0.62
**1385959**	CFP	1.56E-04	11.53	7.13	0.62
**1644493**	GABRG2	8.96E-03	940.4	581.4	0.62
**1644243**	IGSF3	1.21E-03	28.97	17.91	0.62
**1390976**	TACR2	5.02E-03	18.72	11.57	0.62
**1378737**	NDUFA10	1.89E-03	218.81	135.13	0.62
**1396353**	NPPC	2.24E-03	22.97	14.17	0.62
**1383382**	GLS2	3.34E-03	180.35	111.16	0.62
**1646521**	PSMC4	4.67E-04	220.06	135.59	0.62
**1394948**	SLC6A15	2.21E-03	26.31	16.39	0.62
**1388656**	CHMP5	2.93E-03	644.98	401.42	0.62
**1384312**	MOCS2	4.99E-03	158.89	98.75	0.62
**1386433**	KCNN2	1.39E-03	48.23	29.96	0.62
**1380704**	TAF9	7.87E-03	30.7	19.07	0.62
**1643544**	XPOT	7.13E-04	59.1	36.69	0.62
**1646751**	PCDHA3	2.71E-03	30.15	18.71	0.62
**1377516**	SPG21	6.49E-03	28.62	17.91	0.63
**1387715**	PIK3CB	1.80E-03	143.13	89.56	0.63
**1380547**	PIP5K1B	6.53E-03	43.24	27	0.62
**1388504**	PREI3	2.45E-03	71.48	44.57	0.62
**1646372**	RNF38	8.61E-03	118.14	73.65	0.62
**1387009**	RABL3	7.57E-04	76.03	47.96	0.63
**1392041**	YEATS4	4.43E-03	110.27	69.55	0.63

**1644690**	EXTL2	3.07E-04	286.31	180.53	0.63
**1377319**	C4orf27	5.17E-04	288.25	181.74	0.63
**1392332**	NCKIPSD	9.86E-04	468.57	294.84	0.63
**1380195**	SUCLA2	1.12E-03	850.94	534.52	0.63
**1382239**	ADARB1	2.53E-03	44.67	28.05	0.63
**1391554**	MRS2	6.20E-03	61.45	38.58	0.63
**1384764**	PGAM1	2.73E-03	1615.74	1013.9	0.63
**1388856**	WWOX	2.75E-03	45.54	28.9	0.63
**1392291**	ATRX	4.25E-03	25.72	16.31	0.63
**1646088**	ATG4B	2.93E-03	296.43	187.9	0.63
**1383868**	RPL6	4.15E-03	215.82	136.57	0.63
**1390049**	GRM7	8.14E-03	25.73	16.27	0.63
**1386582**	GRIA3	8.76E-04	72.43	45.8	0.63
**1397086**	TSPAN3	8.08E-03	1007.04	643.43	0.64
**1385405**	FAF1	8.07E-05	102.31	65.33	0.64
**1387911**	SLC2A11	3.29E-03	122.99	78.51	0.64
**1383598**	WAC	3.65E-03	28.11	17.9	0.64
**1381722**	RASGRF1	9.58E-03	57.43	36.57	0.64
**1393931**	SERF1A	8.29E-03	24.5	15.59	0.64
**1645759**	SPIN2B	3.26E-03	61.95	39.39	0.64
**1644331**	HIGD1A	9.70E-04	2039.96	1296.99	0.64
**1388468**	SRP72	2.77E-03	176.01	111.84	0.64
**1377405**	FASTKD3	2.47E-03	59.79	37.99	0.64
**1380626**	TBRG1	6.89E-03	32.74	21.04	0.64
**1386044**	OXCT2	2.69E-03	105.97	68.03	0.64
**1382399**	GRIN2A	3.71E-03	79.65	51.11	0.64
**1645950**	SEDLP	2.06E-03	146.33	93.89	0.64
**1387936**	BMP2K	4.36E-04	38.59	24.76	0.64
**1380351**	TCEAL6	5.87E-03	963.91	618.38	0.64
**1387115**	GSTM2	1.27E-04	2788.06	1788.44	0.64
**1395633**	FAM80A	8.49E-03	103.27	66.19	0.64
**1386173**	UGP2	2.69E-04	1101.59	705.36	0.64
**1396788**	PARK2	7.08E-03	34.41	22.03	0.64
**1381771**	CHMP2A	1.66E-03	135.96	87.01	0.64
**1381307**	FGF12	5.64E-03	645.76	413.17	0.64
**1385896**	RAD51C	2.69E-03	202.96	131.26	0.65
**1388213**	AMPH	4.53E-03	1450.49	937.83	0.65
**1389131**	CORO2A	4.22E-04	72.51	46.86	0.65
**1383517**	PMPCB	5.79E-04	800.65	516.16	0.64
**1390069**	RAN	5.19E-03	972.53	626.78	0.64
**1392687**	SLC37A3	1.52E-03	36.51	23.49	0.64
**1643827**	MAP3K7	3.27E-03	70.58	45.41	0.64
**1382558**	AMACR	4.33E-03	100.41	64.6	0.64
**1389027**	ARMC8	1.57E-03	175.36	112.8	0.64
**1645251**	C20orf100	8.33E-03	1594.76	1038.09	0.65
**1382160**	C11orf49	5.03E-04	57.59	37.31	0.65
**1392667**	CASC4	1.70E-03	66.77	43.25	0.65
**1645767**	SPG21	1.35E-03	254.75	164.94	0.65

**1377987**	GPATCH4	2.54E-03	189.83	122.88	0.65
**1396384**	RAB25	9.52E-03	18.95	12.41	0.65
**1377343**	MAD2L1BP	2.49E-03	171.99	112.6	0.65
**1392460**	GSTM1	5.83E-05	1614.7	1055.13	0.65
**1379210**	ATP1A1	1.92E-03	2624.35	1714.87	0.65
**1396209**	CCNC	1.99E-03	131.68	85.91	0.65
**1381142**	CDKN3	6.77E-03	51.78	33.73	0.65
**1644819**	DNAJB12	4.45E-04	59.15	39	0.66
**1386727**	CHMP2B	1.19E-03	282.81	186.44	0.66
**1644382**	GTPBP10	1.36E-03	40.63	26.72	0.66
**1645578**	TMEM70	4.69E-03	30.84	20.28	0.66
**1644260**	IDH3B	2.66E-03	1123.66	738.65	0.66
**1388773**	FBXO17	5.58E-03	9.48	6.23	0.66
**1377548**	NDUFS2	2.23E-03	55.77	36.65	0.66
**1394263**	TIPRL	1.63E-03	323.2	212.29	0.66
**1393632**	PDE4DIP	2.17E-03	107.36	70.44	0.66
**1385842**	C12orf24	2.21E-03	707.34	463.85	0.66
**1646629**	PMS2	9.19E-04	37.79	24.78	0.66
**1378726**	GNG13	4.22E-03	31.42	20.86	0.66
**1378883**	SNTG1	7.24E-03	93.12	61.8	0.66
**1391071**	NME2	1.06E-03	176.51	117.14	0.66
**1384632**	TXNL4A	4.34E-03	89.08	59.11	0.66
**1391498**	CLIP4	1.34E-03	409.93	271.73	0.66
**1391997**	HMBS	1.32E-03	53.37	35.36	0.66
**1386532**	RET	4.66E-04	48	31.79	0.66
**1645951**	SEH1L	1.88E-03	157.26	104.13	0.66
**1645444**	UBA3	7.15E-03	249.73	165.25	0.66
**1380366**	CRYBA2	3.74E-03	24.2	16	0.66
**1644408**	GRIA3	5.58E-05	130.93	86.54	0.66
**1381048**	FEZ1	6.10E-03	771.82	509.92	0.66
**1392364**	TCEAL4	4.43E-03	60.35	39.87	0.66
**1644583**	FKBP1B	5.57E-03	65.01	42.94	0.66
**1377065**	MRFAP1L1	9.12E-03	66.6	43.96	0.66
**1646131**	ARHGAP9	9.95E-03	42.1	28.04	0.67
**1378301**	GPR176	3.71E-04	44.09	29.36	0.67
**1382720**	NBPF14	8.23E-03	42.44	28.23	0.67
**1383914**	C6orf65	3.59E-03	1183.37	787.05	0.67

4) List of genes differentially expressed between F-NAGM and CTR samples

p<0.01 Fold Change≥1.5 vs Control group

(Fold Change F- NAGM/CTR ≥1.5= Up-regulated in F-NAGM)

(Fold Change F- NAGM/CTR ≤0.66= Down-regulated in F-NAGM)

F- NAGM vs CTR					
			Geometric Mean of Intensity		Fold Change
**UniqueID**	**Symbol**	**Parametric p-value**	**CTR**	**F- NAGM**	**F-NAGM/CTR**
**1381814**	HLA-DRB5	2.31E-04	6.96	134.09	19.27
**1389039**	HLA-DRB1	5.50E-04	4.93	75.32	15.28
**1392290**	SERPINA3	6.06E-03	277.4	2285.49	8.24
**1643579**	WDR47	2.72E-04	3.26	23.67	7.26
**1644613**	FCGBP	8.95E-04	205.09	1323.89	6.46
**1384769**	GPR110	4.30E-03	0.66	3.76	5.70
**1392709**	LOC389517	2.75E-03	1.03	5.41	5.25
**1391145**	TNFRSF10D	1.18E-03	1.72	8.8	5.12
**1380783**	SERPINA5	1.91E-04	5.22	25.84	4.95
**1381005**	IGLL1	3.34E-04	12.53	60.63	4.84
**1644333**	HIGD2BP	7.41E-03	0.68	3.28	4.82
**1377824**	NPC1L1	1.38E-05	4.29	19.9	4.64
**1390306**	ANGPT2	8.13E-05	60.66	269.67	4.45
**1377223**	LOC402573	2.00E-07	9.56	41.53	4.34
**1396839**	ZNF257	1.69E-03	1.18	5.02	4.25
**1382346**	DEFA1	8.69E-03	9.49	39.77	4.19
**1397370**	SCIN	7.53E-04	35.35	146.53	4.15
**1644651**	FAM29A	6.76E-03	0.92	3.69	4.01
**1389999**	PSPN	1.52E-03	2.54	10.01	3.94
**1390131**	S100A3	5.50E-03	4.52	17.74	3.92
**1644879**	DEFB114	8.57E-03	1.05	4.03	3.84
**1381800**	GFAP	1.30E-06	1626.88	6221.58	3.82
**1396234**	LOC283755	7.51E-05	36.05	137.63	3.82
**1391959**	BCMO1	1.12E-03	12.4	46.09	3.72
**1644612**	FCGBP	5.21E-03	15.83	57.68	3.64
**1384093**	CCDC80	4.58E-04	2.04	7.28	3.57
**1396240**	SLC5A3	1.00E-07	28.47	101.49	3.56
**1393547**	ADAMTS9	1.02E-04	61.73	215.15	3.49
**1377828**	C10orf10	8.88E-04	196.57	681.47	3.47
**1392217**	STC1	2.97E-03	19.57	67.57	3.45
**1388675**	MB	2.07E-03	1.27	4.37	3.44
**1395897**	NAPSA	1.37E-04	7.47	25.45	3.41
**1378460**	CD44	1.80E-03	121.91	414.82	3.40
**1386288**	AQP1	4.57E-04	19.6	66.62	3.40
**1397153**	CHI3L2	8.43E-03	17.98	59.61	3.32
**1388753**	CHST6	1.40E-06	45.14	149.15	3.30
**1397001**	BTBD14A	< 1e-07	63.23	207.01	3.27

**1392135**	HDAC7A	4.62E-03	8.63	28.01	3.25
**1383402**	SERPINH1	6.00E-07	52.93	168.75	3.19
**1393771**	GBP2	7.01E-05	121.61	387.06	3.18
**1376875**	WISP3	4.32E-03	1.75	5.53	3.16
**1387719**	KCNE4	1.89E-04	4.28	13.5	3.15
**1378719**	STON1	5.95E-04	6.51	20.32	3.12
**1644286**	HSFY1	4.63E-04	1.25	3.9	3.12
**1381799**	TIMP1	2.85E-04	581.02	1791.67	3.08
**1389372**	DNAH11	9.70E-03	1.65	4.97	3.01
**1381556**	RBMS2	5.00E-07	19.34	57.99	3.00
**1389564**	TBX22	7.88E-03	2.61	7.53	2.89
**1645437**	UBE2C	7.28E-04	6.32	18.15	2.87
**1379339**	TCAP	2.41E-04	9.43	26.73	2.83
**1391606**	GJA4	1.40E-06	37.33	104.83	2.81
**1391403**	C1orf87	1.23E-03	7.14	19.91	2.79
**1390389**	MYBPH	2.10E-04	16.36	45.36	2.77
**1391779**	ATM	7.98E-04	1.86	5.15	2.77
**1389551**	LOC440093	3.03E-03	2.42	6.66	2.75
**1385707**	HMOX1	4.38E-04	24.96	67.64	2.71
**1385198**	WFIKKN1	7.00E-07	9.74	26.18	2.69
**1385494**	A4GALT	6.28E-05	24.02	64.48	2.68
**1383911**	TEX14	8.63E-03	3.22	8.59	2.67
**1386137**	COL4A1	8.20E-06	137.59	366.63	2.66
**1392414**	SYTL4	4.53E-04	101	268.99	2.66
**1384389**	CEL	3.00E-07	26.6	70.75	2.66
**1384903**	EVI1	2.12E-05	103.39	274.25	2.65
**1386932**	CT45-4	9.42E-03	10.81	28.61	2.65
**1378754**	OR5AK2	7.99E-03	2.63	6.96	2.65
**1396183**	RARRES3	1.09E-03	301.33	795.79	2.64
**1395534**	SLFNL1	4.03E-04	4.5	11.83	2.63
**1377281**	BAG3	2.71E-04	411.2	1080.36	2.63
**1393448**	ITGA1	2.52E-04	13.42	35.23	2.63
**1392684**	EPB41	7.95E-03	3.8	9.95	2.62
**1388614**	TRIM47	1.29E-05	76.77	200.92	2.62
**1376923**	PLA1A	2.00E-04	40.39	105.54	2.61
**1378256**	HIST1H4L	8.38E-03	2.27	5.9	2.60
**1379424**	HSPA1A	1.22E-03	449.92	1167.57	2.60
**1393800**	TUBAL3	6.92E-03	6.33	16.37	2.59
**1377288**	FGF17	3.00E-07	54.94	140.99	2.57
**1383959**	PGF	1.97E-04	6.13	15.73	2.57
**1394788**	ABCA7	3.00E-07	24.92	63.76	2.56
**1378152**	QRICH2	7.90E-06	10.3	26.31	2.55
**1377170**	GDPD3	2.61E-05	19.93	50.56	2.54
**1393639**	UCP2	6.83E-04	24.77	62.29	2.51
**1381357**	UBQLNL	1.34E-03	8.15	20.38	2.50
**1377255**	SPP1	9.56E-04	1220.74	3052.58	2.50
**1382787**	SERINC5	4.94E-05	9.95	24.86	2.50
**1379335**	FCN3	1.77E-03	22.89	57.17	2.50

**1396050**	AKR1C3	5.32E-05	261.1	650.86	2.49
**1378270**	POU4F1	1.25E-03	4.66	11.61	2.49
**1395686**	ITPKB	3.09E-05	317.43	789.04	2.49
**1395156**	STAB1	7.46E-04	32.55	80.88	2.48
**1384973**	P8	4.19E-04	65.06	160.53	2.47
**1384968**	HGFAC	5.30E-06	14.7	36.24	2.47
**1393417**	TMBIM1	1.69E-03	131.19	323.26	2.46
**1393040**	IER3	8.37E-05	76.62	188.52	2.46
**1395817**	CFI	2.35E-03	10.81	26.57	2.46
**1380284**	TMEM149	5.43E-04	55.03	133.87	2.43
**1396746**	GPR4	1.60E-03	45.63	110.8	2.43
**1383793**	HABP2	7.47E-05	3.95	9.59	2.43
**1390396**	SYPL2	1.14E-03	4.54	10.98	2.42
**1389116**	ANGPTL4	1.60E-03	86.07	207.47	2.41
**1385519**	UNC13D	1.11E-03	8.83	21.27	2.41
**1644348**	HERC2P2	1.87E-04	37.09	89.26	2.41
**1382940**	HSPB1	8.00E-07	1194.5	2872.24	2.40
**1397108**	HEY2	1.90E-06	70.3	168.95	2.40
**1396710**	MPZL1	1.43E-03	5.87	14.1	2.40
**1391000**	TFPI	4.79E-05	41.19	98.92	2.40
**1645789**	SOCS3	2.24E-03	4.89	11.73	2.40
**1382879**	NRM	2.96E-04	9.76	23.29	2.39
**1378166**	FGF11	2.99E-05	37.89	90.31	2.38
**1383211**	STEAP3	4.70E-06	13.75	32.74	2.38
**1378190**	ADORA2A	6.27E-05	55.12	131.14	2.38
**1386240**	CABP4	1.36E-04	6.21	14.65	2.36
**1389467**	MUC1	9.92E-05	33.97	80.08	2.36
**1382306**	ORAI1	1.69E-04	16.52	38.92	2.36
**1388459**	DUSP8	6.03E-05	437.78	1030.65	2.35
**1389074**	IFITM2	2.46E-03	2291.03	5388.41	2.35
**1381157**	IRF7	2.18E-04	61.69	145.02	2.35
**1386895**	TMPRSS3	4.31E-03	8.86	20.79	2.35
**1646390**	RN7SK	< 1e-07	548.81	1287.46	2.35
**1645111**	CD44	5.33E-03	18.42	43.16	2.34
**1383421**	FGFRL1	6.17E-05	109.45	256.31	2.34
**1382454**	LMOD1	7.62E-04	17.46	40.66	2.33
**1385341**	NRTN	5.10E-03	2.91	6.76	2.32
**1388090**	PARVG	3.01E-04	45.91	106.55	2.32
**1645196**	C9orf68	2.70E-04	9.32	21.63	2.32
**1646041**	BACE2	2.67E-03	11.33	26.28	2.32
**1377486**	CSDA	1.61E-03	453.91	1050.57	2.31
**1392315**	HSD3B7	5.90E-06	20.74	47.97	2.31
**1392857**	CSAG1	8.00E-07	32.85	75.95	2.31
**1383077**	P2RY8	7.46E-04	4.27	9.87	2.31
**1393511**	TNFRSF6B	6.51E-03	118.31	273.39	2.31
**1378792**	OXTR	7.51E-03	43.44	100.15	2.31
**1379925**	TMEM137	1.99E-05	59.55	137.26	2.30
**1379079**	AEBP1	4.75E-05	98.17	226.14	2.30

**1395241**	CCDC102A	4.00E-07	39.12	89.86	2.30
**1644234**	IL18BP	2.12E-03	53.67	122.89	2.29
**1645328**	C10orf54	1.64E-04	123	281.63	2.29
**1393216**	CARD14	5.16E-03	2.02	4.61	2.28
**1383273**	DTNA	3.68E-03	613.1	1399.03	2.28
**1381818**	SLCO4A1	3.84E-04	106.07	242.02	2.28
**1385531**	MLKL	7.16E-05	30.26	68.94	2.28
**1389766**	ATOH8	3.44E-04	35.99	81.99	2.28
**1380920**	TEAD2	< 1e-07	122.02	277.64	2.28
**1379492**	SP140	4.77E-03	3.02	6.85	2.27
**1391029**	RGS3	9.39E-03	3.43	7.77	2.27
**1390640**	TLR5	1.46E-04	36	81.17	2.25
**1383897**	NQO1	2.35E-03	238.2	536.3	2.25
**1395050**	C1orf64	1.74E-04	256.88	576.31	2.24
**1395167**	TCIRG1	1.64E-05	21.89	49.02	2.24
**1379843**	C19orf34	9.40E-03	2.13	4.75	2.23
**1379136**	IFITM3	3.33E-03	1843.1	4105.1	2.23
**1393946**	ATHL1	4.19E-04	23.26	51.72	2.22
**1380910**	IKBKE	1.63E-05	15.89	35.24	2.22
**1646238**	AGBL5	2.00E-06	24.35	53.99	2.22
**1378600**	FAM46C	1.56E-04	20.49	45.24	2.21
**1378330**	IL4I1	1.99E-03	4.93	10.88	2.21
**1383748**	PGAM2	2.11E-03	23.51	51.84	2.21
**1395225**	ELF5	6.95E-04	3.52	7.74	2.20
**1380400**	SFMBT2	5.19E-04	34.74	76.25	2.19
**1389616**	C1R	6.02E-03	35.55	77.9	2.19
**1382594**	PLXDC2	1.26E-04	43.29	94.86	2.19
**1380227**	TLR9	5.47E-03	6.92	15.16	2.19
**1386777**	STXBP2	4.68E-03	20.54	44.95	2.19
**1389852**	C12orf28	5.80E-04	11.07	24.16	2.18
**1388481**	BST2	1.39E-04	128.29	279.81	2.18
**1379868**	CDKN1A	3.69E-03	179.28	390.24	2.18
**1390794**	ENG	1.41E-03	110.85	241.07	2.17
**1380447**	PLEKHA4	4.22E-05	20.55	44.62	2.17
**1386335**	MST1R	1.28E-03	3.89	8.42	2.16
**1393261**	TAGLN2	9.15E-04	226.72	490.67	2.16
**1390575**	PTGER1	3.06E-03	20.06	43.28	2.16
**1645748**	SPP1	4.58E-03	944.06	2033.97	2.15
**1390324**	GBGT1	3.45E-04	22.56	48.59	2.15
**1392324**	ITGAL	1.92E-04	12.76	27.46	2.15
**1380093**	TM4SF18	1.02E-03	100.95	216.82	2.15
**1379797**	FLJ14107	1.71E-03	9.58	20.56	2.15
**1644951**	CT45-5	4.41E-03	5.57	11.95	2.15
**1390508**	POLE	2.87E-04	34.69	74.23	2.14
**1384566**	GALNTL2	2.20E-03	57.39	122.75	2.14
**1646857**	OR2M1P	4.59E-03	2.75	5.88	2.14
**1381452**	MOV10	2.53E-05	47.26	101.02	2.14
**1385157**	LGI4	3.55E-05	55.34	118.21	2.14

**1379188**	TNFRSF1A	1.84E-05	180.64	385.63	2.13
**1645560**	TNFRSF10B	4.44E-03	3.84	8.19	2.13
**1379615**	LILRA3	7.86E-04	5.96	12.7	2.13
**1392226**	RUNX3	4.10E-06	21.36	45.48	2.13
**1646813**	OSMR	2.69E-03	11.89	25.28	2.13
**1381950**	PKN3	3.78E-03	14.24	30.26	2.13
**1390799**	TFF1	6.65E-03	3.64	7.72	2.12
**1386061**	IFITM1	4.48E-03	638.84	1352.26	2.12
**1385727**	SLC19A3	1.59E-03	39.79	84.01	2.11
**1383729**	TNS1	1.83E-04	24.31	51.32	2.11
**1379896**	SOLH	6.10E-06	20.1	42.4	2.11
**1378478**	SRGAP1	3.27E-04	55.51	117.07	2.11
**1386388**	KIF1C	6.28E-04	26.9	56.71	2.11
**1644588**	FGFR4	1.99E-05	13.81	29.09	2.11
**1644470**	GEM	2.42E-04	27.19	57.1	2.10
**1397167**	CCL19	1.30E-03	29.43	61.78	2.10
**1379359**	FAM129A	3.05E-03	58.63	122.86	2.10
**1391016**	STH	5.90E-06	53.05	111.02	2.09
**1389376**	PGR	8.74E-03	4.22	8.81	2.09
**1378550**	TGM2	6.07E-03	143.91	300.35	2.09
**1386370**	SBNO2	1.46E-05	12.46	25.99	2.09
**1388204**	POLH	5.37E-03	3.86	8.05	2.09
**1385651**	LY96	3.92E-03	148.56	309.69	2.08
**1385941**	DNASE1L2	4.60E-06	24.87	51.82	2.08
**1394726**	LAT2	2.71E-04	36.73	76.53	2.08
**1393568**	TFPI	6.23E-05	18.51	38.45	2.08
**1390690**	S1PR3	1.38E-05	47.49	98.58	2.08
**1391905**	SMAD6	2.49E-03	158.15	328.06	2.07
**1381564**	DMWD	2.36E-04	93.15	192.94	2.07
**1386218**	GDAP2	7.25E-04	8.57	17.73	2.07
**1380277**	TNFRSF10B	4.39E-05	37.71	78	2.07
**1380768**	ZMIZ2	5.40E-06	82.14	169.5	2.06
**1379395**	COL18A1	7.00E-07	76.52	157.64	2.06
**1382602**	SLC25A41	1.98E-04	10.08	20.76	2.06
**1388404**	C1orf106	6.39E-04	16.82	34.54	2.05
**1385816**	TMEM176A	4.12E-03	40.02	82.11	2.05
**1392254**	BAIAP2L1	4.93E-03	7.93	16.27	2.05
**1385812**	ZNF44	2.68E-03	3.93	8.05	2.05
**1384843**	DPP9	1.66E-05	146.68	300.44	2.05
**1395842**	CFB	2.14E-03	86.08	176.02	2.04
**1384823**	SLAMF8	3.13E-03	8.31	16.98	2.04
**1381220**	TDRD10	2.60E-03	11.43	23.35	2.04
**1381301**	PYGO2	1.30E-06	39.92	81.52	2.04
**1386081**	RAPGEF3	4.05E-04	72.83	148.67	2.04
**1388827**	ANLN	9.31E-04	92.24	188.28	2.04
**1393433**	EFNA1	2.55E-04	130.09	265.38	2.04
**1396159**	FOXF1	4.05E-04	18.42	37.56	2.04
**1392422**	ADH1B	2.16E-03	6.76	13.78	2.04

**1379023**	SLC4A2	1.18E-05	22.59	46.02	2.04
**1384628**	REP15	1.43E-04	22.62	46.07	2.04
**1395553**	FZD2	7.30E-06	25	50.9	2.04
**1377925**	MS4A14	5.10E-03	8.82	17.95	2.04
**1396546**	TTC38	1.36E-04	38.94	79.2	2.03
**1391802**	DNAH17	1.42E-03	19.26	39.16	2.03
**1644262**	HYAL1	4.30E-04	17.64	35.82	2.03
**1385912**	MICB	2.01E-04	20.64	41.85	2.03
**1377701**	S100A4	5.52E-03	87.27	176.85	2.03
**1396385**	HYAL1	5.82E-04	62.83	127.15	2.02
**1643994**	LOC388955	8.83E-03	3.42	6.91	2.02
**1396576**	ADAMTS1	3.10E-03	77.86	157.16	2.02
**1387088**	ATPAF2	4.75E-05	35.69	72.03	2.02
**1643745**	MKNK2	3.58E-05	386.75	779.36	2.02
**1379252**	TM4SF1	5.67E-04	777.18	1562.52	2.01
**1386453**	TRIP10	3.79E-04	16.85	33.84	2.01
**1395207**	SUSD2	4.60E-04	41.63	83.59	2.01
**1378781**	SHC1	4.00E-07	266.29	534.67	2.01
**1377253**	TAOK2	9.91E-05	106.9	214.51	2.01
**1379247**	ID3	7.46E-04	399.85	802.14	2.01
**1391669**	SHROOM1	3.28E-04	22.38	44.84	2.00
**1378559**	DNAJB6	1.20E-04	248.27	496.93	2.00
**1381128**	VWA1	1.45E-04	48.42	96.88	2.00
**1395210**	KIAA1754	3.40E-06	57.98	115.85	2.00
**1387433**	ZNF692	9.00E-07	174.25	348.02	2.00
**1380155**	SCARA3	1.21E-03	54.6	108.93	2.00
**1379246**	CRYAB	2.73E-04	2437.64	4861.9	1.99
**1386581**	SH2B2	8.87E-05	15.54	30.98	1.99
**1396994**	HEYL	1.29E-03	81.11	161.34	1.99
**1395936**	FOXF2	2.26E-04	36.14	71.85	1.99
**1385131**	PLEKHF1	5.90E-06	91.52	181.95	1.99
**1378087**	C1orf125	2.63E-03	5.59	11.1	1.99
**1393219**	RHBDF2	2.00E-03	117.82	233.89	1.99
**1644050**	LLGL2	7.21E-05	8.43	16.73	1.98
**1379097**	SERTAD1	8.30E-06	185.59	368.25	1.98
**1394107**	KLKB1	1.02E-03	32.42	64.23	1.98
**1379715**	BGN	1.23E-05	1761.62	3486.07	1.98
**1644115**	KLHL17	5.88E-04	38.2	75.57	1.98
**1380422**	WASF2	1.98E-04	76.83	151.94	1.98
**1645764**	SPATC1	9.98E-03	2.66	5.26	1.98
**1646053**	ATXN2L	7.00E-07	34.17	67.52	1.98
**1382331**	CLIC1	3.35E-03	182	359.57	1.98
**1393062**	ELK1	1.30E-05	584.29	1152.96	1.97
**1386636**	ISG20	1.30E-03	71.88	141.83	1.97
**1395867**	C9orf61	1.01E-03	119.39	235.27	1.97
**1394637**	CD34	4.36E-04	166.29	327.56	1.97
**1389878**	ZCCHC24	3.15E-04	430.67	848.17	1.97
**1392565**	LOC400464	5.45E-05	23.82	46.91	1.97

**1383646**	SPI1	9.91E-04	31.07	61.12	1.97
**1384720**	VAT1	1.37E-05	96.77	190.34	1.97
**1379654**	LIMK2	5.26E-03	13.02	25.6	1.97
**1645485**	TSPO	4.22E-03	37.92	74.55	1.97
**1377364**	IGFBP4	1.96E-04	117.46	230.78	1.96
**1378357**	HIGD1B	6.11E-03	187.87	368.99	1.96
**1387224**	DOCK6	9.87E-05	42.66	83.76	1.96
**1388708**	GRRP1	1.60E-03	90.77	178.18	1.96
**1389735**	SPATA13	1.16E-03	30.38	59.58	1.96
**1383884**	DISC1	1.40E-04	12.42	24.35	1.96
**1397097**	C19orf51	9.17E-03	4.27	8.37	1.96
**1386865**	EBI3	9.11E-03	32.91	64.48	1.96
**1377905**	FOXD4L2	1.62E-03	6.01	11.77	1.96
**1646448**	RBM38	3.36E-05	48.15	94.18	1.96
**1391059**	TACC3	1.76E-04	8.88	17.36	1.95
**1378380**	FAM124B	4.13E-03	25.42	49.68	1.95
**1380048**	ACVRL1	7.10E-03	33.06	64.59	1.95
**1382345**	HIPK2	1.08E-03	482.67	942.79	1.95
**1379805**	MRVI1	3.67E-03	41.02	80.12	1.95
**1387842**	C12orf34	6.86E-04	49.87	97.19	1.95
**1390431**	BNIPL	4.94E-05	22.76	44.34	1.95
**1395760**	AKAP8L	4.80E-06	157.86	307.45	1.95
**1389444**	GATA2	2.16E-03	14.67	28.57	1.95
**1391364**	MAP3K8	3.98E-03	61.76	120.18	1.95
**1384175**	RESP18	5.08E-03	28.77	55.98	1.95
**1386926**	WSCD1	9.91E-05	50.18	97.44	1.94
**1390611**	TMEM140	1.78E-03	83.21	161.5	1.94
**1384427**	VAMP8	5.20E-03	211.51	410.47	1.94
**1386271**	TBL1X	9.43E-05	286.13	555.25	1.94
**1394993**	ZC3HAV1	4.95E-05	153.01	296.63	1.94
**1384998**	RPS6KA1	3.17E-05	55.14	106.89	1.94
**1376867**	SLC44A3	2.50E-03	55.61	107.78	1.94
**1385071**	KIF7	2.38E-04	24.51	47.49	1.94
**1394218**	RFXDC2	4.70E-05	58.99	114.23	1.94
**1644966**	CSAG3B	4.71E-03	4.56	8.83	1.94
**1390786**	CALCRL	4.27E-04	25.9	50.12	1.94
**1381580**	LAMB2	6.25E-04	77.46	149.88	1.93
**1390255**	HS1BP3	3.80E-06	47.04	90.96	1.93
**1388638**	SIX5	1.18E-03	62.36	120.56	1.93
**1379142**	C9orf167	3.01E-04	9.68	18.7	1.93
**1390366**	FAM64A	5.21E-03	4.68	9.04	1.93
**1378636**	SEPN1	1.10E-06	280.06	540.95	1.93
**1379657**	HSD11B2	4.21E-03	13.58	26.23	1.93
**1390024**	PPIC	3.09E-03	38.45	74.19	1.93
**1379298**	ZBTB40	1.36E-05	107.17	206.74	1.93
**1379306**	IL17RB	1.06E-03	242.55	467.45	1.93
**1389241**	VAMP5	3.71E-05	363.06	699.54	1.93
**1394151**	C10orf85	1.16E-05	53.21	102.5	1.93

**1385817**	BAZ1A	3.24E-04	34.2	65.83	1.92
**1397318**	COL4A2	9.25E-05	34.44	66.27	1.92
**1388811**	INPPL1	3.50E-06	342.95	659.9	1.92
**1381957**	LEF1	3.83E-04	80.73	155.31	1.92
**1395504**	BOC	3.54E-03	8.62	16.58	1.92
**1377387**	PRRG2	8.16E-04	13.5	25.95	1.92
**1644195**	ITGAX	9.00E-03	30.56	58.74	1.92
**1381850**	SNX33	5.54E-05	26.95	51.77	1.92
**1378040**	DFNB31	3.73E-05	35.19	67.57	1.92
**1646197**	ALPK1	3.06E-03	21.37	41.03	1.92
**1377496**	PLOD3	2.20E-06	222.42	427.03	1.92
**1380982**	COLEC12	1.02E-03	191.5	367.33	1.92
**1389275**	DDIT4L	2.91E-03	148.4	284.6	1.92
**1381204**	ABHD4	1.52E-04	30.29	58.06	1.92
**1644420**	GPER	4.82E-03	234.18	448.81	1.92
**1380159**	AHDC1	5.86E-04	78.67	150.74	1.92
**1645255**	C1QTNF1	1.19E-03	99.68	190.9	1.92
**1389505**	TNFRSF1B	7.98E-03	26.12	49.91	1.91
**1379886**	PALLD	2.56E-05	336.36	642.7	1.91
**1391647**	NEK6	1.10E-06	84.51	161.34	1.91
**1384678**	RAB13	4.47E-03	32.52	62.05	1.91
**1378265**	SPR	1.45E-04	69.34	132.11	1.91
**1646921**	NTRK1	2.88E-03	3.03	5.77	1.90
**1378678**	TRIM5	5.52E-05	23.6	44.89	1.90
**1394085**	PLCD1	8.35E-04	89.46	170.15	1.90
**1390479**	FAM124A	4.89E-04	13.12	24.88	1.90
**1382721**	FAM39DP	2.46E-04	182.56	346.08	1.90
**1395457**	RECQL4	3.99E-05	14.76	27.98	1.90
**1645511**	TRIM5	2.06E-04	13.64	25.84	1.89
**1394974**	PMP2	5.13E-04	2531.77	4796.17	1.89
**1646768**	PAPSS2	1.03E-04	109.22	206.67	1.89
**1383209**	GBP1	8.32E-03	23.85	45.07	1.89
**1388842**	SLC9A9	7.35E-05	78.21	147.68	1.89
**1388524**	BCCIP	4.30E-03	13.23	24.98	1.89
**1380127**	TCF3	4.90E-06	44.13	83.3	1.89
**1390294**	TBX2	1.15E-03	36.18	68.22	1.89
**1377874**	FLJ20920	2.28E-03	15.86	29.89	1.88
**1382933**	ZNF609	1.25E-05	88.78	167.21	1.88
**1382449**	DGCR14	1.96E-04	44.35	83.52	1.88
**1390964**	TBC1D26	2.50E-03	17.29	32.55	1.88
**1392303**	SCYE1	5.86E-03	3.65	6.87	1.88
**1394617**	CCDC88B	5.30E-04	14.2	26.72	1.88
**1390770**	EMILIN1	5.68E-03	12.37	23.27	1.88
**1378862**	BEST3	1.04E-03	8.4	15.79	1.88
**1381028**	MYO1F	4.70E-03	13.53	25.39	1.88
**1394492**	ICAM4	6.92E-03	3.55	6.66	1.88
**1385887**	SFTPC	1.20E-04	18.55	34.8	1.88
**1385840**	KIAA0323	3.03E-05	59.21	111.03	1.88

**1396184**	SLC2A4RG	9.00E-07	36.54	68.47	1.87
**1388819**	ITPR3	4.85E-03	184.96	346.58	1.87
**1394207**	GSDMD	1.35E-03	87.66	164.23	1.87
**1387508**	NXN	5.50E-04	81.59	152.84	1.87
**1380846**	PDIA4	9.16E-04	16.26	30.45	1.87
**1386588**	MLXIP	6.20E-03	5.89	11.03	1.87
**1378734**	CDK2AP2	6.92E-05	73.55	137.71	1.87
**1394329**	UPF1	2.15E-05	189.18	353.93	1.87
**1383492**	SLC6A12	4.72E-03	222.33	415.64	1.87
**1392438**	ZFHX3	5.80E-06	83.52	156.07	1.87
**1382665**	BAZ1A	9.13E-03	11.48	21.45	1.87
**1389047**	PLOD1	2.20E-06	561.5	1048.48	1.87
**1380834**	RAI14	2.06E-04	123.8	230.96	1.87
**1396512**	PRKCH	2.37E-03	100.87	188.14	1.87
**1646398**	RHBDF2	1.84E-03	60.4	112.65	1.87
**1385485**	MYO9B	2.66E-05	79.53	148.28	1.86
**1377236**	POLN	2.15E-05	36.51	68.05	1.86
**1392713**	CNN3	5.80E-05	1809.66	3371.3	1.86
**1387867**	TGFBR2	1.12E-03	97.29	181.15	1.86
**1392956**	FAM39DP	1.81E-04	341.7	635.93	1.86
**1388998**	GPNMB	2.20E-04	127.76	237.64	1.86
**1395389**	ANKHD1-EIF4EBP3	3.12E-04	10.5	19.53	1.86
**1385367**	GNG8	2.29E-03	15.85	29.47	1.86
**1381237**	PLEKHG2	8.52E-03	8.01	14.89	1.86
**1380244**	SAMD4B	6.23E-04	154.06	286.06	1.86
**1387430**	MYO1D	6.67E-04	36.44	67.62	1.86
**1382724**	C1S	8.45E-03	127.55	236.38	1.85
**1646547**	PRKX	2.05E-03	43.57	80.7	1.85
**1379475**	BMP1	1.20E-06	44.43	82.26	1.85
**1387415**	PLXNB1	1.99E-03	275.77	510.38	1.85
**1391916**	ATP4A	3.40E-03	18.89	34.96	1.85
**1394627**	ANG	3.58E-03	64.17	118.65	1.85
**1395835**	KCNN3	6.95E-03	115.81	214.08	1.85
**1377816**	EDNRA	1.54E-03	35.77	66.08	1.85
**1378697**	HR	4.09E-03	11.52	21.28	1.85
**1378025**	CLDN9	1.13E-04	27.75	51.26	1.85
**1379113**	SLC7A2	4.13E-03	74.14	136.73	1.84
**1645202**	C9orf58	2.92E-03	161.87	298.46	1.84
**1379666**	C10orf33	2.60E-06	45.24	83.4	1.84
**1382398**	REXO4	1.55E-05	183.1	337.47	1.84
**1380969**	VEGFC	2.75E-03	16.61	30.61	1.84
**1394352**	TGFB3	7.58E-03	103.73	191.11	1.84
**1388902**	CLDN5	3.95E-03	744.2	1370.13	1.84
**1644843**	DLEC1	3.13E-03	14.75	27.15	1.84
**1377609**	PTK7	5.24E-05	15.05	27.7	1.84
**1380961**	HIST1H3H	3.28E-03	5.97	10.98	1.84
**1385522**	ZDHHC18	1.77E-05	35.48	65.23	1.84
**1380315**	BCL6	1.42E-05	803.91	1476.54	1.84

**1397366**	DDR1	2.88E-04	181.68	333.56	1.84
**1377906**	ANO6	1.64E-05	88.04	161.58	1.84
**1645932**	SEPN1	1.67E-04	33.04	60.62	1.83
**1383552**	ERBB2	6.72E-03	50.94	93.31	1.83
**1390373**	ZC3H12A	2.73E-03	5.55	10.16	1.83
**1392665**	FSTL1	3.59E-03	88.35	161.59	1.83
**1378715**	CASP4	9.79E-03	20.45	37.38	1.83
**1396736**	INPP5D	1.70E-04	35	63.93	1.83
**1382698**	SLC1A5	2.03E-03	13.63	24.87	1.82
**1379119**	GIMAP8	5.55E-04	58.42	106.57	1.82
**1385103**	MFNG	2.28E-03	50.73	92.5	1.82
**1387683**	FAM107B	3.48E-03	276.68	504.35	1.82
**1394399**	TTC7A	4.44E-03	11.95	21.78	1.82
**1380569**	CHEK2	6.93E-03	10.71	19.51	1.82
**1386387**	GGTLC1	1.61E-03	11.49	20.93	1.82
**1379488**	ITGA5	2.40E-03	44.9	81.76	1.82
**1389019**	SYDE1	1.22E-03	25.91	47.15	1.82
**1388455**	HSD17B1	1.13E-03	15.44	28.08	1.82
**1388231**	DTX2	1.67E-05	96.91	176.21	1.82
**1390061**	RUNX1	1.90E-04	12.7	23.09	1.82
**1382389**	CLEC1A	4.74E-04	27.59	50.15	1.82
**1381384**	C20orf117	1.56E-05	93.06	169.04	1.82
**1394485**	GPER	6.38E-03	265.42	481.92	1.82
**1389604**	HAPLN3	8.08E-03	10.13	18.39	1.82
**1379748**	KIF15	1.98E-05	19.83	35.98	1.81
**1384310**	ITGA10	1.36E-03	59.18	107.36	1.81
**1644888**	DDX11	4.39E-03	11.45	20.77	1.81
**1396595**	TMEM2	1.27E-05	184.48	334.62	1.81
**1646924**	NRG2	2.86E-03	20.37	36.91	1.81
**1389548**	DNAJB1	3.09E-04	434.88	787.86	1.81
**1388451**	APOLD1	9.23E-03	340.33	616.32	1.81
**1384689**	NFKB2	1.93E-04	10.92	19.77	1.81
**1392938**	HCP5	6.09E-04	108.93	196.95	1.81
**1381486**	SEMA3F	3.46E-05	33.24	60.03	1.81
**1379312**	SLC25A34	5.37E-05	83.38	150.54	1.81
**1384355**	GIMAP4	7.53E-04	300.73	542.79	1.80
**1386911**	UNKL	4.00E-07	89.36	161.22	1.80
**1646393**	RHOD	5.63E-03	11.08	19.99	1.80
**1395928**	CCDC50	2.70E-06	177.54	319.87	1.80
**1394418**	MTHFR	1.09E-04	73.61	132.57	1.80
**1396651**	M6PRBP1	8.11E-04	284.22	511.63	1.80
**1387363**	PACS2	1.11E-04	94.38	169.83	1.80
**1393121**	PRAM1	4.54E-03	18.1	32.54	1.80
**1385847**	CASP7	9.16E-04	39.74	71.44	1.80
**1385756**	STARD8	7.55E-03	49.78	89.48	1.80
**1396935**	GALM	3.20E-04	38.16	68.56	1.80
**1390157**	TMC6	4.07E-03	63.63	114.31	1.80
**1390999**	DMPK	2.53E-04	40.08	72	1.80

**1390276**	RASGRP3	1.59E-03	131.42	235.9	1.80
**1389815**	FAM80B	6.47E-05	53.14	95.32	1.79
**1382246**	WWP2	3.24E-04	6.91	12.39	1.79
**1387414**	PYGL	2.62E-03	42.78	76.61	1.79
**1393382**	GPR146	3.80E-03	28.58	51.13	1.79
**1391747**	HAP1	1.37E-03	28.45	50.88	1.79
**1378461**	RFX1	1.72E-05	69.06	123.38	1.79
**1393507**	MTTP	6.83E-03	5.43	9.7	1.79
**1396858**	CGNL1	5.83E-04	682.97	1218.66	1.78
**1379931**	PLEK	1.79E-03	84.9	151.26	1.78
**1377491**	DUSP18	1.74E-05	91.05	162.13	1.78
**1389288**	TNFRSF10A	7.23E-04	12.28	21.86	1.78
**1395770**	NOTCH3	3.61E-04	106.78	190.07	1.78
**1394066**	LOC92017	2.34E-03	44.28	78.78	1.78
**1378389**	PECAM1	6.00E-03	126.21	224.38	1.78
**1390444**	FER1L3	6.75E-04	61.66	109.55	1.78
**1394022**	RPL14	7.00E-03	50.54	89.75	1.78
**1396643**	EPHB4	4.08E-03	21.69	38.49	1.77
**1394071**	FZD4	1.24E-05	177.01	313.75	1.77
**1391224**	SIPA1	6.49E-05	75.6	133.92	1.77
**1377929**	KIAA1529	8.12E-03	3.98	7.05	1.77
**1393061**	SERPING1	8.22E-04	62.01	109.79	1.77
**1378251**	MAPK7	1.40E-04	52.18	92.35	1.77
**1396167**	TRIM56	4.84E-04	34.16	60.45	1.77
**1383014**	MGC33556	3.14E-03	27.2	48.13	1.77
**1381526**	FAM38A	1.83E-04	172.75	305	1.77
**1387038**	MAP3K11	4.14E-04	70.24	123.98	1.77
**1392542**	TRAF2	3.40E-06	38.14	67.25	1.76
**1646624**	PODXL	6.44E-03	234.39	413.18	1.76
**1385409**	MC1R	5.18E-05	96.64	170.21	1.76
**1395468**	CENTD2	1.15E-03	19.39	34.15	1.76
**1397246**	FLJ43806	8.55E-03	19	33.46	1.76
**1385530**	GLIS3	1.61E-05	36.12	63.59	1.76
**1384359**	FLJ41649	4.06E-04	19.15	33.71	1.76
**1393154**	CYLN2	1.00E-06	360.95	634.77	1.76
**1381436**	FAM176B	7.70E-04	18.55	32.62	1.76
**1383020**	TP53INP2	5.77E-03	385.28	677.36	1.76
**1384036**	GIT1	4.93E-04	186.38	327.1	1.76
**1387956**	HERC5	4.29E-03	151.97	266.58	1.75
**1646250**	AFG3L1	7.74E-04	5.48	9.61	1.75
**1383355**	BBOX1	8.62E-03	151.64	265.9	1.75
**1395101**	RAMP3	2.86E-03	64.81	113.63	1.75
**1381378**	CUZD1	1.98E-05	10.8	18.93	1.75
**1381240**	GSTM4	3.34E-05	56.84	99.58	1.75
**1396945**	MYO1C	2.60E-03	26.58	46.55	1.75
**1393657**	PCOLCE	6.05E-04	28.29	49.54	1.75
**1394619**	SLC38A10	1.59E-03	25.21	44.13	1.75
**1381901**	TBC1D1	2.32E-03	8.9	15.57	1.75

**1389157**	SCAMP2	4.09E-04	50.42	88.2	1.75
**1380637**	HIST1H1A	5.14E-03	14.35	25.1	1.75
**1396743**	CDGAP	2.49E-03	21.33	37.28	1.75
**1389391**	RHPN2	5.90E-04	306.9	536.16	1.75
**1390587**	LYL1	1.32E-03	139.51	243.72	1.75
**1389813**	MIER2	1.09E-03	18.58	32.44	1.75
**1380776**	SQRDL	8.55E-03	23.56	41.12	1.75
**1392253**	MLLT6	1.33E-05	365.55	637.62	1.74
**1382059**	ECHDC3	6.82E-03	36.27	63.25	1.74
**1377968**	KIFC1	3.53E-03	9.83	17.14	1.74
**1388624**	C14orf139	5.91E-03	23.89	41.65	1.74
**1378666**	PRB4	6.94E-03	4.71	8.21	1.74
**1384962**	SEMA6C	1.07E-04	32.64	56.88	1.74
**1389189**	NT5DC2	3.28E-05	99.48	173.33	1.74
**1388807**	UPP2	7.63E-03	38.52	67.05	1.74
**1391167**	HIST1H2BD	2.01E-03	95.89	166.91	1.74
**1643954**	LOC613037	5.57E-03	1686.65	2935.54	1.74
**1395741**	CDK2	2.54E-03	41.24	71.74	1.74
**1391210**	TAP1	2.13E-04	342.1	595.09	1.74
**1379274**	KCNJ2	7.67E-05	156.74	272.65	1.74
**1387243**	RHBDF2	4.64E-03	15.08	26.23	1.74
**1393619**	SASH3	2.91E-03	29.44	51.2	1.74
**1395266**	SLC45A3	6.41E-03	42.85	74.52	1.74
**1646418**	RFX2	9.23E-04	24.25	42.17	1.74
**1378516**	CHST3	1.66E-03	128.24	222.91	1.74
**1384723**	MARCH6	1.31E-03	1262.24	2194.03	1.74
**1380406**	SLC26A6	1.90E-06	37.64	65.4	1.74
**1389064**	PCTK3	7.43E-05	84.42	146.63	1.74
**1381884**	UNC5B	2.11E-05	37.59	65.29	1.74
**1389506**	PTHR1	8.39E-04	287.8	499.63	1.74
**1377355**	AGTRAP	5.76E-03	18.55	32.2	1.74
**1646519**	PSMB8	1.04E-03	76.72	133.07	1.73
**1379053**	DFFA	7.40E-04	357.91	620.68	1.73
**1377982**	STK38	2.40E-06	118.79	206	1.73
**1395440**	RAD54L	9.53E-03	9.55	16.56	1.73
**1379260**	ZNF397	6.09E-04	38.86	67.37	1.73
**1381459**	EPHA2	3.89E-03	8.21	14.23	1.73
**1646863**	OR4B1	6.48E-03	8.93	15.47	1.73
**1377289**	RALGDS	2.60E-06	1256.18	2175.98	1.73
**1392086**	KIAA0913	3.00E-07	459.09	793.87	1.73
**1381970**	GLTP	4.14E-04	1031.82	1783.38	1.73
**1395556**	MORC2	9.49E-05	83.43	144.06	1.73
**1385110**	PDGFRB	1.87E-04	373.22	644.25	1.73
**1389586**	ACTL7B	9.56E-03	9.53	16.45	1.73
**1395122**	NECAP2	4.72E-05	141.65	244.46	1.73
**1646748**	PCDH18	5.41E-05	98.3	169.63	1.73
**1386760**	KDELC2	8.82E-04	58.87	101.48	1.72
**1396812**	NOD1	1.19E-03	25.12	43.3	1.72

**1388986**	DDR2	1.19E-03	60.23	103.81	1.72
**1380440**	ADAT3	2.02E-04	13.55	23.35	1.72
**1387975**	SIX4	5.81E-05	86.35	148.79	1.72
**1377374**	TMEM63A	5.20E-03	62.94	108.41	1.72
**1382967**	AHNAK	2.29E-03	421.07	724.91	1.72
**1384160**	FLCN	9.97E-03	23.62	40.66	1.72
**1384587**	P2RX7	7.00E-07	293.08	504.33	1.72
**1394328**	CD151	1.81E-03	30.47	52.39	1.72
**1645773**	SP100	1.31E-03	13.35	22.95	1.72
**1389935**	TFEB	5.06E-03	13.09	22.49	1.72
**1395464**	FERMT3	1.19E-03	26.46	45.42	1.72
**1396737**	MSN	8.50E-04	379.35	651.16	1.72
**1386607**	SLC15A3	1.87E-03	165.65	284.26	1.72
**1383945**	PROM2	2.13E-04	23.88	40.95	1.71
**1382136**	C6orf201	6.98E-03	3.54	6.07	1.71
**1645866**	SLC30A4	1.11E-03	22.63	38.79	1.71
**1388666**	HIP1	1.86E-03	67.31	115.35	1.71
**1392040**	CCNA2	2.00E-04	26.51	45.41	1.71
**1377307**	NPAL2	5.79E-03	7.46	12.77	1.71
**1377931**	SNX31	1.54E-03	12	20.53	1.71
**1388868**	FAM125B	8.31E-05	304.01	519.99	1.71
**1382335**	STAT3	3.60E-06	419.45	717.44	1.71
**1379376**	CEBPD	2.21E-03	658.1	1125.35	1.71
**1643636**	NBPF20	1.22E-03	1834.4	3136.36	1.71
**1392002**	NFKBIZ	2.32E-04	146.44	250.1	1.71
**1388008**	MAP4	2.70E-03	40.75	69.58	1.71
**1383577**	GNRH1	1.16E-05	44.3	75.64	1.71
**1396586**	NDUFA4L2	1.23E-03	148.2	252.98	1.71
**1377995**	SLC6A9	3.80E-04	86.25	147.21	1.71
**1378223**	ADA	1.67E-03	69.01	117.77	1.71
**1381761**	TPCN1	3.21E-03	16.62	28.36	1.71
**1378267**	ABCC3	2.36E-03	14.66	25.01	1.71
**1381408**	MVP	9.66E-03	125.93	214.72	1.71
**1391335**	C5orf39	6.45E-03	38.31	65.31	1.70
**1377611**	GNG11	3.41E-03	314.68	536.4	1.70
**1388549**	JAG1	1.53E-05	109.33	186.24	1.70
**1382906**	SCARA3	1.70E-03	148.24	252.51	1.70
**1392392**	C16orf5	4.44E-03	76.09	129.6	1.70
**1387167**	CGN	1.00E-07	93.29	158.85	1.70
**1380825**	IL15RA	9.25E-03	7.4	12.59	1.70
**1385315**	MAMDC4	3.49E-04	20.75	35.3	1.70
**1384137**	IFI16	6.68E-03	63.98	108.79	1.70
**1394733**	LYG1	2.03E-04	18.85	32.05	1.70
**1644673**	FAM129B	2.76E-03	22.35	37.93	1.70
**1392954**	TAZ	9.60E-06	77.83	132.01	1.70
**1380733**	IFI35	4.11E-03	88.32	149.67	1.69
**1396985**	PLSCR4	2.85E-03	216.13	366.03	1.69
**1392615**	KCNJ4	9.05E-03	4.81	8.14	1.69

**1394181**	TRIOBP	4.29E-03	121.16	204.88	1.69
**1380712**	SART1	2.75E-04	41.7	70.51	1.69
**1392661**	STK36	2.46E-05	540.88	913.88	1.69
**1387438**	CLDN15	4.37E-03	9.31	15.73	1.69
**1393546**	FOXO4	1.22E-04	273.18	461.41	1.69
**1391431**	C10orf116	2.45E-03	1310.04	2212.18	1.69
**1388294**	LOC402110	2.34E-03	6.27	10.58	1.69
**1378747**	CTAGE5	5.69E-03	20.91	35.28	1.69
**1393290**	CIC	2.77E-05	142.98	241.24	1.69
**1389498**	BOK	4.22E-03	97.21	164.01	1.69
**1382232**	KCNJ8	5.77E-05	99.42	167.72	1.69
**1394361**	HHEX	1.15E-03	24.56	41.43	1.69
**1644438**	GOLPH4	1.25E-03	228.38	385.21	1.69
**1380045**	GRAP	3.61E-03	36.96	62.34	1.69
**1379993**	C1orf144	1.77E-04	245.75	414.43	1.69
**1393041**	TNFRSF25	9.03E-04	349.75	589.29	1.68
**1378608**	ZNF264	2.46E-04	94.44	159.07	1.68
**1395287**	BARD1	4.33E-05	55.67	93.72	1.68
**1644044**	LINCR	7.12E-05	18.63	31.35	1.68
**1645195**	CACNA1I	4.40E-06	737.31	1240.66	1.68
**1377717**	FTCD	2.63E-03	74.63	125.5	1.68
**1646449**	RBM39	8.05E-05	427.51	718.9	1.68
**1377537**	RRBP1	1.22E-03	39.28	66.05	1.68
**1377972**	NFKBIB	8.77E-04	17.77	29.87	1.68
**1379209**	C14orf151	3.15E-03	20	33.61	1.68
**1389051**	SLC9A5	1.49E-05	77.26	129.81	1.68
**1386905**	MPZL2	5.41E-03	6.69	11.24	1.68
**1395462**	LAMA5	5.33E-05	673.07	1130.76	1.68
**1376927**	PARP10	6.79E-04	26.7	44.84	1.68
**1376873**	FXYD5	2.49E-03	120.49	202.24	1.68
**1394202**	GLI2	1.56E-03	13.79	23.14	1.68
**1379728**	NIPBL	9.90E-06	210.22	352.71	1.68
**1644455**	GLIS3	6.66E-04	15.41	25.84	1.68
**1393126**	LDLRAP1	5.10E-03	22.8	38.22	1.68
**1385846**	ABHD14B	5.52E-03	21.8	36.52	1.68
**1646026**	BCL2L12	1.36E-03	26	43.52	1.67
**1390721**	BCORL1	8.90E-05	25.33	42.37	1.67
**1384006**	ZDHHC12	2.48E-03	18.15	30.35	1.67
**1393246**	MTMR3	1.22E-03	36.87	61.6	1.67
**1379227**	ATXN7L2	4.81E-05	82.38	137.58	1.67
**1389682**	MAF1	1.14E-04	92.73	154.85	1.67
**1644189**	ITGB5	1.24E-04	492.07	821.61	1.67
**1643662**	MYH9	1.69E-05	998.61	1665.79	1.67
**1384836**	DIP2A	1.27E-04	17.98	29.99	1.67
**1388571**	GAB3	7.00E-03	9.42	15.71	1.67
**1379375**	ANKRD13B	9.18E-04	26.7	44.51	1.67
**1382934**	PPP4R1L	5.96E-03	7.1	11.83	1.67
**1644423**	GPNMB	1.33E-03	68.38	113.9	1.67

**1395503**	ATXN3	1.58E-04	127.34	211.99	1.66
**1378936**	SESN2	8.38E-05	23.71	39.46	1.66
**1384447**	LDLRAD3	9.54E-03	21.97	36.56	1.66
**1385837**	C20orf94	5.46E-03	57.79	96.16	1.66
**1382778**	SERPINB6	1.33E-04	1238.1	2058.52	1.66
**1381529**	SH3BP5L	4.60E-04	226.19	376.07	1.66
**1397387**	P2RY5	8.35E-03	96.22	159.9	1.66
**1388751**	OGDH	1.30E-04	240.83	400.1	1.66
**1394077**	FLJ37078	7.68E-04	169.87	282.16	1.66
**1391719**	RBM14	3.25E-05	747.24	1240.69	1.66
**1379115**	TEAD4	5.53E-03	43.2	71.72	1.66
**1392172**	FAM48A	9.20E-06	68.32	113.42	1.66
**1644679**	FAM110A	3.30E-06	15.61	25.89	1.66
**1393818**	SNX6	5.20E-04	143.79	238.48	1.66
**1376858**	FOXQ1	1.70E-03	202.51	335.83	1.66
**1378148**	SYNJ2BP	1.72E-04	613.89	1017.71	1.66
**1385392**	FAM111A	2.00E-03	55.74	92.39	1.66
**1646510**	PTGS2	1.23E-04	241.1	399.39	1.66
**1392496**	CPS1	2.16E-03	47.07	77.93	1.66
**1378676**	MOBKL2C	9.86E-05	86.97	143.98	1.66
**1381236**	CDH23	3.11E-04	11.77	19.48	1.66
**1389800**	IL3RA	4.75E-03	14.28	23.61	1.65
**1382166**	RPN2	7.49E-03	35.93	59.4	1.65
**1383148**	SHF	4.21E-04	21.79	36.02	1.65
**1376933**	DNM2	1.11E-03	70.25	116.11	1.65
**1395401**	TSPO	1.99E-03	443.4	732.61	1.65
**1644351**	HDAC10	6.97E-05	14.96	24.68	1.65
**1389187**	TOB2	2.54E-03	49.31	81.33	1.65
**1381807**	JMJD6	5.53E-04	20.09	33.13	1.65
**1380513**	RGL2	1.66E-03	61.55	101.48	1.65
**1397032**	LOC63920	5.64E-04	73.78	121.63	1.65
**1646036**	BANP	3.01E-03	29.23	48.18	1.65
**1383282**	PARP14	5.11E-03	54.18	89.28	1.65
**1383536**	DNHD1	1.90E-04	26.7	43.96	1.65
**1646560**	PRDM16	4.88E-03	70.69	116.37	1.65
**1387236**	UBXN2A	4.80E-06	153.85	253.25	1.65
**1643814**	MARCH3	4.98E-03	58.17	95.75	1.65
**1378273**	PLAC9	7.42E-03	70.59	116.19	1.65
**1393191**	PLOD2	1.89E-04	205.51	338.19	1.65
**1644820**	DNAJB2	1.80E-06	1296.45	2131.66	1.64
**1384168**	PLCG2	9.41E-03	84.38	138.72	1.64
**1645096**	CDC2L2	6.84E-03	49.76	81.77	1.64
**1390830**	FAM107A	3.09E-03	9206.49	15124.78	1.64
**1390831**	TLN1	1.00E-04	110.9	182.18	1.64
**1384374**	FAM109A	3.24E-05	28.88	47.44	1.64
**1395806**	FBLN1	4.71E-03	304.22	499.28	1.64
**1644267**	HYDIN	1.74E-04	10.81	17.74	1.64
**1381947**	COG7	2.83E-05	53.05	87.05	1.64

**1396143**	PKP4	3.00E-03	676.18	1109.11	1.64
**1382167**	EDC3	9.47E-05	81.24	133.23	1.64
**1389093**	PTBP1	5.91E-05	798.32	1308.95	1.64
**1387267**	L3MBTL	1.35E-04	16.5	27.05	1.64
**1384728**	C1orf198	3.44E-03	274.67	450.15	1.64
**1381893**	CDK6	5.42E-05	100.1	164.05	1.64
**1381453**	PHCA	2.60E-06	379.01	620.86	1.64
**1394599**	HMG20B	3.06E-05	256.22	419.66	1.64
**1379559**	ProSAPiP1	7.13E-04	726.5	1189.59	1.64
**1385224**	PLEKHO2	4.88E-03	45.01	73.67	1.64
**1395663**	LMNA	1.46E-03	93.39	152.85	1.64
**1387400**	CD40	5.01E-03	14.42	23.6	1.64
**1382095**	PRB2	2.03E-03	17.14	28.05	1.64
**1644372**	HAP1	9.13E-03	9.05	14.81	1.64
**1379815**	ATP5D	5.88E-04	192.12	314.3	1.64
**1392582**	SHB	3.92E-04	44.19	72.28	1.64
**1380401**	PRR5	1.02E-04	94.27	154.19	1.64
**1388373**	FLNB	4.98E-05	225.61	368.86	1.63
**1385687**	BVES	1.73E-03	23.87	39.02	1.63
**1395736**	PDPN	4.76E-03	126	205.87	1.63
**1381207**	LRP10	4.70E-03	77.29	126.26	1.63
**1377945**	TSEN54	9.20E-05	72.48	118.37	1.63
**1380051**	TLE2	2.06E-04	160.07	261.39	1.63
**1645012**	CMTM3	5.13E-03	28.58	46.67	1.63
**1377947**	APOL3	7.30E-03	58.22	95.04	1.63
**1387795**	NY-SAR-48	6.20E-04	17.42	28.42	1.63
**1644445**	GNA12	2.56E-03	218.01	355.57	1.63
**1395513**	ITGA2	7.56E-03	24.21	39.45	1.63
**1388916**	C9orf164	2.49E-03	215.97	351.71	1.63
**1392489**	RAET1G	2.60E-03	6.86	11.17	1.63
**1385032**	MKL1	5.82E-04	118.22	192.42	1.63
**1393929**	TSHZ1	4.88E-04	377.54	614.36	1.63
**1384379**	STK3	2.50E-03	124.96	203.31	1.63
**1379915**	STK11IP	8.51E-05	102.37	166.55	1.63
**1393073**	MAPKBP1	3.96E-04	81.19	131.93	1.62
**1387202**	CTDSP2	3.90E-06	563.12	914.98	1.62
**1387942**	MYOM1	8.01E-03	186.41	302.86	1.62
**1386710**	EEF1D	3.16E-04	298.68	485.13	1.62
**1378880**	CUBN	5.11E-04	13.96	22.67	1.62
**1383450**	C20orf160	9.26E-03	84.46	137.14	1.62
**1383016**	TMCC2	1.15E-04	124.38	201.95	1.62
**1396745**	BLM	1.08E-03	25.96	42.15	1.62
**1384457**	PPP1R16B	2.38E-04	956.4	1552.26	1.62
**1380359**	AMBRA1	8.21E-03	16.23	26.34	1.62
**1392458**	TRIM21	1.41E-03	24.34	39.5	1.62
**1396153**	PSMB8	4.24E-03	18.23	29.58	1.62
**1389621**	GSDMB	7.30E-06	120.17	194.96	1.62
**1381062**	WHSC2	7.78E-03	35.55	57.63	1.62

**1377576**	IGFBP7	5.28E-03	861.42	1396.39	1.62
**1390529**	VPS37B	1.84E-04	131.63	213.32	1.62
**1395996**	B4GALNT4	8.00E-04	203.25	329.32	1.62
**1644439**	GNB4	5.86E-03	24.03	38.93	1.62
**1384269**	GIMAP5	8.26E-03	89.07	144.25	1.62
**1644152**	KIAA0562	4.96E-05	72.03	116.64	1.62
**1396637**	TMSL8	6.54E-03	13.89	22.49	1.62
**1392957**	GLT25D1	1.67E-05	266.64	431.36	1.62
**1644242**	IGLL3	4.56E-04	12.54	20.28	1.62
**1386077**	TICAM2	1.86E-05	101.3	163.8	1.62
**1391461**	SH3PXD2A	9.57E-05	912	1473.57	1.62
**1380597**	PCNX	7.00E-06	173.95	280.92	1.61
**1381872**	KHSRP	3.93E-04	343.29	554.32	1.61
**1645816**	SMTN	3.85E-03	9.24	14.92	1.61
**1646006**	RRBP1	3.05E-04	193.69	312.66	1.61
**1388746**	PHF19	9.64E-04	63.28	102.14	1.61
**1378574**	DENND2A	4.25E-04	221.07	356.76	1.61
**1383996**	TAOK1	3.88E-04	134.29	216.67	1.61
**1392225**	GPR124	4.46E-03	56.68	91.44	1.61
**1388307**	MBD3	7.77E-04	96.63	155.87	1.61
**1385159**	PARP16	5.64E-03	33.68	54.3	1.61
**1394907**	TMEM79	3.64E-03	96.99	156.33	1.61
**1389732**	MTG1	4.40E-03	26.75	43.11	1.61
**1383434**	HIST2H2BE	9.08E-03	97.41	156.96	1.61
**1644073**	LAT2	1.61E-03	21.36	34.4	1.61
**1394831**	VANGL2	1.14E-04	70.38	113.3	1.61
**1384945**	POLD1	1.07E-04	29.29	47.14	1.61
**1393743**	PPARD	1.32E-04	70.21	112.98	1.61
**1385051**	SLC6A8	4.74E-03	253.57	408.02	1.61
**1388298**	FERMT2	9.42E-04	952.57	1532.46	1.61
**1383474**	IFIT2	6.37E-04	200.07	321.84	1.61
**1395135**	FSTL3	2.22E-04	64.16	103.19	1.61
**1380208**	FAM167B	3.26E-03	10.54	16.95	1.61
**1391546**	ZNF621	1.28E-04	134.62	216.46	1.61
**1389075**	PHF2	1.28E-04	173.32	278.61	1.61
**1394645**	CACNA1A	8.43E-03	64.36	103.43	1.61
**1388398**	STARD10	1.34E-05	249.94	401.64	1.61
**1395275**	HDAC1	1.27E-04	447.14	718.48	1.61
**1388143**	OR5B12	4.79E-03	9.37	15.05	1.61
**1395970**	ABCB7	1.72E-03	77.71	124.79	1.61
**1388861**	ZER1	1.74E-04	184.24	295.86	1.61
**1390449**	FYCO1	1.26E-03	73.3	117.65	1.61
**1393263**	ADCY6	1.04E-05	205.58	329.88	1.60
**1395461**	GLIPR2	2.71E-03	315.99	506.96	1.60
**1393582**	TBC1D2B	1.01E-05	149.96	240.55	1.60
**1393020**	MAN2B1	2.22E-04	30.26	48.52	1.60
**1645116**	CD151	3.48E-05	1094.97	1755.29	1.60
**1381964**	OBSCN	1.84E-03	15.46	24.78	1.60

**1394633**	ESAM	3.73E-03	248.71	398.42	1.60
**1389210**	SLC7A9	1.80E-03	55.56	88.98	1.60
**1394989**	NKIRAS2	4.71E-04	21.35	34.19	1.60
**1379010**	INHBB	8.94E-03	35.85	57.41	1.60
**1383520**	SLC25A1	6.54E-04	117.67	188.33	1.60
**1380073**	OLFML2B	2.25E-03	29.16	46.67	1.60
**1395051**	ASB6	3.46E-04	134.55	215.19	1.60
**1385681**	SEMA4B	2.12E-04	119.41	190.94	1.60
**1382565**	NOTCH4	6.29E-05	122.42	195.75	1.60
**1384055**	ZBTB20	8.58E-04	309.77	495.15	1.60
**1377524**	P4HA2	4.44E-04	50.56	80.76	1.60
**1388845**	KLHL36	5.87E-05	65.88	105.22	1.60
**1388015**	PIM1	9.00E-04	120.18	191.92	1.60
**1379200**	RXRA	3.25E-05	726.36	1159.56	1.60
**1377807**	NEO1	3.09E-04	126.7	202.26	1.60
**1394860**	TTBK1	2.28E-04	53.52	85.42	1.60
**1388849**	RNF166	7.53E-04	20.79	33.18	1.60
**1396396**	ARHGEF10	1.77E-04	383.46	611.79	1.60
**1389518**	CALHM2	3.45E-03	27.19	43.38	1.60
**1645859**	SLC43A3	1.50E-03	19.89	31.72	1.59
**1380798**	ITGB1	4.55E-03	799.13	1272.55	1.59
**1388595**	PREX1	2.32E-03	67.01	106.69	1.59
**1392811**	SERTAD3	2.89E-03	31.17	49.62	1.59
**1388576**	FAM43A	5.80E-04	273.29	434.8	1.59
**1379089**	OAS2	6.50E-03	12.46	19.82	1.59
**1389471**	LMAN2L	2.64E-05	148.17	235.69	1.59
**1385133**	RFTN2	5.98E-03	585.66	931.41	1.59
**1395331**	SFRS16	4.31E-04	31.51	50.11	1.59
**1389449**	BAZ2B	3.59E-04	370.49	589.06	1.59
**1378720**	TEF	1.03E-04	604.01	960.11	1.59
**1384335**	CCDC134	4.87E-03	8.1	12.87	1.59
**1390268**	ARID3A	7.92E-05	100.4	159.39	1.59
**1379328**	FURIN	1.11E-04	42.51	67.46	1.59
**1645721**	ST5	1.05E-03	72.16	114.51	1.59
**1387409**	TBX3	4.17E-03	19.22	30.5	1.59
**1643834**	MAP2K3	1.16E-04	70.73	112.22	1.59
**1393599**	PARP4	7.35E-03	189.69	300.96	1.59
**1397396**	ITSN1	8.15E-05	239.5	379.96	1.59
**1394409**	SLC25A29	5.14E-04	128.64	204	1.59
**1382452**	ZDHHC8	8.90E-05	1159.24	1838.09	1.59
**1644581**	FLJ16793	8.43E-03	13.17	20.88	1.59
**1391366**	SLC1A3	3.64E-03	3935.85	6239.89	1.59
**1387894**	TTYH3	9.22E-04	212.12	336.15	1.58
**1393595**	C6orf59	1.83E-03	55.68	88.23	1.58
**1387600**	NADSYN1	1.54E-03	50.34	79.72	1.58
**1386212**	VPS13C	7.80E-04	81.68	129.35	1.58
**1389345**	MCM5	7.74E-03	37.99	60.16	1.58
**1385268**	NEDD4	8.01E-04	17.69	28.01	1.58

**1379090**	NPAS3	1.59E-03	187.36	296.59	1.58
**1390902**	SLC35D2	8.35E-03	38.38	60.74	1.58
**1379902**	PPFIBP2	3.89E-03	166.5	263.45	1.58
**1646231**	AHCTF1	1.08E-05	95.57	151.15	1.58
**1397240**	STARD3	1.77E-03	44.92	71.01	1.58
**1378914**	LEPRE1	5.65E-05	57.19	90.36	1.58
**1379514**	TTC14	3.50E-04	340.7	538.15	1.58
**1393141**	DNASE1	4.28E-03	15.33	24.2	1.58
**1378421**	LITAF	9.43E-03	360.34	568.82	1.58
**1391879**	ATG16L2	6.84E-04	85.17	134.43	1.58
**1380295**	AKNA	2.51E-04	63.06	99.53	1.58
**1384768**	EPAS1	4.17E-03	896.47	1414.84	1.58
**1391738**	CLEC14A	9.80E-03	152.46	240.59	1.58
**1382861**	PTTG1IP	1.61E-03	1291.82	2037.32	1.58
**1393492**	SLC39A1	1.95E-04	239.15	377.06	1.58
**1396002**	CELSR3	8.57E-05	579.77	912.94	1.57
**1393842**	CCND1	1.92E-03	459.36	723.15	1.57
**1392681**	BIN3	3.75E-04	32.31	50.86	1.57
**1389190**	CYP27A1	3.59E-03	180.24	283.7	1.57
**1381694**	RIC8B	4.31E-03	28.66	45.11	1.57
**1386550**	ST5	9.17E-04	54.15	85.23	1.57
**1392606**	COX19	7.22E-04	291.28	458.36	1.57
**1396291**	FBXL20	1.78E-03	140.07	220.41	1.57
**1380604**	VASH1	2.19E-03	192.09	302.25	1.57
**1383727**	SEPT6	8.39E-03	12.68	19.95	1.57
**1377732**	CDC2L5	1.08E-04	163.04	256.4	1.57
**1646713**	PDE9A	3.18E-03	166.37	261.59	1.57
**1392568**	FARP1	4.78E-04	603.77	949.18	1.57
**1386316**	PLAU	8.89E-03	12.9	20.27	1.57
**1388897**	PHKA2	1.44E-04	151.12	237.38	1.57
**1646777**	PALM	1.05E-03	247.84	389.16	1.57
**1378845**	C19orf25	1.33E-04	62.42	97.99	1.57
**1380292**	GPT2	3.17E-05	1304.84	2047.92	1.57
**1381920**	PHF16	4.09E-04	77.31	121.33	1.57
**1378669**	CHP2	1.89E-03	8.39	13.16	1.57
**1388964**	KCNQ2	6.39E-03	44.91	70.43	1.57
**1394737**	KIAA0329	4.36E-03	30.95	48.52	1.57
**1385073**	C1QTNF6	9.02E-04	15.2	23.81	1.57
**1395624**	ZBTB46	2.51E-04	104.06	162.97	1.57
**1387959**	SOX12	3.34E-04	45.4	71.08	1.57
**1395722**	KIF13B	9.27E-04	166.53	260.56	1.56
**1388515**	LEPREL2	2.61E-03	33.38	52.21	1.56
**1392897**	ASPRV1	5.09E-05	36.63	57.28	1.56
**1385406**	TOP3B	6.53E-05	146.39	228.91	1.56
**1395407**	C17orf62	2.41E-03	161.35	252.28	1.56
**1383921**	MAPK8IP1	9.08E-04	304.51	476.02	1.56
**1383699**	PARP12	5.77E-03	55.35	86.52	1.56
**1377817**	RIT1	8.59E-03	34.78	54.35	1.56

**1389173**	ARHGEF19	4.07E-03	25.35	39.61	1.56
**1385929**	ELF1	7.15E-04	126.81	198.05	1.56
**1388888**	DGKD	1.06E-04	14.57	22.75	1.56
**1387491**	ANTXR2	1.08E-03	41.91	65.43	1.56
**1389550**	MYST3	8.66E-04	359.84	561.34	1.56
**1379866**	VARS2	6.91E-04	386.79	603.17	1.56
**1643436**	NOPE	6.67E-03	109.81	171.24	1.56
**1385633**	TRABD	6.01E-05	464.97	725.06	1.56
**1382904**	CARHSP1	2.80E-03	350.66	546.78	1.56
**1646626**	POL3S	2.99E-05	52.58	81.98	1.56
**1387210**	RFX4	2.39E-03	10.13	15.79	1.56
**1377040**	EFNB1	5.82E-03	30.74	47.9	1.56
**1378727**	RSAD1	6.98E-04	69.96	109.01	1.56
**1643887**	LRCH4	6.94E-05	81.89	127.58	1.56
**1387408**	TGFBR3	6.04E-03	229.51	357.53	1.56
**1394184**	LAMC1	1.61E-04	338.95	527.86	1.56
**1385018**	JUP	3.36E-03	55.15	85.87	1.56
**1392528**	MYD88	6.93E-03	41.95	65.31	1.56
**1387458**	ACTN4	5.27E-04	331.71	516.42	1.56
**1388764**	PAPOLA	2.82E-03	381.52	593.53	1.56
**1378692**	MGAT1	8.65E-04	318.03	494.69	1.56
**1646904**	NUPR1	1.78E-03	204.47	318.02	1.56
**1386923**	IQSEC2	8.34E-04	318.08	494.39	1.55
**1644081**	LASS2	8.87E-03	108.84	169.15	1.55
**1390677**	CIB2	6.96E-03	33.72	52.4	1.55
**1387434**	RHOQ	4.85E-05	1435.23	2230.1	1.55
**1391739**	RIMS4	6.99E-04	226.51	351.91	1.55
**1382827**	LASS4	1.59E-03	41.13	63.9	1.55
**1388198**	SAPS1	1.51E-03	122.57	190.19	1.55
**1394602**	MTMR11	3.92E-05	141.34	219.31	1.55
**1389246**	C9orf114	7.87E-04	230.91	358.28	1.55
**1379582**	SIN3B	5.71E-04	206.91	320.9	1.55
**1383904**	CD247	4.66E-05	69.63	107.99	1.55
**1382522**	SDF2L1	1.49E-03	436.35	676.44	1.55
**1644936**	CUTL1	4.54E-03	185.27	287.01	1.55
**1643821**	MAPK4	1.09E-03	435.04	673.79	1.55
**1389826**	RBCK1	4.02E-05	135.48	209.83	1.55
**1385438**	GAA	2.71E-04	58.42	90.48	1.55
**1381820**	ACTL6A	9.38E-04	107.54	166.55	1.55
**1378828**	METTL7B	6.42E-03	36.27	56.17	1.55
**1380579**	CYP21A2	1.28E-03	20.73	32.1	1.55
**1391027**	BCL3	4.49E-03	50.68	78.46	1.55
**1388011**	CDC2L6	1.08E-04	827.37	1280.66	1.55
**1383072**	PHLPP	2.55E-03	146.68	226.93	1.55
**1390228**	LLGL1	1.58E-04	522.91	808.99	1.55
**1382265**	CXCL16	3.75E-03	308.62	477.14	1.55
**1386999**	PLXNA3	4.75E-04	75.95	117.37	1.55
**1384640**	PTCHD1	1.22E-03	230.67	356.38	1.54

**1382062**	PLEKHM1	1.21E-05	93.58	144.25	1.54
**1385484**	EIF2C2	9.20E-04	517.96	797.96	1.54
**1388183**	TRIM25	7.25E-05	91.25	140.57	1.54
**1377845**	STK32B	1.33E-03	47.33	72.84	1.54
**1643561**	WHDC1	2.20E-04	32.62	50.19	1.54
**1383880**	RHOC	8.81E-03	431.95	664.43	1.54
**1387490**	IGF2BP2	1.87E-03	48.69	74.88	1.54
**1644965**	CSAG3B	7.59E-03	9.26	14.24	1.54
**1388712**	TTC23	6.11E-03	50.79	78.09	1.54
**1382260**	TYK2	6.70E-06	612.71	941.48	1.54
**1385706**	NUMA1	1.16E-04	296.49	455.57	1.54
**1381751**	CBFB	4.18E-03	97.08	149.1	1.54
**1392620**	CEP164	2.31E-03	67.41	103.49	1.54
**1378841**	NPAS3	1.01E-03	11.85	18.19	1.54
**1388005**	PHF6	7.94E-03	7.61	11.68	1.53
**1387271**	ZNF444	2.43E-04	125.29	192.26	1.53
**1382505**	CLDN15	9.65E-04	104.51	160.36	1.53
**1389779**	ST3GAL4	2.06E-04	72.96	111.94	1.53
**1378229**	DTNBP1	2.39E-03	18.06	27.69	1.53
**1385928**	RAVER1	1.62E-04	69.88	107.14	1.53
**1385253**	GNA11	1.67E-03	599.69	919.32	1.53
**1381334**	CDK5RAP2	8.85E-04	178.21	273.13	1.53
**1386982**	VSIG2	3.85E-03	18.4	28.17	1.53
**1380697**	CD320	3.55E-04	192.33	294.43	1.53
**1388344**	NINJ1	6.18E-05	370.65	567.41	1.53
**1378577**	LRDD	7.16E-03	36.04	55.17	1.53
**1384619**	ANXA5	4.45E-04	644.36	986.2	1.53
**1391318**	BRPF1	4.35E-04	62.62	95.84	1.53
**1385976**	SETD1A	4.03E-04	164.19	251.16	1.53
**1376964**	FBS1	5.96E-03	31.48	48.15	1.53
**1378178**	CHD4	2.59E-04	532.39	814.21	1.53
**1396523**	ABCA2	6.53E-05	167.84	256.61	1.53
**1379845**	PRB1	3.27E-03	8.15	12.46	1.53
**1388179**	ACADVL	4.76E-05	1469.81	2247.03	1.53
**1390393**	VWA5A	9.95E-03	30.45	46.55	1.53
**1378583**	DGKG	1.01E-03	65.7	100.39	1.53
**1391156**	TJP1	3.08E-03	749.15	1144.68	1.53
**1383584**	MLL4	8.77E-05	100.9	154.09	1.53
**1394372**	TMEM156	4.60E-03	34.3	52.38	1.53
**1383168**	ZNF202	4.42E-03	33.17	50.61	1.53
**1397248**	JOSD2	3.12E-04	54.29	82.8	1.53
**1394073**	VASN	6.02E-03	263.18	401.36	1.53
**1384057**	FES	2.89E-03	113.65	173.31	1.52
**1387117**	PLAGL2	3.83E-05	93.77	142.91	1.52
**1384604**	TMPRSS5	8.22E-03	17.18	26.18	1.52
**1387379**	SSX9	3.05E-03	13.98	21.3	1.52
**1394876**	PKN1	1.32E-03	29.38	44.76	1.52
**1389433**	HDAC8	1.81E-03	71.66	109.13	1.52

**1389702**	RBM38	5.62E-03	25.81	39.3	1.52
**1378998**	TNPO3	2.23E-05	334.38	508.79	1.52
**1385356**	CSRP1	4.42E-03	2089.55	3178.66	1.52
**1383290**	ROM1	5.80E-04	76.64	116.57	1.52
**1379772**	HPS4	4.80E-05	45.56	69.26	1.52
**1385588**	DYSF	8.84E-03	188.11	285.91	1.52
**1391708**	WDR6	2.22E-05	974.15	1480.58	1.52
**1389198**	FGD1	2.95E-04	117.64	178.79	1.52
**1396617**	ITIH4	3.89E-03	51.14	77.72	1.52
**1381234**	PHLDB1	8.88E-03	698.54	1061.6	1.52
**1387039**	IFNAR2	6.57E-04	126.96	192.94	1.52
**1384882**	IL10RB	4.91E-04	242.09	367.72	1.52
**1384917**	MGC20983	4.79E-05	31.66	48.05	1.52
**1382723**	DVL2	5.02E-04	89.71	136.11	1.52
**1394978**	ANKZF1	2.68E-04	51.64	78.32	1.52
**1380207**	FAM20C	4.12E-04	148.04	224.47	1.52
**1646148**	AP1B1	6.85E-03	43.45	65.85	1.52
**1378699**	PIP5K1C	1.26E-03	812.41	1230.97	1.52
**1382187**	ZNF324	5.97E-04	196.06	297	1.51
**1385316**	CNKSR3	8.14E-03	111.29	168.5	1.51
**1646024**	BCL2	5.77E-03	206.53	312.59	1.51
**1379134**	BAT2	1.55E-04	240.84	364.48	1.51
**1389495**	IMPA2	1.43E-03	61.93	93.72	1.51
**1382968**	STIP1	1.00E-04	741.01	1121.36	1.51
**1385814**	ROBO3	1.07E-03	99.39	150.38	1.51
**1382704**	BCL2L12	2.32E-03	25.47	38.51	1.51
**1395125**	FAM164C	8.50E-03	14.09	21.3	1.51
**1388632**	LRFN4	8.63E-03	79.44	120.04	1.51
**1386237**	PLEC1	1.92E-03	95.61	144.4	1.51
**1377154**	DHX34	1.25E-03	78.74	118.92	1.51
**1392325**	PLCD3	9.89E-03	33.71	50.89	1.51
**1393092**	SH3BGRL3	2.74E-03	348.31	525.76	1.51
**1396972**	STAT5A	8.55E-03	56.91	85.85	1.51
**1378852**	EMP3	2.55E-03	272.64	411.28	1.51
**1388022**	CAPS	8.72E-03	17.88	26.97	1.51
**1390804**	SIGIRR	1.46E-03	40.04	60.39	1.51
**1393941**	SLC25A37	1.52E-03	261.11	393.78	1.51
**1395512**	PITPNC1	1.91E-03	92.57	139.59	1.51
**1379808**	WNK1	5.39E-03	115.17	173.6	1.51
**1393030**	NFIB	2.89E-04	925.11	1394.18	1.51
**1378787**	ATP1B2	5.49E-03	874.07	1317.25	1.51
**1392147**	RHOG	4.30E-03	266.36	401.4	1.51
**1390013**	DAXX	1.11E-04	65.88	99.23	1.51
**1380482**	CEP135	1.55E-04	66.28	99.82	1.51
**1380302**	SCARB1	9.29E-03	201.32	302.88	1.50
**1379509**	FGD3	4.25E-03	51.41	77.33	1.50
**1378076**	RGS11	5.56E-05	536.7	806.88	1.50
**1396439**	C15orf52	4.48E-03	184.06	276.71	1.50

**1643667**	MYO1C	8.61E-03	7.81	11.74	1.50
**1384527**	MXD4	6.31E-04	1339.9	2014.13	1.50
**1397226**	ZNF175	7.44E-04	100.87	151.61	1.50
**1383818**	PTDSS2	3.98E-05	138.01	207.42	1.50
**1387693**	HIC2	1.25E-05	186.21	279.79	1.50
**1385342**	STK10	8.05E-03	20.05	30.12	1.50
**1387622**	CNTROB	2.84E-04	22.9	34.4	1.50
**1395421**	LZTS2	8.31E-04	50.52	75.89	1.50
**1392912**	SAP30BP	6.72E-04	92.04	138.23	1.50
**1395193**	EML3	6.51E-03	325.83	489.26	1.50
**1378314**	SEZ6	9.02E-03	163.6	245.49	1.50
**1644176**	KATNAL2	8.42E-04	49.23	73.86	1.50
**1379300**	SNIP	8.08E-03	3.06	0.27	0.09
**1378185**	TPD52	3.31E-03	37.74	5.06	0.13
**1384366**	MORF4L1	2.60E-06	22.81	3.16	0.14
**1384259**	SETD6	8.76E-03	4.89	0.72	0.15
**1382946**	NMU	2.99E-03	18.96	3.15	0.17
**1645670**	TAC1	1.52E-04	176.79	32.92	0.19
**1391267**	EDARADD	6.39E-03	3.41	0.64	0.19
**1391148**	SST	1.04E-04	630.88	133.72	0.21
**1382605**	FAIM	1.85E-04	7.54	1.6	0.21
**1388744**	RTN3	4.10E-06	168.34	37.27	0.22
**1383314**	RRAGB	1.40E-06	43.17	9.68	0.22
**1383364**	ASB6	1.67E-03	9.34	2.12	0.23
**1383406**	MAP1B	2.42E-05	1373.75	321.11	0.23
**1396744**	SYCE1	3.46E-03	24.69	5.81	0.24
**1392696**	FGF22	1.70E-04	11.94	2.87	0.24
**1381672**	RTN1	3.47E-04	2435.69	588.96	0.24
**1381511**	ACOT7	2.58E-03	8.13	2.03	0.25
**1388248**	DKFZp686D0972	8.47E-04	5.67	1.42	0.25
**1395751**	STMN1	3.35E-03	21.7	5.52	0.25
**1397274**	KIAA1524	5.47E-03	3.76	0.95	0.25
**1391313**	DLGAP1	1.14E-04	43.46	11.96	0.28
**1386183**	MACROD2	4.53E-04	64.48	18.04	0.28
**1393284**	GDAP1	1.70E-06	99.63	28.29	0.28
**1380551**	CSN1S1	9.31E-04	21.67	6.2	0.29
**1378710**	BAAT	3.19E-04	21.92	6.33	0.29
**1389200**	GOLGA8G	1.84E-03	5.17	1.51	0.29
**1384482**	FBXO9	4.80E-06	97.7	28.52	0.29
**1386676**	GOLT1A	5.78E-04	46.49	13.58	0.29
**1378151**	KCNC2	6.80E-04	109.02	32.2	0.30
**1646555**	PRKACB	1.00E-07	193.9	57.99	0.30
**1392346**	SLC25A40	< 1e-07	105.55	31.84	0.30
**1383238**	ZNF596	4.41E-03	7.92	2.4	0.30
**1388064**	ATP6V1H	2.00E-07	38.31	11.74	0.31
**1645918**	SEZ6L2	8.73E-03	8.92	2.73	0.31
**1376918**	HDC	1.41E-04	17.32	5.38	0.31
**1377453**	CRYM	8.66E-05	349.26	109.72	0.31

**1384817**	TRUB1	1.80E-06	71.86	22.68	0.32
**1379872**	SNX14	7.85E-03	5.11	1.61	0.32
**1390709**	FGF13	4.42E-04	27.77	8.82	0.32
**1384214**	HTR5A	7.48E-04	37.42	11.87	0.32
**1384092**	MAGED1	3.13E-03	22.6	7.23	0.32
**1390114**	KRTAP10-1	6.08E-03	4.83	1.55	0.32
**1644519**	FRMPD2L1	6.84E-03	22.54	7.33	0.33
**1387902**	RAB12	4.38E-04	35.93	11.94	0.33
**1394203**	ELMO1	9.68E-05	193.36	64.87	0.34
**1644354**	HAPLN1	1.49E-03	5.43	1.83	0.34
**1646503**	PTPN20A	2.10E-03	111.87	37.68	0.34
**1386633**	MAL2	8.90E-04	817.7	275.33	0.34
**1645380**	VIP	8.55E-04	279.09	94.37	0.34
**1393118**	PNOC	5.40E-04	67.12	22.64	0.34
**1646173**	ANKRD20B	7.99E-03	6.18	2.1	0.34
**1395488**	JMJD2D	7.31E-03	10.05	3.43	0.34
**1394514**	CDC42	5.41E-05	108.98	37.33	0.34
**1381675**	AMACR	1.69E-05	88.35	30.49	0.35
**1378899**	KIAA1033	2.08E-04	7.18	2.47	0.34
**1384533**	HK1	2.97E-04	40.18	13.96	0.35
**1393503**	DYNC2LI1	1.40E-06	270.2	93.82	0.35
**1382854**	NRG1	4.60E-06	35.61	12.4	0.35
**1377551**	VGF	3.26E-04	2746.91	959.64	0.35
**1394136**	EEF1B2	1.59E-04	139.82	49.14	0.35
**1645798**	SNAP25	7.93E-04	5513.37	1936.46	0.35
**1395300**	PRKAR1B	1.25E-03	380.37	133.49	0.35
**1393220**	ECAT1	8.77E-03	9.88	3.48	0.35
**1380538**	NOMO3	6.40E-06	46.09	16.23	0.35
**1385252**	ELAVL2	2.79E-04	189.21	67.21	0.36
**1396369**	WDR69	6.11E-03	53	18.81	0.35
**1644165**	KCNIP1	4.74E-04	7.95	2.82	0.35
**1396358**	TRIM37	1.15E-04	230.18	82.25	0.36
**1376915**	PENK	9.04E-04	121.14	43.27	0.36
**1391600**	PVALB	3.04E-03	1132.23	404.06	0.36
**1394580**	KCNIP4	5.03E-04	350.34	125.59	0.36
**1383057**	SCAMP1	3.00E-06	138.04	49.45	0.36
**1644493**	GABRG2	6.82E-03	940.4	336.79	0.36
**1387095**	SLC32A1	5.26E-04	474.27	171.02	0.36
**1377231**	C9orf72	3.40E-06	33.86	12.31	0.36
**1396482**	ASPH	4.52E-04	78.19	28.52	0.36
**1379547**	CRH	1.83E-03	126.22	46.41	0.37
**1644941**	CTXN3	6.56E-03	156.01	57.55	0.37
**1644753**	EFHB	9.93E-04	26.39	9.82	0.37
**1385975**	UNC5D	4.42E-03	28.48	10.62	0.37
**1644421**	GPM6A	4.87E-05	2099.93	785.43	0.37
**1390880**	FBXW7	1.49E-05	212.86	80.09	0.38
**1389123**	CHGB	1.26E-03	1178.28	443.2	0.38
**1392537**	PSMG1	1.00E-07	172.02	64.95	0.38

**1645227**	C3orf57	1.73E-04	28.79	10.87	0.38
**1644028**	LOC201229	5.39E-05	148.02	55.81	0.38
**1384417**	PCDH8	5.18E-05	57.99	22.03	0.38
**1389485**	BTBD1	1.82E-05	196.98	75.09	0.38
**1643625**	NCKAP1	2.45E-05	203.26	78.02	0.38
**1645348**	BRUNOL4	7.87E-03	123.41	47.2	0.38
**1386448**	NEUROD6	4.35E-04	190.2	73.09	0.38
**1643434**	NOMO2	9.48E-03	16.37	6.32	0.39
**1392288**	MAGED2	2.00E-06	49.62	19.14	0.39
**1392178**	FGF14	4.19E-04	153.6	59.64	0.39
**1645254**	C1QL3	2.72E-04	51.23	19.95	0.39
**1394742**	HNRNPA1	5.00E-07	57.66	22.54	0.39
**1380193**	GABRA1	1.90E-03	490.59	192.73	0.39
**1390147**	CNTN4	4.24E-04	108.29	42.54	0.39
**1646168**	ANKRD56	9.24E-03	9.87	3.87	0.39
**1380165**	RGS12	8.00E-05	29.08	11.47	0.39
**1380032**	CCNH	5.31E-04	94.58	37.25	0.39
**1396667**	KMO	1.58E-03	13.17	5.17	0.39
**1380644**	CNTN1	5.40E-06	55.67	22.12	0.40
**1645135**	CCNL2	3.95E-05	85.56	33.97	0.40
**1644394**	GRIA4	3.07E-04	119.97	47.6	0.40
**1644521**	FRMPD2L2	3.59E-03	27.64	10.96	0.40
**1394862**	PSMG1	< 1e-07	270.29	107.06	0.40
**1397134**	BEX5	2.10E-04	1916	764.47	0.40
**1380649**	TASP1	< 1e-07	104.44	41.64	0.40
**1378095**	PLEKHB2	3.44E-05	358.92	143.03	0.40
**1393588**	SYT1	9.89E-03	3885.62	1545.37	0.40
**1377829**	OLFM3	6.81E-04	138.17	55.57	0.40
**1388812**	RTN3	5.60E-06	95.31	38.22	0.40
**1384151**	CHURC1	2.25E-04	589.16	237.53	0.40
**1388266**	CCT6A	2.60E-06	152.42	61.34	0.40
**1396211**	ENSA	3.30E-06	325.25	131.83	0.41
**1381927**	EFCAB1	5.51E-04	71.17	28.81	0.40
**1386066**	GAD2	2.89E-04	578.14	235.65	0.41
**1389211**	PSMA1	6.27E-05	193.24	78.74	0.41
**1387994**	NRXN1	1.61E-05	209.07	85.84	0.41
**1380389**	RPL41	1.53E-05	63.86	26.19	0.41
**1384834**	ASAH1	5.60E-06	190.55	78.88	0.41
**1397381**	HSD11B1	5.40E-04	86.28	35.67	0.41
**1389673**	UBE1DC1	5.36E-03	18.8	7.81	0.42
**1644104**	KPNA2	3.78E-04	63.14	26.19	0.41
**1376885**	ADCYAP1	3.88E-03	337.3	140.58	0.42
**1393471**	AK5	1.84E-05	67.2	27.96	0.42
**1384585**	PDCD10	4.63E-05	49.68	20.66	0.42
**1391383**	CORT	4.15E-03	138.67	58.09	0.42
**1383790**	GABRA5	3.74E-03	148.52	62.2	0.42
**1382038**	SRPK2	4.10E-06	73.3	30.62	0.42
**1396029**	RFC3	2.78E-05	16.73	7.04	0.42

**1644293**	HS6ST2	3.16E-05	68.59	28.85	0.42
**1645405**	USP14	9.90E-06	225.49	94.84	0.42
**1646728**	PCSK1	1.19E-03	306.17	128.63	0.42
**1382774**	RWDD1	< 1e-07	194.41	81.65	0.42
**1646929**	NR4A1	7.71E-03	10.77	4.52	0.42
**1384978**	RTN1	1.06E-05	72.43	30.56	0.42
**1386269**	CIRBP	4.13E-05	3202.77	1351.31	0.42
**1391782**	MAEL	3.10E-05	49.03	20.79	0.42
**1645009**	CNTN1	1.11E-05	445.99	188.69	0.42
**1388268**	AP1S1	6.47E-04	885.35	377.5	0.43
**1378751**	EID2	5.45E-05	360.35	153.53	0.43
**1383369**	TM2D3	1.00E-07	102.72	43.67	0.43
**1384776**	ZMYM3	3.49E-04	18.77	8.01	0.43
**1380702**	MAEA	8.50E-06	100.59	42.92	0.43
**1393805**	ATP5C1	1.02E-05	764.17	328.56	0.43
**1646297**	ACOT1	3.07E-04	151.76	65.18	0.43
**1383377**	RBM11	6.78E-05	50.09	21.48	0.43
**1381919**	FAM3C	1.04E-04	579.86	250.1	0.43
**1397393**	NETO1	1.00E-06	28.35	12.22	0.43
**1389904**	RAD23B	7.00E-07	426.89	183.94	0.43
**1388504**	PREI3	5.28E-04	71.48	30.75	0.43
**1644783**	DYRK1A	2.60E-05	151.47	65.69	0.43
**1645578**	TMEM70	5.30E-06	30.84	13.35	0.43
**1377573**	STAT4	7.43E-04	628.75	273.44	0.43
**1646458**	RAP1GDS1	2.93E-05	114.15	49.61	0.43
**1390148**	CADPS	2.56E-04	170.01	74.4	0.44
**1381201**	TCP1	1.91E-04	90.13	39.43	0.44
**1394850**	PPP2R2B	4.17E-03	11.43	5	0.44
**1379695**	FLJ42986	2.97E-03	17.41	7.6	0.44
**1384764**	PGAM1	5.58E-05	1615.74	709.5	0.44
**1396666**	PPEF1	3.59E-04	115.75	50.81	0.44
**1387766**	RPS24	6.30E-06	423.94	186.88	0.44
**1645438**	UBE2E3	1.40E-06	285.89	126.01	0.44
**1645074**	CEP170	7.97E-05	21.79	9.6	0.44
**1645132**	CCNC	8.66E-05	194.5	85.68	0.44
**1644158**	KCNQ5	8.46E-03	24.94	11.06	0.44
**1387879**	SNX10	5.32E-03	446.92	197.94	0.44
**1646285**	ACP1	7.12E-05	1110.78	490.69	0.44
**1379809**	SCN1B	3.20E-03	395.32	175.96	0.45
**1393415**	NUDT18	1.00E-06	177.13	78.73	0.44
**1386149**	DMXL2	9.28E-05	216.39	96.02	0.44
**1389714**	FAM174A	6.41E-03	16.87	7.55	0.45
**1383942**	UCHL5IP	7.50E-04	32.66	14.61	0.45
**1395139**	SLC10A4	8.04E-03	21.97	9.82	0.45
**1646602**	PPM1B	5.66E-04	30.46	13.59	0.45
**1384568**	UQCC	2.06E-04	47.23	21.06	0.45
**1644197**	IQWD1	2.14E-03	37.78	16.97	0.45
**1643603**	NHSL2	1.70E-03	11.96	5.37	0.45

**1645141**	CCDC85A	2.04E-04	37.82	17.03	0.45
**1390261**	SLC25A3	1.05E-03	64.31	28.94	0.45
**1393294**	LEO1	1.68E-03	21	9.45	0.45
**1384263**	TSPYL2	1.41E-04	215.14	96.8	0.45
**1396998**	PPM1A	1.30E-06	102.11	45.94	0.45
**1389580**	MAT2B	7.70E-06	30.7	13.8	0.45
**1384744**	OPN3	8.22E-03	367.22	166.51	0.45
**1393994**	DLX1	6.90E-05	432.2	195.6	0.45
**1389253**	ABHD11	1.86E-03	47.85	21.65	0.45
**1396774**	UQCRC2	5.23E-05	288.15	131.22	0.46
**1384538**	HSPB3	8.36E-04	380.31	173.14	0.46
**1646305**	ABHD11	6.60E-06	40.06	18.23	0.46
**1392767**	TRHDE	3.45E-04	19.33	8.78	0.45
**1396933**	GPR89A	1.32E-05	87	39.81	0.46
**1391134**	ATP5G1	5.80E-03	116.78	53.36	0.46
**1390063**	NCOA2	6.80E-06	23.21	10.6	0.46
**1645289**	C17orf91	2.00E-07	112.43	51.33	0.46
**1380607**	RLBP1L1	2.51E-05	53.11	24.24	0.46
**1384806**	GPR155	2.84E-03	10.52	4.8	0.46
**1388938**	HSPC159	7.10E-06	36.36	16.59	0.46
**1396319**	SCP2	1.63E-05	132.41	60.41	0.46
**1380899**	CREG2	5.90E-03	1421.82	648.18	0.46
**1389939**	HMGCLL1	8.30E-06	55.29	25.39	0.46
**1646629**	PMS2	3.00E-07	37.79	17.34	0.46
**1387159**	RCHY1	3.00E-07	127.88	58.54	0.46
**1646886**	OPN3	4.17E-03	93.58	43.21	0.46
**1378419**	TFG	1.20E-04	35.13	16.22	0.46
**1645113**	CD47	1.56E-03	103.84	47.92	0.46
**1397395**	CENTD1	5.96E-03	3.69	1.7	0.46
**1383868**	RPL6	1.07E-04	215.82	99.42	0.46
**1382408**	CLCN4	5.16E-05	72.8	33.53	0.46
**1392960**	RCAN1	1.98E-03	30.25	14.03	0.46
**1396080**	TRIM37	1.57E-03	1096.66	508.6	0.46
**1380351**	TCEAL6	5.79E-05	963.91	446.62	0.46
**1387322**	PPP2CB	2.54E-05	116.71	54.05	0.46
**1644653**	FAM3C	2.22E-04	582.24	269.45	0.46
**1397255**	RPS7	1.68E-03	83.59	38.67	0.46
**1383370**	ACY3	2.10E-03	15.03	6.95	0.46
**1392030**	PHF14	1.15E-04	152.05	70.75	0.47
**1380344**	PPP2R5D	3.65E-04	38.92	18.21	0.47
**1389875**	LRCH1	6.66E-05	37.99	17.77	0.47
**1389700**	ELMOD1	2.57E-03	1911.22	893.69	0.47
**1384312**	MOCS2	6.14E-05	158.89	74.12	0.47
**1395408**	PIP4K2B	1.89E-05	306.97	143.14	0.47
**1390772**	DLD	2.69E-05	282.69	131.81	0.47
**1389598**	DOK5	6.73E-04	44.33	20.86	0.47
**1386401**	TXNDC9	1.04E-04	80.78	37.99	0.47
**1388656**	CHMP5	9.98E-05	644.98	303.31	0.47

**1644062**	LETMD1	3.81E-05	108.65	51.06	0.47
**1385905**	OLA1	1.79E-04	36.55	17.17	0.47
**1645481**	TTC8	3.30E-06	159.69	74.87	0.47
**1397061**	VAMP1	4.31E-03	2937.83	1388.21	0.47
**1395004**	SORCS3	5.38E-04	28.56	13.49	0.47
**1389110**	TOMM20	4.41E-05	2864.4	1348.15	0.47
**1395628**	TMEM70	1.19E-05	150.9	71.01	0.47
**1388689**	ADAR	1.59E-03	16.81	7.98	0.47
**1394739**	GAL	7.42E-03	17.39	8.25	0.47
**1392358**	ATP2B1	3.13E-03	415.52	197	0.47
**1390069**	RAN	2.60E-05	972.53	460.57	0.47
**1382390**	SULT1A1	7.78E-05	203.62	97.09	0.48
**1646512**	PTHLH	8.99E-05	57.54	27.43	0.48
**1380665**	GAD1	2.22E-03	2995.28	1427.8	0.48
**1389807**	NXPH1	1.00E-06	309.74	147.6	0.48
**1645784**	SORD	1.81E-04	7.6	3.62	0.48
**1381566**	SNCA	6.17E-04	941.18	451.1	0.48
**1390049**	GRM7	1.71E-05	25.73	12.32	0.48
**1389358**	TSC22D3	1.19E-03	25.95	12.41	0.48
**1397086**	TSPAN3	3.38E-05	1007.04	481.01	0.48
**1382558**	AMACR	6.54E-05	100.41	48.36	0.48
**1391231**	TATDN1	3.09E-03	47.95	23.06	0.48
**1378791**	DYNC1I1	2.65E-03	1574.57	757.1	0.48
**1645392**	USP33	3.00E-07	79.46	38.19	0.48
**1383598**	WAC	1.81E-05	28.11	13.5	0.48
**1394413**	CHM	1.21E-03	31.72	15.23	0.48
**1392824**	MATR3	1.74E-05	205.11	98.39	0.48
**1393995**	CDC42SE2	1.33E-03	49.93	24.12	0.48
**1384952**	ASB3	1.44E-04	134.4	64.88	0.48
**1644243**	IGSF3	6.44E-04	28.97	13.98	0.48
**1646606**	PPCS	1.48E-04	123.34	59.45	0.48
**1385204**	TM6SF1	1.90E-06	377.54	183.6	0.49
**1377197**	SGPP1	1.05E-05	49.71	24.16	0.49
**1395453**	LINS1	5.41E-03	13.47	6.54	0.49
**1395861**	ST6GALNAC5	1.04E-03	101.48	49.18	0.48
**1387840**	B4GALT6	3.34E-03	101.35	49.55	0.49
**1388251**	CITED2	7.78E-05	268.88	131.4	0.49
**1380293**	NAP1L5	8.18E-05	2048.82	1000.57	0.49
**1382858**	PPM1B	3.24E-04	70.55	34.43	0.49
**1386915**	C7orf20	2.40E-06	46.27	22.58	0.49
**1645686**	SYNJ1	5.76E-05	78.33	38.16	0.49
**1386497**	KHDRBS2	1.45E-04	52.05	25.34	0.49
**1392403**	LOC729399	7.40E-04	73.54	35.79	0.49
**1643646**	NAT5	3.65E-05	1093.99	537.54	0.49
**1396459**	VPS16	1.46E-03	34.89	17.14	0.49
**1394339**	SEPT5	2.57E-04	64.22	31.49	0.49
**1381142**	CDKN3	8.29E-04	51.78	25.39	0.49
**1380341**	GNG2	2.87E-03	399.99	196.12	0.49

**1391034**	PPP1R2	1.63E-05	574.25	281.25	0.49
**1645950**	SEDLP	5.78E-04	146.33	71.66	0.49
**1394939**	METTL6	9.87E-04	28.16	13.91	0.49
**1386433**	KCNN2	6.42E-05	48.23	23.81	0.49
**1644901**	DCLK1	5.78E-03	4568.07	2250.26	0.49
**1379619**	ZNHIT3	9.00E-06	89.7	44.13	0.49
**1391215**	SNRPN	9.91E-04	4403.59	2185.23	0.50
**1378328**	HSD11B1	9.48E-04	114.47	56.8	0.50
**1384675**	KCNK1	1.41E-03	279	138.27	0.50
**1380188**	DUSP6	9.18E-04	43.31	21.45	0.50
**1386582**	GRIA3	1.74E-05	72.43	35.83	0.49
**1381809**	F8	2.15E-05	23.5	11.62	0.49
**1388885**	STS-1	2.58E-05	580.28	286.72	0.49
**1383210**	CACNB4	4.67E-04	123.51	61.59	0.50
**1381388**	GNAO1	3.73E-03	310.17	154.65	0.50
**1397081**	PCP4	5.27E-04	2761.72	1375.8	0.50
**1396728**	PIAS2	1.20E-04	159.62	79.48	0.50
**1643869**	LRRTM3	3.21E-05	51.1	25.41	0.50
**1377405**	FASTKD3	2.20E-05	59.79	29.71	0.50
**1377588**	PRDX2	6.32E-05	725.72	360.4	0.50
**1384950**	BCL11A	1.18E-03	142.76	70.86	0.50
**1382563**	CD5	2.53E-03	41.12	20.41	0.50
**1386435**	HISPPD2A	5.62E-03	9.38	4.7	0.50
**1646751**	PCDHA3	1.17E-03	30.15	15.1	0.50
**1382719**	OSTN	8.49E-03	8.19	4.1	0.50
**1377086**	USP16	5.20E-06	139.78	69.94	0.50
**1391901**	KIAA0564	2.94E-04	22.77	11.39	0.50
**1390376**	TMEM35	2.69E-03	361.45	180.79	0.50
**1384183**	TMEM14B	1.93E-05	740.94	370.47	0.50
**1387885**	LYNX1	6.21E-03	37.69	18.8	0.50
**1644331**	HIGD1A	2.25E-05	2039.96	1026.53	0.50
**1388408**	MYL5	1.83E-03	133.02	66.9	0.50
**1391061**	MKKS	6.65E-05	47.09	23.68	0.50
**1645759**	SPIN2B	2.92E-05	61.95	31.15	0.50
**1383335**	STAG3L1	1.79E-04	57.74	29.03	0.50
**1380467**	RAB3C	6.78E-03	56.2	28.25	0.50
**1396881**	KCNA1	4.50E-03	32.56	16.36	0.50
**1387454**	SPAG8	1.24E-03	28.23	14.29	0.51
**1391453**	MAGED2	3.68E-04	45.84	23.19	0.51
**1395476**	CLTC	1.60E-04	48.03	24.28	0.51
**1390986**	PCGF6	3.00E-05	23.21	11.72	0.50
**1646123**	ARID4A	5.40E-05	72.66	36.68	0.50
**1396875**	MBTPS2	1.28E-03	21.77	10.97	0.50
**1396558**	CCT6B	3.41E-03	39.38	19.84	0.50
**1643618**	NETO1	1.20E-04	46.11	23.45	0.51
**1646773**	PARG	7.40E-05	35.28	17.93	0.51
**1396977**	SCN8A	3.79E-04	26.52	13.44	0.51
**1385842**	C12orf24	1.75E-03	707.34	358.36	0.51

**1394037**	C14orf126	5.19E-04	29.1	14.74	0.51
**1646673**	PIAS2	4.00E-07	44.66	22.62	0.51
**1395730**	RIMS1	3.88E-04	114.8	58.14	0.51
**1392687**	SLC37A3	1.33E-04	36.51	18.49	0.51
**1383621**	ATP5S	6.78E-04	50.66	25.9	0.51
**1388953**	TSPYL5	4.70E-06	331.2	169.2	0.51
**1644834**	DLX1	2.29E-05	64.17	32.76	0.51
**1388217**	ACTR3B	1.19E-03	88.55	45.2	0.51
**1378110**	DNAJC19	4.00E-07	286.28	146.09	0.51
**1392246**	KCNMB2	2.56E-03	14.52	7.4	0.51
**1386857**	NRG1	1.93E-05	21.3	10.94	0.51
**1385946**	TRIM23	3.16E-04	542.91	278.8	0.51
**1388667**	KCNH5	2.60E-03	22.88	11.74	0.51
**1378733**	C6orf114	1.32E-04	22.32	11.45	0.51
**1646060**	ATP9A	6.20E-06	35.61	18.26	0.51
**1388130**	USP12	6.93E-03	12.72	6.52	0.51
**1396209**	CCNC	4.25E-05	131.68	67.48	0.51
**1645049**	CHCHD7	1.11E-03	49.32	25.25	0.51
**1643923**	LOC645039	4.01E-04	62.98	32.55	0.52
**1381722**	RASGRF1	1.53E-04	57.43	29.65	0.52
**1390021**	APITD1	5.51E-04	72.69	37.52	0.52
**1392667**	CASC4	4.50E-06	66.77	34.46	0.52
**1389133**	PCSK2	2.15E-03	47.34	24.43	0.52
**1377357**	D4S234E	3.45E-03	3294.2	1699.26	0.52
**1377531**	CCKBR	3.33E-03	298.09	153.74	0.52
**1383095**	AADAT	1.36E-03	29.15	15.02	0.52
**1390019**	TMEM200A	1.88E-03	62.31	32.08	0.51
**1387219**	THYN1	2.44E-03	146.56	75.41	0.51
**1384813**	NLRP6	1.09E-04	7.56	3.93	0.52
**1646680**	PHYH	2.10E-05	157.59	81.85	0.52
**1646521**	PSMC4	2.62E-04	220.06	114.29	0.52
**1643504**	ZNF226	9.52E-04	39.76	20.63	0.52
**1645879**	SLC22A4	4.42E-05	33.54	17.39	0.52
**1383032**	PFAAP5	9.26E-05	319.08	165.39	0.52
**1643648**	NAE1	1.10E-03	147.37	76.26	0.52
**1379524**	LOC400120	3.20E-04	178.41	93.13	0.52
**1387949**	APOL1	1.21E-03	136.71	71.34	0.52
**1383310**	FBLN7	9.74E-03	146.5	76.39	0.52
**1376970**	MED12L	4.04E-03	14.07	7.33	0.52
**1384300**	SUB1	2.35E-05	669.68	348.88	0.52
**1393491**	TARBP1	9.57E-05	276.08	143.54	0.52
**1380760**	GAD1	3.08E-03	36.73	19.28	0.52
**1393565**	KIAA0251	5.70E-05	57.27	30.04	0.52
**1644029**	LOC201229	7.77E-04	125.46	65.77	0.52
**1396992**	P2RX5	7.17E-05	21.6	11.32	0.52
**1395814**	HSD17B11	2.93E-03	32.5	17.03	0.52
**1391465**	EIF5	9.18E-04	61.71	32.33	0.52
**1378701**	ACADSB	3.40E-06	55.75	29.18	0.52

**1388473**	HAS1	1.96E-03	23.12	12.1	0.52
**1643856**	MAEA	1.25E-04	272.03	142.35	0.52
**1377485**	ARMCX5	8.62E-04	64.79	33.85	0.52
**1393606**	SNX12	9.73E-04	38.02	19.86	0.52
**1395872**	MGC4172	3.87E-05	553.16	291.67	0.53
**1383548**	GLRB	9.10E-03	1284.76	676.91	0.53
**1383439**	SNAPC5	7.33E-05	71.9	37.86	0.53
**1377987**	GPATCH4	1.76E-04	189.83	99.92	0.53
**1396106**	GULP1	1.13E-03	43.86	23.07	0.53
**1380703**	TTC32	7.69E-04	164.33	86.42	0.53
**1389541**	TCTEX1D2	6.75E-05	706.91	371.69	0.53
**1377389**	METTL5	< 1e-07	522.3	274.29	0.53
**1392815**	KIAA1967	9.85E-03	21.81	11.45	0.52
**1382226**	RFC2	1.90E-04	34.71	18.22	0.52
**1394263**	TIPRL	4.98E-05	323.2	171.41	0.53
**1378090**	GPRASP2	2.63E-04	800.25	424.27	0.53
**1381046**	CMC1	2.60E-04	133.48	70.74	0.53
**1395793**	PFKFB2	3.50E-04	74.03	39.21	0.53
**1385080**	TUBGCP5	6.48E-05	203.11	107.54	0.53
**1646020**	BEX4	6.04E-05	2019.92	1069.28	0.53
**1645365**	WDR17	1.80E-06	107.46	56.83	0.53
**1397265**	C6orf105	1.39E-03	24.81	13.12	0.53
**1381307**	FGF12	6.22E-04	645.76	341.13	0.53
**1379413**	FGF13	4.81E-03	122.05	65.08	0.53
**1644204**	ISCA1	8.26E-04	238.86	127.33	0.53
**1381257**	PTHLH	2.62E-03	34.22	18.24	0.53
**1390828**	PRPF6	6.00E-04	42.18	22.48	0.53
**1391946**	HNRPK	< 1e-07	1274.62	678.39	0.53
**1392845**	PFN2	3.15E-04	1265.34	673.12	0.53
**1384966**	PPP1R1B	4.40E-04	20.1	10.68	0.53
**1387791**	COPS4	4.53E-05	752.48	399.77	0.53
**1378782**	IFT52	5.89E-04	41.45	22	0.53
**1396914**	CDC42EP3	2.54E-03	33.09	17.74	0.54
**1644297**	HPRT1	2.41E-03	2451.78	1312.06	0.54
**1646035**	BCAP29	1.26E-05	311.32	166.59	0.54
**1643827**	MAP3K7	3.21E-04	70.58	37.74	0.53
**1386838**	NALCN	4.11E-05	66.34	35.45	0.53
**1383250**	SYNCRIP	6.00E-07	397.83	212.51	0.53
**1390427**	ACP1	1.01E-05	927.72	495.36	0.53
**1386064**	HACL1	2.45E-05	520.37	277.68	0.53
**1377516**	SPG21	3.54E-03	28.62	15.27	0.53
**1382399**	GRIN2A	5.37E-04	79.65	42.89	0.54
**1397293**	CASD1	8.10E-06	208.48	112.26	0.54
**1379237**	SLC25A46	2.40E-06	232.49	125.18	0.54
**1646807**	OSBPL3	2.23E-04	155.71	83.83	0.54
**1387569**	RIT2	1.86E-03	238.6	128.32	0.54
**1392291**	ATRX	3.98E-04	25.72	13.83	0.54
**1382048**	PCDHAC1	4.74E-03	9.99	5.37	0.54

**1394534**	MAP2	4.25E-04	295.7	158.88	0.54
**1646104**	ASPH	7.15E-04	99.28	53.33	0.54
**1644440**	GNL3	1.45E-04	393.37	211.09	0.54
**1643949**	LOC653803	6.16E-05	24.51	13.15	0.54
**1643606**	NFU1	< 1e-07	555.47	297.91	0.54
**1377996**	FBXL17	3.35E-03	67.83	36.37	0.54
**1394773**	NME5	8.24E-04	487.34	263.92	0.54
**1381771**	CHMP2A	7.70E-06	135.96	73.57	0.54
**1384535**	DNAJB6	2.56E-04	1461.78	790.83	0.54
**1644041**	LOC134997	7.54E-04	1770.86	958	0.54
**1386996**	C1orf215	5.78E-03	29.89	16.16	0.54
**1392255**	AUH	2.33E-04	474.03	256.2	0.54
**1392638**	C20orf107	1.06E-03	18.18	9.82	0.54
**1382789**	ADK	2.98E-04	56.24	30.32	0.54
**1377072**	C1orf183	6.54E-04	35.49	19.34	0.54
**1394020**	MOXD1	2.14E-03	95.14	51.84	0.54
**1382971**	GABRB2	2.72E-04	11.41	6.21	0.54
**1378012**	ABCA5	2.34E-04	96.1	52.3	0.54
**1396225**	TSC22D1	1.44E-03	76.81	41.77	0.54
**1379166**	C6orf120	4.27E-03	37.97	20.64	0.54
**1381537**	UBLCP1	2.70E-04	280.17	152.29	0.54
**1386727**	CHMP2B	2.30E-06	282.81	153.69	0.54
**1388622**	KCTD16	9.72E-04	70.81	38.47	0.54
**1644020**	LOC344405	6.01E-03	24.63	13.38	0.54
**1376926**	GGH	1.90E-03	29.03	15.76	0.54
**1396325**	RFC3	3.67E-05	41.29	22.41	0.54
**1391792**	NCAM2	1.48E-05	737.76	400.39	0.54
**1393396**	C3orf14	1.33E-03	1916.02	1039.24	0.54
**1383166**	KRT222P	7.20E-03	679.31	372.12	0.55
**1394016**	PPA2	6.90E-04	387.15	212.05	0.55
**1646372**	RNF38	1.52E-04	118.14	64.7	0.55
**1644180**	KCMF1	9.36E-05	264.21	144.52	0.55
**1384839**	CDH13	4.80E-03	435.82	238.19	0.55
**1384022**	CACYBP	8.23E-03	740.04	404.43	0.55
**1390976**	TACR2	7.70E-03	18.72	10.23	0.55
**1396282**	GCC2	2.01E-05	32.52	17.77	0.55
**1386375**	C14orf45	9.00E-03	13.07	7.14	0.55
**1377680**	IARS	1.37E-04	577.11	314.68	0.55
**1646609**	POT1	1.65E-03	36.18	19.72	0.55
**1395786**	FLJ13614	8.33E-03	8.95	4.93	0.55
**1380393**	THAP7	4.06E-04	25.18	13.87	0.55
**1396507**	CCBL2	1.02E-03	101.64	55.93	0.55
**1394170**	NPTN	8.97E-03	511.29	281.11	0.55
**1385249**	CCNDBP1	1.97E-04	39.68	21.81	0.55
**1644931**	CXorf40B	1.80E-04	137.56	75.6	0.55
**1393282**	PAIP2	1.36E-03	37.59	20.65	0.55
**1390985**	PDE4D	4.41E-03	218.29	119.9	0.55
**1390177**	FAM19A1	1.01E-03	343.7	188.73	0.55

**1386292**	HOPX	1.53E-04	22.97	12.61	0.55
**1645391**	USP32	3.90E-04	33.72	18.5	0.55
**1385357**	POP4	1.13E-04	55.15	30.25	0.55
**1382752**	RPA3	1.23E-04	519.83	285.11	0.55
**1380195**	SUCLA2	8.20E-05	850.94	466.65	0.55
**1393661**	RPH3A	3.88E-04	143.09	78.45	0.55
**1390589**	GNAQ	1.61E-04	379.44	208.03	0.55
**1390906**	GRIK2	2.49E-04	74.57	41.31	0.55
**1396788**	PARK2	8.00E-07	34.41	19.06	0.55
**1382182**	SRPK2	2.42E-04	279.6	154.69	0.55
**1378303**	DHDH	7.45E-04	102.16	56.49	0.55
**1380808**	PRND	8.85E-03	7.65	4.23	0.55
**1643720**	MRPL21	8.12E-05	725.93	400.95	0.55
**1644772**	EBI2	9.17E-04	25.45	14.05	0.55
**1384633**	ROPN1L	4.66E-05	62.8	34.66	0.55
**1387979**	CACNB2	5.75E-03	312.62	172.46	0.55
**1378617**	C17orf97	3.64E-05	368.23	202.93	0.55
**1386173**	UGP2	9.39E-05	1101.59	606.99	0.55
**1387448**	TMEM177	8.00E-07	219.67	122.38	0.56
**1396779**	MOAP1	1.61E-03	1990.9	1108.41	0.56
**1646216**	AKIRIN1	5.37E-05	298.91	166.29	0.56
**1386799**	TUSC3	4.45E-03	174.69	97.09	0.56
**1387911**	SLC2A11	2.60E-06	122.99	68.33	0.56
**1393258**	MRPS35	1.01E-05	229.95	127.72	0.56
**1383353**	CHAF1B	3.73E-04	70.7	39.24	0.56
**1377200**	PCNA	1.77E-03	20.76	11.52	0.55
**1390785**	PPP2R2C	2.93E-03	47.46	26.32	0.55
**1645687**	SUSD4	1.01E-03	47.17	26.15	0.55
**1384437**	TRO	2.26E-04	404.83	224.38	0.55
**1382706**	RQCD1	1.73E-04	299.95	167.95	0.56
**1379972**	ACOT4	5.67E-04	141.78	79.34	0.56
**1379537**	KRT81	2.95E-03	18.16	10.16	0.56
**1384563**	AMDHD2	2.68E-04	46.08	25.77	0.56
**1646023**	BCL11A	2.42E-03	284.97	159.31	0.56
**1396926**	C11orf87	7.97E-03	879.48	491.46	0.56
**1382748**	C14orf138	1.23E-04	164.8	92.09	0.56
**1395064**	NRG3	6.48E-04	224.88	125.65	0.56
**1390657**	ACTR10	1.19E-05	872.99	487.61	0.56
**1380975**	MDH1	4.36E-03	11425.32	6376.05	0.56
**1378587**	FKBP3	3.64E-03	285.86	159.37	0.56
**1383702**	PDPK1	5.61E-03	285.56	159.17	0.56
**1389530**	TMEM14A	3.67E-03	1216.19	677.67	0.56
**1388745**	SLC2A11	5.09E-05	72.39	40.74	0.56
**1378945**	MASTL	3.42E-03	12.45	7	0.56
**1394559**	MDM4	1.07E-05	32.39	18.17	0.56
**1646325**	ABCA11	1.43E-04	53.12	29.79	0.56
**1386018**	FATE1	6.12E-03	20.56	11.65	0.57
**1378536**	MRPS30	2.59E-04	507.79	287.58	0.57

**1646379**	RNF14	4.40E-06	397.21	224.84	0.57
**1644498**	G3BP2	4.51E-04	2311.47	1308.37	0.57
**1377963**	MRPL20	2.51E-05	306.15	173.14	0.57
**1378396**	EIF1B	5.28E-04	1012.09	571.92	0.57
**1388901**	CCDC132	8.09E-03	85.99	48.59	0.57
**1396238**	RTCD1	7.33E-04	177.56	100.25	0.56
**1383382**	GLS2	5.55E-03	180.35	101.8	0.56
**1382498**	SMYD3	2.05E-04	342.62	193.39	0.56
**1378737**	NDUFA10	4.44E-04	218.81	123.5	0.56
**1389267**	ALG1	5.41E-04	35.89	20.25	0.56
**1385218**	LARP6	4.99E-03	176.72	99.67	0.56
**1643832**	MAP2	1.30E-04	177.27	99.87	0.56
**1645444**	UBA3	2.78E-04	249.73	142.21	0.57
**1646088**	ATG4B	2.21E-03	296.43	168.69	0.57
**1385405**	FAF1	2.21E-05	102.31	58.21	0.57
**1385983**	MPHOSPH6	7.44E-05	71.07	40.43	0.57
**1646283**	ACOT7	7.24E-03	657.48	373.85	0.57
**1644307**	HNRNPH2	8.25E-04	240.67	136.83	0.57
**1389380**	ZNF707	6.44E-04	23.22	13.2	0.57
**1386785**	STK16	3.83E-03	14.32	8.14	0.57
**1644396**	GRINA	9.30E-06	717.06	407.37	0.57
**1645769**	SPAG8	3.21E-03	21.77	12.36	0.57
**1390539**	TMTC3	7.63E-04	80.26	45.54	0.57
**1386973**	DRP2	2.65E-04	22.96	13.02	0.57
**1393516**	C1orf102	1.07E-05	53.44	30.28	0.57
**1380704**	TAF9	1.86E-03	30.7	17.59	0.57
**1386405**	C6orf106	2.57E-04	57.16	32.75	0.57
**1391132**	NME2	3.86E-03	39.72	22.75	0.57
**1390953**	SARS	7.40E-05	340.56	195.05	0.57
**1383957**	ANO5	5.04E-03	16.12	9.23	0.57
**1378445**	C1orf165	1.20E-06	200.08	114.56	0.57
**1394654**	DLX5	7.59E-03	110.31	63.12	0.57
**1387677**	SNCA	3.89E-03	3863.87	2210.47	0.57
**1380030**	ZFYVE9	7.27E-04	13.55	7.75	0.57
**1390090**	BACH1	2.53E-03	9.18	5.25	0.57
**1378580**	ZNF25	6.27E-04	2069.9	1183.47	0.57
**1394977**	MPPED1	1.63E-03	32.69	18.69	0.57
**1391010**	C18orf19	3.97E-04	41.72	23.85	0.57
**1387413**	PPIA	1.26E-04	65.11	37.2	0.57
**1644192**	ITPA	5.11E-05	146.8	83.86	0.57
**1387919**	NEFL	1.24E-03	2247.19	1283.58	0.57
**1391071**	NME2	1.17E-04	176.51	100.8	0.57
**1379348**	SLC22A18AS	2.11E-03	83.92	47.92	0.57
**1643942**	LOC653566	5.70E-06	306.87	175.21	0.57
**1645318**	C13orf1	7.79E-04	425.32	242.67	0.57
**1382273**	SCOC	4.08E-05	976.99	557.43	0.57
**1384249**	NGEF	6.16E-03	711.97	406	0.57
**1385896**	RAD51C	9.40E-04	202.96	115.72	0.57

**1646522**	PSMD10	1.48E-04	285.27	162.63	0.57
**1382414**	ADH5	1.72E-03	64.28	36.63	0.57
**1386072**	PQBP1	1.40E-04	31.19	17.95	0.58
**1397007**	PREI3	1.98E-05	456.87	262.82	0.58
**1644408**	GRIA3	1.79E-05	130.93	75.28	0.57
**1393934**	B3GALNT1	2.79E-05	27.85	16.01	0.57
**1379201**	ZFPM2	8.21E-04	135.83	78.08	0.57
**1378525**	CXorf56	9.84E-04	68.86	39.54	0.57
**1644279**	HSPBP1	1.98E-03	81.89	47.02	0.57
**1381184**	C14orf124	3.59E-05	108.62	62.34	0.57
**1646829**	OR8B12	4.27E-03	9.76	5.6	0.57
**1389023**	NEGR1	7.49E-03	45.39	26.04	0.57
**1645671**	TAC3	6.11E-03	41.52	23.81	0.57
**1393738**	GNG10	1.09E-03	339.48	194.61	0.57
**1383755**	BFSP1	4.72E-04	34.7	20.12	0.58
**1392840**	ASMTL	2.32E-04	96.33	55.84	0.58
**1382815**	CACNB4	4.49E-03	10.1	5.85	0.58
**1377298**	MRPL15	3.95E-04	433.81	251.19	0.58
**1393305**	ADSSL1	5.66E-04	22.59	13.08	0.58
**1381039**	HTR3B	2.57E-03	24.76	14.33	0.58
**1381382**	ZNF655	1.78E-04	29.22	16.91	0.58
**1384270**	ATCAY	1.22E-03	424.93	245.89	0.58
**1391096**	UTP18	1.16E-05	101.21	58.56	0.58
**1387009**	RABL3	1.13E-03	76.03	43.97	0.58
**1385269**	BTRC	3.83E-03	19.71	11.39	0.58
**1646337**	RPS26L	1.49E-03	925.1	534.34	0.58
**1382010**	BCAT1	2.58E-03	196.16	113.27	0.58
**1396813**	SMPD1	9.01E-05	98.06	56.62	0.58
**1392041**	YEATS4	4.04E-04	110.27	63.67	0.58
**1389208**	PSMA3	9.11E-04	100.27	57.82	0.58
**1382250**	IMMT	1.44E-04	443.63	255.8	0.58
**1389671**	CHCHD6	7.62E-04	448.92	258.79	0.58
**1645403**	UQCRH	1.63E-03	5473.82	3191.65	0.58
**1383879**	PPM1E	4.45E-03	698.55	407.22	0.58
**1646146**	APEX1	9.60E-06	618.3	360.26	0.58
**1393098**	GOLGB1	5.25E-03	94.86	55.25	0.58
**1377319**	C4orf27	1.99E-05	288.25	167.84	0.58
**1384855**	CBWD5	1.63E-04	134.94	78.45	0.58
**1395257**	WDR61	3.23E-05	829.81	482.41	0.58
**1394034**	WDR16	6.80E-04	60.14	34.95	0.58
**1386972**	KLHDC10	5.02E-03	23.32	13.55	0.58
**1643544**	XPOT	5.42E-04	59.1	34.33	0.58
**1377065**	MRFAP1L1	1.20E-03	66.6	38.66	0.58
**1396674**	TSGA14	3.51E-03	352.69	204.68	0.58
**1393330**	MSC	6.24E-04	32.81	19.02	0.58
**1387113**	MFSD8	1.00E-06	96.21	56.42	0.59
**1387504**	ZNF184	8.88E-03	54.35	31.87	0.59
**1380887**	UBE2N	5.79E-05	2104.55	1233.76	0.59

**1381097**	SMYD2	1.83E-04	529.25	310.15	0.59
**1386843**	GDA	6.53E-03	19.87	11.64	0.59
**1381368**	C11orf51	3.60E-06	107.07	62.71	0.59
**1386977**	SCAND1	1.80E-06	117.92	69.06	0.59
**1391692**	UBE2E3	6.30E-06	838.01	490.78	0.59
**1393621**	NDUFAB1	6.24E-04	2033.1	1190.61	0.59
**1379210**	ATP1A1	2.77E-03	2624.35	1536.71	0.59
**1387424**	NPTX2	5.95E-03	1198.12	700.57	0.58
**1381622**	UCHL5	1.80E-03	346.08	202.16	0.58
**1646179**	ANKRD29	2.79E-05	312.11	182.14	0.58
**1387016**	ING3	1.56E-05	33.37	19.47	0.58
**1395767**	DNM1L	3.79E-04	906.89	529.08	0.58
**1383322**	CAMK1G	5.92E-03	637.91	371.97	0.58
**1385523**	C1orf135	3.82E-04	45.82	27.03	0.59
**1377343**	MAD2L1BP	6.22E-04	171.99	101.42	0.59
**1380435**	CRYZL1	2.00E-07	849.87	501.04	0.59
**1388401**	NOV	1.67E-05	303.92	179.16	0.59
**1396212**	NDUFS4	1.76E-04	1796.81	1058.7	0.59
**1380626**	TBRG1	3.20E-04	32.74	19.29	0.59
**1382050**	MCEE	9.00E-06	348.05	205.05	0.59
**1389289**	TAC3	2.56E-03	152.2	89.61	0.59
**1645176**	CAMK2G	9.47E-04	663	390.33	0.59
**1390733**	MAK16	5.29E-04	99.74	58.7	0.59
**1644383**	GTPBP8	7.70E-06	99.47	58.51	0.59
**1646117**	ARMC10	1.59E-05	625	367.54	0.59
**1389869**	COX7B	6.86E-04	718.55	422.55	0.59
**1395677**	NRXN1	6.58E-03	213.68	125.65	0.59
**1380222**	FLJ20125	1.76E-04	43.95	25.83	0.59
**1384864**	CYB5D2	1.00E-07	402.6	236.61	0.59
**1386640**	PSMD7	3.58E-05	1121.34	658.87	0.59
**1385194**	CLASP2	1.13E-04	611.99	359.51	0.59
**1385346**	KCTD8	7.60E-03	436.26	256.22	0.59
**1388074**	SLC25A17	1.42E-04	45.88	26.93	0.59
**1385670**	LYPLAL1	1.77E-04	260.37	152.81	0.59
**1386516**	RSPH1	6.64E-04	199.48	117.06	0.59
**1388743**	ATOH7	8.68E-03	30.3	17.78	0.59
**1388340**	IL1RL2	3.59E-03	20.98	12.31	0.59
**1379877**	PEX1	6.48E-05	110.07	64.56	0.59
**1393931**	SERF1A	8.84E-04	24.5	14.37	0.59
**1382083**	ATP6V1A	2.24E-03	5770.9	3424.79	0.59
**1645655**	TATDN3	7.13E-04	159.43	94.61	0.59
**1378432**	CUL3	2.19E-03	108.93	64.62	0.59
**1377603**	SFTPD	3.00E-06	129.74	76.93	0.59
**1393293**	UBE1DC1	9.50E-06	94.78	56.19	0.59
**1392321**	C2orf47	5.30E-05	295.58	175.21	0.59
**1644388**	GSTZ1	2.00E-05	91.22	54.06	0.59
**1388219**	UQCRFS1	1.13E-04	3551.17	2103.37	0.59
**1645316**	C14orf19	7.33E-03	7.97	4.72	0.59

**1379268**	HECW1	1.53E-03	48.18	28.52	0.59
**1644237**	IHPK2	1.65E-03	85.6	50.66	0.59
**1388358**	HINT1	1.05E-04	6296.41	3724.12	0.59
**1395216**	MDH1B	6.38E-05	34.22	20.23	0.59
**1397228**	APITD1	3.49E-05	237.89	140.56	0.59
**1383478**	MPO	5.18E-03	33.61	19.85	0.59
**1378028**	CCDC25	3.60E-03	122.51	72.34	0.59
**1382617**	C4orf30	2.30E-06	171.02	100.98	0.59
**1380547**	PIP5K1B	2.34E-03	43.24	25.53	0.59
**1391147**	ITGB1BP1	1.97E-04	776.89	458.63	0.59
**1381549**	NFS1	5.43E-04	64.97	38.35	0.59
**1396279**	MTUS1	2.96E-04	18.59	10.97	0.59
**1390410**	TDRD9	8.51E-03	135.21	80.71	0.60
**1389647**	ALDOA	2.18E-04	4505.5	2689	0.60
**1393239**	C3orf10	5.05E-05	721.37	430.28	0.60
**1385838**	ME3	2.73E-05	201.71	120.28	0.60
**1382702**	AMN1	1.23E-03	132.45	78.98	0.60
**1377394**	NDUFB6	2.81E-03	937.99	558.96	0.60
**1386157**	FAM162A	7.94E-03	126.83	75.56	0.60
**1382710**	FLJ20628	4.36E-03	89.69	53.43	0.60
**1382112**	ANKRD13C	6.00E-07	144.45	85.99	0.60
**1381882**	GSTM4	3.07E-04	35.97	21.41	0.60
**1646252**	ADD2	2.64E-03	16.42	9.77	0.60
**1645247**	C20orf7	1.52E-04	83.58	49.73	0.59
**1387850**	SLC7A4	4.36E-03	68.81	40.93	0.59
**1395474**	NF2	1.87E-03	17.35	10.32	0.59
**1377151**	ICA1L	1.28E-05	43.28	25.72	0.59
**1383022**	BRWD1	2.11E-03	1128.76	670.78	0.59
**1386234**	MDP-1	4.80E-06	195.95	116.44	0.59
**1390439**	GSTA4	3.58E-05	569.87	338.61	0.59
**1389381**	GNB5	4.00E-04	251.63	149.48	0.59
**1388468**	SRP72	2.56E-05	176.01	104.54	0.59
**1388335**	AMD1	9.31E-04	402.13	241.29	0.60
**1382560**	LIN7B	9.63E-04	604.96	362.99	0.60
**1393653**	VPS29	1.90E-04	1325.08	794.97	0.60
**1387715**	PIK3CB	2.10E-03	143.13	85.85	0.60
**1388788**	C7orf28A	4.83E-04	62.19	37.3	0.60
**1646688**	PGK1	2.90E-05	435.23	261.01	0.60
**1388116**	PTCD2	1.90E-04	152.67	91.54	0.60
**1393360**	PAIP2	1.24E-04	1293.81	775.72	0.60
**1396547**	APOO	5.30E-04	531.14	318.41	0.60
**1645878**	SLC22A18	4.43E-04	107.18	64.24	0.60
**1377057**	POLR3C	5.04E-05	382.12	228.99	0.60
**1645598**	TIMM23	5.11E-04	750.85	449.95	0.60
**1392023**	TUBB3	3.93E-03	2978.08	1784.07	0.60
**1379747**	PSPC1	1.85E-04	80.69	48.33	0.60
**1376883**	MAP1B	6.40E-03	2273.43	1361.18	0.60
**1379708**	ENTPD3	6.49E-03	164.46	98.43	0.60

**1395426**	NDUFC2	4.98E-04	70.55	42.22	0.60
**1378066**	C2orf25	2.50E-06	591.15	353.66	0.60
**1390103**	GLE1	8.27E-05	36.28	21.7	0.60
**1386871**	UBE2H	8.13E-03	29.06	17.38	0.60
**1391830**	MAPK9	2.09E-03	642	383.86	0.60
**1645016**	CLSTN1	2.78E-03	190.76	114.01	0.60
**1391287**	JAZF1	8.62E-03	263.77	157.64	0.60
**1395332**	ATP6V1C1	4.07E-03	323.71	193.46	0.60
**1391713**	TMEM126A	1.83E-04	853.97	510.31	0.60
**1377017**	HRASLS	3.34E-05	101.51	60.63	0.60
**1386801**	ATPIF1	1.49E-04	960.91	573.89	0.60
**1379133**	PRPS1	2.69E-04	605.87	365.94	0.60
**1646072**	ATP5A1	3.39E-04	7639.38	4613.99	0.60
**1391068**	FGF9	9.17E-04	699.45	422.44	0.60
**1385603**	MRPL35	1.50E-04	164.99	99.61	0.60
**1389605**	GPM6A	1.66E-04	325.5	196.46	0.60
**1378510**	ABCC12	7.10E-03	160.96	97.14	0.60
**1644583**	FKBP1B	8.88E-03	65.01	39.22	0.60
**1393483**	GOLGA8B	6.33E-03	1537.14	926.85	0.60
**1377350**	RELN	8.84E-03	155.7	93.87	0.60
**1389625**	HSPBP1	1.22E-03	69.65	41.99	0.60
**1380445**	GOLT1B	1.96E-03	86.35	52.05	0.60
**1391763**	VWC2	2.34E-03	74.17	44.7	0.60
**1382769**	ATP5J	3.76E-04	623.24	375.45	0.60
**1645849**	SLC4A8	8.20E-06	44.33	26.69	0.60
**1645178**	CAMKK1	7.33E-03	72.7	43.76	0.60
**1392260**	ATP5L	7.05E-05	474.2	285.43	0.60
**1377830**	IL1RAP	3.67E-03	12.73	7.66	0.60
**1377835**	BEST4	9.70E-03	26.88	16.17	0.60
**1391936**	RNF122	1.30E-03	56.99	34.26	0.60
**1379861**	SNX4	2.47E-04	299.31	179.93	0.60
**1394589**	UBR1	1.56E-04	29.71	17.85	0.60
**1646065**	ATRN	5.01E-03	71.29	42.82	0.60
**1393342**	TRIM32	3.90E-05	155.39	94.39	0.61
**1386211**	PRMT8	1.09E-03	118.58	72.03	0.61
**1387228**	MRPL1	2.48E-03	135.4	82.24	0.61
**1388958**	SPIN2A	2.54E-03	19.99	12.14	0.61
**1389566**	GLRA2	7.68E-03	92.57	56.19	0.61
**1385079**	SPCS1	1.70E-06	2435.68	1477.77	0.61
**1380880**	SGPP2	9.34E-04	22.12	13.42	0.61
**1378916**	HSPD1	3.71E-03	51.56	31.28	0.61
**1382966**	PDE1A	1.92E-03	281.58	170.78	0.61
**1646380**	RNF149	4.28E-03	28.13	17.05	0.61
**1379006**	NGFRAP1	1.13E-03	976.74	592.01	0.61
**1394885**	TXNL2	6.32E-04	323.98	196.25	0.61
**1377702**	CCDC103	7.10E-03	39.39	23.85	0.61
**1391498**	CLIP4	4.00E-05	409.93	248.13	0.61
**1386866**	MLN	6.62E-03	9.6	5.81	0.61

**1382321**	PRMT6	1.78E-04	146.7	88.71	0.60
**1394946**	RBM9	3.04E-03	45.13	27.29	0.60
**1645951**	SEH1L	2.43E-03	157.26	95.08	0.60
**1395191**	C8orf38	8.58E-05	91.94	55.56	0.60
**1378651**	GNG3	2.14E-03	1605.26	970.01	0.60
**1378070**	C3orf26	2.51E-03	639.44	391.02	0.61
**1397169**	COG5	8.60E-03	50.83	31.08	0.61
**1395777**	ZNF510	1.63E-03	48.56	29.69	0.61
**1645767**	SPG21	8.89E-04	254.75	155.75	0.61
**1378854**	MAPK10	1.01E-03	1138.43	695.75	0.61
**1643591**	NME1	6.53E-05	170.5	104.19	0.61
**1644169**	KCNIP4	1.40E-03	1923.11	1174.75	0.61
**1384632**	TXNL4A	6.09E-04	89.08	54.41	0.61
**1387586**	C6orf203	7.26E-03	25.31	15.45	0.61
**1393856**	PPM1A	4.28E-04	58.96	35.98	0.61
**1378722**	SSSCA1	4.50E-04	36.55	22.3	0.61
**1378873**	DHX9	3.56E-05	99.68	60.81	0.61
**1389027**	ARMC8	1.12E-03	175.36	106.96	0.61
**1382824**	RMND1	1.21E-04	68.54	41.8	0.61
**1644713**	ERP29	1.18E-03	261.75	159.44	0.61
**1388029**	BAIAP2L2	3.06E-03	536.53	326.76	0.61
**1381194**	ACSL3	4.08E-05	282.53	171.97	0.61
**1389165**	CREBZF	1.20E-03	81.42	49.55	0.61
**1384665**	C12orf54	4.66E-03	28.37	17.25	0.61
**1392850**	TM2D3	1.37E-03	1497.54	910.52	0.61
**1395046**	FAM161B	8.76E-04	34.82	21.17	0.61
**1645210**	C7orf44	1.30E-03	1052.84	647.7	0.62
**1381295**	ANKRD16	1.07E-05	66.7	41.03	0.62
**1389654**	MAP2K1	8.80E-03	1434.42	881.97	0.61
**1381612**	NETO2	1.62E-03	381.72	234.67	0.61
**1393312**	C10orf46	8.96E-04	26.17	16.08	0.61
**1383170**	LIN52	3.53E-05	60.66	37.27	0.61
**1381048**	FEZ1	1.64E-04	771.82	474.18	0.61
**1384540**	TSC22D1	4.97E-03	1775.21	1090.34	0.61
**1385065**	OSGEPL1	6.90E-05	48.25	29.62	0.61
**1390156**	RBP4	7.60E-03	85.82	52.65	0.61
**1644113**	KLHDC9	5.25E-04	301.12	184.68	0.61
**1393367**	EPB41L3	1.86E-03	2040.91	1251.67	0.61
**1381642**	TMEM85	1.03E-04	2033.08	1246.2	0.61
**1377956**	CHST13	2.58E-03	29.28	17.94	0.61
**1645840**	SLC6A1	6.18E-03	607.61	372.23	0.61
**1390838**	RAB2A	5.23E-04	144.38	88.42	0.61
**1396073**	OXCT1	1.51E-03	403.05	246.78	0.61
**1386839**	YME1L1	4.47E-04	219.86	134.52	0.61
**1645326**	C11orf49	7.53E-04	247.96	153.49	0.62
**1388945**	INPP5F	8.32E-04	135.37	83.74	0.62
**1392646**	DTD1	2.28E-05	278.14	172.05	0.62
**1379941**	SUV420H1	7.10E-06	55.04	34.02	0.62

**1395716**	TBC1D23	4.26E-05	57.67	35.63	0.62
**1387417**	KHDRBS1	3.36E-05	1353.66	835.99	0.62
**1391603**	EIF5	3.97E-03	1077.43	665.31	0.62
**1644405**	GRB14	4.97E-03	127.27	78.58	0.62
**1388347**	FAM65B	3.51E-04	194.62	120.16	0.62
**1393537**	C1orf128	1.39E-03	2700.06	1666.76	0.62
**1382490**	C17orf75	7.84E-03	237.74	146.65	0.62
**1644934**	CUL5	6.69E-04	570.28	351.5	0.62
**1387861**	NME1	4.30E-03	1636.06	1007.91	0.62
**1391109**	C9orf103	4.00E-05	550.98	339.31	0.62
**1392561**	THOC4	2.78E-03	50.96	31.38	0.62
**1390206**	SSX2IP	2.20E-03	691.06	425.53	0.62
**1390470**	PNMA1	6.01E-03	613.33	377.5	0.62
**1646527**	PSMA3	1.06E-03	265.15	165.13	0.62
**1646240**	AGGF1	1.15E-05	451.36	280.94	0.62
**1391831**	LDHB	5.67E-04	1508.83	938.33	0.62
**1395532**	PVRL1	1.03E-03	27.21	16.92	0.62
**1391301**	DGKI	1.40E-04	67.33	41.86	0.62
**1386998**	SH3BGR	5.59E-03	95.45	59.34	0.62
**1394052**	DNAJC25	1.64E-04	156.15	97.05	0.62
**1378728**	ANKMY2	4.37E-04	561.59	348.89	0.62
**1643531**	ZC3H15	2.02E-03	322.87	200.53	0.62
**1646019**	BEX2	4.45E-03	3922.75	2435.84	0.62
**1381276**	CTH	1.62E-04	100.96	62.67	0.62
**1383637**	CYP2E1	3.35E-03	197	122.28	0.62
**1384630**	TTC19	4.20E-05	809.01	502.08	0.62
**1380575**	CST9	1.80E-03	76.77	47.63	0.62
**1394261**	AMFR	3.00E-06	64.17	39.81	0.62
**1386700**	C6orf57	5.60E-06	180.93	112.24	0.62
**1393101**	SLC25A26	3.81E-04	146.79	91.05	0.62
**1393029**	ESD	1.09E-03	101.67	63.06	0.62
**1377148**	C3orf31	2.08E-05	262.18	162.6	0.62
**1644819**	DNAJB12	1.98E-05	59.15	36.67	0.62
**1378523**	NCKAP1	1.54E-03	987.86	612.19	0.62
**1643654**	MYO5B	8.13E-03	21.43	13.28	0.62
**1380653**	BCKDHB	1.17E-03	23.69	14.68	0.62
**1385714**	PEX7	6.21E-05	114.3	70.82	0.62
**1390141**	UBE2B	5.53E-04	33.67	20.86	0.62
**1395706**	COMMD10	2.66E-04	234.71	145.34	0.62
**1394709**	TTPAL	8.69E-04	132.62	83.08	0.63
**1646658**	PJCG6	1.46E-04	56.44	35.34	0.63
**1394186**	AGPAT3	3.50E-05	46.43	29.06	0.63
**1383779**	HOXD13	2.63E-03	157.17	98.37	0.63
**1380699**	AZIN1	2.07E-05	963.77	603.08	0.63
**1380526**	ZNF804A	5.27E-03	50.29	31.46	0.63
**1381942**	PPIL1	1.68E-03	221.23	138.38	0.63
**1380505**	JTV1	1.11E-05	472.56	295.48	0.63
**1392424**	PLK4	8.93E-03	39.81	24.89	0.63

**1396669**	PLD3	1.55E-03	32.23	20.14	0.62
**1382060**	LYRM2	1.32E-04	709.81	443.46	0.62
**1378616**	TSPYL4	9.44E-03	503.79	314.69	0.62
**1644605**	FEN1	6.37E-04	444.47	277.56	0.62
**1387716**	EBNA1BP2	7.90E-05	1555.36	971.23	0.62
**1393164**	SLC6A18	7.43E-03	14.67	9.16	0.62
**1645644**	TCEAL1	4.11E-04	305.78	190.93	0.62
**1645590**	TMEM120B	6.13E-04	26.38	16.47	0.62
**1389469**	PFKM	2.28E-04	1126.03	702.95	0.62
**1643607**	NGDN	4.05E-05	232.94	145.32	0.62
**1378071**	RBM18	2.19E-04	107.96	67.35	0.62
**1388710**	ZDHHC15	3.14E-03	21.13	13.18	0.62
**1381769**	SLC25A36	1.13E-04	260.33	162.35	0.62
**1385880**	SLC25A4	7.88E-03	4706.16	2933.35	0.62
**1380862**	FBXO44	5.63E-03	74.02	46.65	0.63
**1389528**	APEX1	2.00E-06	1141.84	719.36	0.63
**1377871**	BLOC1S2	2.71E-04	68.29	43.01	0.63
**1644435**	GOLGA6B	3.57E-03	42.08	26.5	0.63
**1381955**	SCP2	1.41E-03	69.08	43.45	0.63
**1646657**	PJA1	4.94E-03	566.61	356.29	0.63
**1384498**	FBXO3	1.94E-05	140.74	88.49	0.63
**1384019**	BECN1	7.75E-05	332.38	208.98	0.63
**1386044**	OXCT2	5.88E-04	105.97	66.62	0.63
**1383105**	TUBA1C	6.34E-03	8643.45	5432.88	0.63
**1396704**	EXOSC2	2.93E-05	227.69	143.11	0.63
**1395900**	POLR2B	5.39E-04	299.42	188.17	0.63
**1392109**	TXN	6.09E-04	1714.18	1077.25	0.63
**1646016**	BCLAF1	5.84E-05	224.98	141.32	0.63
**1378929**	LCMT2	5.24E-05	127.28	79.94	0.63
**1382085**	HN1	1.63E-03	131.2	82.4	0.63
**1383204**	MYRIP	3.63E-03	323.26	203.02	0.63
**1377808**	LRRC8B	8.85E-04	40.67	25.54	0.63
**1377068**	ADSS	1.22E-05	1246.19	782.18	0.63
**1645175**	CAMK2D	2.71E-03	57.07	35.82	0.63
**1377482**	C12orf4	8.07E-04	196.28	123.19	0.63
**1382211**	C11orf31	6.02E-05	75.71	47.49	0.63
**1394125**	ZBBX	6.64E-03	125.93	78.98	0.63
**1646563**	PRDX3	1.27E-03	1333.52	836.14	0.63
**1391129**	TSGA10	1.64E-03	43.35	27.18	0.63
**1386009**	ARPC4	5.99E-04	11.12	6.97	0.63
**1386188**	LAPTM4B	5.18E-03	905.76	575.01	0.63
**1384171**	NECAP1	8.20E-03	2700.91	1712.94	0.63
**1381127**	GPRC5C	3.03E-04	34.04	21.57	0.63
**1380677**	PCDH20	6.97E-03	263.73	167.09	0.63
**1377733**	CEP68	8.74E-04	180.47	114.31	0.63
**1388777**	ICA1L	5.70E-03	187.31	118.56	0.63
**1382158**	ECOP	8.15E-05	131.06	82.95	0.63
**1393275**	FUNDC1	1.10E-03	279.7	177.01	0.63

**1393049**	CASC4	4.21E-04	394.3	249.49	0.63
**1378911**	ZBTB41	2.48E-03	40.49	25.6	0.63
**1644221**	ING5	5.37E-03	10.93	6.91	0.63
**1393685**	RPAIN	3.14E-04	368.66	233.05	0.63
**1378096**	HMGB3	5.09E-04	115.7	73.11	0.63
**1395881**	HNRNPA0	3.49E-05	1394.31	880.96	0.63
**1643852**	MAGED1	3.78E-04	1550.88	979.82	0.63
**1392066**	IQWD1	8.74E-03	840.67	531.05	0.63
**1386929**	RBBP7	4.25E-03	162.2	102.45	0.63
**1396079**	DHX36	5.98E-04	124.63	78.7	0.63
**1377892**	DLG3	3.88E-04	52.1	32.89	0.63
**1378375**	TMEM18	4.26E-04	68.02	42.94	0.63
**1382329**	NAT6	2.24E-03	118.9	75.03	0.63
**1393421**	ARPC3	6.93E-04	1273.12	803.29	0.63
**1381931**	BTF3L4	3.93E-04	422.57	266.61	0.63
**1395318**	NHP2L1	6.40E-04	2322.88	1465.56	0.63
**1387968**	PAPD4	9.40E-06	278.94	178.21	0.64
**1384718**	UQCRB	5.02E-04	102.35	65.38	0.64
**1380907**	CFL2	3.40E-04	49.38	31.54	0.64
**1391654**	PSMA1	1.35E-04	844.58	539.39	0.64
**1646779**	PANK2	5.19E-05	342.04	218.42	0.64
**1377936**	SNX25	1.62E-04	176.93	112.98	0.64
**1385333**	SLC25A5	1.58E-03	4427.72	2827.06	0.64
**1392604**	KCMF1	1.34E-03	57.07	36.42	0.64
**1386238**	SC5DL	1.02E-03	1510.86	963.98	0.64
**1389131**	CORO2A	2.61E-04	72.51	46.23	0.64
**1644065**	LGALS8	9.67E-03	122.22	77.92	0.64
**1385520**	ZMYM5	1.77E-05	53.31	33.97	0.64
**1395915**	DNM1L	6.71E-03	107	68.17	0.64
**1384107**	OCIAD1	2.77E-04	2800.74	1784.13	0.64
**1389661**	MAGOH	3.22E-04	117.88	75.09	0.64
**1386721**	ALS2CR8	1.08E-04	31.07	19.79	0.64
**1377360**	CXorf40A	7.90E-06	359.17	228.7	0.64
**1384197**	SEC61G	4.75E-03	1656.99	1055.06	0.64
**1379248**	OPA1	4.48E-03	698.21	444.44	0.64
**1644166**	KCNIP2	2.91E-03	26.08	16.6	0.64
**1397357**	ATP5O	4.80E-04	3557.91	2264.1	0.64
**1394110**	CDH9	3.03E-05	45.56	28.99	0.64
**1396687**	NIT2	1.04E-04	207.11	131.77	0.64
**1377101**	SPA17	6.51E-04	220.28	140.11	0.64
**1645966**	SCRN3	2.98E-03	37.56	23.89	0.64
**1377267**	CD2BP2	3.62E-04	768.83	488.71	0.64
**1393787**	MAN1A1	1.67E-04	144.55	91.82	0.64
**1382126**	BTBD10	8.25E-03	559.16	355.13	0.64
**1378693**	PSEN1	7.37E-04	22.38	14.21	0.63
**1386512**	PMS2	3.04E-03	49.28	31.69	0.64
**1646687**	PGBD4	1.78E-03	21.68	13.94	0.64
**1380296**	NRN1	2.68E-03	144.46	92.78	0.64

**1385386**	ARL9	5.73E-03	69.29	44.5	0.64
**1391592**	YWHAB	9.58E-03	212.88	136.69	0.64
**1387661**	TMEM69	2.35E-04	262.41	168.44	0.64
**1383044**	CYP4A11	3.93E-03	36.81	23.62	0.64
**1381926**	BHLHB9	2.89E-03	283.39	181.84	0.64
**1394072**	CMTM4	2.35E-03	26.92	17.27	0.64
**1391377**	TPM3	1.09E-04	592.93	380.37	0.64
**1390056**	RPS26	9.03E-03	1654.51	1061.15	0.64
**1386579**	AGK	1.98E-03	559.66	358.93	0.64
**1645973**	SBDSP	8.69E-05	192.8	123.58	0.64
**1392901**	ARMCX3	1.61E-03	566.58	363.02	0.64
**1385189**	RANBP1	2.05E-03	478.52	306.37	0.64
**1389455**	FBXO32	2.16E-04	160.65	102.82	0.64
**1378055**	KIT	5.84E-03	257.44	164.61	0.64
**1387541**	NOLC1	1.30E-03	102.7	65.66	0.64
**1646556**	PRKAG1	3.84E-05	187.17	119.65	0.64
**1391771**	SUMO2	1.94E-04	846.15	540.81	0.64
**1377790**	TRMT11	5.49E-04	130.65	83.49	0.64
**1383389**	NKIRAS1	3.75E-03	1362.26	881.62	0.65
**1646074**	ATP5J	3.34E-03	5585.71	3614.25	0.65
**1394296**	C20orf7	1.22E-03	485.56	314.17	0.65
**1397337**	ABHD7	5.18E-03	147.43	95.38	0.65
**1644643**	FAM86A	1.16E-03	20.73	13.41	0.65
**1380360**	PARP6	3.33E-03	49.12	31.77	0.65
**1382239**	ADARB1	3.28E-03	44.67	28.88	0.65
**1382354**	ANAPC10	1.48E-04	173.57	112.18	0.65
**1387195**	CCT7	1.03E-04	393.66	254.36	0.65
**1393636**	C1orf149	5.99E-04	410.96	265.51	0.65
**1394208**	ATP5F1	3.17E-04	1409.85	910.8	0.65
**1393137**	XRCC5	6.50E-04	303.37	195.91	0.65
**1390031**	ISCU	6.29E-04	762.88	492.47	0.65
**1380906**	IHPK2	9.23E-04	137.12	88.44	0.64
**1392259**	DENND1A	7.26E-04	24.22	15.62	0.64
**1388714**	PPP4R4	8.38E-03	15.74	10.15	0.64
**1386703**	CCNDBP1	5.68E-04	1113.85	718.02	0.64
**1384256**	NDUFA12	2.96E-03	2338.95	1507.02	0.64
**1390881**	MRPS23	1.16E-04	205.66	132.47	0.64
**1394822**	CCDC148	6.49E-04	19.84	12.77	0.64
**1380741**	MRPL39	6.37E-04	344.6	221.64	0.64
**1376959**	FAIM	2.62E-05	57.25	36.82	0.64
**1382646**	RAD51C	1.33E-03	257.32	167.57	0.65
**1390290**	PLEKHA3	1.49E-03	140.27	91.34	0.65
**1644648**	FAM21A	3.01E-04	34.65	22.56	0.65
**1383517**	PMPCB	5.00E-04	800.65	521.27	0.65
**1645341**	BTBD1	3.34E-04	255.46	166.29	0.65
**1384694**	MAGED1	6.27E-04	5097.99	3318.44	0.65
**1646170**	ANKRA2	2.81E-03	171.17	111.38	0.65
**1381898**	NRXN3	4.43E-03	24.93	16.22	0.65

**1380318**	USP33	2.13E-03	153.1	99.6	0.65
**1378953**	WWP2	1.72E-03	179.71	116.9	0.65
**1388619**	C17orf80	1.45E-03	64.98	42.23	0.65
**1378885**	TMEM55A	1.19E-04	450.91	293.02	0.65
**1378585**	C1orf59	7.42E-03	573.75	372.81	0.65
**1383556**	SUDS3	5.89E-05	18.04	11.72	0.65
**1381274**	THYN1	6.68E-05	698.55	453.82	0.65
**1381862**	TMED4	5.00E-07	556.95	361.72	0.65
**1380714**	CISD1	6.63E-04	3497.36	2270.45	0.65
**1645542**	TOX3	1.37E-03	22.56	14.64	0.65
**1387664**	MCART1	2.11E-05	23.81	15.45	0.65
**1381468**	RPP40	7.24E-05	418	271.21	0.65
**1395380**	ICA1	3.05E-03	526.07	341.29	0.65
**1385136**	TADA1L	8.85E-04	212.14	137.62	0.65
**1380100**	JAK1	1.73E-03	263.33	170.79	0.65
**1392236**	C1orf19	2.10E-06	454.74	294.9	0.65
**1391258**	NUP35	1.18E-04	50.54	32.77	0.65
**1387524**	DUS4L	8.55E-04	173.69	112.58	0.65
**1379683**	DLK2	7.60E-03	99.21	64.3	0.65
**1395293**	CNIH	3.80E-03	446.6	289.34	0.65
**1644678**	FAM10A7	1.32E-03	141.9	91.92	0.65
**1381406**	STXBP5	7.99E-04	216.46	140.18	0.65
**1378021**	NDRG3	5.09E-03	1187.94	768.92	0.65
**1382923**	RTN4IP1	9.01E-04	138.38	90.72	0.66
**1389435**	C3orf38	1.20E-03	126.9	83.19	0.66
**1384715**	MRPL27	5.32E-03	50.6	33.17	0.66
**1645221**	C5orf42	6.49E-03	29.23	19.16	0.66
**1644909**	CYP4X1	2.49E-03	710.31	465.51	0.66
**1395491**	VDAC1	1.61E-03	2773.54	1817.66	0.66
**1644077**	LCMT1	1.36E-03	938.09	614.58	0.66
**1380117**	LGALS8	5.08E-04	145.94	95.61	0.66
**1643565**	WIPF3	8.94E-03	8.38	5.49	0.66
**1390120**	HOPX	9.36E-04	3903.66	2557.33	0.66
**1389593**	C10orf67	5.48E-03	18.09	11.85	0.66
**1383256**	ID2	8.37E-03	642.52	420.84	0.65
**1380767**	C1orf41	2.64E-03	279.2	182.77	0.65
**1396675**	C12orf5	5.38E-03	96.47	63.14	0.65
**1644357**	HAX1	1.78E-04	90.53	59.25	0.65
**1378205**	CMTM1	8.43E-03	58.68	38.39	0.65
**1390876**	LACTB2	8.51E-05	258.86	169.35	0.65
**1381075**	CSNK2A1	1.85E-04	249.3	163.07	0.65
**1383937**	VAPA	1.13E-03	45.27	29.61	0.65
**1392854**	C9orf24	1.36E-05	664.05	434.22	0.65
**1387936**	BMP2K	1.02E-04	38.59	25.23	0.65
**1397180**	SYN3	4.66E-03	18.63	12.18	0.65
**1644593**	FIGNL1	2.72E-03	40.01	26.15	0.65
**1379883**	FH	4.18E-03	727.42	474.55	0.65
**1646167**	ANKRD55	2.03E-03	29.35	19.14	0.65

**1646071**	ATP2C1	4.00E-03	363.35	236.93	0.65
**1645876**	SLC25A15	5.03E-03	18.82	12.27	0.65
**1384193**	FANCF	3.42E-03	20.25	13.2	0.65
**1388091**	MAPK14	9.32E-04	35.21	22.95	0.65
**1391693**	TXNDC15	7.52E-04	111.93	72.95	0.65
**1645625**	TCP1	6.17E-03	101.72	67.13	0.66
**1378504**	PIGH	4.07E-03	114.9	75.82	0.66
**1385230**	LOC198437	5.78E-03	119.75	79	0.66
**1379526**	ALKBH6	1.67E-04	292.85	193.13	0.66
**1392381**	TIMM17A	3.86E-05	47.01	30.99	0.66
**1390956**	MMACHC	7.24E-05	112.65	74.26	0.66
**1379380**	TMEM168	4.86E-04	47.84	31.53	0.66
**1645643**	TCEA1	6.30E-03	24.71	16.28	0.66
**1388718**	MTHFD1L	8.93E-04	217.47	143.25	0.66
**1393192**	CACNA2D1	7.25E-03	15.32	10.09	0.66
**1396391**	SCAND1	1.67E-04	393.08	258.82	0.66
**1386490**	C15orf44	2.76E-04	42.64	28.07	0.66
**1384804**	LYCAT	2.16E-04	140.15	92.26	0.66
**1377247**	VKORC1L1	2.15E-04	342.35	225.17	0.66
**1384704**	RPP25	3.18E-03	74.22	48.8	0.66
**1378883**	SNTG1	6.34E-03	93.12	61.22	0.66
**1394623**	EEF1B2	6.05E-03	1099.38	722.55	0.66
**1394521**	ARL4A	8.89E-03	31.23	20.52	0.66
**1645971**	SCG3	5.18E-03	1272.18	835.85	0.66
**1377600**	BCCIP	2.74E-05	318.91	209.53	0.66
**1393831**	KLHL23	3.60E-06	70.14	46.07	0.66
**1390862**	PAFAH1B1	4.69E-03	1801.82	1183.36	0.66
**1392791**	EXOSC6	6.08E-05	332.15	217.99	0.66
**1391997**	HMBS	5.34E-04	53.37	35.02	0.66
**1394260**	RAB18	1.96E-03	143.7	94.27	0.66
**1643970**	LOC440354	9.98E-03	95.98	62.95	0.66
**1384726**	CUL4B	2.71E-03	27.7	18.4	0.66
**1395219**	VRK3	7.80E-03	53.99	35.86	0.66
**1396610**	C6orf205	7.26E-03	33.24	22.07	0.66
**1645720**	ST3GAL5	5.68E-05	295.6	196.26	0.66
**1380523**	PSMD14	8.77E-04	496.78	329.72	0.66
**1388160**	GABRG3	1.25E-03	20.66	13.71	0.66
**1395386**	AGA	1.19E-03	152.08	100.88	0.66
**1387749**	ZNF425	2.86E-03	78.45	52.02	0.66
**1382160**	C11orf49	3.80E-04	57.59	38.17	0.66
**1387187**	COX6B1	4.47E-03	4113.35	2726.11	0.66
**1378576**	COQ7	1.99E-05	81	53.66	0.66
**1645948**	SEC61G	3.26E-03	1716.58	1137.14	0.66
**1384299**	CUTC	9.51E-05	197.24	130.66	0.66
**1644260**	IDH3B	3.11E-03	1123.66	744.3	0.66
**1643519**	ZFYVE9	1.80E-03	19.13	12.67	0.66
**1644206**	ISCU	1.47E-03	3147.27	2084.09	0.66
**1386925**	MAGEF1	4.48E-03	118.48	78.45	0.66

**1396904**	SLC22A17	6.37E-03	44.12	29.2	0.66
**1394082**	CNOT7	4.26E-05	341.85	226.19	0.66
**1378739**	SLC35E3	1.95E-05	320.27	211.87	0.66
**1376910**	DNAJB14	3.08E-03	208.53	137.95	0.66
**1385256**	CTH	1.01E-03	32.67	21.61	0.66
**1377548**	NDUFS2	6.59E-03	55.77	36.88	0.66
**1379976**	RALB	2.61E-04	2025.38	1338.51	0.66
**1391263**	LMBR1	7.71E-05	620.54	410.08	0.66
**1393297**	C7orf30	2.39E-03	980.58	647.67	0.66
**1388637**	VPS35	2.99E-03	2806.09	1870.32	0.67
**1646652**	PKP2	3.65E-03	32.82	21.87	0.67
**1383732**	HM13	3.75E-03	80.58	53.65	0.67
**1383018**	ATP5J	4.47E-03	5043.85	3357.86	0.67
**1393199**	C1orf74	4.73E-05	63.24	42.09	0.67
**1391859**	MRPS28	2.94E-03	285.68	190.13	0.67
**1389222**	H2AFY	8.47E-04	465.51	309.78	0.67
**1394579**	BOLA3	1.15E-03	1903.6	1266.65	0.67
**1380380**	MRPL21	2.04E-03	762.69	507.42	0.67
**1384693**	DHRS7B	1.38E-04	164.09	109.15	0.67
**1394402**	CD200	2.46E-03	122.67	81.58	0.67
**1397074**	TCEAL5	3.38E-03	37.52	24.95	0.66
**1645442**	U2AF1	9.06E-03	28.86	19.19	0.66
**1396695**	TRIM9	4.99E-04	241.37	160.45	0.66
**1387912**	PHYHIPL	1.04E-03	941.79	625.92	0.66
**1397190**	GBAS	1.14E-04	306.77	203.85	0.66
**1392404**	BRI3BP	7.30E-04	43.63	28.99	0.66
