# Supplementary material for: Meningeal inflammation changes the balance of TNF signalling in cortical grey matter in multiple sclerosis
Source: J Neuroinflammation. 2019 Dec 7;16:259. doi: 10.1186/s12974-019-1650-x (PMC6898969; doi:10.1186/s12974-019-1650-x)
Supplement: Supplementary file 2 — Additional file 2: Table S1. Primary antibodies used for immunohistochemistry/immunofluorescence. Table S2. Complete list of genes differentially expressed between each MS group and CTR samples 2. Table S3. Complete list of 89 Gene Sets significantly modulated in MS samples vs CTR, according to Biocarta Pathway analysis (p<0.05) (significant p-values are in red). Table S4. Complete list of 63 Gene Sets significantly modulated in F+SPMS samples vs F-SPMS, according to Biocarta Pathway analysis (p<0.05) (significant p-values are in red). Table S5. Complete list of 42 Gene Sets significantly modulated in GML vs NAGM samples, according to Biocarta Pathway analysis (p<0.05) (significant p-values are in red). [file 12974_2019_1650_MOESM2_ESM.zip › Suppl Table 3.pdf]

Supplementary Table 3

Complete list of 89 Gene Sets significantly modulated in MS samples vs CTR, according to Biocarta Pathway analysis (p<0.05) (significant p-values are in red)

class 1:CTR, class 2:MS

Biocarta Pathway	Pathway description	Number of genes	LS* permutation p-value**	KS permutation p-value	Efron-Tibshirani's GSA test p-value
h_integrinPathway	Integrin Signaling Pathway	48	0.00017	0.00025	0.005 (+)
h_etcPathway	Electron Transport Reaction in Mitochondria	10	0.00057	0.00299	< 0.005 (-)
h_setPathway	Granzyme A mediated Apoptosis Pathway	21	0.00069	0.08611	< 0.005 (-)
h_tnfr1Pathway	TNFR1 Signaling Pathway	40	0.00175	0.07589	0.015 (+)
h_il6Pathway	IL 6 signaling pathway	26	0.00198	0.02935	0.07 (+)
h_p38mapkPathway	p38 MAPK Signaling Pathway	48	0.002	0.12612	0.205 (-)
h_tsp1Pathway	TSP-1 Induced Apoptosis in Microvascular Endothelial Cell	12	0.00248	0.10586	0.03 (+)
h_epoPathway	EPO Signaling Pathway	25	0.00249	0.01729	< 0.005 (+)
h_il2Pathway	IL 2 signaling pathway	27	0.00252	0.04345	0.04 (+)
h_caspasePathway	Caspase Cascade in Apoptosis	37	0.00358	0.02078	0.015 (+)
h_ceramidePathway	Ceramide Signaling Pathway	33	0.00623	0.02541	0.29 (+)
h_deathPathway	Induction of apoptosis through DR3 and DR4/5 Death Receptors	43	0.00637	0.0285	0.005 (+)
h_fasPathway	FAS signaling pathway (CD95)	44	0.00677	0.16691	0.015 (+)
h_rasPathway	Ras Signaling Pathway	33	0.00751	0.12001	0.145 (+)
h_hsp27Pathway	Stress Induction of HSP Regulation	18	0.0086	0.10261	< 0.005 (+)
h_shhPathway	Sonic Hedgehog (Shh) Pathway	24	0.01273	0.00142	< 0.005 (-)
h_eif2Pathway	Regulation of eIF2	11	0.01445	0.00251	0.01 (-)
h_erkPathway	Erk1/Erk2 Mapk Signaling pathway	44	0.01484	0.05879	< 0.005 (+)
h_mitochondriaPathway	Role of Mitochondria in Apoptotic Signaling	37	0.01554	0.07642	0.14 (+)
h_cdMacPathway	Cadmium induces DNA synthesis and proliferation in macrophages	16	0.01745	0.0286	< 0.005 (+)
h_chrebpPathway	ChREBP regulation by carbohydrates and cAMP	28	0.01921	0.02812	< 0.005 (-)
h_carm1Pathway	Transcription Regulation by Methyltransferase of CARM1	17	0.01936	0.07807	0.01 (-)
h_carm-erPathway	CARM1 and Regulation of the Estrogen Receptor	31	0.02031	0.12029	0.2 (+)
h_stressPathway	TNF/Stress Related Signaling	30	0.02151	0.05632	0.275 (-)
h_pparaPathway	Mechanism of Gene Regulation by Peroxisome Proliferators via PPARa(alp	69	0.02179	0.02152	0.01 (-)
h_il10Pathway	IL-10 Anti-inflammatory Signaling Pathway	14	0.02532	0.0919	0.095 (+)
h_pdgfPathway	PDGF Signaling Pathway	37	0.02565	0.26815	0.17 (+)
h_rarrxrPathway	Nuclear receptors coordinate the activities of chromatin remodeling comp	10	0.02598	0.19384	0.18 (+)
h_vobesityPathway	Visceral Fat Deposits and the Metabolic Syndrome	13	0.0274	0.44017	0.01 (-)
h_akap13Pathway	Rho-Selective Guanine Exchange Factor AKAP13 Mediates Stress Fiber For	15	0.02797	0.14765	0.005 (-)
h_DNAfragmentPathway	Apoptotic DNA fragmentation and tissue homeostasis	15	0.02955	0.24093	0.03 (+)
h_tgfbPathway	TGF beta signaling pathway	24	0.0305	0.06381	0.375 (-)
h_prionPathway	Prion Pathway	15	0.03076	0.21396	0.02 (+)
h_keratinocytePathway	Keratinocyte Differentiation	56	0.03129	0.11962	0.01 (+)
h_gpcrPathway	Signaling Pathway from G-Protein Families	41	0.0315	0.0382	0.015 (-)
h_cell2cellPathway	Cell to Cell Adhesion Signaling	13	0.03336	0.04594	0.185 (+)
h_ngfPathway	Nerve growth factor pathway (NGF)	29	0.03542	0.33504	0.12 (+)
h_il22bppathway	IL22 Soluble Receptor Signaling Pathway	10	0.03557	0.28911	0.23 (+)
h_egfPathway	EGF Signaling Pathway	38	0.03716	0.31139	0.11 (+)
h_bcellsurvivalPathway	B Cell Survival Pathway	27	0.04099	0.12136	0.005 (+)
h_ndkDynaminPathway	Endocytotic role of NDK, Phosphins and Dynamin	24	0.04173	0.0442	0.01 (-)
h_ranbp2Pathway	Sumoylation by RanBP2 Regulates Transcriptional Repression	16	0.04243	0.17982	0.005 (-)
h_ifnaPathway	IFN alpha signaling pathway	11	0.04402	0.16846	0.21 (+)
h_soddPathway	SODD/TNFR1 Signaling Pathway	14	0.04491	0.36741	0.025 (+)
h_il3Pathway	IL 3 signaling pathway	18	0.04552	0.05944	0.035 (+)
h_stat3Pathway	Stat3 Signaling Pathway	9	0.04619	0.13686	0.115 (-)
h_igf1Pathway	IGF-1 Signaling Pathway	29	0.04677	0.23452	0.28 (-)
h_d4gdiPathway	D4-GDI Signaling Pathway	18	0.04922	0.19998	0.295 (+)
h_vdrPathway	Control of Gene Expression by Vitamin D Receptor	30	0.05151	0.02809	0.255 (+)
h_akapCentrosomePathway	Protein Kinase A at the Centrosome	20	0.05539	0.1518	0.005 (-)
h_ranMSpathway	Role of Ran in mitotic spindle regulation	12	0.05817	0.17768	0.02 (-)
h_pcafpathway	The information-processing pathway at the IFN-beta enhancer	15	0.06078	0.03135	0.095 (+)
h_akap95Pathway	AKAP95 role in mitosis and chromosome dynamics	13	0.06111	0.26981	< 0.005 (-)
h_igf1rPathway	Multiple antiapoptotic pathways from IGF-1R signaling lead to BAD phosp	36	0.06522	0.15017	0.02 (-)
h_cftrPathway	Cystic fibrosis transmembrane conductance regulator (CFTR) and beta 2 ad	13	0.07168	0.06385	0.005 (-)
h_plcePathway	Phospholipase C-epsilon pathway	14	0.07264	0.04315	0.005 (-)
h_ck1Pathway	Regulation of ck1/cdk5 by type 1 glutamate receptors	21	0.07427	0.04745	0.015 (-)
h_agrPathway	Agrin in Postsynaptic Differentiation	69	0.07946	0.0894	0.025 (-)
h_dreampathway	Repression of Pain Sensation by the Transcriptional Regulator DREAM	18	0.08712	0.22022	0.01 (-)
h_npcPathway	Mechanism of Protein Import into the Nucleus	15	0.09929	0.23541	0.025 (-)
h_bcrPathway	BCR Signaling Pathway	47	0.09952	0.04403	0.08 (+)
h_rnaPathway	Double Stranded RNA Induced Gene Expression	9	0.10829	0.10812	0.02 (+)
h_mhcPathway	Antigen Processing and Presentation	18	0.10894	0.00765	0.255 (+)
h_barr-mapkPathway	Role of b-arrestins in the activation and targeting of MAP kinases	18	0.11043	0.0373	0.255 (-)
h_parkinPathway	Role of Parkin in the Ubiquitin-Proteasomal Pathway	8	0.1176	0.16009	0.02 (-)
h_pkcPathway	Activation of PKC through G protein coupled receptor	5	0.12113	0.10072	0.045 (-)
h_agpcrPathway	Attenuation of GPCR Signaling	18	0.1259	0.22779	0.025 (-)
h_smPathway	Spliceosomal Assembly	14	0.13917	0.15546	0.045 (-)
h_ranPathway	Cycling of Ran in nucleocytoplasmic transport	6	0.16082	0.41112	0.01 (-)
h_nos1Pathway	Nitric Oxide Signaling Pathway	24	0.16828	0.20998	0.02 (-)
h_vipPathway	Neuropeptides VIP and PACAP inhibit the apoptosis of activated T cells	32	0.17553	0.16821	0.025 (-)
h_crebPathway	Transcription factor CREB and its extracellular signals	36	0.18798	0.15017	0.01 (-)
h_mPRPathway	How Progesterone Initiates the Oocyte Maturation	27	0.19497	0.18344	0.02 (-)
h_appPathway	Generation of amyloid b-peptide by PS1	5	0.20655	0.17319	0.015 (-)

h_lymphocytePathway	**Adhesion Molecules on Lymphocyte**	15	0.20747	0.01409	0.055 (+)
h_badPathway	**Regulation of BAD phosphorylation**	41	0.22991	0.27827	0.005 (-)
h_EfpPathway	**Estrogen-responsive protein Efp controls cell cycle and breast tumors grov**	16	0.23394	0.03586	0.145 (+)
h_p35alzheimersPathway	**Deregulation of CDK5 in Alzheimers Disease**	10	0.23731	0.03173	0.035 (-)
h_actinYPathway	**Y branching of actin filaments**	18	0.27284	0.40098	0.02 (-)
h_sppaPathway	**Aspirin Blocks Signaling Pathway Involved in Platelet Activation**	21	0.27291	0.01927	0.085 (+)
h_GATA3pathway	**GATA3 participate in activating the Th2 cytokine genes expression**	26	0.29502	0.48182	0.035 (-)
h_bArrestinPathway	**b-arrestins in GPCR Desensitization**	21	0.30375	0.0399	0.285 (-)
h_rabPathway	**Rab GTPases Mark Targets In The Endocytotic Machinery**	15	0.3041	0.18942	0.035 (-)
h_ps1Pathway	**Presenilin action in Notch and Wnt signaling**	18	0.30961	0.04712	0.345 (-)
h_salmonellaPathway	**How does salmonella hijack a cell**	10	0.33787	0.59468	0.015 (-)
h_eicosanoidPathway	**Eicosanoid Metabolism**	29	0.48111	0.55017	0.04 (+)

*Tests used to find significant gene sets are: LS/KS permutation test, Efron-Tibshirani's GSA maxmean test,
**The threshold of determining significant gene sets is 0.05, Type of univariate test used: Two-sample T-test
Number of genes used for random variance estimation: 22303
Number of total investigated Gene Sets: 300
