# Supplementary material for: Meningeal inflammation changes the balance of TNF signalling in cortical grey matter in multiple sclerosis
Source: J Neuroinflammation. 2019 Dec 7;16:259. doi: 10.1186/s12974-019-1650-x (PMC6898969; doi:10.1186/s12974-019-1650-x)
Supplement: Supplementary file 2 — Additional file 2: Table S1. Primary antibodies used for immunohistochemistry/immunofluorescence. Table S2. Complete list of genes differentially expressed between each MS group and CTR samples 2. Table S3. Complete list of 89 Gene Sets significantly modulated in MS samples vs CTR, according to Biocarta Pathway analysis (p<0.05) (significant p-values are in red). Table S4. Complete list of 63 Gene Sets significantly modulated in F+SPMS samples vs F-SPMS, according to Biocarta Pathway analysis (p<0.05) (significant p-values are in red). Table S5. Complete list of 42 Gene Sets significantly modulated in GML vs NAGM samples, according to Biocarta Pathway analysis (p<0.05) (significant p-values are in red). [file 12974_2019_1650_MOESM2_ESM.zip › Suppl Table 4.pdf]

Supplementary Table 4

Complete list of 63 Gene Sets significantly modulated in F+SPMS samples vs F-SPMS, according to Biocarta Pathway analysis (p<0.05) (significant p-values are in red)

class 1:F-, class 2:F+

	Biocarta Pathway	Pathway description	Number of genes	LS* permutation p-value**	KS permutation p-value	Efron-Tibshirani's GSA test p-value
1	h_cptPathway	Mitochondrial Carnitine Palmitoyltransferase (CPT) System	7	0.00007	0.00695	0.005 (-)
2	h_PDZsPathway	Synaptic Proteins at the Synaptic Junction	24	0.00102	0.0002	0.01 (+)
3	h_eradPathway	ER¿associated degradation (ERAD) Pathway	19	0.00181	0.14896	0.015 (+)
4	h_plcdPathway	Phospholipase C d1 in phospholipid associated cell signaling	5	0.00247	0.01009	0.025 (-)
5	h_vipPathway	Neuropeptides VIP and PACAP inhibit the apoptosis of activated T cells	31	0.00565	0.02828	< 0.005 (+)
6	h_ranMSpathway	Role of Ran in mitotic spindle regulation	12	0.00598	0.02247	0.145 (+)
7	h_reelinPathway	Reelin Signaling Pathway	11	0.00751	0.18808	< 0.005 (+)
8	h_ranbp2Pathway	Sumoylation by RanBP2 Regulates Transcriptional Repression	16	0.01521	0.00395	0.045 (+)
9	h_RELAPathway	Acetylation and Deacetylation of RelA in The Nucleus	16	0.01919	0.04597	0.03 (-)
10	h_lympathway	Adhesion and Diapedesis of Lymphocytes	21	0.01938	0.41919	0.055 (-)
11	h_cb1rPathway	Metabolism of Anandamide, an Endogenous Cannabinoid	8	0.02048	0.03019	0.035 (+)
12	h_prionPathway	Prion Pathway	15	0.02692	0.32337	0.07 (+)
13	h_aMANpathway	Steps in the Glycosylation of Mammalian N-linked Oligosaccarides	17	0.02767	0.53283	0.1 (-)
14	h_vitCBPathway	Vitamin C in the Brain	15	0.02957	0.13485	0.065 (-)
15	h_il17Pathway	IL 17 Signaling Pathway	21	0.03406	0.24413	0.14 (-)
16	h_bard1Pathway	BRCA1-dependent Ub-ligase activity	13	0.04067	0.00704	0.105 (-)
17	h_amiPathway	Acute Myocardial Infarction	25	0.05309	0.16576	0.015 (-)
18	h_npp1Pathway	Regulators of Bone Mineralization	14	0.05453	0.23183	0.045 (-)
19	h_btg2Pathway	BTG family proteins and cell cycle regulation	13	0.05894	0.03468	0.11 (+)
20	h_erkPathway	Erk1/Erk2 Mapk Signaling pathway	44	0.06057	0.0142	0.14 (-)
21	h_stressPathway	TNF/Stress Related Signaling	30	0.06298	0.02984	0.055 (-)
22	h_plateletAppPathway	Platelet Amyloid Precursor Protein Pathway	17	0.07587	0.07221	0.04 (-)
23	h_soddPathway	SODD/TNFR1 Signaling Pathway	14	0.08014	0.14645	0.01 (-)
24	h_epoPathway	EPO Signaling Pathway	25	0.08772	0.00803	0.065 (-)
25	h_EfpPathway	Estrogen-responsive protein Efp controls cell cycle and breast tumors growth	16	0.08818	0.13709	0.01 (-)
26	h_akapCentrosomePathway	Protein Kinase A at the Centrosome	20	0.09087	0.11783	0.005 (+)
27	h_tsp1Pathway	TSP-1 Induced Apoptosis in Microvascular Endothelial Cell	12	0.09101	0.24773	0.015 (+)
28	h_il4Pathway	IL 4 signaling pathway	16	0.09364	0.08369	0.045 (-)
29	h_deathPathway	Induction of apoptosis through DR3 and DR4/5 Death Receptors	43	0.09574	0.14602	0.015 (-)
30	h_gabaPathway	Gamma-aminobutyric Acid Receptor Life Cycle	11	0.10507	0.02007	0.015 (+)
31	h_p27Pathway	Regulation of p27 Phosphorylation during Cell Cycle Progression	13	0.10876	0.05002	0.005 (+)
32	h_tob1Pathway	Role of Tob in T-cell activation	20	0.11504	0.00545	0.085 (-)
33	h_MITRPathway	Signal Dependent Regulation of Myogenesis by Corepressor MITR	8	0.11663	0.00547	0.095 (+)
34	h_actinYPathway	Y branching of actin filaments	18	0.11816	0.01465	0.105 (+)
35	h_stathminPathway	Stathmin and breast cancer resistance to antimicrotubule agents	25	0.134	0.24243	0.025 (+)
36	h_setPathway	Granzyme A mediated Apoptosis Pathway	21	0.13577	0.06267	0.035 (+)
37	h_melanocytepathway	Melanocyte Development and Pigmentation Pathway	12	0.13722	0.25209	0.03 (-)
38	h_smPathway	Spliceosomal Assembly	14	0.15292	0.40545	0.025 (+)
39	h_il3Pathway	IL 3 signaling pathway	18	0.15534	0.01038	0.045 (-)
40	h_flumazenilPathway	Cardiac Protection Against ROS	9	0.16119	0.0294	0.04 (+)
41	h_keratinocytePathway	Keratinocyte Differentiation	56	0.16246	0.0165	0.235 (-)
42	h_hifPathway	Hypoxia-Inducible Factor in the Cardiovascular System	21	0.17476	0.21481	< 0.005 (+)
43	h_mcmPathway	CDK Regulation of DNA Replication	15	0.17642	0.40971	0.035 (-)
44	h_cdk5Pathway	Phosphorylation of MEK1 by cdk5/p35 down regulates the MAP kinase pathway	15	0.18756	0.017	0.045 (+)
45	h_no1Pathway	Actions of Nitric Oxide in the Heart	37	0.18903	0.02444	0.005 (+)
46	h_HBxPathway	Calcium Signaling by HBx of Hepatitis B virus	10	0.19951	0.02454	0.055 (+)
47	h_eif2Pathway	Regulation of eIF2	11	0.20448	0.02867	0.09 (+)
48	h_p38mapkPathway	p38 MAPK Signaling Pathway	48	0.21572	0.01696	0.31 (-)
49	h_trkaPathway	Trka Receptor Signaling Pathway	23	0.22686	0.02528	0.255 (-)
50	h_fMLPpathway	fMLP induced chemokine gene expression in HMC-1 cells	46	0.2304	0.00748	0.225 (+)
51	h_ngfPathway	Nerve growth factor pathway (NGF)	29	0.25119	0.03354	0.095 (+)
52	h_dspPathway	Regulation of MAP Kinase Pathways Through Dual Specificity Phosphatases	14	0.25205	0.55048	0.04 (+)
53	h_leptinPathway	Reversal of Insulin Resistance by Leptin	19	0.25574	0.01353	0.305 (-)
54	h_agrPathway	Agrin in Postsynaptic Differentiation	69	0.25802	0.01833	0.265 (+)
55	h_Lis1Pathway	Lissencephaly gene (LIS1) in neuronal migration and development	24	0.26563	0.53241	0.02 (+)
56	h_sumoPathway	Basic Mechanisms of SUMOylation	9	0.29817	0.02794	0.045 (+)
57	h_integrinPathway	Integrin Signaling Pathway	48	0.30053	0.04607	0.355 (-)
58	h_intrinsicPathway	Intrinsic Prothrombin Activation Pathway	28	0.3091	0.06005	0.045 (-)
59	h_gpcrPathway	Signaling Pathway from G-Protein Families	40	0.34032	0.03844	0.065 (+)
60	h_pyk2Pathway	Links between Pyk2 and Map Kinases	38	0.36784	0.04802	0.145 (+)
61	h_Ccr5Pathway	Pertussis toxin-insensitive CCR5 Signaling in Macrophage	22	0.38818	0.51836	0.03 (+)
62	h_dreampathway	Repression of Pain Sensation by the Transcriptional Regulator DREAM	18	0.39322	0.15533	0.01 (+)
63	h_eifPathway	Eukaryotic protein translation	22	0.44112	0.13902	0.03 (+)

*Tests used to find significant gene sets are: LS/KS permutation test, Efron-Tibshirani's GSA maxmean test,
**The threshold of determining significant gene sets is 0.05, Type of univariate test used: Two-sample T-test
Number of genes used for random variance estimation: 22303
Number of total investigated Gene Sets: 300
