# Supplementary material for: Meningeal inflammation changes the balance of TNF signalling in cortical grey matter in multiple sclerosis
Source: J Neuroinflammation. 2019 Dec 7;16:259. doi: 10.1186/s12974-019-1650-x (PMC6898969; doi:10.1186/s12974-019-1650-x)
Supplement: Supplementary file 2 — Additional file 2: Table S1. Primary antibodies used for immunohistochemistry/immunofluorescence. Table S2. Complete list of genes differentially expressed between each MS group and CTR samples 2. Table S3. Complete list of 89 Gene Sets significantly modulated in MS samples vs CTR, according to Biocarta Pathway analysis (p<0.05) (significant p-values are in red). Table S4. Complete list of 63 Gene Sets significantly modulated in F+SPMS samples vs F-SPMS, according to Biocarta Pathway analysis (p<0.05) (significant p-values are in red). Table S5. Complete list of 42 Gene Sets significantly modulated in GML vs NAGM samples, according to Biocarta Pathway analysis (p<0.05) (significant p-values are in red). [file 12974_2019_1650_MOESM2_ESM.zip › Suppl Table 5.pdf]

Supplementary Table 5

Complete list of 42 Gene Sets significantly modulated in GML vs NAGM samples, according to Biocarta Pathway analysis (p<0.05) (significant p-values are in red)

class 1:GML, class 2:NAGM

	Biocarta Pathway	Pathway description	Number of genes	LS* permutation p-value**	KS permutation p-value	Efron-Tibshirani's GSA test p-value
1	h_ace2Pathway	Angiotensin-converting enzyme 2 regulates heart function	19	0.0008	0.0068	0.2 (+)
2	h_vitCBPathway	Vitamin C in the Brain	15	0.00116	0.00994	0.05 (+)
3	h_stemPathway	Regulation of hematopoiesis by cytokines	20	0.00377	0.00005	0.07 (+)
4	h_inflamPathway	Cytokines and Inflammatory Response	33	0.00908	0.0035	0.1 (+)
5	h_cytokinePathway	Cytokine Network	20	0.00929	0.02284	0.2 (-)
6	h_plateletAppPathway	Platelet Amyloid Precursor Protein Pathway	17	0.01112	0.05347	0.06 (+)
7	h_npp1Pathway	Regulators of Bone Mineralization	14	0.01165	0.03503	0.115 (+)
8	h_erythPathway	Erythrocyte Differentiation Pathway	17	0.0165	0.00483	0.13 (-)
9	h_atrbrcaPathway	Role of BRCA1, BRCA2 and ATR in Cancer Susceptibility	36	0.01812	0.01297	0.08 (-)
10	h_amiPathway	Acute Myocardial Infarction	25	0.03499	0.13851	0.185 (+)
11	h_tnfr2Pathway	TNFR2 Signaling Pathway	16	0.04039	0.09912	0.015 (+)
12	h_intrinsicPathway	Intrinsic Prothrombin Activation Pathway	28	0.04577	0.18263	0.08 (+)
13	h_d4gdiPathway	D4-GDI Signaling Pathway	18	0.05424	0.04197	0.055 (-)
14	h_AcetaminophenPathway	Mechanism of Acetaminophen Activity and Toxicity	7	0.07651	0.10461	0.03 (+)
15	h_bard1Pathway	BRCA1-dependent Ub-ligase activity	13	0.07838	0.01165	0.045 (+)
16	h_GATA3pathway	GATA3 participate in activating the Th2 cytokine genes expression	25	0.10339	0.03085	0.135 (-)
17	h_asbcellPathway	Antigen Dependent B Cell Activation	16	0.11663	0.2184	0.02 (+)
18	h_bbcellPathway	Bystander B Cell Activation	11	0.11776	0.14478	0.04 (+)
19	h_pitx2Pathway	Multi-step Regulation of Transcription by Pitx2	19	0.17475	0.19953	0.03 (-)
20	h_ucalpainPathway	uCalpain and friends in Cell spread	20	0.18615	0.5543	0.02 (+)
21	h_sumoPathway	Basic Mechanisms of SUMOylation	9	0.25319	0.08801	0.005 (-)
22	h_eicosanoidPathway	Eicosanoid Metabolism	29	0.28164	0.04408	0.21 (-)
23	h_shhPathway	Sonic Hedgehog (Shh) Pathway	24	0.28486	0.84989	0.015 (-)
24	h_wnt-lrp6Pathway	Wnt/LRP6 Signalling	8	0.30872	0.12165	0.01 (-)
25	h_crebPathway	Transcription factor CREB and its extracellular signals	36	0.31411	0.14428	0.005 (-)
26	h_DNAfragmentPathway	Apoptotic DNA fragmentation and tissue homeostasis	15	0.33226	0.63772	0.045 (+)
27	h_IL12Pathway	IL12 and Stat4 Dependent Signaling Pathway in Th1 Development	23	0.33819	0.20352	0.025 (-)
28	h_wntPathway	WNT Signaling Pathway	30	0.3557	0.81674	0.01 (-)
29	h_no1Pathway	Actions of Nitric Oxide in the Heart	37	0.37794	0.77186	0.005 (-)
30	h_tollPathway	Toll-Like Receptor Pathway	34	0.38178	0.2078	0.015 (-)
31	h_mitochondriaPathway	Role of Mitochondria in Apoptotic Signaling	37	0.38417	0.51631	0.02 (+)
32	h_ctbp1Pathway	SUMOylation as a mechanism to modulate CtBP-dependent gene responses	12	0.43641	0.46661	0.035 (-)
33	h_pparPathway	Basic mechanism of action of PPARa, PPARb(d) and PPARg and effects on gene expression	11	0.45362	0.80448	0.03 (-)
34	h_agrPathway	Agrin in Postsynaptic Differentiation	69	0.45551	0.90435	0.045 (+)
35	h_melanocytepathway	Melanocyte Development and Pigmentation Pathway	12	0.46947	0.58405	0.035 (+)
36	h_atmPathway	ATM Signaling Pathway	31	0.50288	0.53151	< 0.005 (+)
37	h_gsk3Pathway	Inactivation of Gsk3 by AKT causes accumulation of b-catenin in Alveolar Macrophages	28	0.59269	0.39437	0.04 (-)
38	h_raccPathway	Ion Channels and Their Functional Role in Vascular Endothelium	20	0.59597	0.65898	0.005 (-)
39	h_chemicalPathway	Apoptotic Signaling in Response to DNA Damage	32	0.66205	0.48376	0.015 (+)
40	h_pparaPathway	Mechanism of Gene Regulation by Peroxisome Proliferators via PPARa(alpha)	69	0.66712	0.40667	< 0.005 (-)
41	h_p53hypoxiaPathway	Hypoxia and p53 in the Cardiovascular system	37	0.76782	0.61249	0.045 (+)
42	h_RNApol3Pathway	RNA polymerase III transcription	8	0.78756	0.7987	0.02 (+)

*Tests used to find significant gene sets are: LS/KS permutation test, Efron-Tibshirani's GSA maxmean test,
**The threshold of determining significant gene sets is 0.05, Type of univariate test used: Two-sample T-test
Number of genes used for random variance estimation: 22303
Number of total investigated Gene Sets: 300
